# Supplementary material for: High-Throughput Identification of Putative Antimicrobial Peptides from Multi-Omics Data of the Lined Seahorse (Hippocampus erectus)
Source: Mar Drugs. 2019 Dec 29;18(1):30. doi: 10.3390/md18010030 (PMC7024384; doi:10.3390/md18010030)
Supplement: Supplementary file 1 [file marinedrugs-18-00030-s001.zip › File S5.docx]

>Neuropep7

MDVFMKGLSKAKEGVAVAAEKTKEGVAVAAEKTKEGVMFVGNKAKDSVGSVAEKTTGAMENIAAATGLLKKDEFPSDMNPEEYGQEAMEGQSEGMLEPEGETYDDTQQAHQCLAQTEGIKQHFGLQSTQTHVVDLAGTKSGAMASLKKSFEGLKGSSHSAVKGVTETAVQAAQDAVQQVADSSKETANAVAQEASKQSQAAIGKAADKATETIKELGQKRETK

>BPTI15

MKHLLLFAIIFAAFHLSYSNVPADDEGFGKKFIIALYYDAANDQCSPFVYKGEGGNGNRFLNERECMKNCSAHIERSYPSDAREACLLKKLGGGCNGNFLRFYYDAVHGRCKKFFWTGCHGNGNRFFDADSCNATCAGVHEEGEEEEEDEPDTPIAIICGVLIGLIVTSIIITIIVLTVQSQPPCARATRPLCGDRDRHTMDLIQLNRECLLHLFSFLDKDSRRNLSLTCTPLRDVFFDARLWNVLHFSSLSQLRGNNFVLGHSLRHLSICWHSSRVLQVCNIEDWLKSSFQRDICSKHDKLVSIFLARVCHMCPNLLSLKLSGCGHISDQDVIILLQRCRKLRRLHLENCVRITDRILDGVAAYGVSLGEVKVDFCRNITQEGLKVVREQRPNLRLSAERSAGMIPDSKPEEKVPLRRTLQKLLEISG

>Hemo4

MVVWTDQERSAITNIMGSLIYEDDGPKALTRCLIVYPWTQRYFGAFGNLYNAEAIKNNPRIGEHGAKILHGLDRALKNMDNIKETYAELSKLHSEKLHVDPDNFKLLADCLTIVIAAKMGSSFNPDIQAAWQKFLSVVVSALGRQYH

>Hemo3

MLSKKEKELLESVWGQLTPAAEYIGADALLRMFTAFPGSKTYFSHLDISPGSNHLYTHGKKIVQAIAEGAQNISRLTETLASLQSLHAYLLRIDPSNFKHFSRCMLVALAVHMGEDFTEVLHAAMDKFLSAFAAVLAEKYR

>Throm50

MFINGEILSTCENTFTVLAELEPQPRYLEDVSERVVGGEVARPNSWPWQISLQYQSGSRFYHTCGGTLIQRGWVMTAAHCVDSRRTWRVVLGEHDLNSNSGREQVMSVSRVYVHPRWNSNNVGAGYDIALLRLSGEASLNSYVQLGSLLPSGNILPHNNRCYITGWGRTSTGGNLSPQLKQAYLPVVGHSTCTSSSWWGGGIKTTMVCAGGGSESGCNGDSGGPLNCLVNGKYYVHGIASFVSGLGCNAPRKPTVFTRVSAYNEWMDSVVLAELEPQPRFLEDDEVEGRVVGGEVARPNSWPWQISLQYKSGSSFYHTCGGTLIKRGWVMTAAHCVDRSRTWRVVLGDHNINSHEGREQYMSVSRVYLHPRWNSNNVAGGYDIALLRLSSEASLNNYVQLGAIPPSGHILPHNNPCYITGWGRTQTGGQLSAQLKQAYLPVVDHRTCSSYGWWGSTVKNSMVCAGGGSESGCQGDSGGPLNCQVNGQYVVHGVTSFVSSSGCNAYRKPTVFTRVSDYISWMNSVSIQLLYIE

>Ixodidin

MLTCHTLPSLEDELPPGKGQEPTSIFDPLVINVNQSIIIISSINISTTKTETSTPTGKGQEPTSIFDPLVINVNQSIIIISSINISTTKTETSTPTGTMQLLLLLHACLVCLVTPNFPTQKSVSEMVVTNFNRETSVEIFLSGDVVFNGALYSEGSFLKLRLGLFESFYLESNFSLSGSELYSQEPVGVIVGFTCVKHLLGDCLYGFSELKPVSLWSYKYFLPPLVDSKTSPSLLLAMTNINSNLDVTTRKEQKSMSLNGGVMKTIPVSALDEVHVISDVPLQLIYYRHDIEQRASTLTALLSVEDICQNGPVSDANVLESVGHYLNTLNKELNPSLCEKKISSCDDLHCGHNKQCSFRNGHPLCVLKTKICSAWGDSYYRNFDGKHFVLLGNCNYTLVQTTCPGINDSTPLQINIARAYLNGAAVSGIYSVEIKIHGFNISMVKGERDQIRVNGHRKNLPVILGNRILRLYPSGQSVVLETFFGVTLQYDWVHNIQVEAHPELHGLVCGLCGNANDSISDDTITSNGTVSKTMDFTLPWALESNTDACMEDCGDGPCPLCSPTQSYPGIMGNPYENNCTLLNRKDGPFSDCHLFIDPEPFVRSCENNLCLGGAASSVCKVFAAYANICQRLGARIQNWRAIAKCHMTCPINSHYEVCGSACQATCGNPDPSHNCGLPCLELCQCYRGYLLSNGKCVLPSECGCVQNGSYFLPTKTFWMDQQCQEKCVCQPQSKTIMCTQSHCQAGEVCMVLNGVIGCHAGRPGLCVAKGYPHYTTFDGRKFEVHGNCSYLLTSHCPAWGDLQDFSIKVQNHVKAGANVSFRHVEMSVAGYSIEMSPEWRSKVMVNGLLLNLPSVLSQGKVMLYLKGQSECIETDFGVVVTYSPDILTVTMPKVFAGNLCGLCENFNDDPQDDLVTDDFDITQAVRYWRTSSEHECWDVPMSTSGCSPQKQDLYQGKEFCGRLMDPLGAFQSCHKIVDPQDFYDNCVYDLCNGNQTTLCQILSHYVAVCQEMGAAVHEWRDSNFCFFICPVNSHYESCGTACPATCEAHLNSFCTEACVEGCQCDPGFLLDGGICITPSQCGCTYNGDHYHSNATFWADESCTQHCICNAYTHQIHCSDASCDEDQYCGIQNGVRSCVQHNYQMCRYTGHRVLTFDKREYNFLGTCRYQLLGICEQLPGLDVITVDVQADAYNQSQMVLVSINGQQVQLDNKNPESIEVNGTKRNMPFSANGTTLVFSMGLHIYVYSTFFFLSISKEGIVVINLSSKYADATCGLCGNFNSDLADDLTVNGTREHLTPEQFGKAWRRGQNHGCVEGCLGGSCPNCSSEALHPFSDPVACGKILEVNGPFRHCHSKVDPSSFYKSCVSDLCLYGDVQPAVCRSLAEYADVCLHHKALVYAWRSSEFCYDACPSSTSYNMSNSPVEICLDWLNLTIQVPPNIGENCLCGAGLVHSGRNCVNPANCGCWYGNSEYILPGQEKSTCEQRCLCHPGGNLTCVDVSCSQEEECTLIGGILGCHPKLKMANCYINGSQLSTFDGHLYEFRGSCNYILVQTCKDLTMEPLLITINGNQSEGKRIYLQFNNMTFMMSTAYPENIQVNEMYENLPFSRNNVTVHQKNEWRTVKVGHLVEIISDFGNYVAVKLPKAYHQTTCGLCGNYNDDPSDDMQLPNGTAVSDPDIFGQSWKLSGSESSCSETCDSTCQRCRSPVPEYTSNLYCGRLTQPKGPFSSCHNLICPQNYYTECLNNLCVAEGQKQALCEALEVYDAACKVAGVNVDPWRNITGCERGQDSCAVKCDKSERCHLSNGEAVCESRPGLCWTWGSQHYHTFDGLSYDVKGTCTYLLSGSRGAAGGATPFLVSKKSDCKEVSSVQLVTVQAYGFVIKFGDKDSVYVNGQVNYIPVTLLWGKIEVSNKEGKTLLKTDFGLRVLFDWNSTILITLDPNYKDIVYGLCGNFNGDTQDDFAIHLHGVRLAKTSVELAQAYRVFDGDHNCCTGCEKNTLDNSTLLEDLSNGIVVTQKSHCDDLIDPAGPFAHCHTHLSSDSFYQSCIADLIQGRSEAFLKQATTSYSVICEELSDYVAPELTIVARCPPNSHYKTCGSACPPTCESKKRVCHSACVQGCFCNPGFINSPEGCVPPKQCGCTDPRGKYHRLNSTFWIPDDCGQLCVCKSGKTKCSSSRCPKGMSCKQLPGKRMCQADKPQNCSIITGMHFTTFDGHSYDFRDSCAYVLVQANTNINGSEPFNITISDASCHKRFFHSFTLTLSIYNLKVVVGKGDSDKVLVNGLQKPLPYSHHTGHFTAYQTPSSLVIHVDMGLQVTVYKTGTVMVGLPSSYESFVRGLCGNANSDPYDDQIMPDEEEAQNTLEFVHSWRLGGAMACRSRCTSRPKNCPANARKRFEGTNFCGILRDEFGPFAECLLVLSPKLYFHNCLADACFYGGHYLALCNSITAFAAACQAAQLPIRHWRSDAFCGMTCPKNSHYELCGPRCPVVCEGLSSATKCSGGCEEGCQCDPGYVLRHDRCVLVADCGCFYEGRYHPAGYFSEPKNCRICYCREGKVTCRPSLCRPKEPFLSSDGLFEPTPRHHGACEVFAGFGYVTFDGLVIRHHGACSFLVSERSPKDIHGHSLLLSFEEGVDDSIFTVSKVEFILAAVEITINPEAPWKIQVNGEDFKVPYSIDVLEAYEDGGRLIITSVAGVSLEVSSTKYLRLSIPQDYNGTTSGLCGNFNGDESDDLQLRSGYLAKNISAFFYDWSIVASGRNCSGSCGKDCGQCTLSAKAETICDILLTTHTEFNHCWNKGVKPKVYRDVCLRAICGGAAYTEAVCLVLEAYMAACQAKGVNVGSWRENSPCSFKCPDRSSPSQCVDYNSNSCPALLHPESSADGCSEGCRCQNGKVFDEGECVPVSQCGCVLHGKYIKIDEQLYSENCTERCWCHPQGGALCEKAACSPGQLCSLKNGYWSCVGREVCQLKDSLQLSTFNGQQLSLAPQTPYQLIGLCDETSKNWFNLISYYGRCDRRGTRLVTVFQILLVGSSIVIQDGIVKVNGHLVPLPYTLPLGVSLSSAMAQDKSEVVVVLTKEAGLESELEMEIGITMATLKAAPWYAGKLCGICGNLSDPHWHTSVKPWALSDFQGW

>Throm6

MYAARYDGHSDRHERIDFLTSKEKAPSKRKLGAVIGILVAVLVLAAVAAFLIWLFVFKDAEEATISRQLKPSVLVFSGHMKLPDVQYQQQLEDTSSPEFEALAVKLEAVLEENLKKDPLLATYYTKSVVTAFSEGVTAYYWSQFDVPVTDLEVVPEFTEELVSETLENGIKEQQRTRSVGQIKISEITATCECFFRLEAEETASEFSSPGYPSSYPPKSRCQWQIRASATNVISITFPFFHVEDDCSDDFVSIYDSLSPDDSQAITEKCGHRPPSNPLEVVSSGNIMLINLITDGETQRPGFQAVYKAIPKLEGKYTNCATYSSLMSLLIFYPPAVDCKWTIKVPAGKKVRVKFNLFRMKEPGVDVRVCNKDYMEIMGKKYCGELSSLALTSTTNTLDVIFHSDESYTDKGFSAEYNAYDPGNPCPDMFSCASGICIARELKCDGWNDCGDMSDEMKCKCDRDQFNCGNGLCKPKLWVCDRVNDCGDGSDEQSCSCEENEWRCGDGTCMPQDVVCDTRTDCEDGSDEASCKTSPGICSHFSFKCKSGDCVNKVNAECDRVDDCADHSDEAGCNCGTRPYKLNRIVGGQNAELGEWPWQVSLHFMTYGHVCGASIISEKWLLSAAHCFVTSDQANHVAANWQTYSGMQDQYRLDDVQRRSVKRIITHPDYNQMTFDYDIALLELNEPLELTNTIQPVCLPAPSHVFPAGMSCTVTGWGALREGGQKAQLLQKASVKIINDSVCNVVTEGQVTSRMLCSGFLTGGIDACQGDSGGPLVCFEESGKWFQAGIVSWGEGCARRNKPGVYTRVTKLREWIKTETGV

>Throm33

MDRVISSEEGNRQAGRQEPRVRSRRRLAGVSSPAQAAARLTLSGVMDTEGRVDESRLRMHIFQNGGVSPTERGLAWRFLFGMYPCSSTALERPLLQEQLTVRYQVMKAKWQNFLPSAVQMHLNGTDAELVAAVGYFNQRQALAQQEADNLSDEVRDRVMFLELQAQILFERVTFDQKELQEAIRIINKDVPRTNRDLSYYQGEGSGNLLVLRDILITYAAFHPEVSYAQGMNDLCSRFLEVMDCEVDSFWSFSCYMEKFAKDFREDGLHRKIELEAALLKELDPQLHVHLVTDSMESFTFCHRWLLLGFQREFEHSDALRLFEILSCDHLELISQRVDRARYQERLAQKYCPDDKCESELRAVNTDFTFELFICAAILLDHKDTLLQCQDEVQLIQFTSRMSKGPAVGIDLGTTYSCVGIFQHGKVEIIANDQGNRTTPSYVAFTDTERLIGDAAKNQVAMNPSNTVFDAKRLIGRKFDDSVVQADMKHWPFKVVNDSTKPKVEVEYKGEIKTFYPEEISSMVLTKMKEISEAYLGKTVTNAVVTVPAYFNDSQRQATKDAGAISGLNVLRIINEPTAAAIAYGLDKKVGSERNVLIFDLGGGTFDVSILTIEDGIFEVKSTAGDTHLGGEDFDNRMVNHFIGEFKRKFKKEINTNKRAVRRLRTACERAKRTLSSSTQASIEIDSLYEGTDFYTSITRARFEELNADLFRGTLEPVEKALRDAKMDKSQIQDIVLVGGSTRIPKIQKLLQDFFNGRELNKSINPDEAVAYGAAVQAAILAGDKSENVQDLLLLDVTPLSLGIETAGGVMTVLIKRNTTIPTKQTQTFTTYSDNQPGVLIQVFEGERAMTKDNNILGKFELTGIPPAPRGVPQIEVTFDIDANGILNVSAVDKSTGKENKITITNDKGRLSKEDIERMVQEAEQFKSEDEAQREKVTAKNALESLAFNLKNTVEDEKLQDKISPEDKKAIIDKCNEVISWLDRNQTAEKDEYEHQQKELEKLCNPIMSKLYQGGTAGGMPGGMPGCGRPSITPQVSGFNKIVNGENAVPGSWPWQVSLQDGRGFHFCGGSLINQQWVVTAAHCRVSPRSHRVILGEHDRQSNQEQLQVKTITRAISHPYYNSQNFNNDITLLKLSSPVQITRTVAPVCLASSSTSIPTGTRCVTTGWGRTGQTSSPRYLQQTSLPILSPTQCKQYWGYNRITDAMICAGASGVSSCQGDSGGPMVCNNGGAWNLVGIVSWGTSNCNVRTPAVYARVSYLRSWIDRTIASN

>Throm15

MKAFILLALFAVAFASPIDDEDDKIVGGYECRKNSVPYQVSLNAGYHFCGGSLISSTWVVSAAHCYKSRIQVRLGEHNIAVNEGTEQFINSARVIRHPRYSSRNLDNDIMLIKLSKPATLNSYVRTVSLPSSCAGSGTRCLISGWGNMSGSGNNYPDRLRCLDAPILSDSSCRNSYPGQITSNMFCAGFLEGGKDSCQGDSGGPVVCNGQLQGVVSWGYGCAQRNKPGVYAKVCNYNSWIRSTMSSN

>Throm16

MKYFIVLALCAAAFAAPIDDEDDKIVGGYECRKNSVPYQVSLNAGYHFCGGSLISSTWVVSAAHCYKSRIQVRLGEHNIAVNEGTEQFINSARVIRHPRYSSRNLDNDIMLIKLSKPATLNSYVRTVSLPSSCAGSGTRCLISGWGNMSGSGNNYPDRLRCLDAPILSDSSCRNSYPGQITSNMFCAGFLEGGKDSCQGDSGGPVVCNGQLQGVVSWGYGCAQRNKPGVYAKVCNYNSWIRSTMSSN

>Neuropep2

MLLVVVLCLLACVHSGIDAYPRQPVTPKEGAPPEEMAKYYSALRHYINLITRQRYGKRDIPDTMFSDVFVKESTETIPGSSYGSCVLDVVFVFIPSAMQQVLDNLKDLPTGSLAKDIDLIFLRGIMESRIVRSLAKAHERLEEVKLEAVRDDNVQLISEILDSLNKLTEKDSATVELAHILQDPHFKSLIEAHDQVAAKCYEMPSVAPLQNGTDALASCLMPADAIRMIGIQKKAGELLGVTFRAERGEMVIARILHGSSIDRQGMLHTGDVIREVNGHQVGGDPHQLQELLRDCSGSVTLKVLPSYKDSPALPQVYLKAHFNYNPASDNLIPCKEAGLAFSKRDILHVVNKDDPNWWQARSVVGGATGLIPSQFLEEKRKAFVRKDCDLSGKGLLCGNLAVKKKKKKMMYLTAKNAEFDRYELQIYEEVTKMPPFQRKTLVLIGAQGVGRRSLKNRLIVMNPLRYGTTVPFTSRSPREEERDGQNYSFVTRHQMERDIKDSRFLEHGEYDGNLYXS

>β2m2

MHTLSSLCLCLVFFHADAQVGYGEIQCQGMSSDDALLTEKIYFNKMLYLQYNSSGGKVIGYTKSGRAIADILNSDAFLKDQKKFLQQCKSWITAAYSTLTKKVEPKVRLRLTEAQADSEHPTTLVCSVYNFYPKDILVTWLKNGEQVTSEVTSTDSLPNGNWLYQRHSYLEYTPTHWDKISCVVEHPSLEKPRIYDWEPTSESVKNKIILGAVGLIVGVVSSSAGLIYYRSNVPDPPQTVIYFADEVLQGEENTLICFVDQFFPPNIRVHWTKNGIEVSEGVSLGRCYPNEDATFRQLSTLTFTPKEGDIYGCTVEHSALDRPKTIFLDTQCMHALLKCQGSSSDGHDASVVEKTYYNKMFYLQYNSTWGNVTGSTKMARKVADILNMYYYLKGIRETLEHCKTWITRAYSILAQKVEPKVRLRLTEAQADSEHPATLVCSVYNFYPKDILVTWLKNGEQVTSEVTSTDSLPNGNWLYQRHSYLEYTPTHWDKISCVVEHPSLEKPRIYDWEPTSESVKNKIILGTVGLIVGLVSFAAGLIYYRRSVPDPPQSVIYLGDELQLDQENTLICLVNHFFPPNIQIHWTKNGVEVSEGVSLSRYYLNEDVTFHQLSPLTFTPKEGDIYGCTVEHSALDRPKTAFLDTQCMHALLKCQGSSSDGHDASVVEKTYYNKMFYLQYNSTWGNVTGSTKIARKVADILNTDFYLKSINESLELCKSWITRAYSILAQKVEPKVRLRLTEAPADSEHPATLVCSVYNFYPKDILVTWLKNGEKVTSEVTSTDSLPNGNWLYQRHSYLEYTPTHWDKISCVVEHPSLEKPRIYDWEPTSESVKNKIILGAVGLIVGLVSFTAGLIYYRRSVPVIVNTWISISCCTDTDLEVWTDNGNELAYTDFIRQEIVYTVPKFLGLDPVTADGIVTYRRSTRNIAMCHRVLVFAEQDVKYPADIEDPPQSVIYLGDELQLDQENTLICLVNHFFPPNIQIHWTKNGVEVSEGVSLSRYYLNEDVTFHQLSTLTFTPKEGDIYGCTVEHSALDRPKTAFLEVEFSHPSIGPDVFCGVGLTVAVTQKHSSSYSSVFCSLAVPTSSRLKVILKSSPVPPGDSRSRWSSVHCSPHFLLSASKDEVTRLPVELSSDGVRAEKWVKDGLHVWEVMWKPNHRGSHAVLGISRQNCPLQASGYNVLVGADSQSWGWELQSNQLWHDRQSRGAYPEKRRICCTKAAENFPSRFSQCSPSTLEVPDAPFPIPERIVMVLDADAGTLGFVVDNHFLGVAFKNLPRGVELFPAVSSVRGGACIRLRYLNGATHKSLSTILSIAVPTYKIEVVSEINQTGKTLSLRSAHLSGDSLQGQVGLCFYDSKDSSIHYMVDTPDNHELHLLARVIQEICPQVIITSAKQERCMTRFLIQLGSNPDYKPEVVIYPYLDFGLELGKTRLLSAHLPFLSASISERDKMAYLSSCISFDSPLMTLLRRMSLSHTKVTDWQSLYKTVCNAVCIRDTVCHLPQSIQLFRDISEGLGDDLQHIASLISRVVDFETSIVENRFTIKPNIDPAIDEKKRQMAGLSDFLTDVARRELAHLDASIPSCCVIYIPLIGFLLSVPRLPTMVEKEDFELEGLEFMFLSEDRLNYRSQRTKELDDLLGDLHCDIRDMETTVMTQLQNAILERSAVLYKVLDLAAELDCLMALSKASQEYGYTSPKLITRKAIAIDQGRHPLLEVCSPVFVANSFESLESRGRIKIITGPNSSGKSIYLKQVGLIVFMALIGSDVPAKGAEICLVDAIFTCMPGRESVSVGLSTFMIDLNQMSQTLNSSTGNSLVLIDEFGKGTNTVDGLSLLAATISHWLKKAPVDIPHILLATNFHSLLQLGHLPTSTMLSLLTLETAVDGDELVFLHQLKEGICQSSYAANVARLAGLPANVVQRGMEVSELYRTGRAIKRVDSVSSDEQANRYRTVVERFMSLDLEDDNLDVQRFMKEEVLPSAGQLQNT

>Ubiq4

ITLEVEPSDTIENVKAKIQDKEGIPPDQQRLIFAGKQLEDGRTLSDYNIQKESTLHLVLRLRGGMQ

>Ubiq5

ITLEVEPSDTIENVKAKIQDKEGIPPDQQRLIFAGKQLEDGRTLSDYNIQKESTLHLVLRLRGGL

>Ubiq2

TLTGKTITLEVEPSDTIENVKAKIQDKEGIPPDQQRLIFAGKQLEDGRTLSDYNIQKESTLHLVLRLRGGMQIFVKTLTGKTITLEVEPSDTIENVKAKIQDKEGIPPDQQRLIFAGKQLEDGRTLSDYNIQKESTLHLVLRLRGG

>Ubiq11

EPSDTIENVKAKIQDKEGIPPDQQRLIFAGKQLEDGRTLSDYNIQKESTLHLVLRLRGGMQIFVKTLTGKTITLEVEPSDTIENVKAKIQDKEGIPPDQQRLIFAGKQLEDGRTLSDYNIQKESTLHLVLRLRGGMQIFVKTLTGKTITLEVEPSDTIENVKAKIQDKEGIPPDQQRLIFAGKQLEDGRTLSDYNIQKESTLHLVLRLRGGMQIFVKTLTGKTITLEVEPSDTIENVKAKIQDKEGIPPDQQRLIFAGKQ

>Ubiq22

MQIFVKTLTGKTITLEVEPSDTIENVKAKIQDKEGIPPDQQRLIFAGKQLEDGRTLSDYNIQKESTLHLVLRLRGGMQIFVKTLTGKTITLEVEPSDTIENVKAKIQDKEGIPPDQQRLIFAGKQLEDGRTLSDYNIQKESTLHLVLRLRGGMQIFVKTLTGKTITLEVEPSDTIENVKAKIQDKEGIPPDQQRLIFAGKQLEDGRTLSDYNIQKESTLHLVLRLRGGMQIFVKTLTGKTITLEVEPSDTIENVKAKIQDKEGIPPDQQRLIF

>Ubiq9

MQIFVKTLTGKTITLEVEPSDTIENVKAKIQDKEGIPPDQQRLIFAGKQLEDGRTLSDYNIQKESTLHLVLRLRGGMQIFVKTLTGKTITLEVEPSDTIENVKAKIQDKEGIPPDQQRLIFAGKQLEDGRTLSDYNIQKESTLHLVLRLRGG

>Chem1

YKCRCTRKGPKIRYKDVQKLELKPKHPYCQEKMIFVTMENVARFKGQEYCLHPKLQSTKNLVKWFHIWKDKHR

>Histone5

MAFLGAQIPRQTQRRCSLGPEENADDIVSLYCCPQAGGKAGKDSGKAKAKAVSRSQRAGLQFPVGRIHRHLKTRTTSHGRVGATAAVYSAAILEYLTAEVLELAGNASKDLKVKRITPRHLQLAIRGDEELDSLIKATIAGGGVIPHIHKSLIGKKGQQKTV

>Defensin1

DNEAASFPWSCPSLKGICRKVCLPTELFFGPLGCGKGFLCCVSHFL

>Histone33

MVEKARASKRPSISKMIMEVMAESKQHKGMSVGALKKVLALKGVDVLKLNKRINQSLVRLEKSGALTQVKGAGASGSFKVTKEKPAPKTKSPKKTSSVGLKSIQKVDKPKKPTSSAKIRSSLISVQKYVKPKQKPAAKKSETSVKVSKKGSKKAAAKATKPGKKRAKRRRESYSIYVYKVLKQVHPDTGISSKAMSIMNSFVTDIFERIAGEASRLALYNKRSTITSREIQTAVRLLLPGELAKHAVSEGTKAVTKYTSSK

>Histone32

MPEPAVKASKKGSKKAVNKSTKPGKKRVKRRRESYSIYVYKVLKQVHPDTGISSKAMSIMNSFVTDIFERIAGEASRLALYNKRSTITSREIQTAVRLLLPGELAKHAVSEGTKAVTKYTSSK

>Histone45

MARTKQTARKSTGGKAPRKQLATKAARKSAPATGGVKKPHRYRPGTVALREIRRYQKSTELLIRKLPFQRLVREIAQDFKTDLRFQSSAVMALQESSEAYLVGLFEDTNLCAIHAKRVTIMPKDIQLARRIRGERA

>Histone21

MSGRGKGGKGLGKGGAKRHRKVLRDNIQGITKPAIRRLARRGGVKRISGLIYEETRGVLKVFLENVIRDAVTYTEHAKRKTVTAMDVVYALKRQGRTLRQLVFSKTNRRRAMGEEAPAPPATTPSKAAGKAKKKKLSTKRANRDAPTLSKQIVAVIANSKERKGTSVSAMKRVLASQGVDVPKVNKRINTSLVALVAKGVLSQTTGIGASGSFKLAKKEPVDKKPRKTKDVKKKPAPKKPTTTTGSTAAVTGRKSTMTAKKRRVSGKSDKKPKKLAKKPAAKKIPKLATTPRKTTTSRAKLIPKSKKETQKKGRKSAVKKSVPKKGKKAPAKKTKK

>Histone2

MSSRGGKKKLTKTSRSTKAGVIFPVGRMLRYIKRGLPKYRIGVGAPVYLAAVLEYLTAEILELAGNAARDNKKGRVTPRHILLAIANDEELNQLLKGVTIAAGGVLPNIHPELLAKKRGAKGKLETPLSPAPEKKAKPAKKTAGKKMGGKKGAGKAKKQGEASKTASADSTTEGSPADSFTVLSTKSLFLGQKLNLIHSEVSNLAGFDVEGVINPTNAELELKDDLGSALEKKGGKELAEAVQEMKKKNGPLDVAGAVLTAGFGLPAKYVIHCNSPAWGSDKCEDMLEKTVKNCLALADEKKLKSVAFPSIGSGRNGFPKQTAAQVILKAISSYFVATMSSTIKTVYFVLFDSESIGIYVQEMAKLEAS

>Throm32

MPAPLRAPTGSLKVLRVLLAVLVGLPERGVGLELIPLASQVLLQPHSNFTVVCSGWSEVSWRLPRQEFEEGNAAVEKQGSRSVLRLFNVTWRNSGRYACEESASDQIREMDIFVPGRGPDEWFLPVASGVVMKDGEEATIPCAVSDPLLNVSLYRRGDQTPTSGPTYEPGRGFTGRLDDASYVCVASGGSEETRSRLYYVFSVVVAKAMEADLTASGSVLKRGQPLLVNCTVRHVDMAYFSWKFPRSEEFESLTEFLPDRIRSFVNIPSATLADSGVYACAVQESLQGRRVEKNITVTILDRGYVSARATHDTNVSSLVGHTVTLRVAADAHPPPTVAWHKDNRSVVVTAGTAVVSSARVSDGRYVSTLTLARVRLEQTGSYTARIFNEDEREDVLFYLQVQAPPRITSLTETSTRAMLCVSEGAPPPSVTWFTCHSAHGCANGTGRWTSQSGASLPPENISVLANGGLTQVRSLLIPDSPKTLTAVRCEASNPAGRRARDLRLVSTSLLSRVAMLALVLVLGLIAIIFLVILIILWRKKPRYKVRWKLIESVSPDGLECVYLDPDRLPYSAAWEIPREDIELGEDLASGAFGRVVEARVSGLLQDRKTTRGAVKMVKSNSGARRSLLSELKLLCHLGPHVNIVNLLGACTSGGPVYVVTEFCCHGDLAGYLRRHKRAFVHADALAKREADGGYMDMSKREGAAIVGDTDNAASAQDQQGAPSPLVSDSAALDRNDLHSFSFQVASAMDFLSSRKCVHGDLAARNVLVCEERLVKVGDFGLARDPLKDQDYVARGRASLPLKWMAPESISHGAYSSQSDVWSFGVLLWEIFSLGERPYSDLPATRRFYAALKRGHRLAPPPHADSNMWDARHQACLAPLARMTSYDRATCDMMQACWSELPASRPSFASLAASLDVMLTDDRRKVTAPSACRSRCRRLSRRSTTSACLRPAQAYAGLTERFLTGDHPAVVRSRLASAAAEAAGDGETPSRDVRRAPENVRLHRPEAGPSHGEFGLAVDAGGREGERGAALDADRSKSERLRIGWKAPRPQKGRDASGALFVSVAAEGYVKREVLRGLGELGRGSEEAGPAPIRKLKSRSLECLSVSQKKKMKTWSFPLLLLSVVSAQDVSQPSIRLNSRPVRNQSEAALTAGSAFGLSCHGDASLRWSSTALLSPPLARGGELKVGRARPRHTGTYRCAYAERGLQHLHAAVHVYVRDPASPSDVFVTPHFTPDVKEGDDFLFRCLLTDPSFTNLTLRVEEEERAGGRDRDRXPSDPASPSDVFVTPHFTPDVKEGDDFLFRCLLTDPSFTNLTLRVEEEERAGGRDRDRDLPPGMKVSTDPRAGVLIHKVDRRFNGRYVCAAWNSNKTRVTSNTLHLIVTPRQRHPPEVSLNQSEFFRVVGETFAVTCVGSNPSLLYNVSWTDPEVEAASMRLSHRYNGNRLYINNTLTLAAVTLQQSGRYTCTASNEAGNATATARLRVLGAPFLTAFLERRTIAPSGANVSAKGNAGDEPVTSEREVLANLSLDATAARRLNGSRTVDVLEGGDVTLTVTVEAYPPLTGLEWSSPTPANRRAAHALSYAADGYRSRASLLLRRVRDEEKGRYALRYVNALVSGSFNFDLRVLRWKAPRPQKGRDASGALFVSVAAEGYVKREVLRGLGELGRGSEEAGPAPIRKLKSRSLECLSVSQKKKMKTWSFPLLLLSVVSAQDVSQPSIRLNSRPVRNQSEAALTAGSAFGLSCHGDASLRWSSTALLSPPLARGGELKVGHARPRHTGTYRCAYAERGLQHLHAAVHVYVRDPASPSDVFVTPHFTPDVKEGDDFLFRCLLTDPSFTNLTLRVEEEERAGGRDRDRDLPPGMKVSTDPRAGVLIHKVDRRFNGRYVCAAWNSNKTRVTSNTLHLIVTPRQRHPPEVSLNQSEFFRVVGETFAVTCVGSNPSPPLQRLLDRPRGRGETGPSMRFRRLGRFDARSPAGPQAASMRLSHRYNGNRLYINNTLTLAAVTLQQSGRYTCTASNEAGNATATARLRVLGAPFLTAFLERRTIAPSGANVSAKGNAGDEPVTSEREVLANLSLDATAARRLNGSRTVDVLEGGDVTLTVTVEAYPPLTGLEWSSPTPANRRAAHALSYAADGYRSRASLLLRRVRDEEKGRYALRYVNALVSGSFNFDLRVLRAPRGVITMENDTLTCSGSGFPPPTLLWSQCSGFVETCGDVAVDGPEDVAAREGEEPKKQLALPRALDDNITIECVSTNSVGRSRDVFFLLSTGSGGRSVLATPLAVSLGAAALFLLLLIVILVWRIRRVRRSSSFALAPPPIIIIIIIISFLLIASQQPKYEIRWKIVESCSGNNYTFVDPSLLPYNNLKWEFPRDKLRLGNPAAGCVGHARPRHTGTYRCAYAERGLQHLHAAVHVYVRDPASPSDVFVTPHFTPDVKEGDDFLFRCLLTDPSFTNLTLRVEEEERAGGRDRDRDLPPGMKVSTDPRAGVLIHKVDRKFNGRYVCAAWNSDKTRVTSNTLHLIVTPRQRHPPEVSLNQSEFFRVVGETFAVTCVGSNPSLLYNVSWTDPEVEAASMRLSHRYNGNRLYINNTLTLAAVTLQQSGRYTCTASNEAGNATATARLRVLGAPFLTAFLERRTIAPSGANVSAKGNAGDEPVTSEREVLANLSLDATAARRLNGSRTVDVLEGGDVTLTVTVEAYPPLTGLEWSSPTPANRRAAHALSYAADGYRSRASLLLRRVRDEEKGRYALRYVNALVSGSFNFDLRVLRAPRGVITMENDTLTCSGSGFPPPTLLWSQCSGFVETCGDVAVDGPEDVAAREGEEPKKQLALPRALDDNITIECVSTNSVGRSRDVFFLLSTGSGGRSVLATPLAVSLGAAALFLLLLIVILVWRIRRVRRSSSFALAPPPIIIIIIIISFLLIASQQPKYEIRWKIVESCSGNNYTFVDPSLLPYNNLKWEFPRDKLRLGGVLGSGAFGKVVEATAYGLGSQDETRVAVKMLKPSAHSEEREALMCELKILSHLGYHDNIVNLLGACTRGGPMLMITEYCFHGDLLNFLRVHARHFAADLMSLEPPERDAKYSNRFCPLVAIISVSTGSGGRSVLATPLAVSLGAAALFLLLLIVILVWRIRRVRRSSSFALAPPPIIIIIIIISFLLIASQQPKYEIRWKIVESCSGNNYTFVDPSLLPYNNLKWEFPRDKLRLGGVLGSGAFGKVVEATAYGLGSQDETRVAVKMLKPSAHSEEREALMCELKILSHLGYHDNIVNLLGACTRGGPMLMITEYCFHGDLLNFLRVHARHFAADLMSLEPPERDAKYSNVRLRRFAGLFYFLHFCIHRDVAARNVLLTDRHVAKICDFGLARDIRNDDSYIVQGNARLPVKWMSPESIFQCVYTVQSDVWSYGVLLWEIFSLGKSPYPNIAVDTKFYKMIKDGCHMDRPDFAPPQIYRLMTSCWSLEPTERPTFKMIGRLMERLLPCANKAGQSEQPTYKNIDERGEDEETEEADGGDPLKGEEQDFRTRAVGCVDAVTPRGFTSPSLTGRCVCAFVCSSSLCSAAGPSSGCGRRDSESAGDSPHKSQCDASRSCFLQSNHPSSDGCVRLRAGSRMRRRGACSSFNFLLAALCVLLLLCCAALTALMWAEAPPPDAEGAGLRGEMAIARGAEFSQDLRNGSSAAFKALAFDVQRLVREAFRRGGLSRRFVTCDVVAFTRGSVLVTFDLWFTGSVERQEAEQQMKAGLEESGGLVVDKDSIRITGELAADVSLKSTGLMSWRLSTEKEQQEEATATTTAACTPSATPVTVTCPPYHASCDRSSTCVHAERLCDGIDDCADASDEDAARCATTCDGQFELRGPSGWFASSAKGGFCRWIIRVQSGFSVQVNFHEFDSRGSDIVRLYEGVGADKTMAAELSGSTPPGTVWLLTDQSTIEFSSDNFEDASRFNATFSASNLSALTDSEKLSCTFERGLCFWRQDLEDDGDWLRSRGATFPPLSGPSADHTLGNLSGDVGTVVFHRDGNYGDNWNYGQVLLSSPTDSQVVFEALKRGGMRNDIALDDITLTSEPCGPAPPQPTLVPTPTAAPTIAADCGGPFDVWEPNSTFGSPNYPHSYGNRARCVWTLRATPGRNIQVHFLDLDVEENYDIVEVRDGVGPNSTSLAILTGSGGPAHDLFSTSNRVTVWFFTDSSGSGRGFKANFTSGVGLGSPEPCPADQFRCGTGDCIRANRQCDARVDCGDASDEANCVVLQANTSSRLRFQIASSWFSVCADTWTPRLSRFTCQYLGHRSGVVTLLPAVPGDSPFASVKVTSNGSLETSVSDGCPGHRVVSLRCNNQPCGLRLVANDTVDQSAARDGKVKVVGGSDAAKGAWPWTASLHWNGRHVCGAALLGRRWLLTAAHCVYGKDRPLSRWSAKLGLRAQSHAGDARSFQIDAVLVNPHYNRLTKQADVAMMRLSAPANFTDLIQPVCLPEHGEEFTAGRKCWIAGWGRQAEDGSLPDILQEAKVPLVDRVSCQRALAEYSITSSMLCAGFPEGGVDSCQGDSGGPLMCEEEEGRWTLAGVTSFGIGCGRPGRPGVYARVSAFVAWIAETRRSSSLP

>Throm20

MASVRLGLLLLLLTFGVQAAADDFEGNPRGVRLPRRAASQVLRRQRRFNAGPFEEMWAGNLERECREETCDFEEAREIFEDETKTMQFWAGYVDGDQCKPPPCQNGADCEDGVNSYVCWCNSSFTGKNCEIEVSKQCSVNNGGCSHFCTMKSNAVRCSCALGYKLADDLASCEAAGEFSCGGTGAASAPAPRSSNGTDGDVTDVIPDEWLDYEYEEELERGYNISAGSRLRRRSARRASEPPPLPSYRAFFPALPTVTVKENSDLRIVGGYEAKAGDFPWQAALISHSARLTESTAEPFCGGSLLSELWVITAAHCVVEARMTGLTFFVRLGEHDVGRPEGPERDHEVAEELVHKSYKFHKSRFNHDVALLKLATAAELSARRRPVCLGPAAFTERLLREAPISEVSGWGRLRDGGLRSATLRRLQVPFVERGVCKASSRTPVTPFMFCAGFRDRKEDACQGDSGGPHVTRRRDVWFLTGIISWGEGCAAAGKYGVYTRVSRYYAWISNTTGVRTYR

>Ubiq20

MTEGQLRSKRDEFWDTAPAFEGRTEIWDALRVAAGAFESQDHQLAQAILDGASITLPHGALSECYDELGNRYRLPVYCLAPPVNMIEERRDEPDASDRDSGGADSDGGGERPLRLRLSTGRDLRLRVRASDTVGAMKRRLQAQEGVPAAAQRWFFSGRPLTDRLRLEQINISRDYVVQVVVGQPPPDGAPARETRRRPAPAEA

>Throm47

MPFPLVEAEEVTLILMKMKPGLLVHQIHKYLSRTYTSRPSLNEVVIVSSVRTPMGSFKGSLAAVPATKLGSVAIKGAIDKAGIKPEDVNEVYMGNVLQAGEGQAPTRQALLGAGLTLGTPATTINKVCASGMKSIMMAAQSLMCGHQEVMVAGGMESMSNVPYVMPRETPSYGGVRMEDLIVKDGLTDVYNKFHMGNCAENTAKKSSITREEQDSYAIGSYTRSKVAYESGVLAKEIVPVSIPQKGKPDVVVSEDEEWRRVDFSKVPKLKAVFQKENGTVTAANASTLNDGAAALVLMTADAAKRHNVTPLARIVSFADAAVAPIDFPIAPAYAVPKVLDAAGLTKNDIAMWEINEAFSVVVLANIKMLDIDPAKVNVNGGAVSLGHPIGMSGARIVGHMVHNLKSGQYGLADCGESSAFPSSRIVGGTEASNGVWPWQVSLQISQRGHICGGSIISSYWIVSAAHCFQRFSRPSVWTVYAGDISLLEMRNYHGKKVEKIISHEKYDPETYDNDIALLKLDTPLTFSERVKPVCLPNFGMEFLAGDAAWITGWGSLSSSGPSPDKLNQALVTIYSREACNNREILNGAVTESMICAGKLSGGVDSCQGDSGGPLVVKEADVWWLAGDTSWGIECALKNKPGVYGNVPYFLDWMYWKMQVQ

>BPTI13

MNMKREPLGCALWFLCVGLVVSCDWRPLDNDLGAPSFLEDGPTVLERLDISDPAECREECCDDLRCQMALLGFPADGPAECLLVRCLIGGHNVCELSPNSQYQLVGWKKPLKAVPLVEASQPSKNDSTNPCLQPMKVGSCRAAFPKFYYDVVNQTCRNFTYGGCDANGNNFESREDCEATCNGVTGSVLPEASTRPRSSVKSLRMAQPIDDQSELWDDALDKVLSAEDYSERCEAEPMAGPCRAAFRAWYYDSQEHKCKTFIYGGCQGNKNNYDSQQTCLDTCYVSEDDCLSSPDPGPCRAAFPKFYYDPATGSCQSFIYGGCGGNKNQYSSAEECQTRCNGATDGYFAGRDKTRSRWTAAFFVLLTLAAICTVLVSTLVVTMLRRLTLPRRASVVSDKEELLPEVRSSLESLNIPALPISGKA

>Histone43

MRHDSSSSRRKASHPRRRPPPSPAATPRGHRPPSSPQPGPSGAGRSPRRRRYRPGTKALMEIRKYQKSTELLFRKAPFSRLVREVCQAYDRGALRWQVFALMALQEAAEAFLVMLFSDANLCAIHAKRVTLFPRDLQLARRIRGTQCT

>Neuropep1

MSIFPTSAQHPSAVDVNTYLKITPKAADICAESVETKSERVARGRPVGGDVPYAAMGGLTDFSSLVDGYIALEASRLPMTASAVTLAWRKAAAACSSDARLVRAYKAQCAPGALQISEERRMSIAHRHLKSTVENMHSNFVSLVGTLAFVLWALLCLGTLTNGYPVKPENPGEDAPAEDLAKYYSALRHYINLITRQRYGKRSNPEILDTLVSELLLKESADTLPQSRSVLCSRKASFRFSCRHFTAFVCSSLSSGLTHRAPPLLLYRCG

>Defensin3

MLRLSVWLALGAFVSCKIPCPDETVCSDHTTCCQTEQGYSCCGYPNAVCCPDLSHCCPQGFRCDLVSQQCEKVTTSEDPHLCVGLEAAPSFCRMPPADTITQRNSLLSPLLLFTVAQSEEERRRAAEL

>CDK-like4

MDKETIGTQYEPVAEIGEGAYGKVYKARDLKNGGRFVALKRVRVQTEEEGMPLSTIREVAVLRQLEAFEHPNVVRLFDVCTVSRTNRETKLTLVFEHVDQDLTTYLEKAPDPGVPPETIKDMMYQLLQGLDFLHSHRVVHRDLKPQNILVTSGGQIKLADFGLARIYSFQMALTSVVVTLWYRAPEVLLQSSYATPVDLWSVGCIFAEMFRRRPLFRGNSDVDQLGKIFDVVGVPSAEDWPQEVALPQSAFIPQPAKPIEDLVPDIDEQGRALLLQFLAFNPSRRISAFAALTHPYFQSVDSLSRSVYAAQPIPGNKPAIEERTA

>BPTI11

MERYYYNTITQKCELFYYGGCQGNANNFKTYHECHKTCFRIPKIPQTCRFPMEEGPCRALFSSYFFNMTSMQCELFYYGGCQGNDNRFPDLTSCKEYCSPRKSVPVLCLDPLDKGKCSASIPRYYYNKTTKTCEEFTYSGCGGSSNNFVSRHSCMDVCVKGGKKGSGGKMRRLRRNRITFLQA

>CDK-like18

MPNSDLLRDGRGRSPPAQRNANRQDRRNASKPGSSAAQRDRHRDKRRSSASRRKKRRQAKDRDLWHIGATEGTVSDRHGTFGKEAELRTLVEYDDVSSQSERFSGSPSPKLDHQVDRFPMDRLSDFEEYNAPTRDRQAVFLNKDVHAKGPAERSKDPGRKKERPNRERNAKVRGGLSPSSSKNQRDVVKTNDKNGKRASSSQSSQSADKRDSARQRNKTRADKEPPSAYRDVPPQSYRDEELRAYRRSPGFKAESPYGTSYPYSYQSPSGYNQMTSRRSPPYPSKRTSPAPNYYGRDADVYGAYGVAKSPSSYSGSKRKRSPTSPANWRRSPSYSRHSPYEQGEFVASPYAGRRRSRSPYRKSLSPSPDVRRSVRSRSRSPYSSSRHSRSRSRHRHSRSRSRPSSLSPSTLTFKSSLAAELSKQKKAKAAEAAKAKGAGKVSTPTKNAAARQPSPKTNHAARKGRLPSPPAPPATAAAEKGPRTPTSSQPQSPSDCSSKKSSEQPVSREKDGKAKDDSKRKAAAAASGPSKDKERASGTFISGVPSLPLPLSVLEQIDKGDSAKDTSAKKKSDRKGRQLLTDLPLPPLLSSRTSSPHSPADEKKGQTPRKRPKMCGPRYGEIKETEIDWGKRCVDKFEIIGITGEGTYGQVYKAKDKDTAEMVALKKVRLDNEKEGFPITAIREIKILRQLNHKSIINMKEIVTDKEDALDFKNDKGAFYLVFEYMDHDLMGLLESGLVHFNESHIKSFMRQLLEGLDYCHKKNFLHRDIKCSNILLNNKGKIKLADFGLARLYNSEESRPYTNKVITLWYRPPELLLGEERYTPAIDVWSCGCILGELFTKKPIFQANQELAQLELISRICGSPCPAVWPDVIKLPFFNTMKPKKQYRRRLREEFAFIPPTALDLFDHMLNLDPSKRCTAEQALGCEFLKDVDPDKMPPPDLPLWQDCHELWSKKRRRQKQMPEELAGPPKAPRKELGLDDSRSNTPQGFPASAGIKAPSAAASSLLDPKGPNAQLTQEQLAVLLNLLGPPNSTAANTAQFFQAVNSKVNQETLQQLSKALPAPDAVEPPAQPPPPAKPPQPAVPAGAPRTPPMPKPPSPPPPEGDAAAATQTAVTMLLAQLLQAQRQEVGDGVGDAAEAPANNLGAGAPVLPLENKQAPESSPVSPALDMTGVPILPPDQRPPEPPEPPPHADLDYRRPPPEPKPGHAPPIASAGGGDGGARPPEPDYPPLPTAEGPGYGGDFSHPLPPPHFTPTGFGDSFMGHMLGAGLPPHSLREVFSGPGQAPPLEPFPPPPGPPSTGPPGMMFAGDKDHRFEYSPLPVEGQPNPGALHLYNHALVRKDGIPPLPPPPIPAPGHAWASPSSQVPLGFAPHINSTIRGRGLPF

>Chem2

MEGRTVYLLAALLILTTFTTSTHSASCCLRYMKRKLPCQKVSDYSLQSIGKSCDINAVIFHLPGRFLCANPIASWTQRVMRCIDDRKRKSEKALIQQSVNSTAAA

>Histone42

MGSFILRFEIKTVTYRSSVLSHWPGTVALREIRRYQKSTELLIRKLPFQRLVREIAQDFKTDLRFQSAAIGALQEASEAYLVGLFEDTNLCAIHAKRVTIMPKDIQLARRIRGERA

>Neuropep9

MEAMMKGLNKAKEGVVVAATKTKQGVSGAAEITKDGVLYVVAGKTVEGVSQVSGAVVSGMTNIAQKTVEGAGNIMATTGMSKKDPAKPSNDNATAQDAAESPVDTDTTDATEDDTD

>BPTI4

MRTAEVAVVMVMLCTSMAHSDVKPQRDFNLQRFAGRWYRVGLAYDSPSFVQYRDKLRVSLGVVTAQASGNVNLTMWDATPAGCRSKMYYYEKTNIPGQFTYFSTRHNMVKDITVVETNYNEYALVLKHKVFHREYTQVALYGRTPRVRVEVIQKFKAFALSRGFPREAILTPPPAEWGTEVDAFVVHTNYEEYALMISVREKSPGQSSISVKLYSRTMDVRDTVLDDFKRLVREQGMSDDTIIIKQNKGNCEPEEQVEEPATVFEVPRMRRDVAPTVALAEMELDGSGDNMLTFNGSEACSLGPEPGPCFGIHQHYFYNSSAMSCQMFRYGGCLGNKNNFKTERECLQRCRTEGNCEPEEQVEEPATVFEVPRMRRDVAPTVALAEMELDGSGDNMLTFNGSEACSLGPEPGPCFGIHQHYFYNSSAMSCQMFRYGGCLGNKNNFKTERECLQRCRTEAVCRLPLSPVACTGEPLIWSFSSTLGECVPYKVGFCQTDGNKFYSKAECEEYCGVKAPKILEEE

>Lectin6

CQAGWRENDHKCYYFSADTMTWWEANTFCLSQNSNLMSIQDIHERLWVRTQIGTEIFWIGLTDGITEGVWEWTDGSPFIQYISYWVQGQPDNWGDEPGEDCGQVVGGSSGRWNDEHCDVKRKYICKHANSNPGPQCDLSNGWDRYSSNCYKLKSGTAKSWSAARHDCIVEGGDLVSITSEREELYVTGMLDPFRIDLWIGLSNLDRHQAPIRPAYPNKYTPLSATSPNSNGEVGSCTAIIKDHSDEFGKWRSHLCRHERPYMCKRPVNTICPAGWQSFSGSCYWLVSNTDLLTTWYEAFATCSNEGAHLLVINSQDEQFFINGKLPDFHQVDVPDIWIGLSDKDQDGTFHWVDKTAVNYTNYGPGWPRNTANIWDCGMIFTASHCDPGYLLYGDFCYHFEGELVKTWQDAETYCVAQQGHLVSFHSQEELSFLIAHMPGKAWVGLNDINIESQFVYTDGTATNLLPWAPKQPDNWQNNEDCVHLEGPMHTEAGKLRDDFCTATNEFICKKGPKVIHSFFACILMWSLIFHTYGIPAAKGQGPPPQPPTSGPGWNEKCSSWTADPFNDFCYLFNSLSTRTWAEARADCVNQGGDLVSITDPFEQAFIQGVIQVSPTGISLWMGGHDSITEGGWEWTDGSPFRYVRWSAGNPDNYFGEDCLSIIINNGYWNDDNCENRRGYICKRRGFMTAIACQETSVVLHCPQESVINIQSAFYGRKSSDVCPHLGGSDGSCMVEGVLPYYRKVCDNRPFCFAYAYSEVEPCPSVSKYLEIVYSCEQKVCLHGLGMEDMNITDSQLSASSSMSSFTPNKARLNGNSCWKPIGNPTGSWIQANLGQKRKVTGIVIQGCPNSDHWVTKFKMQHSIDGQSWTDYTADGQTKSEKTFKNVFGSEWVKSMLLNSTLHLTDAITCASKANFDLGNDQMTVHCPAGCAKSSYAVYGTDIYRADSNICAAAIHAGVVLSEIGGDCTLLKAQGKNFYAGSTRNGITSTQYDGEYPLSYTFANGELRCPGPDWYEFGEFCYKSFEDKKTWADAQDTCRSIGAELVSIRSMVEQSWLESYLYFATSDMWTGLNDLLVSGMYVWSDHHMTGRWNDVSCTELNRFMCKMPKGHYQLPSVKPTVYGCPQGWEAYGYGCYWMEETGRSWSDAKAFCKEQDAFLLHIGDIYEQAHFTVVLSGKTGLWWIGLRAKGGTGGGVQYIWENGYPMTFTHWDRNQPDNGDGTCVAMTTGQVGGFWDDKQCSEKFAFVCEKPRPDITPPTEAPTPPPAQGCVDSWTSKPNFRNCYRLFHNVDWSLKKSWGAAHEDCVARGANLVSIHSQEEEAFLSLYSKGSSKWIGLRNNPTEGGYSWSDGSALSHTHWGPGEPNNHEGREECVEMVSSTNGTYSWWNDLNCDAHQDWICMIAKGQNPIPPPVPPSPVPAPDCGSNPGWRKNNNMCYYYNDTDIVDFRTAMHRCWVEKARLVSILNKDEQAYVNSMVGTGQISAAWIGLRMLGIADGQYAWQDNSPVSYTHWSPGEPNNANGEEQCVQMNRHQGGWNDVNCGRAAAGYVCKKFPGTDHTPPPPTQPWEGYCPAGWMHFNDKCFLFKGKKNDIKANWTYARSWCKDQGGELAVIDNQYENDFVSSYLKDMELPTWIGLSDILVENQYAWSDGVSPVLYTNWNGKEPNNAGGTEHCVSISHSVLVTGKWNDDACHKKHSFVCFRKKSSSIAPPHPTSSPCPAGYISWYLNCYKLVEEPATWDAAQKACEQQGANLASIDMSYDQAFVAGVVLQGKADAWIGLKLKDDGSFAWTDGWPVFFTQWGPGEPDNLNDEGCVSMRGSPLFHGTWNDTRCDQAKPYICKISSERPPVTPAPGDGKCLPLWIPYGRYCYWVYNEQKGFSWPDSRHYCQEAKTELASFHSRAELEFIRNLNHTKYHNFWIGLTRDRNFGWGWTDKTPLGFLNWAPGEPNAAFHPGQVAEENCVEMYPDGRWNDNDCLQKRGFVCRHRQYYTTDDGGNPVFPTDGPNVDNSGVNFGVVAAVVVVIVSLIAGLLFYVFSVRGYKLSSLRQSFPKRATTNVDIPAFSNPNFVDD

>Histone11

MTGRGKTGGKARAKAKTRSSRAGLQFPVGRVHRLLRRGNYAQRVGAGAPVYLAAVLEYLTAEILELAGNAARDNKKTRIIPRHLQLAVRNDEELNKLLGGVTIAQGGVLPNIQAVLLPKKTEKTAKAKAGFITVDWNAPCYSTNIFNQITMSGRGKGGKGLGKGGAKRHRKVLRDNIQGITKPAIRRLARRGGVKRISGLIYEETRGVLKVFLENVIRDAVTYTEHAKRKTVTAMDVVYALKRQGRTLYGFGG

>Histone40

MARTKQTARKSTGGKAPRKQLATKAARKSAPATGGVKKPHRYRPGTVALREIRRYQKSTELLIRKLPFQRLVREIAQDFKTDLRFQSSAVMALQEASEAYLVGLFEDTNLCAIHAKRVTIMPKDIQLARRIRGERA

>Histone25

MSGRGKGGKGLGKGGAKRHRKVLRDNIQGITKPAIRRLARRGGVKRISGLIYEETRGVLKVFLENVIRDAVTYTEHAKRKTVTAMDVVYALKRQGRTLYGFGG

>Histone7

MSGRGKTGGKARAKAKTRSSRAGLQFPVGRVHRLLRKGNYGERIGAGAPVYLAAVLEYLTAEILELAGNAARDNKKTRIIPRHLQLAVRNDEELNKLLGGVTIAQGGVLPNIQAVLLPKKTEKPAKSK

>Histone35

MPEVAKSAPKKGSKKAVNKAAGKPGRRRKKGRKESYAIYVYKVLKQVHPDTGISSKAMSIMNSFVNDIFERIASEASRLAHYNKRSTISSREIQTAVRLLLPGELAKHAVSEGTKAVTKYTSSKIIPRHLQLAVRNDEELNKLLGGVTIAQGGVLPNIQAVLLPKKTEKAAKSK

>Histone31

MPEPAKTAPKKGSKKAVAKSVSKSGKKKRKTRKESYAIYVYKVLKQVHPDTGISSKAMSIMNSFVNDIFERIASEASRLAHYNKRSTITSREIQTAVRLLLPAQYEEELNGTKEKNERLDAVFKKARVGFQRAEQEEEEAPHIIEAEEVPHQIKEEVEGEDVASMSVPVKSEDEGQTADNSEPESPSSTSNRHITEEISETLYPGQNESSSPHIKEQIEDKEHPVIKEGDEPEPLHMKEELQPEPFYDKEKEETEHHRIEEAGKPVHIKEEEHPATLHNMEEGTPRPLYIKEQEEEEEEPPHIKEEEVEEDMTKMPLTGGPLKSEADGQCEENRRVEPPSSSSDQYMNTEGDGDHRGRPLEHSLLAPLSDTDDTLFHSPHTDDERAEDVNEDVYPSSSDLRSEFPQIKEEVKDKEPSQIKEEGEEFLYIKEEEQENIIKVLLTDVPLKLMETTVEDHKQVDS

>Histone20

MSDNQSWNSSGSEEDSEPREETGNLVGVGVFSGVLSKWTNYIHGWQDRWVVLKNNTLSYYKSQDETEYGCRGSLCLSKAVVTPHEFDECRLDISVNDSVWYLRAQDPEHRRLWIDSIELHRADPGYGSESSLRRHGSMLSLTSATSGYSATSTSSFKKGHSLREKLAEMETFRDILCRQVDTLQRYFDGCADGVSKDELQRDKIVEDDEDDFPSTRTDGEFLHNSSNSAKDKLFHCSNPKAVNGIDFKGEAITFKATTAGILATLSHCIDLMVKREDSWQKRLDKEMEKRRRIEESYKSALNDLKKKSHFGGPDYEARPSKKSSIFISVCFSHECSRVDTSSCHRQEGPNSLINEEEFFDAVEAALDRQDKIEQCQTEKTRIQRSSPLPPEDFFSSSGSHRLSEQVEEMVQSHMTYSLQDVGGDANWQLVVEEGEMKVYRREVEENGIVLDPLKATHSVKGVTGHEVCHYFWDTAYRNDWETTIENFNIVETLSENAVIVYQTHKRVWPASQRDVLYLSAMRKMVASNESDPDTWLVCNFSVDHHDAQPSSRCVRAKINIAMICQTLVSPPEGNKEISRDNVLCKITYVANVNPGGWAPASVLRAVAKREYPKFLKRFTSYVRGLVIRVPALSSSARYTSYSTLVENMSGRGKGGKGLGKGGAKRHRKVLRDNIQGITKPAIRRLARRGGVKRISGLIYEETRGVLKVFLENVIRDAVTYTEHAKRKTVTAMDVVYALKRQGRTLYGFGG

>Histone34

MPEPQKPAAKKGSKKAVSKATNKAGRKKKKTRKQSYAIYVYKVLKQVHPDTGISSKAMSIMNSFVNDIFERIASEASRLAHYNKRSTITSREIQTAVRLLLPGELAKHAVSEGTKAVTKYTSSK

>Histone46

MARTKQTARKSTGGKAPRKQLATKAARKSAPATGGVKKPHRYRPGTVALREIRRYQKSTELLIRKLPFQRLVREIAQDFKTDLRFQSSAVMALQESSEAYLVGLFEDTNLCAIHAKRVTIMPKDIQLARRIRGERA

>Histone12

MSGRGKSAKSRAKSKTRSSRAGLQFPVGRVHRLLRKGNYARRIGGGAPVYLAAVLEYLTAEILELAGNAARDNKKTRIIPRHLQLAVRNDEELNKLLGGVTIAQGGVLPNIQAVLLPKKTEKAKAK

>Histone17

MYRYTSHLNLVENMSGRGKGGKGLGKGGAKRHRKVLRDNIQGITKPAIRRLARRGGVKRISGLIYEETRGVLKVFLENVIRDAVTYTEHAKRKTVTAMDVVYALKRQGRTLYGFGG

>Histone13

MARTKQTARKSTGGKAPRKQLATKAARKSAPATGGVKKPHRYRPGTVALREIRRYQKSTELLIRKLPFQRLVREIAQDFKTDLRFQSSAVMALQESSEAYLVGLFEDTNLLLRKGNYAQRIGAGAPVYLAAVLEYLTAEILELAGNAARDNKKTRIIPRHLQLAVRNDEELNKLLGGVTIAQGGVLPNIQAVLLPKKTEKVKAK

>Ubiq14

MWIQVRTIDGKETRTVDDLSRLTKIETLRVKIRDIFNVSPQQQRLFYRGKQMEDGQTLFDYNVGLNDIVQLLIRSQTDPPDSPVTKDPHSVACTSAPAAASAVPRAESPDRPAPVSPAAMETSTDKDNDCSAAAETANEPKPDQAVALNNSAGTKNGFKPSSPTRDTQLPTSSKNTLVDPGIGKYKINELVDCRDVSVGAWFEACVENVSRAPKGPVKNKVGRPPKRTNGKLESEQGQQPLGPGQSTDNVRNNVNALNSESNGASTSQTDSTDEDVVYHIKYEDYPENGLVEMRAVDVRPRARTLLRWDQLMVGMHVFVNYNMETPEERGFWFDAEIQTLNQASRTNKELRVKILLGGPGDVIGDCKVHFLDEIYQVEKPGAPALSAADGQFKRKSGPECKHCKADPDAECRFCSCCVCGGKQDAHMQLLCDECNMAFHIYCLNPPLATIPDDEDWYCPTCKNDTNEVVKAGEKLKASKKRAKMPSATTESQRDWGKGMACVGRTKECTIVPSNHYGPIPGVPVGTTWKFRVQVSEAGVHRPHVGGIHGRSNDGSYSLVLAGGFEDEVDRGDEFTYTGSGGRDLSGNKRIGEHSFDQTLTHMNRALALNCDAPLNDKDGAESRNWRAGKPVRVVRSSKGRRISKYAPEEGNRYDGIYKVVKYWPEIGKCGYLVWRYLLRRDDQEPAPWTPEGLDRIKKLGLLIQYPPGYQAVMANKTKKEAIARPGRGGRSKHHSGRGRGRGRPRKQLKIEEEEDEEYEHLMAAVEEEEEERPQSNGDQKTSKDSDSSPASEPPSKRPKVEEAFVLSQQQRQLIGEDAANKKLWDEALGHLAEGPNFLRKMEQIFMCVCCQELAFQPITTVCSHNVCKTCLQRSFRAEVYTCPACRHDLGKDYVMTQNKTLQLLLDQFFPGYTKGR

>Lectin16

MVLCASVLTILLPLLCASSARPQAAASHAGMDKMEDTWRVEGSLADTALLPCKLPMAPSAPADDEQLRVKWTRVEAGTEKVVLVAQGGAVKLGQDFMGRVSLPSDAPEMGEAALAVTGLRASDAGRYKCEVTRGMEDRRSSAILSVRGVVFHYRANTSRYSLDFPAAAEACLSVDAAIATPEQLTAAFHDGLDHCDAGWLADRSVRYPITQPRPGCEGDLKSRPGVRTYGVRLATEKYDVFCYVEKLDGEVFYPPSISGKLTLQEAAAECRKHDAVLASPGQLFAAWAAGLNRCDFGWLSDGSVRYPITAPWPQCGGGQLGVRTLYKYENQTGYPDPEDRHGAYCFKAKLPEATLTSPTSTEVVPEPSATNAQHTSPASELQTNHVTPPASHDDDVITEKHLTQLESLFFTTASALHTTHGISGGGESGSSDEDSPSGADPEVEGVTPAPLFPDWSVTQKTALVFKEEASPGSFGVDQSVAVPAGEGGSTAKPPLHVIIVNVHDKNQSVDRILQLLNSPAGDAVVFPQITDLSQSIGELVHGSGDADAAEAPLQAISFINGKHKVTLDARPPEEARGDQFETAAPIRVSEEEASVMPFDYDAFGEEPAGGPVTDGRHRDFLSYFSTPASPTRTPSPSASSRDTTREGSAVDGLAGTLDASPVSTNGMELGGSEGPSFAPETETQDANVPTKDDEGSTSGQDHGETEEEPAALAPTPPAPGGFSGEQLSGNGEAQPDPQPANLRQEESTSSFTAQQKLSTVTPPSHTVSNHAVPQWALSLDSAAIPTLPEDFLDYDSKMTPQLVESRLPWPEETMDQPDLLEATFINVTDVQPCSVNVCQNGGSCYQRGSERVCACAPGYTGGRCQTDVDECQSNPCLNGATCLDAVNGFSCLCLPSYTGKLCEQDTASCGSGWHKFHSHCYKHFPQRRTWDAAERECRLHGAHLASILSLEEQLFVNRLVDDYQWIGLNDKMFEGDFRWTDHNPMQYEHWRPNQPDSFFERGEDCVVLIWHAGGQWNDVPCNYHLGFTCKKGTVSCGPPPAVRDARVFGAPRSRYEVNALTRYHCKPGFIQKRTPTIRCRPDGRWDVPKVICISPATYRLQPSAVTHRHVHHATSHHGDRRQNYDGQRSFLNPLLDLER

>Histone26

MSGRGKGGKGLGKGGAKRHRKVLRDNIQGITKPAIRRLARRGGVKRISGLIYEETRGVLKVFLENVIRDAVTYTEHAKRKTVTAMDVVYALKRQGRTLYGFGG

>Histone18

MSGRGKGGKGLGKGGAKRHRKVLRDNIQGITKPAIRRLARRGGVKRISGLIYEETRGVLKVFLENVIRDAVTYTEHAKRKTVTAMDVVYALKRQGRTLYGFGG

>Lectin10

MDHTAFAVPTLFFLFLSLICFVAGETDAVSCPEYQESFDGSCYEFVGLQRPFLGAQKWCERGGGHVAFILNSETQKFLQTHLDPKKDWWLGLAPAAPNLTLDTAATQVSLAWLNGSDVSYNNWASKPDEQATCGHILKKSGFRWVASENCTQELSFICQFESRRSFACDGQNVTLQCDSGQVIEIDHSFYGRKTIHYCRTKLAPTPSPPQEECTWTDVADSVKGSCHGLQACQAPADAKSYGEPCPLLGSYLSVEYRCKDGLHLLMSKLAAVFDNVTLNIKWLLQPYEGNLTCALNAGDGYTIDSITPEEMETTVMHTFKTPGLFVVTVECSTSDWHVTAQKSINIQEPVGQFGAIKCYSINTSEGGTNCSLLYIGGPVDVQVTVETGTNVSYTLQHKDLLLANSSADRGIARHNITLNASAVEQLGHGCNNLTLNASNNVTSQIVSTQFELCLLEPVEGLQASVIAEDDRCPNSSDLIIGVSLERGAPVQLLFTLTVNNGTRLETRNMLNGSLQMYTFGSPVEVEQLGHGCNNLTLNASNNVTSQIVSTQFELCLLEPVEGLQASVIAEDDRCPNSSDLIIGVSLERGAPVQLLFTLTVNNGTRLETRNMLNGSLQMYTFGSPVEGSFQLNVQAANALSTSDFNVSLESIFLACENHSDSENMMTGKSYDLKIISTPESVVDYTENVTLSMNGSVSLPSKGLKFKWKCRGDCKCKNQTNDTTHMIDKQCLPNPFEMAVYTVTVKKTAWFRKWRDDLGTCYSQEGRRPLVRKQEGNSEFLVNSKELKYSRDNLLNITVIAQGKHLRCSNKEFVKSILLPVGDEKNNYNLSIVITVKNEYTEVTTMVHTQVRKNNSSGSVDTLQSVLEDTVSQLEQDGLLSSEALSQIVTSVADMLNAEADEDQKDARTKLREQMLTKMTTVLLDSPSNTSYEVQVTARAVAELTQRSDELSPAAQEEASSLLVDLSSSLNIISVNQDGSGDDGEVVDGAAPIVEAASNILNAPSNEKVSTSLLTGMGNVQNALLNHKKLNGDPIIIDSNQISVYVNRVSPESMQMQDINIQRSSASRFSFPTLPSDVFSPDEPVDVRMMSFEKNPYSWKGGNISGTVGSVSLTRPNGTVIPIKNLPEEIEIRLPRPDVGQENSTVLDLANFSTLIIDVPSPDVTLVLKMEPSENITFILLLGHKNYPTIEHFVAKTQMPLENVKEEEKYTWVLSPKDRTGDAGVHFLVLKPKVEPGVKSVNATVSIVTIAAQCKYWDEEESSWSEDGCRVGPLTTPLLTQCLCNHLTFFGSSFFVMPNLVDVSRTAELFSTFFNNPVVVCFVGAIFCAYLLVVVWARRKDIQDSAKVKITVLEDNDPLAEYCYMLTIGTGHRHGASTSSQVTITLLGTEGESEPHHLTDLEKPLFERGAEDVFLLTTHFSLGELQSVRLWHDNSGAHPAWYVNKVMVQDLESGQKWHFLCNSWLAVDVGECNLDKVFPVATEEDLKRFSNLFFMKTAKDFRDGHIWFSVISRPPCSTFTRVQRVSCCFSLLLCTMLTSIMFWGIPTDPSEQTMDLGHIEFTWQQVMIGIQSSIIMFPINLLIVSIFRNTRPREKSQKSDKTKQGKTVRVSPSQTSSPQKEQKDITPDTVLKDIKRIAQSLSKAMKSPLPYLELRPGHQTDINTLLSLVEDIIRQQNRISSDFYTDTAKRERSILSLGAVDLQEKSVWGSPERTSDEVQKKSNNSRYLYRQLRHVEKKLGFLGSSRFQNPDSYNKAMQQVQGMKNLLEFHLPSHSLDGEQSERSPSPEESANKGTAKRCCQGGLPWWFVFVGWILVIATSGVSGYFTMMYGLTYGKDRSISWLISMVVSFFESLFITQPLKVLGFAAFFALVLKKVDQEEYGEPQIDESLKNYDDPDGVRAARRDSTCSFYQPPPPTDIEKMRNNMIKEQKVFALIREILAYMGFMWMLLLVAYGQRDPNAYFLTQHIRQSFSKGISDTMSIQDVFNWANTTLLSNLFGEHPGFITDGNSKLVGNARLRQLRVQKNSCRVARRMQRAVHDCHSLYSWESEDMGSYSPGWSSSEPDNTSVNLPSPWKYQSQGKLRAYPIWGSMILYRGGGFVVDLGPDLENSSRSLQYLYENRWFDAHTQAIFVEFTVYNANVNLFCIVTLMLETTALGAFQFRSELQNVRLYQSTGGLHIFVMAAEAIYFLFILYYMFVQVKLMKQQRWAYFKSKLNLLELAIILLSWSALSVFIKRTLLGKRDMGYYQNHKDQFASFYETAKADAVLGYLIAFLVLLATVKLWHLLRLNPKLHMITATLQRAWTDISGFLVVMTIMFLAYSIASNLMYGWKLYSYRTLFDAAQTMVSLQLGIFNYDEILSYNPILGAFLIGSCIVFMTFVVLNLFISVILVAFSQEQIHHKPSEEEEIVDMMMMKLCSLFGIKSKTKQGDDGQTEKAQLTCASKNEIATISPGRIDKDSYVLL

>Neuropep5

MKLIFQAFLYCCLVATAAHCMELEVKPKLKKRLSIWLGSRLRRDLDSKTVNSEHFVRQDDIRDTMLPHSSTETHVRTKRSKNSANQSRRQGCYLGTCSVHDLAHRLHLLNNTLKIGTAPPVKISPQGYGRRRRSLPDRRVALRLEQGRLRPVWSTTDSQVHKLEALLRQT

>Throm22

MRCLVFVLLIGAAFATEDDKIVGGYECQPHSQPHQVSLNSGYHFCGGSLVNENWVVSAAHCYKSRVQVRLGEHNIRVTEGTEQFISSSRVIRHPRYSSYNIDNDIMLIKLSSPATLNQHVKTVALPSSCASAGTMCKVTGWGNTMSSTADKNKLQCLDIPILSDRDCKNSYPGMITNAMFCAGYLEGGKDSCQGDSGGPVVCNGELQGVVSWGYGCAEKDHPGVYAKVCIFNDWLESTMASY

>Throm36

MRCLVFVLLIGVAFATEDDKIVGGYECQPHSQPHQVSLNSGYHFCGGSLVNENWVVSAAHCYKSRVQVRLGEHNIRVTEGTEQFISSSRVIRHPRYSSYNIDNDIMLIKLSSPATLNQHVKTVALPSSCASAGTMCKVTGWGNTMSSTADKNKLQCLDIPILSDRDCKNSYPGMITNAMFCAGYLEGGKDSCQGDSGGPVVCNGELQGVVSWGYGCAEKDHPGVYAKDCDNSYPGMITESMFCAGYLEGGKDSCQGDSGGPVVCNGELQGVVSWGYGCAEKDHPGVYAKVCLFNDWLESTVSSY

>Throm2

MYQFVTVAHRLVRLLAAVCALGILGGLAVGVWILVKFLLRPSSSHSAVGLGDTKETSFCNVTEDIAIADPRKGSVFYRISPENSLLEIQLGRLPNWLPVCYERWNSSLGTLVCRQLGYLRLTKHKGVNLTDIGPNYTEGFLQINSEQRGTLENMWQFRRSCITDKVIALQCFECGTRAKLARIIGGVEATLGRWPWQVSLYYSNRHTCGGSIITSQWIVTAAHCVHNYRLPQVSSWAVHTGVVTRSLPITTEPTGHAVEKIVYNKNYNHVSHDSDIALMKLRTPLNFSDTVRPVCLPQYDYDPPGGTQCWISGWGYTQPDGVHSSDTLKEAPVPIIGTNKCNSSCMYNGEITPRMLCAGYTEGKVDACQGDSGGPLVCQDDNVWRLMGVVSWGNGCAEPNHPGVYTKVAKFLGWIYEIMECVDSVRYLFRETYVSSNCYPLGLEMSSGGGPGNVVQQVNQLWSMLEDLCQNDPEAYRSFMEKQLKSGMDYNAPPRLDSCIRTDMMDPHGGSLYINICSWKRVPASQDSNKPIPVYAGSLETHKDEGAYTVLDIAFNPEVLEDCKKDKAGMDQVYMLALSFAQQQHGLMLSQQYNVVSLCPKNGPEDLRRRLGFQQQQQQRLMTDKQSNAASQSPDSLLEQINSLRLAKDDTGPPVEIVCQPAVRKKGLIEVISSTTFEQPEKPEYRLEVKTLDEGFPCKLELTVELPKISSMSEWQLKMSKDDAMLEVEDIYYLLVDFPSAVNENSAVAIFNKRRRRLTVTADIL

>Glyrichin

MPVAVAPYGQGQAQPSCFDRVKMGFMMGFAVGMAAGAMFGTFSCLRIGMRGRELMGGVGKTMMQSGGTFGTFMAIGMGIRC

>Ap2

MPTTEKDLAEDAPWKKIQQNTFTRWINEHLKCVNKRIVDLQLDLGDGLRLIALLEVLSRQKMHRRCHPRPNFRQMKLENVSVALEFLDNQNIKLVSIDSKAIVDGNLKLILGLVWTLILHYSISMPMWEGDDHESDSKTPKQRLLGWIQNKVPDMPITNFHQDWRTGKALGALVDGCAPGLCPDWETWDPVKPVENATEAMQLADDWLGIPQVIAPEEIIDPQVDEMSVMTYLSQFPKAKLKPGAPLKPKLNPKKARAYGPGIEPSGNRVLRPAVFTVDTFSAGHGQVTVYLDQPDGTRQELKVEPNDAKKTYAVSYVPQVVGTHKVTVLFAGQPIPKSPFEVGVEKAIGDASKVALKGPGIEPVGNIANKPTYFDIYTAGAGMGDVTVMIKDPQGRDNSVEVMMEDKGDNLYRCTYRPTQVGLHSLAVTFGDVAVPKSPFTVDVGPGCLPSACRATGRGLQASGMRVQQVGDFKVDTRNAGSGELKVVVQDASEWKHLCANAGPFEVVVGPEAEPQQLRAWGPGLEGGVVAKPAIFVVESMGSDVGVLGFAIEGPSQAKIECEDQNDGSCDVRYWPTEPGEYAVHVTCDDDDIEDSPFMAYILPDRHSNYPGKVQAFGPGLEKSGCLVHQPAEFTVNAKDAGNGPLKIMAQDAEGLPVEVKVRSKGEGQYSCSYTPASALKHTLAVAWGGVSVPGSPFRVNVGKGSHPGEVKVFGPGVERAGPKANEPTHFTIDCSGAGDGDVSVGIKCNGNMLGDREADVDFDIIPNANDTFTVKYIPPAAGQLTVKVLFTDKEVPQSPFLVNVDPSHDAMKVKAEGPGLARTGVESGIPTHFTTLTKGAGKAPLDVTFSSPVNNFDIIDNYDYSHTVKYTPITPGPMSIAVTFGGDPISKSPFTVGVAEPLNLNNVAVDNLDGRVEVGQEQQFLVDTKGAGGQGRLEVEVLSPSQRCVPCKVEPQPGKAEVRRVRYTPTEEGAYVINVSYDGHPIAGSPFPVEGLLPPDPSKVKAHGPGLTGGFVGNQAEFTIDTKGAGTGGLGLTVEGPTEAKIECSDNGDGTCSVSYLPTEPGEYLVNIQFEEVHIPGSPFRADIHMPFDPSKVVATGSGLNKAKVGEASVVNVDCSRAGRGQLSLEAIPDPSSASAPAGVKAKTEVLDNKDGTYTVTYVPLTSGMYTLLLKYGGKPVPGFPAKVMVVPAINVSQVKVSGRGVDGKVFREATATFTVDARPLSRQGGSRVNAEIRNPSGALTDCLVKDMNDGTYSIEYTPYENGDVSVGIKCNGNMLGDREADVDFDIIPNANDTFTVKYIPPAAGQLTVKVLFTDKEVPQSPFLVNVDPSHDAMKVKAEGPGLARTGVESGIPTHFTTLTKGAGKAPLDVTFSSPVNNFDIIDNYDYSHTVKYTPITPGPMSIAVTFGGDPISKSPFTVGVAEPLNLNNVAVDNLDGRVEVGQEQQFLVDTKGAGGQGRLEVEVLSPSQRCVPCKVEPQPGKAEVRRVRYTPTEEGAYVINVSYDGHPIAGSPFPVEGLLPPDPSKVKAHGPGLTGGFVGNQAEFTIDTKGAGTGGLGLTVEGPTEAKIECSDNGDGTCSVSYLPTEPGEYLVNIQFEEVHIPGSPFRADIHMPFDPSKVVATGSGLNKAKVGEASVVNVDCSRAGRGQLSLEAIPDPSSASAPAGVKAKTEVLDNKDGTYTVTYVPLTSGMYTLLLKYGGKPVPGFPAKVMVVPAINVSQVKVSGRGVDGKVFREATATFTVDARPLSRQGGSRVNAEIRNPSGALTDCLVKDMNDGTYSIEYTPYENGEHSVQVLYEDTPVLRNPARVCVCDGCDPSRVVATGPGLKKGLTDKNNSFNIITRGAGTGGLGITVEGPSESKMACKDNKDGSCSVEYVPFTPGLYDVNILYGGEHIPGSPFKVPVEDLVDPSKVKVYGPGVAPGVRAHIPQMFTVDCSQAGLAPLAVTVDAPKGLSEPVEVLDNGDGTHTVSYTPSVEGTYAVAVQFAEEDIPRSPFRVRVLPTHDARKVRASGPGLTSQVLASFPVEFNVDAKQAGQGQLSVLITDQDGKPKLPTIHDNGDDTYKVSYVPDRAGRYTIVVKYGGDDILDSPYKVQAMVTGDASKCSTTGPQVGPREELDQELFLLVNAKGAGNGKVNCVVVQPNGSEVDAQVTDNQDGTFDAFCTLLEPGDYIMYVRFGGENIPGSPFKVKATNEVPAMQELTSIQQQAAPPGVGFQPWVTSEDFGNGISGVGFRPFHMVIPFSFKRGQVTGEVLMPSGNCAQPLITDNLDGTVTVQYSPTEAGLHEMHIKYNGAHIPESPLQFYVNRSSSPSVTAYGPGLSYGVANKTSAFTVNTEDAAEGGLDLAIEGPSKAEISCVDNKDGTCSVSYLPTLPGDYNILVKYNNKHISGSPFTARITEDKQRRSQVKLGSAADFFLNINETDLSLLSASIMAPSGREEPCLLKRMANNHVGISFIPREVGEHRVSITKNGRHVANSPITIMIVQSEIGDAGRVRAHGDGLVHGTTFTDASFIVDTREAGYGGLALAVEGPSKVDIQTEDMEDGTCHVSYCPTEPGNYIVSIRFSDKHIPGSPFTVQVTGEGRIRESITRKQRAAPVAGVGSVCDFSIKIPALDMRDVTAEVTSPSGALQSAQLVAVGNDTYCVQFVPTEMGVHWVSVRYRGNHVPGSPFQFTVGPLGEGGAAKVKAGGPGLEGAMVEEPAEFNIWTREAGAGGLSVAVEGPSRAEISFDDRKDGSCGVSYVAQEPGDYEIAVKFNDQHIPHSPYLVHVMAPTYDTQHMTVTGLQKSGLKVNQPTSFDVSMEGAKGKPEAKVTSPSGSQEECAVKHLEKDKYSIRFIPREQGLHTVDVKFGPQGRVTSFSVMVGSPEQAGQAGLVSAYGAGLERGTTGVQSEFIIDSAAAGQGAVAVTVEGPSKVQMECLEVAGGYKVLYTPMAPGSYIVAVKYGGPYHITGSPFVAKVTGPPLVNVTNSSMSSSLTLGPSAEASVVSSSGGSLQSVNCVSAVSDASKVLSRGLGLSKAYVGQKSSFSVDCSQAGKNMLVVGVHGPQVPCEEVLIKHLGDLHYNVSYVLRERGDYMLVVKWGEDHIPGSPFHVTVP

>CDK-like16

MREGRQQHLTEKCDVQDGSVFFGFPRAVGSELADTWSGSGVPTAGVESAVSRWNGVEHGKQGQGADAEPPSQVTGDLLPLTWQLKKLSEDSLTKQPEEVFDVLEKLGEGSYGCVFKAHYRETGEIVAIKQVPVESDLQEIIKEISIMQQCNSPHVVRYYGSYFKNSDLWIVMEYCGAGSVSDIIRIRSKTLSEEEIASILQSTLKGLEYLHFMRKIHRDIKAGNILLNTEGQAKLADFGVAGQLTDTMAKRNTVIGTPFWMAPEVIQEIGYNCVADIWSLGITAIEMAEGKPPYADIHPMRAIFMIPTNPPPTFRNPDSWSAQFRDFVSQCLVKNPEHRATATQLLQHAFIKAAKPSSILRALISDAMDIKAKRQEAAAALAEQDDDNEYDNSEEDEVDQGTMVRAGPGDSATMRAAGSLGNTLNAAGQRDPDNGEEEEVGTMRLQLGTMIINSEDEDEAGTMKRTDEASPPVKPSFLEYFEQKEKEASSPNGGRGGGGEECKVLSDADLQLVSSWSVEDLRVRLAALDPQMEQEMEEIRQRYQAKRKPILDAIEAKKRRQQHNNL

>Ubiq16

MQLFLRAQNTHTLEVTGQETVGQIKAHVQTLEGLLVEDQVVLLAGCPLEDDASLASCGISEHCTLEVAGRLLGGKVHGSLARAGKVRGQTPKVDKQEKKKKKTGRAKRRIQYNRRFVNVVPTFGKKKGPNANS

>ERH1

MLACPHYCLNLGLCRVHSGSHTILLVQPTKRPEGRTYADYESVNECMEGVCKMYEEHLKRMNPNSPSITYDISQLFDFIDDLADLSCLVYRADTQTYQPYNKDWIKEKIYVLLRRQAHQAGK

>BPTI1

MWPRPPLSCFLLSFVLAARPLGARAEDSACAGSFRSGQENFVVDLEDAVTEGAVLVAVVRAASLPECRAACCALRRCNVAQADANLTCALFDCAPRNKFACRFVRRPGYRSVIRDLEFRKYLQGPQDADEPAPPIAIAGEDVVAQPGATVTLDGIQSLAIGDAHIVRYDWTLQSGEADVVLEKTALAEQVRVSNLVAGRYRFRLTVTDSNERSHDAGVQVLVLSAEMSARYCLAEAKVGPCRAAFPRWRYDAVEQRCQAFVFGGCKGNLNNFLSRDDCVKACGGVSVTSSELQRIALPAGEVCGSACVPGQLSCGDRCCLDATLECDGVTQCSDAADEKHCGQLNETFTQLLEIDVNQRKARCTEPPQTGPCRASLTRWYYNPLNTRCQRFTYGGCHGNDNNFAQETQCDTSCHGVTERDVFARGTFERFEDQEEEESSSGSVALAVILSVAVLTLLAVLAYCFLKKRKERSGGHADAGAPRQPASEQDMLVYNSTTEPLEHPRLQLAHMDTS

>ERH2

MSHTILLVQPTKRSEGRTYADYESVNECMEGVCKMYEEHLKRMNPNSPSITYDISQLFDFIDDLADLSCLVYRADTQTYQPNNKDWIKEKIYVLLRRQAQQAAN

>BPTI10

MVKSRRCITHRNDGGHNCVGPDKSYLACNTEECPAGTKDYREEQCSQFDGTDFKGSRYVWVPYYGAENPCELNCVPKGDNFVYRHSAAVKDGTPCHPGRPDICVDGVCRRMGCDNVLDSRVQEDPCLQCGGHGQSCSLVKNTFNTQRLAHGYNQMFTIPAGATSISIREKRPSRNYLAVRNLQGDYYLNGNWLLESTRATHIAGTKLFYQRGVEGDNIPETIIGRGPTTEPLVVELISQEPNQGVEYEYYLPVGHPTEGYSWSFGSWSACTKECGSGHQSRAVYCSIDNEVVPDYLCVYYARPENNRTCNVQACPITHSWRTGEWNDCDAPCGGGFQYRGVDCLYNDESGPRVVEDAYCAQYSQAPTDQQKCNMQACPDYGRAIISYIPAENAVQCRTTTYGCCYDRTTPAAGPNGEGCRDPPAPYERSICSLPKAAGSCASWTARYFFNVLTNKCTEFWYGGCHGNSNHFATEEECHRHDNGNGNGNGNGNDHSNGNGNGEHDNGNGHDVSNGNGNGEHDNGNGNGHSNGNGNGEHDNGNGNGNGNGHSNGNGNDHSNGNGNGEHDNGNGHDVSNGNGNGEHDNGNGNGHSNGNGNGEHDNGNGNGNGNGHSNGNGNGRSNGNGNGNGNGNGNGHSNGNGNGEHDNGNGNGNGHSNGYGQRSNGNGNGHSNGNGNGEDDNGNGNSENGNGNGRSNGYRFKIASRVAHTAHKGRVYMKARKPYFIIVKKHYIPGASLTMQAQVRIDQSDPSAVEALVGQTVVLPCRVSPPPSASSWSSSAAVVVEWRKDGAALTSRRQQQQPNGSLLLGPISKSDAGWFLCVATREQERDHRYVYLTVSEGPSQPQPTTSPTDKLSPGFTLERSAPSLLETLVGNTVRLSCTVVPAAALPFVSIQWTQDGRAISDSRFVQQSDGALLIESLRTEDTGMYTCSASTQHQLEQRHVQLRVQAAVRITKAPDNIQVPEGSTALLPCVASGDNVSIGWSRNGVPVRPDGRKVQVSPDGSLILNNVQVFDEGTYTCNAYAGVYSVSASADVRITKDGHTGVLVTSSSSLGTPCLDQPKLANCELVVYARMCTNPYYAAFCCASCTRQARSGDRLAQ

>CDK-like5

MDKYEKMGKIGEGSYGVVFKCRNRDSGQIVAIKKFVESEDDPTIRKIALREIRMLKVFRRKRKLHLVFEYCDHTVLHELDRHPQGTLLKMVVVVVVVECSVPEHLAKSITWQTLQAVNFCHKHNCIHRDVKPENILITKQHIIKLCDFGFARILTGPCDSYTDYVATRWYRAPELLVGDTQYGPPVDVWAVGCVFAELLSGIPLWPGKSDMDQLYLIRKTLGDLICRHQQVFSTNHFFHGLSIPEPQQLEPLELKYPNLSHQTLSLMKACLRMNPSERLACEQLLQHPYFDSMRHKSLPNAKQVNINAGHDRHGNRRTRLPRKHLPAGCLPQLTGSSIFPAPDTKKYFHNLNKFNYHLPNI

>BPTI5

MLSRELLCAFRLFCVCLLGTRGAAAQETGAQPELFIFDELCALKEDPGPCRALRDKYFFNVDTGRCELFEYGGCGGNANNFETLEACRETCLVSEDKNPCHLPEAEGPCRGLLTRYFFDNSSQQCKRFYYGGCFGNANNFRSMAECQAKCRQPEGVQVQTGSGEIVDVPSTALSGQQTVNVAVAQTNNTDPDHSQDAKLPDACSAPVAKGTCDGSVRRFAYHAEAKRCHEFLYSGCGGNNNNFKHRRQCIRKCIKSQKGIFFSSPFTQFQCSYLPRLWRHLLAPQIKKMSFCVGGSYVNSSKSSQHFRNKQRTKD

>Lectin18

MRKSPTRQVCWQAELLTGAYIPHSLRMMKEKNSSTFLSRSLSTPMGPPFTSALSRPRQDVLPSRYGFVDFDSPAAALKAVHALKTSGIQAQMAKQQEQDPTNLYISNLPLSVDEKELENMLQPFGQVVSTRILRDYGGNSRGVGFARMDTTEQCNAVISHFNGKFIKIASGALAPSQPLLCKFADSQRKKHAHGGFVPNGQTGDLRLGAMTLTYDPSSAAMQNGYYPSPYPVPNRIMTVQPAMSPYMSPLSAYQGPVMSPSVDPSMSLHPSTMITQQMGQLSLGNTGAYIAANPGAQGAYIPQYHPLPAAPNFTNWLSVNERCDTPEHLLEKGCAESLLEFPISKGEILQNQPLGKKSGNLNLTQISPQKMALKLRPGSQVTFQVKIQHTEDYPVDLYYLMDLSASMFDDLNMIKDLGSTLSKEMANLTSKFRMGFGSFVEKPVLPFIKITEEELANPCRSADATCLPTFGYKHVLSLTSNTGKFNEMVSMQHVSANIDVPECGFDAVMQAAVCGDRIGWRNDSMRLLVFVSDADSHFGMDSKMAGIVIPNDGQCHLDTNNEYSMSTEQVLTPFEYPTLGQLIDKVVENNILLIFAVTENQKLNYENYANLIPGATVGVVAMDSRNILELILTAYKVVFNVSVELPGCLSGSRHFSLKPVGLQDTLEIELESLCSCECGRPPEADNSSQCMEGQGTFRCGVCECQPGFSGAECECNEESALLSNCVANNESDICSGQGQCYCGECVCDTSNFGHIYGPYCECDNYSCVRFRGELCGGHGVCDCGECHCQSGWTGEYCNCSSSSDACLSEEGVMCSGRGMCECGHCVCLVSGASGDLCEKCPTCGDACSVARTCVECHLQAKENAELCELNCSLPNIYINTTAEVDKSAFMQCTLISENECWISFSVLGGDTMTVYNPHMYGCPQPPNILMIILGVSLSIVCIGLILLAVWKVLVSVHDGKEVARFEAEMAKAKWQSEEEDNGEILEQDELAEFYTKGLFILESKLLQRCVTVEQSNLVLRDCSRPTRRMLWKWVSHHRLFNAGTSTCLGLNMSDPAQPLGTFQCDMDLPVLWWRCRGNMLYGTSRWKVAVAGNLVVVKRNSYYEWKRYNTPREGPCSNPYEDIHTLLGNAHGMPCALPFKYNNKWYSECTTEGREDHLPWCSTTTRYDEAEKWGFCPVHDYSCEHFWESNQELRACYQFNLYTILTWSQAMLTCQAQGGNLLSITSLAEHRYIRALPSSPLEDNRQCGVYNSGPDGLWQSLSCESALPYICKKTPNDTHRAEPFENWQYVHTVCDEGWLPHNGFCYQILAHAGSWEESSQACISRSANLVSLHSLSEVEMVHSLLANYSGEASAMWIGLRKQVSSATVEWSDGSPVTLTMWHQYHPPHNLTGATLCAKADRKEGAWVLVSCDEQLPAMCRKVKHQTSAHELGTWDEGCPEGWKRHSHSCYTITKQEQTHKDAQSGYFCNAPLLTVENRFEQAFVNSLLSANGANGSMSYWIGLMDSGERGEYRWLHNNGSTQPLTFTNWNKHQPVSAYGCVAMSGGSALGQWEVKDCNSHKALSVCKQSISSYQEVELLPEAHDAFSACPPGWESQPGLLYCFKVFHDEKVLLKRSWVEADYFCQALGAQLASFRHYEEQVFVKQLLSTMFEGTEGRWLWVGFNKRNPEHPGVWEWSDGSPVMTSFVEDKNDEDDRRDCAVYSDLPNSLMPRPCESKHEWICKVARGSEPWVFYRGAEYLVAKHPFNWDVVSLACKMMGAYLLSIHSREELNFVKERLRRLSLGPTDWWIGLSIDQHGEEVRWSDKTEVNFHNWAQESDGHHDKKTGWCVTMSSSTGKWTANKCSNLHGFVCKRKTVSVVETPRELHYIGRCPEKWLYFGHKCLLLHLPDTPSEGKSWNDARSICSSFQGSLVAIEDEIEQAYITMLLQGISVGVWIGLWNQDTMKWTNGRPVSYTNWSPIEPKSYLNEDELLTGFADEPLCTVLSNNHNVHLTGKWYDEKCSEDGYGFVCQKPQDTSKRPTHSSRHPLPDSIEYKSRSYKVLSGNMSWYDAMHMCMDNDSDLVSITDAYHQAFLTVLVNRLGVTHWIGLYSQDSGINYQWSDGSDTVYTNWDSAEDDFEIGECVYMDVNGGWRRADCETLLPGALCHIPPPRNKPFISYEAVCPSTWVKFGRGCYNFEPLVQRLTFEEAREHCRRKVNTSDVLSIENEAENRFVLEQLWTSGFLHQNMWLGLYFNIDTDSMAWVDGSPLDYSSWPSKAPDVKLLTADTCVTTRAVDGVWLLLGCTERLGFVCKTISDVTTEVEVEPFNGLHYGVILAAVLVAVLIFALLAGALCFAYRRNTAHFRGLPALGSAYYRQSGSQATESDGLHYGVILAAVLVAVLIFALLAGALCFAYRRNTAHFRGLPALGSAYYRQSGSQATESDVEDSFTVTVEDSFTIQHSLTGSCLSAQDSGSLGVSACDANSTSHLWKWGSGHRLFHVATALCLALDVHSKSLSLADCGSDILWWRCLDGAVFTVYQMSLVVSGGQLAAQRDAHDAWVRGGSQESICRRPYRVVHTTNGNSAGAPCEFPFKHNGSWHHECLPDSDLPGLSWCSTTTDYDKDRKKGTCLKPEKGCQTLFTGPEGNSCYQFVPNAAVTWHQALDSCRSQGADLLSVTTLDDLHSSTFLDGLNMMPERMWIGLHQLDASQGWQWSDGSPLSMLRWETGMPPVSLIIASDCGVLNTKQKYEAEACSKQLPYICKKIVNTTQTGPSDGQQLNVWIGLVGEGTNPTVFKWVDQTPVTFTFWGPNQPLQPSQDISCVFYSSSHDWQVGRCSEKQAFMCQTKGEVSESTGTAGCRFEDGWRRHGNSCYRVNTTQVSFKDRCHITIRNRFEQAFISRLLGDYISNTPQYFWIGLQDVKSTGEYQWHGQDDSQVQVTYTNWGSFEPAHDGGCAVVATSNGLGKWEVKNCTLFKAGTICRTDLSPVIPPEPEPDLTLPCPDGWVSVPDSKYCYKVFHEERLSRKRSWEEAERFCQALGANLPSFTTLDEMRVLHGIMRDTISDDRYFWVGLNRRNPADRSWQWSDGRPVTMDVLHHDFQEDDAYSRDCTAFKCYTLLQNVKPVSFKTANEVCLSVRGTLVTISDQVEQVITCKVLLLLSLDFINSLLPGMKDMGGIWIGLKQKRNKLEWLDKSPVNYVNFHPLLIGMHKAINVNVTMDVLHHDFQEDDAYSRDCTAFKTMKRTMKHLLIFLFHDLYPTSFHATPFHCDAKLEWVCQIPRGVHHETSIFIDGSEYWFVNEPRLTFEEATLFCTMNGSKMASPPSSTAARLIHENLEKAKYEHSCRQQLPFVCERLNITLVESNPLEAHPGGLPCENTSLAFRNKCYTLLQNVKPVSFKTANEVCLSVRGTLVTISDQVEQDFINSLLPGMKDMGGIWIGLKQKRNKLEWLDKSPVNYVNFHPLLIGMHKAINVNTLDPDNMDLCVFLINNPSSAVMGTWDYTSCTHYQHGAVCQHYADQVEEPSVTSEPFQINNHTFQLLIKNLTWFEALEQCMEQEMDLASVADTLLQSTLSVHVSRARTPLWIGLFSEDDGIHYRWTDHSHTVFSRWSSEVTEGACVYLDTDGFWKATECEQTLGGAICHKPHKEIITTPEDVAVKCPHKINGPNWIPFKNNCYAFHLAASRWEQMDQGPIRRTCVNLHKDADILTIRNAEENEFIRQQLLPFQNLAQFVWLGLVKNNTIGNHLEWYDGTTVQYSNWRNGRPEVDGNFMAGVNLQGKWVLITNRMLFSEFKQRAVVTCKLDNEPKEKYSKSTRDYQHLGSLTYEVVTQRELSWYQALELCAQRGGHLASVHDNEHGAHVKLLAKTDGFPLWIGLSNQEAGRETYEWSDGTSYQYKADISESVEGASADERDVGGCVFVMPSGAWVRSSCYTLLDGAICYNTNTSTPFQKAKLQARARVNSCPQGNGTSKWVQRDDRCYAFDMSFYNYSVYDMQRAKSICQSMGAQLLAIKSKEDNDFVAKYVSDDPLITSRVWLGLDIDAQGSPTGWEDGSALSYSNWKSGALVSAAQRKEAPCAIMATEDGGSWRVVNCQASLSRVVCQTKAESSGSPVAVGFFVLILIALALAVAFIVYKKQRGHFSSSVRYKRTTDQSDTTSIITEELRDPARVQSATTHIPLKSQVFFSPIRLELHLRKTIPQTL

>Throm5

MVRKGSMELIKESGSSRGLSTLEIWLMILFLVMTGVSVALVVLYFVQNEEDPAPLQEGPDSGCGGPQELTAASGTFTSRHYPSSYDNGRSCSWHITVDPDKNNCVISPGPDSGCGGPQELTAASGTFTSLHYPSSYDNGRSCSWHITVDPDKVISLWFEEFALEDTNLCTADFITLRDSLGVIGKYCGYSKPMPLVSLTNRLTVYFDTNDRKTDQGFKARYEAVSPERTSAGAGGALHGDRGDLLTPGFPEQNYPNGALYQWTITVPAGERVRLTFTSFDLVPEVCGDFVQVYDGSLSLGKFCGGALPEPVESSTNTMMVRFKSDSSKSSRGFSATYTKSSLPPIVIPTTVKPTTTTSKPTTQTLPPTTTSTGGPVIISGRKGVIQSEGFPNPYPAHQNVSWRIIVPKGFLVKLHIVELAITGETGQCEEDKLVVSDAYSTLGTHCGYIRPPVAVAATNTLSVSFSTDSRLSDRGFSAKWEAAYPEDIAEIQGCGLSSKEDIGVMKSPNWPMNYKANAECMWSIAVPLGKKITLTFTHFDLEPRDLLSFKCFDNVVVYDINGLTNVLNQKHGPFCGTKIPRTIQTKGNRLVVRFHSDLFAEAKGFRAYWTTNPSLPAPTEPPVPPNPWDGINIDWPSTCGKPAIPPAIMSRIVNGEPARPHSWPWQVSMQVWPSSQPEPTFFHTCGGTLIHKNWVLTAAHCFINYANELHRWQMCLGKHNLTFTEPSERCLGVRGIYRHEGFRYPTVPTVEFDIALVRLDGEAEIGDEVAYACLPNPEEVLPEGKKCYATGWGDETGDSMNAKVAEALNQVALPVVPYDTCKRMDYWWFQVKPSMICCGYTLPDELKSVCQGDSGGPLVCQDHPDSPWEVHGITSFGPIGCIMNKKPSVFTRSSAYLPWISNVIRRDIYEQHTSGCGGAKDLTGTGGTLSSMGFPGSYSNKARCQWNIYAPAGKLVHLHFSNFSLEESPMCLSDKVSILDGTGSLGTYCSHKPPMDLVSDGNSLHISFSSNDKVVDTGFAATWKVVDPSEAPCGGTFSSPQGELTSPDWPKDYPAQAVCTWRIRVPTAKSIHVVFTHFELQAVNLLGNCVDYVEVFSGGNMMSQGRFCGFAAPPKLNVPGDTAVIRFLSNSANRQQGFRGYWTTDAGVVPTLPPPTPNPWDNITISWPEDCGNPAVAPNTGAAKVVNGEEAVPHSWPWQVSMQASPMSPIPYMHGCGGSLIHEEWILTAAHCFMAPLNQISYWRMCLGKHHMNSSRDVPSAEACYKVDGIIRHKDFVYEEDPSDITHDIALVHLATPVNITREISPVCLPRPGVVVEAGTRCFVTGWGDEKGNMFPKVSEKLNQAALPVVDFETCSKPAYWWDTLRPSMICAGYESPDELKSACQGDSGGPFACRAGGGANATWEVHGVVSFGPQGCIRDKKPSVFTRVSAFSHWIHDNIRKFMYERDN

>Throm48

MTGLRLQRSPSQTPFQWANPSPEESTRPLNPKHVVVPRPGRHRRPMTAPKTQKEKASKTKKVLLTIAAVVVLLGILVTAGYFIKQLIDSKYFFCTKSFKFIPLDKACDGTDDCTGGEDEQTCLSNLKVNTTFPVRLMTRQSVLQVYNPSSGWRSVCSEGWTDKHTQTACTQLGYTNKPRSINVPVNTLLSNLKSGPFTALRSGTGNTPVHQATADRSACRSGAVVSLSCSDCGEGDAQNRIVGGTDATIEDWPWQVSLQQGGQHTCGGSLVTLRWVITAAHCFAGSSKKELSRWKVVSGRTYMGTLGGSSVDRIILNGNYDPARNDYDIALMRLTSPITVGVSRMPVCLPPKAFDLPPTAATVVTGWGYLEENGIISSSLQKANIPLIERNKCASPTVYGSAITNRMICAGFLEGKVDACQGDSGGPLVHFTSSKWYLIGVVSWGVGCAREGRPGVYCNVEEMLNWIHTIIEVRSYSSILSISMKVLTTLKYFNKG

>Throm19

MYTRSERQYKSQGNRCKVFSPVGTAHFVIAMSSDNALDDDDTFKILIATDIHLGYLEKDAIRGGDSYTTFEEILKCAKTNQVDFILLGGDLFHENKPTRRCLHTCITMLRKYCMGESPINFNILSDQTVNFNTTQFPWVNYQDENLNISIPVFSIHGNHDDPTGAEGLCALDLLSASGLVNHFGHSQSVEKIEISPILLQKGNSKLALYGLGSIPDERLYRMFVNNHVTMLRPKEDQDEWFNLFAIHQNRSKHGPTNYIPEQFLDEFLDLVVWGHEHECLIAPSRNEQQLFYVTQPGSSVATSLSPGEATKKHIGLLRVKGRRMNLQKIPLKTVRQFFIQDVVLSDYQDAFTVDTPNVIKKVENLCHAKVNEMLEQAERERLGCPLTPEKPLIRLRVDYTGGFETFNTSRFSQKYVEQVANPKDLIHFLRCREKKERIKDELSVDYSRVLKTTAVEGLRVEDLVKQYFEATEQKVQLSLLTEHGMGKALQEFVDKDEKDAIEELITYQLEKTQRHLQGRSVITEQEIDDEIKRIRESKKNTMEEENEIDEAINRAKAHRLERGQTSLDINMSDPPSDVDSDQDAPPARGRGRGTRGRGRGRGAKASEPKSSTRGASKKSQMTSQSRSIMQAFQAPSQRPSRSTLPTSYAEDDLTIDDSDEDVPVMKTRPSSKTASTSSFLRESSQSQMSKGVSFDDSDENEPPPPYYSVAVHTQPPLKSYEEVVYGVSPAQTPANQPHFVPQYATPLAAPQVTQPSLLRYGTRVWTQGRFNSTDTQNESIDENQLILMSDICPNDTINCDGIRDCELGSDEMVCVRFGIRNRLEVKTAQDGRFLPVCFNGWDQSIADQTCTQLGFKRFYSYTNITTQDSLGLIATNTLSDSIQGQVEVRSSCPDQSTVSLQCVDDVHPACLPVTAQQFLPGTACWTSGFGTTDPESGQVSDELMEVTVDIISTIECNNPWVYGGAVTENMVCAGHLEGGRDSCQGDSGGPLVCQADDVWYLVGITSWGDGCALANKPGVYTKVNSLLPWIHTNMQDTRGCGDALRPGKGRRERAAEAVLSPRLVSRYRRVCGTRAGNTCPGKHQAKKAVQSSTEPRPPGRPAAPVRSSFGGEPHHIPVNYNCFEWHVACNFTSAVFFARSSVGVSLPCPAGGAPHAVLRWYLAAGDDIYDVPHIRHVHANGTLQLYPFSPSAYNSIIHDNEYFCTAENQAGKIRSPSIHIKSVFREPYTVRVADQRSMRGNVAVFKCLIPSAVQEYVSVVSWEKDTVSIVPGNRFFLTSFGALYISAVQKEDALSTYRCITKHKYSGETRQSNGARLSVMDPTESTPSLLDSFQSGEVKVGQSVELPCIASGYPNPTIRWLKDGRPLPADSRWTRRVTGLTISDLRLEDSGNYICEVTNSFGSKEVTGHLNIIEPLRVTLSPKNLKTGISSTVILSCAVQGSPHYSVSWYRNTEPVLPDQHFSIQGGHNETLFISAAQKRHSGAYQCFATRKAQTAQDFSIILLEDGTPRIVASFSEKVVVPGEPFSLMCTAKGAPPPTITWTLDDEPVARDLSRVRANQYTHSDGSTVSYVNVSNPQIRDGGLYRCAARNSAGSAEYQARINVRGNPRIRPMKNITAVAGRSSFVNCRVIGYPYYSINWYKEGLLLPDNHRQVVFENGTLKLTDVQKGMDEGAYICSVLIQLQLFIKQTVYVTVKVPPLIQPFDFPPTSIGKLMYIACVVASGDMPIRITWRKDGQEIVPSSGITIDTKEFMSSLQISKVSLKHNGNYTCIASNDAATVSTERQLTVTVPPRFVVQPNNQDGIYGKSGILNCSVDGYPPPKVMWKHGKSALAGIGNPQQYHPVPLTGRIQIMNNGSLLIRHVLEEDRGFYLCQASNGVGSDISKSMALTVKIPAMITSHPNTTMAKKGHVKELNCTARGEWPIIIRWERGDTVIDPDRNPRYSITTSPNEKTDEVLSTLKLKPAEREDSVFFSCHAINSYGEGRGLIQLTVQEPPDPPELEVREVKDRSMNLRWTQRFDGNSVIASYDIEYKNKTDTWELKHATRNISPTNNQANIVDLHPASVYSIRMYSYNAIGKSEASKELTISTEEARKYIAASEKRFYYLCARVTNGTLCIFAEPDGPPMDVILQPVTSQSIRLKPAEREDSVFFSCHAINSYGEGRGLIQLTVQEPPDPPELEVREVKDRSMNLRWTQRFDGNSVIASYDIEYKNKTDTWELKHATRNISPTNNQANIVDLHPASVYSIRMYSYNAIGKSEASKELTISTEEAQPDGPPMDVILQPVTSQSIRVTWKAPRKELQNGVIRGYQIGYRENGPGSNGQYSIVEMKATGDSEVYTLDNLKKFAQYGVVVQAFNRAGTGPSSSEINATTLEDVPSQPPQNVRAISVTSDEAVISWAEPPRMTLHGVLKGYRVIFWAVFPDGEWGEMQNITTTKEQVELRSLEKFTNYSIQVLAYTQAGDGVRSNVLYIQTHPGPPAGIKAVPSSASSVVVSWLPPHKPNGIIRKYTIYCLSPGSGQPMPSEYEANPELVFYRISHLNHRQQYLIWAAAVTTAGRGNFSDKVTVEPAAKAPAKILSFGGTVTTPWMKEVRLPCNSVGDPTPTIKWTKDSENTAIPVSLDGQRLIMANGTLVLRTVKAEDSGYYTCTATNTLGFDTIIVNLLVQVPPDQPRLTVSTTSTSSITLAWIPGDNGGSSIRGFVLQYSVDNTEEWKDVFISSTERSFKLDNLRCGTWYKVKLAAKNSVGAGRISEIIEAKTHGREPQFNKDQPVFTHINSSHARLNLQGWASGGCPIGAVTLEFRPKGTWAWQNVRTNATKEVFLAELREATWYELKMKACNSAGCGNQSSQFATLDYDGSTIPPIKSPLEKNDDVKKLFSIGCPVILVTLGVALLFIVRKKRKEKRLKRLRDAKSLAEMLISKNNRSFDTPVKGPPQGPRLHIDIPRVQLLIEDKEGIKQIGEDKATIPVTDTEFNQTGNPQSFCAGVSVHHPALIQNTGPLIDMSDIRPGTNPVSRKSVKSAHSIRNRYSSQWTLTKCQASTPARVLASDWRTVSSQHGITVTESDSYSASLSQDTDKGRNSMVSTESASSTYEELARAYEHAKLEEHLQHAKFEITECFISDSSSDQMTTGTNDNADSMTSMSTPSEPGICRFTASPPKPQDYDRGKNVAVPIPHRAKSDYCNIPLYMKSDPFFRKQSDHHDPCPVVPPREASIRGLAQRTYHTQGRHMTMDSTKQQALTMGHSGLSNLTSSGTSAGGASGSGGMSASSSCSTLPQRTVTMPGSSATGAQSAAAAAAATAAAAAGSSSSGGAVGATGGTPGGASSSSSSKMGGSRDSLLESSSSGLGRLQKQRDGGAGAYSKSYTLV

>Throm11

MPPPPPPLLALLLLAGLRVVCADAGNVTRGARRGQRHSGNFAGRYGTASDASRGTLTYRFSRSFAAFRVRWTNCELWELAGVRSFAAHQKAATRIIGGQEAWAHSWPWQVSLRFATMPACGGAVLAPLWVVSAAHCFKRCRAAALGDRDAAAFELLCRRRYNKASFWTVLAGKHDLDDPDEEGQQVLPIALPHIAAAAFAFYKLANAAQVADVSAIVSHHRYRSGSKEFDVALLRLERALAFDRFVRPIRVWMAPLPTREKCTVTGWGATRENGPRATRLQEVNVTVMTLDLCQSYYKSRIRTRMFCAGQEAGGVDACQGDSGGPLSCYDGGRYRLAGVVSWGVGCGRARKPGVYTKLQEHAGWIADVIGKNLAGGSVVGPDTRPLRCLSENQDVAYDLKPSEERRCGWRREAVCRNLPGPAGLRVPRAGPARAENLTEACPGAWPWQVSLQANGVHYCSAVLIRRRWVLAARHCAKAATRIIGGQEAWAHSWPWQVSLRFATMPACGGAVLAPLWVVSAAHCFKRYNKASFWTVLAGKHDLDDPDEEGQQVLPIALPHIAAAAFAFYKLANAAQVADVSAIVSHHRYRSGSKEFDVALLRLERALAFDRFVRPIRVWMAPLPTREKCTVTGWGATRENGPRATRLQEVNVTVMTLDLCQSYYKSRIRTRMFCAGQEAGGVDACQGDSGGPLSCYDGGRYRLAGVVSWGVGCGRARKPGVYTKLQEHAGWIADVIGKNLAGGSVVGPDTRPLRCLSENQDVAYDLKPSEERRCGWRREAVCRNLPGPAGLRVPRAGPARAENLTEACPGAWPWQVSLQANGVHYCSAVLIRRRWVLAARHCAVSAGDDVAVLGGHDLGLSLSQTVPVDRVFDPPQEDGFPPKNDLSLLRLAVPARLGAGVTPLCVAEEDEELDDGWRCISAGWGATSATAGLNAERLHHAGVPLVERAKCRAEWGRGRVDEGHVCAHPVAGAACAVRYRLPSVRRPDFDERPGGSTLAAVLAQGDSGAPLFCQKHGTYFLFGLLTWGSRRCDPGKPAIFSKVADYHSWIGEVTDA

>Histone14

MSARGGKKKVTKLSRSSRAGVIFPVGRMMRYLRTGTHKYRIGMGAPVYMAAVIEYLAAEILELAGNAARDNKKGRITPRHIKLAVANDEELNQLLRGVTISNGGVLPRIHPELLSKKRGSRVKADSQASVVPEKKALRAKSTKAIKPSKKVKGKRGRKPKVVTEIGKIGTIKVDAIINPTNAEMDLRDGVGNALEKAGGQEFLEDVKELRKAQGPLGVASVAVSQAIGMTARVIIHCNVPQWGSDKCEEHLEKTVENCLSAAEEKKFKSLAFPSLPAGRHGFPKPTAAQLILKAISNHFVSSTNSSLKNIYFVVFDSESIGIYIQEMAKLDSK

>Transfe2

MSTMKHLHLSHLRSTLLLSYLVAVVLSASTNHVRWCLKSDKEHQKCLALSTKAPVFSCVKKSNTLECLTAIHEGTADAITLDGGDIYTAGLDDYKLHPIIAEDYCAASSSCYYAVAVVKKGSGFGIRDLQGKRSCHTGLGKSAGWNIPIGTLIKLGVLKWGGNENETIESAVGNFFSESCVPGAEKGSQLCKGCKGDCSRSHNEPFYDYGGAFQCLVENAGDVAFVKHLTVPDGMKADYELLCMNNTRAPIDSYKECHLARVPAHAVVTRPDFKLADLIWNSLTTVANFDLFSSEAYAPAKNLMFKDSTEKLVRLPANTTSFLYLGAAYLSIVRSLNKEVTAAGSNAIMWCSVASSETQKCNKWSVNGMVDGENMILCQTAPTVQDCIKMILHNDADAMAVDGGEVYTAGLCGLVPAMVEQYDEASSYYAVAVVKRDSGVTWANLMGKRSCHTGFGRTAGWNIPMGKIYKQTGDCDFTKFFKSGCAPGSPTGSPFCSQCAGNGKTVDDETKCKANSEEKYYGYAGAFRCLVEGAGDVAFIKHTTVAENSDGKGPAWAAGVLSSDYELICPSKGPVPITDFEACHLGVAPAHAVVTRPETRDVVVSVLQEQQASFGRSSRNAPFKMFESAPERNLLFKDSTQCLQEVPSGSNYQSFLGPDYMDAMTSLRQCTDSTPGFFGGVGVPA

>Lectin15

MIPTSSPVNPNATSVFSIDYLRIKWTKLEGDVESTVLVAQNGIIKIGSSYRNRVSVPSHPEIVGDASLTMVKLLASDAGTYRCEVMFGIEDTRDTVDLNVDGVVFHYRTNNSRYTLDYQKAVQTCEDIGATIATYDQLKAAYEDGFDHCDAGWIADQTVRYPIVNPRKGCFGNLQNKPGVRSYGTRKPTETYDVYCYVDKLYGDVFYAPVSQKMTFEEAKEECKKRNSQLATPGQLHAAWRRGLDRCDYGWLSDGSARHPVSVPKLKCGGGLLGVRTMYRYRNQTGFPEVTTQLGAYCFRGRKLVINQTSFVDVFVINDTSTTFSSSAVPLLESSSVAQTQTSETGSEAEEDTVTTDSPSMFSTSMVPPRPTPAGTEEKLFTTVAPTILEDHEEDIDDLRPVDPDFDVDDFGHENVTFLESVPHRGDTLPEAMEDPDDHSVIEISTIETDAVLHDASPRTEPMFAEGETEETILNSNLTDADTDVESEHVISSSKTTPTDSTLPFPFYDEIETDLLVEAPPPAVTGPSQDILHSTDETTTIVIPASTIFMCNTKPGGTEVDVSTATPSPLETTTQNKVLIQHVATPEESLFDTGTGTPMLVEDVTSDDGSMSVFDQSTDQVSEQNHDTLTLIDTTENIDAEFFTPAAVVLTANSNSTAPEATVTKESSTKETTTTMPEQNVSDAQILQSQNSQSPVLLNPSITSMVDGITSITGKPSVSLVSDMTNPIPTISGPTMRSEVASFSSTTLKESSGDGADVFSGASEITPTSISSMFSTKTPRATSQEDGIQDSSKKPLTTASSLYSTEKTTQVHPEMQNSLSSATSKEALALTPVDRDGSGEQTQNLLHQTSSPNTESPNITGASPAESPVRLSTLPDIPIEIVSTELNIPEKNGSFPENQEEGSGEQKTEESQKLPASPNTSLFLSTKQTTESHEPGITSITGKPSVSLVSDMTNPIPTISGPTMRSEVASFSSTTLKESSGDGADVFSGASEITPTSISSMFSTKTPRATSQEDGIQDSSKKPLTTASSLYSTEKTTQVHPEMQNSLSSATSKEALALTPVDRDGSGEQTQNLLHQTSSPNTESPNITGASPAESPVRLSTLPDIPIEIVSTELNPSDNEPSITPETDYFRDIDDGIMGGVLVESLPSLHGTTMMPGMSIDIESSGDSINEFRGESKVKTTTVSSMFSTETPAVTASHEDVTQDEKSSTATSSLYSTTKTPPMSHHITTSVQVQVDGSGDQTEDIFIQTSSVTTATLSITEALAEPQTSPLTVTNVENYDVSTESISSVIGSSITPDTESLSLFSTALNEGSGSFPSLSESTIQPAMSSSFTVTNTDGSGNSIEDFTGSVVSVSRTDTPAVTVSYGNETNTFSNTSIAVSSLHSTEAPTAEPMIPSTSVLGANMTPETNISVSTIDGTVALVDQTGKSFISATTSSSSVFSTGTLSMRVSHETTSEPHAHVIKNEDSSLLPETLPTSTDIEEESSGEQITKMNQMVLSSQMAISVLRTERPTQLPLQEKETFTKVDSLYSTSPLEPVSSKTTESYEKRLFSVTAADFESSGDDSDGLTGEPKITSPLASGVNTSDGDSTSDISKTSVTVASPFFSMEKATPTVSSALLEIEDIDDDGISNDTMLVQAIPSHTGSTIKLGILPYATTSDYESSGDSTYGFTKELARPTISSMFSTETPTMTASQESQNNQVSTVSVTQSSFHSTNKPTSVSPEIQKYVTTAKTDGVLFTMRTKPFPDHLEGSGYQTQDIFAKTSSVSNTEMPELEATSAVSTANDKSNMNVAIEETITIMSSVSSLSPEKQSPESELLTSSGQVKGFTEATPTSNLHGQSTLPGITTTTAAQSINTQTKIQPSTSAIPIINEFDAGSAIIIPEQDSSNGQTTEILTEESSSLPFLSMTDNTVKHSSHSAVVIGTSQEMSTTSPASLKPSIQIIDDTELIDQNPPTKVTGKTDENISSMFSTKTPTLSTASYSMEGNGKDYSHYSTDTPISPITQKNTDKSIVTVMLPTADRTLTVEPTSHPVSSTSLYSPVKAAMTTISLLSTTGSDAEVINIHEGSGDLTSALTGFTLANTESPAASKDSISTDMNSTSRKTLAPTTRATFPTIDAGSDDVPSDPVSTMFSTNKPFSTFAGLNPSTEAHTEEAEIHVLSESVNPTTVSKPQVTSVSSLFSTKKPTSMTPIEARSTSSLPATTEQSRNAESQNVGSLGDVTSAVTIDLSPVTPKEESSVEQVILSKTTITPLVTGTEESLGDSLFVISSNPRIIEGMQPMEVSSHSLFPSKESSAYGDMESSGLPPEEFDLETSSDGSGGDVAIGKTDETELYKSTDDTFTQPSRQPTQEFILSTQSPDKTSDEIYITEQGSGVFDEDLTPEDTVLTSSPPAITATSGTDETSPIPSKMSIHSDTVKQHMPMFTPTSQISDQESSMYDSDEGSGGENVFSTKTTATRDSSSGWTASDVVTLSASSLFSTKKSEIISGADNGRKTTSIWPSFYSTVKPMHSLPPDVPGTSSNERLTVSTVKPKTISHLDVLPEPGSGEAEILLSTDASSLYSTEKATATSFMDLSKTQSHEHIHVTEHPTAATPKTLLTTSLYPIATEGFADEISDDTTASPIIVAFDNRNTSTLSTLLSTDKAIRSQEQETNNVTIMQHEAGTVSHSSDRITFSPSQDESTVEHVTSSFTKDSFSTTTMFPIDWSSQYLDPSGTHAEVEYNTFEPSPTNEIISLDTTSQSSSSAKPEEEITWMKFESSPESSSEHSPRGITESASVQPESTTSSSTEQVSSVETVTSTTAAPDTAGESTSDSKEASVSTETSFITSTEEDGSGVTIPSIYNADLGSSTSPQTESSFYEETSGSTSTNTSMTDDYSTLPPGMSQISTESVSTDVENKETQFIDITTHTGESTTSYQVVATTKPEEHISEVPFGSLPSKGSILVQFVTTFAPPKHPTPSRESLEQAQSQIAITNRPQTDFSSEDVSPTSLTSAKQFDELTTIAPSVAEPASATVALATIDLTEELVSSGEMTESPPETDVRVTQSPPDSVYEDLTDETPDYEELNPNIVESIPEDTESIKSTETTMYTTSQAADSQPSDSLNSIGRETVTSALSGNLITTTYPGSNDIQTVFKVDATTTASNVESTTVHSVGIEEGADKTERSTVKPQVQEFTKTTPAETQSQNASVDPTQPLSGKDHKLDCDITPPPLLTDGETHYPACDLGHIILGETVDIYVIESCLDNICLNGGSCFMSGSIQICNCAPGYDGDHCETDTETCAYGWHKFHGQCYKYFPSRRNWDTAERDCRMQGGHLTSILSHEEQFFVNRLGQDYQWIGLNDKMFDSDFRWTDGSPMHYENWRPSQPDSFFSSGEDCVVMIWHENGQWNDVPCNYHLTFTCKKGTVACSQPPLVENARTFGRQRERYEINSLVRYQCGMGYIQRHVPTIRCRGNGHWDIPKITCMNPSNYQRSFFRKHHHTRVYSITHFRQWPDQQAFRLHHQRYRGRRDKPEHKRKRM

>CDK-like3

MEKMKRIKKRLSLTLRSSQTIDESLSELAEQMTIEDGGTKDNEPFMRNGRPPTSHSMHSFLHQYTGSFKKPPLRRPHSVIGGTLGSFMSIPRNGSRLDIVHENLKMGSDGESDQASGTSSDEVQSPTGVCLRNRVHRRISMEDLNKRLSLPADIRIPDGYLEKLQLTSPPFDQPLSRRSRRASLSEIGFGKLETYIKLDKLGEGTYATVFKGRSKLTDNLVALKEIRLEHEEGAPCTAIREVSLLKDLKHANIVTLHDIVHTDKSLTLVFEYLDKDLKQYMDDCGNIMSMHNVKVFLFQILRGLSYCHKRKVLHRDLKPQNLLINEKGELKLADFGLARAKSVPTKTYSNEVVTLWYRPPDVLLGSSEYSTQIDMWGVGCIFYEMAAGRPLFPGSTVEDELHLIFRLLGTPTEENWPGISSIEEFKSYNFPKYKPQPFINHAPRLDGDGIELLLAFLRYESKKRISAEDAMKHSYFRHLGMRVHTLHESVSIFTLKEVQLQRDPGYRNSSYPEPGNGKINRRQSMLF

>Lectin11

MVCLSTLSVENVEYIYIIGLLIAGWTLLDCNCYVYQEERRTFADAEAVCNILGGNLVSIHSDLENAVVQQLIVAGANDDTLAWIGLHDAILNGDYIWTDGTVEDFRNFGAGEPNAANGDCVAMDETGWTLLDCNCYVYQEEQRTFADAEAVCNILGGNLVSIHSDLENAVVQQLIVAGTNDDSFAWIGLHDAISNGDYIWTDGTVEDFLNFASGQPIALNGDCVSMDETDNECPKGWTQLDCHCYIFEDEARTFADAEAVCNILSGNLVSIHNDLENALVLELIRAGGDDNQGWIGLHDAIEEDDYIWTDGSDENFLNFDTTNSQPDGNVGDCVVIAENVGLDAAGKTTILYKLKLGEIVTTIPTIGFNVETVEYRNISFTVWDVGGQDKIRPLWRHYFQNTQGLIFVVDSNDRERVGEAADELSKMLGEGDLKDAVLLVFANKQDLPNALSIGELTEKLGLNKLRNKEARENLVKAIPPKLLCLLACGGVDCRYEGPECWKTNQQVIRGIFSSWVTDDIIAMARPSKILIDKYNIIEQFQRLNIRSIINMQMPGEHAHCGPPLDAKSGFTYSPQTFMENDIYFYNFGMPDFCASSLVNIIDGVKVLAFAVGEGRVAVHCHAGLGRTGVLIACYLIYTLRITPSEAVHYVRIKRPRSIQTRAQISQVFDFARLLSTQLMQYPDLSLRHGSPVTLQHYLNRQALLLHGQEARTLRHIPKVVYLLCVHLSCLALGLPAAPEVNAELEKRSALRILTRVVRETLVSKQFLPLLREHLNIKRVGSGSVSSWDEQLGFMERKRDLLLDKRSYSDSDLCKMVDLEMSPWCAWDVTEPQLRPLRSKTAPLSPQMITKNHSHARFMQPSNNSARNFKCTVKKAQSKYSSNLELSRSTQNVRPTSVARAIAKAMADQGPPEETILRRSSLLQEELNSSECGWALLMTETDARVLTCLLWTWLEKLKSQRHTIYCLLRCVNTVTSLCPHREDAVLKRLLRALTKIMPDEEPWVGSWRPHRPRGPIAALYSSPGPKYALPSLTGSRLHDPTKHKAPSFSFRAHYDISTQETSPGPKHLIPSNITKNGRDGTPAFSFGSRPKDAVRAQVPAPNHYHLESSDKVTYHTSPSYTLSGRWKQVAQSNQTTPGPASNTLPPVFGPKTVNIRAAPAHSLYGRSKNGKFMEDYTKSPGPAAYQAVDPQIYLNKSPQYSMPGRKFVPQSATRTPAPGVYCPEKVGDILLPNGAK

>Lectin25

MAFALRLLFLLCGISGLLTGVDTDYIWTDGTVNDFENFDGTPNDNNDCVQLLQTDNNCPKGWTRLDCFCYIFQRDPRNFADAESVCNILEGNLVSIHSLLENAFVRELAAAGGNMNQVWIGLTDALMDTDYIWTDGTVNDFDNFVGTPAASINDCVQLLQTDGTWQTAACIDTEQFVCIRDVMY

>Lectin21

MGLNGTLTAYQVSLLVQLSLADLVAALILMFTSAMNKVRTDDSVTICQYSLPLSLTFYLISFLLVAVYAWKSKNSIKGWRATPAEDEGRRSHFGSRLFSLPVYMYVWLFPAAVYLAYVLTPFIKSTPLIPDTRSVTTQDHAKYCTSCILFLHIWCDSCSDAWSLEKIQFKDNNCPKGWTRLDCHCYIYQDDPRDFADAESICNVLDANLVSIHNDLENAFVLELIRAGGNDDVTWIGFSDAIVEGDFIWIDGSEQDFLNFDGTQVPAEPDDSGDCVVLSEGDGLWLDQPCTDEEPYVCIKEVSH

>Throm53

MKFLILLALLGAALVAAEQDDKIVGGYECPRHSVPYQVSLNAGYHFCGGSLISSQWVVSAAHCYKSRIQVRLGEHNIAANEGTEQWIDSAVLVKHPQYNSHNLDNDIMLIKLSRPAALNNYVRTVPLPSRCTFADENCLVSGWGNMAANGNNFPDRLQCLRQPIIDDRICQNAYPHLFTQNMLCSGFMHGGASSCQGDSGGPLVCNGELQGVVSWGYDCAMKGHPSVYARVCRYNSWINSVMRSH

>CDK-like14

MEQSAPKSKLKKLSEDSLTKQPEEVFDVLEKLGEGSYGSVFKAIHKESGQVVAIKQVPVESDLQEIIKEISIMQQCDSPYVVKYYGSYFKNTDLWIVMEYCGAGSVSDIIRLRNKTLTEDEIATILKSTLKGLEYLHFMRKIHRDIKAGNILLNTEGHAKLADFGVAGQLTKSKVMAFTQKDTMAKRNTVIGTPFWMAPEVIQEIGYNCVADIWSLGITSIEMAEGKPPYADIHPMRAIFMIPTNPPPTFRKPELWSDDFTDYVKKCLVKNPEQRATATQLLQPGQAGLHPEGPXYFKNTDLWIVMEYCGAGSVSDIIRLRNKTLTEDEIATILKSTLKGLEYLHFMRKIHRDIKAGNILLNTEGHAKLADFGVAGQLTDTMAKRNTVIGTPFWMAPEVIQEIGYNCVADIWSLGITSIEMAEGKPPYADIHPMRAIFMIPTNPPPTFRKPELWSDDFTDYVKKCLVKNPEQRATATQLLQHPFISQAKPVSILRDLITEAMEMKAKRQQEQQRELEEDDDNSEEETEVDSRTMVKSGSEGAGTMRATGTMSDGAQTMIEHGNTMLESDLGTMVINSDDEEEEEEPGSMRRHATPQQPMRPSFMDYFDKQDSNKAAQHHHQHQRQQENYNHNQPQEQPGYHIQSKNIFPDNWKVPQDGDFDFLKNLDFEELQMRLSALDPMMEREIEELRQRYTAKRQPILDAMDAKKRRQQNF

>BPTI9

MRDFVQSVVETLSLDDDKDRVSLVQYSTDAAVQFYLNTYTTKGEILDIVKGLRHRGGRTLNTGAALQYLRDNVFTASAGSRRLEGVPQILILLSGARSSDSVEAPASALKQLGVLIFAIGTRSSDSGELQKIAHDPRYALSVTEFTDLPRVQQQLQSSVEAVVIDVTPETPPVIAEAKKDIVFLLDGSDGTRDGFPAMRNFVEQVLEKLNVGANKDHVSLVQYSTDPEANFYLNTYTTKEDVVNAVRGLRHRGGSPLNTGAALRYLRDNVFTNSAGSRRTQGVPQILILLNGGRSFDSVDAPVSALKQHGVFTISIGTQNSDSSELKKISHDPRHALVVSELSDLPSVQEQLSSVISRVLRATPLTPTVTADRPGRDVVFLLDGSNATRNTFTAMRDFVQRVVETLSLDDDKDRVSLVQYSTDAAVQFYLNTYTTKGEILDIVKGLRHRGGRTLNTGAALQYLRDNVFTASAGSRRLEGVPQILILLSGARSSDSVEAPASALKQLGVLIFAIGTRSSDSGELQKIAHDPRYALSVTEFTDLPRVQQQLQSSVEAVVIDVTPETPPVIAEAKKDIVFLLDGSDGTRDGFPAMRNFVEQVLEKLNVGANKDHVSLVQYSTDPEANFYLNTYTTKEDVVNAVRGLRHRGGRPLNTGAALRYLRDNVFTNSAGSRRTQGVPQILILLNGGRSFDSVDAPVSALKQHGVFTISIGTQNSDSSELKKISHDPRHALVVSELSDLPSVQEQLSSVISRVLRATPLTPTVTADRPGRDVVFLLDGSNATRNTFTAMRDFVQRVVETLSLDDDKDRVSLVQYSTDAAVQFYLNTYTTKGEILDIVKGLRHRGGRTLNTGAALQYLRDNVFTASAGSRRLEGVPQILILLSGARSSDSVEAPASALKQLGVLIFAIGTRSSDSGELQKIAHDPRYALSVTEFTDLPRVQQQLQSSVEAVVIDVTPETPPVIAEAKKDIVFLLDGSDGTRDGFPAMRNFVEQVLEKLNVGANKDHVSLVQYSTDPEANFYLNTYTTKEDVVNAVRGLRHRGGSPLNTGAALRYLRDNVFTNSAGSRRTQGVPQILILLNGGRSFDSVDAPVSALKQHGVFTISIGTQNSDSSELKKISHDPRHALVVSELSDLPSVQEQLSSVISRVLRATPLTPTVTADRPGRDVVFLLDGSDATRNTFTAMRDFVQRVVETLSLDDDKDRVSLVQYSTDAAVQFYLNTYTTKGEILDIVKGLRHRGGRTLNTGAALQYLRDNVFTASAGSRRLEGVPQILILLSGARSSDSVEAPASALKQLGVLIFAIGTRSSDSRELQKIAHDPRYALSVTEFTDLPRVQQQLQSSVEAVVIDVTPETPPVIAEAKKDIVFLLDGSDGTRDGFPAMRNFVEQVLEKLNVGANKDHVSLVQYSTDPEANFYLNTYTTKEDVVNAVRGLRHRGGRPLNTGAALRYLRDNVFTNSAGSRRTQGVPQILILLNGGRSFDSVDAPASALKQHGVFTISIGTQNSDSSELKKISHDPRHALVVSELSDLPSVQEQLSSVISRVLRATPLTPTVTADRPGRDVVFLLDGSNATRNTFTAMRDFVQRVVETLSLDDDKDRVSLVQYSTDAAVQFYLNTYTTKGEILDIVKGLRHRGGRTLNTGAALQYLRDNVFTASAGSRRLEGVPQILILLSGARSSDSVEAPASALKQLGVLIFAIGTRSSDSGELQKIAHDPRYALSVTEFTDLPRVQQQLQSSVEAVVIDVTPETPPVIAEAKKDIVFLLDGSDGTRDGFPAMRNFVEQVLEKLNVGANKDHVSLVQYSTDPEANFYLNTYTTKEDVVNAVRGLRHRGGSPLNTGAALRYLRDNVFTNSAGSRRTQGVPQILILLNGGRSFDSVDAPASALKQHGVFTISIGTQNSDSSELKKISHDPRHALVVSELSDLPSVQEQLSSVISRVLRATPLTPTVTADRPGRDVVFLLDGSNATRNTFTAMRDFVQSVVETLSLDDDKDRVSLVQYSTDAAVQFYLNTYTTKGEILDIVKGLRHRGGRTLNTGAALQYLRDNVFTASAGSRRLEGVPQILILLSGARSSDSVEAPASALKQLGVLIFAIGTRSSDSRELQKIAHDPRYALSVTEFTDLPRVQQQLQSSVEAVVIDVTPETPPVIAEAKKDIVFLLDGSDGTRDGFPAMRNFVEQVLEKLNVGANKDHVSLVQYSTDPEANFYLNTYTTKEDVVNAVRGLRHRGGRPLNTGAALRYLRDNVFTNSAGSRRTQGVPQILILLNGGRSFDSIDAPASALKQHGVFTISIGTQNSDSSELKKISHDPRHALVVSELSDLPSVQEQLSSVISRVLRATPMTPTVTADRPGRDVVFLLDGSNATRNTFTAMRDFVQSVVETLSLDDDKDRVSLVQYSTDAAVQFYLNTYTTKGEILDIVKGLRHRGGRTLNTGAALQYLRDNVFTASAGSRRLEGVPQILILLSGAKSSDRVEAPASALKQLGVLIFAIGTRSSDSRELQKIAHDPRYALSVTEFTDLPRVQQQLQSSVEAVVIDVTPETPPVIAEAKKDIVFLLDGSDGTRDGFPAMRNFVEQVLEKLNVGANKDHVSLVQYSTDPEANFYLNTYTTKEDVVNAVRGLRHRGGRPLNTGAALRYLRDNVFTNSAGSRRTQGVPQILILLNGGRSFDSVDAPASALKQHGVFTISIGTQNSDSSELKKISHDPRHALVVSELSDLPSVQEQLSSVISRVLRATPLTPTVTADRPGRDVVFLLDGSDATRNTFTAMRDFVQRVVETLSLDDDKDRVSLVQYSTDAAVQFYLNTYTTKGEILDIVRVLRHRGGRTLNTGAALQYLRDNVFTTSAGSRRLEGVPQVLILLSGARSYDSVEAPASALKQLGVMIFAIGTRNSDSRDLQKIAHDPSSVFSVTEFTELPSVMSRVLSALSMKPTLTAESQGPRRDIVFVIDGSDGIGRDFPIIQEFIRKVVENLNVGENKIRIGVLQFADYPQADMYLNTHPTKEDVLNAVRGLRQRGGRGRNLGQALQFVNEDMLTAARGSRKQEGVPQFLVVVSSGSPTDDINRPASTLKRSRVIPFSIGTRDMKPKDLQIVSFVPNFAYTVDDLPSLDTVQENVINRLTELSDDDIARIIPQFPVIEEIVPSTGGEKRDVVFLIDGSSAARNDFPSIREMIKRVVERLDVGLDKVRISVVQYSDDAKVEFLLNEFSTKNEVRQAVTQLRSRGGSRLNTGRALEWVSRNIYQRSAGSRIEDGVPQFLIVVTGGSSTDDVTTPADQLKRSHVAPIAIGSRNSDPDELRKISLKPELAYTVETFQDLPAVEQQLIDSVKTISATDIIESYRPPDEPLEVLEVGRKDIIFLIDGSDNTGVTGIAHIRDFILNLIRQLDVQPDQVRVALVQYADRVKPEFSLNSYNAKPDVITAVRKLRQMGGRSSNLADAIEYVIQNELKPSAGVRPLEASQHLVVLTGGRSPQDVSLYGPLLKGSRVNCIGIGASGADTRQLAQIAKSPEDVLQVPTFPGLPAINDRLVARLKGRIIDDTTPEEIPTQGLPAAKKADIVFLVDGSINLGRENFNKVMAFVAELIDLFYTDRDNLRIGLAQYGTDVSDVFYLNTYKNKPDIISAIERADYPGGRRTNTGAAIRHIQNVQFSKEKGSRKDEGTPQILMVITGGPSSDDSKSAILNLKNSGVRVFAVGVGDADSELETIASDSSTVARARTIQELSELNEQILGTLDDEVKGKLCVGVQEAPKACNLEVLVGFDVSAQNIFSAQTNLLNKMAAILQRIAKMSTVSCSSGQIPSIQIGMLAIDSTGQPVQVDFSDNADKLLDAFRALRARGPFVLSGKTISAYTDRFRNRQDDTVKVVIHLTDGIDAPYSEMKRRVDELRQSGVNSLILVGLERVPRFEDAALLEFGRGFRYTRPLRLNILDLDYELMEELDNIAERECCGVPCKCTGTRGDRGSIGVSGPKVIIGQKLRSKYPKKVVQVVQEAKDILVMKVDRVLKASKAKQETEEKWESEETLAQVERITPNLDPRETPVMPDQRVRLDPTVRRDPEEKTEILDPGVLGEARALREKREEGEPSVARESPEIQGLKASSDPSDSAESLAKMAEMVSASRGPKEEGQGDEGFPGFPGPKGEAGNAGTKGGPGLQGNRGQRSRSHSFYGDIYKLGSISRQGVSGNAGTLGQKGEVGYPGPYGQKGPRGPGVVQCDLVKKIRDNCPCCYGKLECPLYPTELAFVLDASSDVGRPAFNSMREATLRLLRGLTVSESNCPRGARVALALYNSEVTTEVRFADALKKRALLRHVEGLQTLQTNKRRSLETAMSFVAHNTFKRVRSGFLVRKVAVFFVGGSVANAQAVTNAAMRLHDSGVATLFLVKSDDRALARALQVNNTALAQVIVLPNPGSAQFDSVMTKVMNCHLCFDICAPDQMCDYIPPSAGRDRRSFTTDVDIDMGFVVDSSESTYPVVFTEIKRYIAHMVEHLQVSSSPETSAHHARVAVLQQAPYQFLHNQTKSPVRVDIGLTEHQSAQGIVNFLLDKTPQLEGGRSLAAAMEWTVEQVFEKAPLQRPRKVLTLFVTGGVEEDEERLVRVATDIKCRGYFVVIFGVGENLSAADARVLARMASEPSDVFFKRLDAISQFYDKHLLTFGQMLPKYISIENAFFMSPEVSKHCKWFQSDQPLKNPFTTLARQPHPETSAHHARVAVLQQAPYQFLHNQTKSPVRVDIGLTEHQSAQGIVNFLLDKTPQLEGGRSLAAAMEWTVEQVFEKAPLQRPRKVLTLFVTGGVEEDEERLVRVATDIKCRGYFVVIFGVGENLSAADARVLARMASEPSDVFFKRLDAISQFYDKHLLTFGQMLPKYISIENAFFMSPEVSKHCKWFQSDQPLKNPFTTLARQPQSQTKHHENQQETHPVKHKAVDAEELHVSNVTRSGFKLRWSNPDPKNFVYFQVTVTRLRDHALAVKTNVSASELSVDKLESGQTYHAVVTGHGVDGHVLATHKGVVTTKAAAQRPLINSRVITAAVNRPLDKPDTVTVPEPEPKQQQVDTAELHPAPAASTAVDICQLPKEEGTCAKFVLKWHYDAPSKTCARFWYGGCGGNQNRFDTHEQCAKACGKPAPIKQGVMASLST

>Amyloid3

MHHPRVPAIILLLFVLLRCITAAPSNRHSSSSSFGEEESALLPDGRESSVPMFPFSNPFLGLMTTREVPDENSHLIQKRKCNTATCVTQRLADFLVRSSNTIGTVYAPTNVGSTTYGKRELLLPPNYYFPL

>BPTI17

MSQEQEGISLPVPCLGACAARGADVGGGGAAGASGVGLPAAGTFWQDSVIAGGGHGQTSANCAMDGGGLLSVGKSCKSRPVSVAYVLNNEPNQQNNAECMALRCLKDACEAVGSKLETVHFGKLDFGETTVLDIFYNADIAVVEMTDAFRQPSLFYHLGVRESFSMANNIILYCDTNSESVQSLQTCSANYTFIPYMVTPHNKVYCCEGSLMKGLTELMQPNFEMLLGPICMPLLDRFIQLLKVSQASSHHYFRETILSEIRKARELYTGEELTAELGRIQQRLDNVECLSVDIVINLLLTYRDIQDYESVVKLVETLGKLPTFDLVAHPHVKFHYAFALNRRNQPGDRQKALDIMLPLVEAKEQVASDIYCLIGRIYKDMFLESHFADTQSRDSGTHWFKMGFESEPTLHSGINYAVLLLAAGHQFETSFELRKVGVKLSSLLGKKGSLDKLQSYWDVGFFLGASILACDNTRVIQASEKLFKLKAPIWYLRSLVETILIYQQFKKPCVEQPAPKLEVMDFWMDFLVEATKKDVSSVRFPVLILEPTKVYQPSYLSINKDVDDNTVSIWHVTHDDKGIHEWNFSATSVRGVSISKFDERSAFLYVLHNAEDFQIYFCTEMHCKRFCDLVNSITEEAWRGPDEGDCDTDALEYDYEYDEHGERVVLGKGTFGVVYAGRDLSNQVRLAIKEIPERDSRYSQPLHEEIALHKHLKHKNIVQYLGSISENGFIKIFMEQVPGGSLSALLRSKWGPLKNNEPTIGFYTRQILEGLKYLHDNQIAHRDIKGDNVLINTYSGVLKISDFGTSKRLAGINPCTETFTVYHFCIWYVLHNKGIHEWNFSATSVRGVSISKFDERSAFLYVLHNAEDFQIYFCTEMHCKRRNQPGDRQKALDIMLPLVEAKEQVASDIYCLIGRIYKDMFLESHFADTQSRDSGTHWFKMGFESEPTLHSGINYAVLLLAAGHQFETSFELRKVGVKLSSLLGKKGSLDKLQSYWDVGFFLGASILACDNTRVIQASEKLFKLKAPIWYLRSLVETILIYQQFKKPCVEQPAPKLEVMDFWMDFLVEATKKDVSSVRFPVLILEPTKVYQPSYLSINKDVDDNTVSIWHVTHDDKGIHEWNFSATSVRGVSISKFDERSAFLYVLHNAEDFQIYFCTEMHCKRFCDLVNSITEEAWRGPDEGDCDTDALEYDYEYDEHGERVVLGKGTFGVVYAGRDLSNQVRLAIKEIPERDSRYSQPLHEEIALHKHLKHKNIVQYLGSISENGFIKIFMEQVPGGSLSALLRSKWGPLKNNEPTIGFYTRQILEGLKYLHDNQIAHRDIKGDNVLINTYSGVLKISDFGTSKRLAGINPCTETFTGTLQYMAPEIIDKGPRGYGKPADIWSLGCTIIEMATGKPPFYELGEPQAAMFKVGMFKIHPEIPESMSPEAKAFIVRCFEPDPDRRATAFDLLTDEFLTVTSRKKRSKSSFTALSPGTEYLRSISLPVPVVVEDTSSSSEYGSVSPDNDLHTNPFIFKPSVKCYSERDVKGPRSLFLSIPVENFEDHSAPPSPEEKDSGFFMLKKDSERRATLHHILTEDLDKVVANLTEALTQGSEEMKMKPQHISTLVVSLADFVRVADRKIIARTLSQLKLELDFDSTAISQLQVVLFGFQDAVNKVLRNHNIKPHWMFALDNIIRKAVQTAITILVPELRPHFSLASESDAPDLEDADVEDTERNSPQQRRPPASSSQDETVVTSGVSTLSSTVSHESRNGQHSVSMELGRMKLETNRLLEQLLDKEREYQAILQQVLEEREQEIRLLRLRSHTTANISTSTCGPQARGAQPAQKREETELSGWLRLYGADQDSIDVILSEEYTLNDILHDVTREDLKSLRLSIPVENFEDHSAPPSPEEKDSGFFMLKKDSERRATLHHILTEDLDKVVANLTEALTQGSEEMKMKPQHISTLVVSLADFVRVADRKIIARTLSQLKLELDFDSTAISQLQVVLFGFQDAVNKVLRNHNIKPHWMFALDNIIRKAVQTAITILVPGNHIAVNKVLRNHNIKPHWMFALDNIIRKAVQTAITILVPELRPHFSLASESDAPDLEDADVEDTERNSPQQRRPPASSSQDETVVTSGVSTLSSTVSHESRNGQHSVSMELGRMKLETNRLLEQLLDKEREYQAILQQVLEEREQEIRLLRLRSHTTANISTSTCGPQARGAQPAQKREETELSGWLRLYGADQDSIDVILSEEYTLNDILHDVTREDLKSLRLSQADSSSSAVSILTAAGTQGAVLGKSTNMAAMQHHRSPRIAVVAPLQLLLVLCALQPIHTRMTAISDLKSKITGVEELLEEFRKQLQQDQTYRPSEVSDSCVSDFSSTRESIIRAKASIEQGATFLMAPDRVYTWRDCVHACCAQPHCTVAVVQVEPRQAGDSLNCYLFNCTYRSQNVCSFAPQPGFASHSRPLNVTLGRVLQDSPGGSENERRPGEADDTQEIDEPPRCDAGQDVVIQLPTDWAVLDGRDSVDDHGISHYDWTLVKGDAAITMKASHPGLLKITGLQEGVYTFQMTVTDTSVCTGRCSNYQFKCDDGCCIDITFACDGKQHCPDRSDEDFCPNFDTSRKSVTHAEGLPIHPLPVAHTVESLDSFVLQTGTKKTNDNSRPSQERSRVTPPPSQKQDSNSQDPCSTAPIVGPCKGTFARWYYDREAGECKHFLYGGCQGNHNNFLQESDCVSECIQKSPGLKPATVAPPITKQTEIAAPGVSQIQEGEIPVYKRFPVKGGHPVAESGAVLPLALGLIITALLLLLIAFRLRLVRHKLKKARPLTTEESDYLINGMYL

>Antipro3

MHGFFASCTVAALLLATCLAIPHHHQGDQADCHMLSSPNADFGFALYKNLRAKAGAAENIFFSPLGISTALSLMSTGAHGETHSQMFSTLGYSGQEQATVNGAYKHLFEMLAHHDQEHHQQLDVSNAVAIDDGFSPSDAFMKSAKEFYSAEVLKVDFGKSQEAAAEINSYIANKTHNMIKEHVKDLNEDMAMMLINTVYFKGEWFRPFNKSLTGKADFHVDETTKVEVDMMKRTGRYDMYYDNDNGTIILLLPYKSNTSMMIVLPDEGKMKDVEGFISKDYIKHWHNSVYRQSVDVFLPKFSISAKAALDSTLKEMGITAAFGDTADFSGISDDVKLKLSKASHEAVLNVDETGTEAAAVTTLEVMPMSMPMRIKMDRPFLVLIVEDSTQSVLFMGKIANPTAK

>Antipro4

MHGFFASCTVAALLLATCLAIPHHHQGDQADCHMLSSPNADFGFALYKNLRAKAGAAENIFFSPLGISTALSLMSTGAHGETHSQMFSTLGYSGQEQATVNGAYKHLFEMLAHHDQEHHQQLDVSNAVAIDDGFSPSDAFMKSAKEFYSAEVLKVDFGKSQEAAAEINSYIANKTHNMIKEHVKDLNEDMAMMLINTVYFKGEWFRPFNKSLTGKADFHVDETTKVEVDMMKRTGRYDMYYDNDNGTIILLLPYKSNTSMMIVLPDEGKMKDVEGFISKDYIKHWHNSVYRQSVDVFLPKFSISAKAALDSTLKEMGITAAFGDTADFSGISDDVKLKLSKASHEAVLNVDETGTEAAAVTTLEVMPMSMPMRIKMDRPFLVLIVEDSTQSVLFMGKIANPTAK

>Throm25

MSSFLDPFFEILDGALDDEDDDDDDPSEWLYELQEPAGRCNPNPCQNNGVCKEKGNQKFKCDCPKHFKGRRCERDTLCEPNPCKNGGRCIKDGDDFDCECPSGFRGRFCHVGPNDCYDDDGESYRGTVSETVNGNECLYWNSHFILKNGIDPFNSFEDSDGLGPHNFCRNPDGDLTPWCFFRRGKKLLWNHCDVNECSEEGMTPPAPPDSPTPPQPPAPKPQPTGSSPDQKPTQPPQPSDTSPTLAASPKLFNTCGKPQPKKVVTRIFGGLKVSPGALPWQVSLQVRPKRSTQKFKHICGGVLIESCWILTAGHCIERGSDMQVVMGRLSLDTDEPTEQIINVEEAIVHENYRETPVSVHNDIALLRLESTDGVCANETQFVKAACLPEEPLDDGEECTISGWGATEESDYGSNHLLEANVLLINQAKCSDSNIYGTVLDNTMFCAGHLKGGIDSCQGDSGGPLTCEQNNTHVVYGLVSWGDQCGQKNKPGVYARVSHFLDWIKSKTQASS

>Throm24

MRIQGQPEGARRHLDLSTPGELRRVEVYRGRSGYGFTISGQWPCLLSSILEGSPADLVGLKQGNGIMAINGADVSTALHETVVQMIGRCIGPLRLVVHTEGRVIGNPILHDEKFLISPKKAGVLRAISHDSPNSDNWHKTLAGKQRPLSEPDMSQWSKQRSAEIQAPKDEHRSVLTDSEEANSDWSILNMNLVVGYLSSTELILNTSQSEDDGLKAIRDRIRQLGTEQDTHTLVVMKITFDCIRLCDDMGAILAAFPTENLVLGAVCTEDPRFFCLVTTAHIINGCASQDAPLRASCHVFFIDPELGNHEEHTGIAGRFGFDCTPDPDTSGCLEFPQTPPDVLHFVTVLYSNMGDAVERLRVKLDIEAQQQAKENVQHGSSSSNGDSGIDNASPPEERAARDFPSVIQNHPGVLLPTCPWDNPGSPDHIPRNLYNNGQRYHPENSGSTHSLSESLPGPDSLSDFYGGPPPRLKFQFKPPPPLAPLEKKNSLSEEPLRGSQRWFSKPKWPKSLSGDPEPQMTWNNDNWLAGVGLGAGGARNLPPAMSQIPSERYRSAEFLLMMPPKEEWTQAFVHRKNERGKNGSRFWGLGVLRASKRRSSKHLNLARSLDDLESAASSDGDSSGVQLQGCCSHSSLNSNGSLPGGHRGLPEQRVASWAACFERLLQDPVGVRYFSEFLKKEFSEENILFWQACEYFSHVPASDKKQLSQRAGEIYNSFLSSKATMPVNIDSQAQLADDVLTSPRPDMFKTQQLQIFNLMKFDSYSRFLKSSLYKECMRAEVGGLPLPDPYQIPCSPAPSKHSASSDRSTLSTPKKDARKQRSGRSLNEDSRDESADKKRGIFFSWSRNRSFGKGPKKKDIGDINLDYWGSNGRRESQGSLSSSTSLEMPTSCSAGKIESDNRHSMGAWERSPKHCSVTLPDGSCSSITLRPGASIREVLQVLCHSINVNMAAVDLFLVGGEKPLVLDQDCMTLSSRDLRLEKRTLFRLDLVPINRSVGLKAKPTKPVAEVLRPVVSKYGLLLTELVVKISGQTEPLDLGAPISSLDGLRVVLERIDSASAKDTAKSASLKSQPPTRSLSGTEDERSAAPKEFGGRAGGPDLALPGEKRKQKKINIDEAEEFFELLSRAQSTRANDQRGLLTKEDLVLPDFLRLVPLPTCPSSDQVCSTPDSLKQNRENGGHVRGPLASGLRSESLDSSLVHSGARRCLMPPSRHSPFGSHLSPIPRPPDARSSLHTVEEDAHADLTLVGEGDINSPNSTLLPASPSPMPSLEGSLPEANFTPPPLPCPHAQDTNAKSTPGGTKAPGEKEALEQEVMDVEGVHLEERAPPDTPHGDESELSLSFQGYVAELRQCQSRMRNAQQMPHGAVHLDEPDCKADLFKATVHIDAYFHHDIRALTFSSICSQHTRVVTTFGKECAIPFRQGGRIHHHCITVIASRPWCSLTSNFDRDRKWGFCAPERRHPNEQCYETVHLRHYDAGESWGRIHLRNVEQCTCLGGKIECQRARYTRCRSNPCQNDGTCRLITATGKEVCNCRYGYSGPHCSFKPESECYNSRGISYRGVVSTAASGARCLPWNSDLLYNELHVGTVAAAALSGLGEHAFCRNPDGDKMPWCYTLSKGAISWEYCAVPSCKMPVSSSRRMNTNTLPELGDSDHSVADKKPVCGTKHKKRVPVARGRIMGGNLALPGSHPWMAAIYIGQSDFCAGTLIASCWVISAAHCFFRNPLMSQLRVVLGQHHFNVSGPDTRTFGVDKYIFPKQFSVFNPTLHDIVLVKLKKQDGRCVKRTPFIRPICLPENGVAFPDNYCCSISGWGHMHENSYSSLQEAGVRLIRHDTCRRPEVYGNHVTADMICAGLNGCVDACQGDSGGPLACAKDDVNFLYGIISWGEGCGRSGKPGVYTKVANYMDWINSVIRRKAKKT

>Ubiq13

MEEPSADIEVTVKTLDSQSRTYTVGGQLTIKEFKEHIASSVGIPIDKQRLIYQGRVLQDERTLADYNVGGKVIHLVERAPPPPSQPGSGGTSADGGGGSSSSQDGPQAPPHDRNANSYVMLGTFNLPVNIMDPQQIQMSVQQMVSGMGDNARNARVTTSTGSNGSVNVHVDMDQPVHSEPRLRLVLAENMLRDVNDLIERMEGRADDPSVHTQRETPSTAAPPPPSSSSTTSTPSPSAQPMDTSPPPVTPPPPPPPSSAPPEGPTPQPGPQHPSPTELAEMLSELRRVEERLQPFIQRAHTILETATSAEYNNNTEREDDQQTLVLTWECLRLLGNALVALSDLRLNLLSPVPRHLHVVRPISHYTVPVTMPGAVHHHIPVQMNLGATMSAPNGTEGQAPPAPPAGQSEQAGQGQTSPPQTSPSNQQAGQGQGQGQGQAGPRVIRISHQAGPVVMMQMNTDGGLPPDFMRNLMQQITQYATAVATSAATAAATGQPVPPQPTPPGYNSTANSSATTTGPNTPTTTPTAPPPPPHAGPHPQARVVFTRPPFAPNISLPAFGTRGTTINVRAAMPGQQPGQAFNAAAVNQMISAVVAGQTANASTPTTTAPTSSSSTSSSASSHTSSSSFSVSSGDTNTAGPPNQSEGSGGEQAQPPEMHPDLGQMLGSLLGATGQAGAPSITVTTSSIPNLMRGVADLMQQPIFSQPPPPPPPSTGTNGAPNASTDPATNPAAAPTDALNPEPFIGIVRGVLTSMMGSLGQAQNDTESIAQFMQRLSQSTNIFISPEEPSGFFGELLMLVCQTFTMSDLVMLLHGHHQPLGRIQTQLAQFFHQNYLNGQEPTDANIAAAADAIVQELEEDITESFRESSVTVLEGVDVTQTNMSFFRQQLTHIATHILRCTDDSFGLRLLQLCNQALFECLALNLHCLRGDQRALTAVINHRIRRMSSDVSPSLLNWMTSMMTMRLQYVLEHLPITTEQIQNYIVYTQMSETLSPAAATTAEEAMSVSSSRETRAAPADLGAAGGAAPLGGDKAAAGAEGTSEVSAGESEAWAATVPPEWVPIIRCDMMTQRKMKAQPPLSDAYLHGMPAKRRKTGLGSGSLVSLSDAVSQAARTAGVKPITSGERLQEELDSPQLREAYSEQVKADIKKRVRDDTDFSAQQFPDTHRAFLSDS

>CDK-like9

MDLQHREDFSTRNPQDDFEILLKVGWGTYGDVYKARNKQTGDLAAIKIIKTEPEDDFSTIQQEILIVRSCKHANIVAYYGSYIRVNKLWICMEFCGGGSLQDAYSATGPLSEAQIAYVCREMLQGLDYLHVQRKMHRDIKGANILLTDHGEVKLADFGISAQITATLARRMSFIGTPYWMAPEVAAVEIKGGYNELCDIWSVGITAIELAELQPPMSSMFYNFVKAVLVKNPKKRPSASKMLSHMFLSQQSLSRALTLDLLEKCRHPENFCPATDDDEMEKELANYLRWYSAVSSLLAQIYFKKIFQGCPLKVKCTTTWKHPDTKEQHLVVGTEEGIYTIKLNSSEATMELLYPGKCTWVYSCHNVLMSVSGKSSQLHSHSLKELYEQARRDQRMVALPTHRLLTRRCAVTCKIADTKGCRTCSVADNFLCCALESSVVLLQWYEPMNKFMLIKHFDFPLTNALRVFETVLDPQQEYPLGLDKTCIHQEVSYTFYKDSNCVYIVTDQRQPDTVQVKQLDSNSLLVLLEGAAHIVNLEGELKSCQPHKTTFSHNVESVVYLEDTFLAVWRHGWLRTKKGKVLEENTDPKKIFRLVQSDRMVVMETHQTKDPSGLSNLYLLEIAENYVMLP

>Lectin22

MDSMELDDCGYLKKNLTMQGIVTEVNFQQKKKSSRCITVSLGLLCAVLLAGNIGQLIYYEILPRFSTEHQAKSDHLHNNIFLTDTNHRERNELEVTLNNLTRDKDQLQTNYESLVSERARLLDSEMALTAERDELRANFQRVKQQHDELQTNYSILNREIDVLHTLTRALGTDKDQLRTSYESLIDVKDQLQTSYNSLVKEQESLQANKTTVTAERDDFKSRLNNLEKEKDQLQRDYSSVRSEKDQLQTTYKSLVKEQDKLQTSYNSLVEEKELLLTSETNVAAERDDFKSRFDNLKNEKDQLQRDYTSWRKDKDLLQVSYDSLVREKDQLRASYESLIKVKDQLQTSYNSLVKEQESLQANSTTVTAERDDFKSRLNNLEKEKKQFQRNFSSLRSEKDQLQTTYESLVKEKDKLQTTYNSLVEEKELLLISKINVAAERDDFKSRFDKLKNEKDQLQHDSSSLRKDKDLLQVSFDSLVREKDQLQASKTTLSAERDQLRSHFDTLKNEKVQIQRDYSSVRSQKDQLQASYELLVKEKAQLQSSYNSLGREKQLLLTSKATLSTERDNCMSRFNNLKNEKNQLQQSYSSLGSEKAQLDARYATLVQEKQLLIANKAKVASERDVAKSLYDNIKKDRDYLQKNYTLLEEDKDRLERSYTLLSERSSMCQIRYKLLQGQKTDVDGDYNTLRVEYERLRRNHSSLETERYELLKTVDKMAIKVKSMLCGVGWKKFDGNCYFVSKLSRSWTESRLDCISRGADLVVINSREEQVFLNGLLGRGKDVWIGLRDHIEEGTWVWVDGTPLTTEFWDKNQPNNLENQDCVEIDTRPVGLGTWNDLGCSVQQPWICEK

>Throm57

MPCATSQADPTSPELAPGEECTVSGWGVASPYRVILSPELQAVDVNIMAFCNYYYWVNVTPNMLCAGSQIRGKDSCQGDSGGPLISNGFLEGIVYWGISCVNPYFPGVYTKVRLHALDQLDK

>saposin-like

MPRYELALILKAMQRPETAAALRRTVETLMVRGAVVRDMENLGERLLPYVMTKHNQRHRRGSYFLIDFYAAPSILTDVIDHLHRDVDVAPFKMGSAVLRECMEQIGATPLLGTEQCARGPSYWCQNVKTASLCGAVTHCQQNVWNKPQMRSVPCDLCKEVVMVVMQILKDNATQAEVLGYLEKACQLIPDKGLSAECKELVDSYYPIIVSIIIGELENPGVACGAIGLCLSQQMALAEFHEQLMSNEIPQIDLSKSATPFLLNVPGLLYPQKTPKPETPVKGSNDVCQDCIKFMTDAQSEEKDNPSFVNSLIVKLEENCDKMGPGLSDMCKQYISMYGSDVLQRIFSSDPKDVCSIAGFCSAGKNSTPMLKLQAAEIRPASKMVPALKLVPATKVESAKLTKVTSQPMVRVRDSPSCVLCEVVMEQLENILKNQSTEEEVVAALEKVCYYLPSSMTNECKDLISTYGKAIFELLVQQADPKTVCTILALCNNARPAYIPALDKTTFKNGAYCKVCKMAVTYIDEILEKNATEAQIEEALRKVCNYLPASIQTECDQLVQEYEPMLVQMILQTLDPDFVCLQIGACPEPLRRLMGREQCTMGPSFWCKNMMTAIQCDVRHLHIRYSIE

>Lectin24

MAVLQWELPTELCCRPMAFVALTGLDVVYNAVHRAIWDVFSANRRADRVPISFKILPGDHEYPKCRTKRTSYEWYIPKGILKTSWMNKHLNEVPSLVVLFYELDWDDPQWKEKQSECATKVEIVRLENAFYEHAQTYYYNEIRRVKSHKEFLNKTTHQLLFVRHQFKIAFFSELKQDTQNALKFQSFGELFDDAIKLGLAAIQTQNPGFYYQQAAYYSQERKRLALQLSQVNFKLFQSFGELFDDAIKLGLAAIQTQNPGFYYQQAAYYSQERKRLALQLSQELIITLLSNAVAQFKKYKCPRMKSHLMVQMGEEYYHAQDYAKALKLLDYVMCDYRAERWCGIQTAILTTALHCAYLMASVKDYIIFCMELLGRASTLKKEQKLRIEQNLIQVLKNKIPDAEPQCDQVSAQAGRSLWNECMASGGSNEFIIDVQDYIPFIQCKAKFRSPSFHVDEVIQLQVLVRADCPNPVSFTKLAVTLSNQILYYLQIMSRVPKISVQLSHQPPALSNEMYCIHCTVQSLEEGVAKDVKLTAGLKPVQCKAKFRSPSFHVDEVIQLQVLVRADCPNPVSFTKLAVTLSNQMTGIEVMLGSGSGRCVFLRWRGAGGDAATTHEFLQSNKSSSWPDTEEDLDWESMTILQNTMIMSRVPKISVQLSHQPPALSNEMYCIHCTVQSLEEGVAKDVKLTAGLKPGQDANLSQTTRVTLDNSKACDDTTPSLLPDILLGNLKPGKKVQILSLGFLILRKDECASECLCLRCPPLADNNNDVATGQYVILWRRHSSSPDSPLISTTVTLPHASLERVPLYIHADIPPFGRVRESLPVCFHIENRTALVQEVQISVEPSESFMFSGLKQVHIRILPGSKQQMLYNYYPLIAGYQTLPQLSISVDRCTTKAYNLRRFLPHRIFVKKNQTNIYTVCIGDLMRKQNPFHPSSRIQDGILPGFSTDHLAKSDHLLNDIFPTDTNHSERNELEVTLNNLTRNKDQLQTNYESLVSDRARLLDNEMALTAERDELRANFRRVKQQHDELQTNYSLLNRETDVLHTLTSALGTDKDQLRARYESLAKVKDQLQTSYNSLVKEQESLQANKTTVTAERDDFRSRLNNLEKENDQLQRDYTSLRNEKDQLQTTYESLVKEKDKLQTSYNSLVKEQESLQANKTTVIAERDDFKSRLNNLEKQKEQIQRDYSSVRSQKDQLQVSFELLVKQKEQLQTSYNALVQEKELLLTSKTNVAAERDDFKSRFDKLKNEKDQLQRDYSLLRKDKDLLQVSFDSLIREKDQLQASKTTLSAERDQLWSRFNSLKNEKEQIQRDYSSVRSQKDQLQASYELLVKEKAQLQSSYNSLGREKHLLLTSKAALSTERDNCMSRFNNLKNEKNQLQRSYSSLGSEKDQLNARYASLVQEKELLRANKAKVASERDVAKSFYDNVKKDRDHLQKNYTLLEEDKDRLERNYTLLSERSSMCQIRYKLLQRQKTYVDTTCNTLRVEYERLRRNRSSLETERYELQKTVDKMTIKVKSMLCGVGWKKFGGNCYFVSRLNRTWAESRTDCISRGADLVVINSREEQAFLNGLLGNSRDAWIGLSDHIEEGKWVWVDGTPLTTEFWKAGQPNDLRNQDCGEILAKQSQLLTWNDYDCVHQQRSICEK

>Histone48

MARTKQTARKSTGGKAPRKQLATKAARKSAPSTGGVKKPHRYRPGTVALREIRRYQKSTELLIRKLPFQRLVREIAQDFKTDLRFQSAAIGALQEASEAYLVGLFEDTNLCAIHAKRVTIMPKDIQPGTVALREIRRYQKSTELLIRKLPFQRLVREIAQDFKTDLRFQSAAIGALQEASEAYLVGLFEDTNLCAIHAKRVTIMPKDIQLARRIRGERA

>BPTI18

MGCVPVILALVLAVVVPSLAGYIEALAANAGTGFAMSEPQVAMFCGKLNMHVNIQTGRWEPDPSGTKSCVGTKEAVLHYCQEMYPELQITNVVEANQPLRIDNWCKKEKKVCKGHGHIVVPYKCLVGEFVSDVLLVPEKCKFFHKERMDLCASHQQWHGVAKEACSKSSTVLHSYGMLLPCGTDKFHGTEFVCCPPSRTEESADSAPEHEEEEEDVEDEEIDEAKLDDDEDALENSEAPAVEQPTQKEEPLDEEDTEEDVDEADEEDYHYVYEDEEADNDEDHKRDSSRMSDSREDEDKTLQEVKAVCTLEAETGPCRASMPRWHFDLSQRKCVLFIYGGCAGNRNNFDSEEYCMAVCKRLATLPTPQPADDVDIYFETPADDKEHSRFQKAKEQLEIRHRNRMERVRKEWEEADRQAKNLPKAERQTLVQHFQAMVESLEEEAASEKQQLVETHLARVEAMLNDRRRLALENYLAALQADPPRPHRILQALRRYVRAENKDRQHTIRHYQHVLAVDPEKAAQMKSQVMTHLHVIEERMNQSLSLLYKVPYVAEEIQDEIGELLQEQKANMDQFLSSISESQPDVTVSSEENVEVPVVSEGKPYRPIQVTSLGSRPEPEGSAVSGLDGLIGAEERIINSKTKMGPNVVIDESLDVKEVVYSAERLHILHDDELETYRPLGEDFSFGSSVLIGLLVIAVAIATVIIISLVLLRKRQYGTIRHGIVEVDPMLTPEERHLNKMQNHGYENPTYKYLEQMQI

>Throm34

MAGLLVWHFHFRKDVRVKKMYSGSMRITNQVFEDAYENANSSEFQALAKQVKTQLKSLYSKSPQLAKYYVGSTVQAFSEGSVIAYYISEFSVPSGQEAAVDGAMSAMDKLVDKEQRSGYRQGNALLLEDVVSSELDSRLFSTEFSRFLRFSEHTRTNHIGQIRSPGFPDSSYPPNSFIQWQVRADPNYIIKLDFDTMNLEENCNNDFVKIYDSLVAIEANVLQEMCGYYSPSEPLTFLSSGNVMLVTMATNEEKNYPGFRAQVSQVRRGSTRTTCGSQLTGEVGRFTSPNFPNYYPPRTSCKWTIEVPAGNAVKVTFKKFLMSEPGQDASKGCRKDYVEINGKKFCGDQPDGSLTETSRTNKMTVQFFSDGSYVDIGFDATYEAVDVTDPCPKQFQCKSQRCIKSELKCDGWNDCGDMSDELNCKCNSSHIHCKNGLCKPMFWKCDGVDDCGDGTDEKNCGTCSTGQLTCKNGKCVSEKNRCDGRDDCGDGSDELDCGRGSGSGVCTDLTFRCKNNKCISKVNPECDDIKDCEDGSDEDNCDCGRSKFKTSRIVGGQDAVAGEYPWQVSLHVKRYGHVCGASLISPRWLVTAAHCVQDDRSTRFSQPGTWEAYMGLHSQRNISSSVMKRNLKRVIPHPNYNDYTFDNDIALMELDTPVTYSDYIQPICLPAAQHDFPVGNVVWITGWGATREGGFAATVLQKAQVRSINHTVCDSLMGGQLTSRMMCAGVLSGGVDACQGDSGGPLSIPSGSRTFLGGVVSWGDGCARRNKPGIYTKVTKFRGWIKEKTGV

>Throm64

MLPQFPCDVTVKKTSEVIFDCNGRRLQKIPYGITSNATVLKLSENQIKHIKYASFSGLMNLTHIYLNQANGRQSVKIDDNTFRNLTNLRVLELNGNCLSAVPLTLPLSLDDFELKTNRILSLDNSSLIGLTNVTKLLLSENCYYWNPCGTNMNIADGSFKAMSKLRNLDLSFNNLTNVPKGLPSSLQMLQLASNQIQYISNDDFRGINQLKILKLQGNCPRCQNAPFPCVPCQNESLAIHADAFEDLTQLQTLNLAGNSLMNITSSWFKNLNQLKELFVSFNFLQTVFTEEATFLAYLPKLEKLDISFNFLLKIYPQTLNLSHYFSRLESLRTLHLAGLVFQEIGADTLKPLYKLKNLSALDLGTNFIIQFDSSIFSKFSNLKMIYLAENRLYPTNVKSVMHSSEGYQSISAFRSHHQHESSLFEIRHGQIKQECCDSGRVLSLSSNNLFFISPQQFEGYINITCLNLSGNGFSSALNGTEFSSIPTLTYLDLSFNKLDLAYDNAFSELKNLQVLDISYNSHYFQSYGITHNLNFLKNLPVLRVLNMSHNSISTLTTKELYSRSLSELQFQGNRLGHLWKEGDHSYITLFRYLTNLTLLDISSNYITRIPDSVYEFLPRNLTTLRISRNFLTGFGWDKLNHFLQLQSLDLSFNVISDVTGINWSTPITLISLDLSHNNIYHLDDGFLKGAKSLTTLSLSFNKLTLINQSTFQLSPDSPMQKLFLGNNPFQCTCHMLDFILWIENTGVHIPRLTTDVNCDAPENQKGRALIYFDIYQCVNDDHAFLLCLVSSSFIIVFLFVVTSAHLFYWDASYILHYVKAKLMGYRTLNSSHNVYDVFVTYDTKDPQVSEWVMRNLRVKLEEEGEKHLPLCLEQRDWLPGVPLMDNLLQSIRQSRKTMFVLTEGYVKTGVFRLAMYLAHQRLLDENVDVIVLLMLEPVLQHSYFLRLRRRLCGKSVVEWPKTAAAEPWFWQNLRNVIRVDNQVMYNKTYSMYFTNRFDVLHRATAVYATMCDKPDLEEVKSFDKTKLKKTDTCEKNPLPTKESGQLRQVSTGNQPGVCRYGRRLECCYGWKKNRRGQCEDLNECGLIPRPCEHRCMNTHGSYKCYCLNGYTLMTDGSCGNSRTCSLAHCQYGCEEVQGETRCLCPSVGLQLAADERTCVDIDECATGSNLCPYNRQCVNTFGSYFCKCRDGFDLKYVDGKYDCVDLDECASSANKCSRQAVCLNTQGSYKCKCKSGFRGNGFECSAIPDSEERPGNLGSKLENEGIKNIIPEPVVTPPPRTHQQPFDYAGEIHTGNPTVETPVDEFPEEEEEEEEEEEDNQLNPRGDVFKFIMDCNFDQGACEWVQDKTDDMDWSVAYHGRGAEYYMSMSGLLGEEEDVAKLKLLLSDRTTQGSFCLTFNYRVVGHNVGTLKVLLDNNAYPVWEQSHSRNQEWQTELLTVAWKEEAPQSIIFEAQRGRHVGGEIGLDNVVLTSGPCQEDDSPIF

>Amyloid1

MGGHGAIVLLLAATLTLSSEVPTDDSEGLMTEPQVAMFCGKLNMHINVQTGNWEPDPSGSKSCISTKEGILNYCQEVYPELQITNVVEANQPVSIPNWCKTGRKPCRSHNHIVVPYRCLVGEFVSDALLVPDKCKFLHKEYMDQCESHLHWHAVAKESCIDRAANLHDYGLLMPCAIDLYRGVEFVCCPIEAEQESDSAGLDGEESDVWWGGAEPENNDNSMTQQTDSEPAAAEEEDEEDDEEADTFERDENDDEEIDEEVEEEEEEEEEEDEDMTNERDSDERSAHVAMTTTTTTESIEEVVRAACWARAESGPCHDMLERWYFMPEKARCAPFLFGGCGGNRNNFDSEEYCLAVCSSSLPTLAPSPPDAVDQYLESPGDDNEHGDFQRAKESLEAKHREKLSQVMKEWEEAERKAKNLPRADKKAVIQHFQEKVEALEQEAAGERQQLVETHMARVEALLNSRRRMALENYLSALQANPPRPRQVLSLLKKYVRAEQKDRQHTVKHYEHVRTVDPKKAAQIRPQVLTHLRVIDERMNQSLGLLYKVPNVADEIHSQVSVIVQRVQSELSQQVSSLQSDNRVDGRMSYGNDALMPDQAYSSAPMDPSLDRLGFIHPESFNQPNTENHVSALKPEEMPQVHLETHERQSAGYEVHHQKLVFFAEDVGSNKGAIIGLMVGGVVIATVIVITLVMLRKKQYTSIHHGVIEVDAAVTPEERHLAKMQQNGYENPTYKFFEQIHN

>CDK-like12

MDESPSQSADTASMSTKLGASQRKDCGYGRASKRLKTDGETVADARGGGKDLEEGEQDSDSSSDVDLAADNRAQNVAGREDDSKSSHHISPSPVVHVRGLCEAAVEADLIDALENFGAIWFKSNQYAQKAKAALNGADIYAGCCTLKIEYARPTRLNVIKNDSESWDYTKPYLVRRDHGKGRQRQAILGEHPSSSGDNNYGSHSSLLPMPSSSRYKLTSVDVPDVVSYPLPQASYSSYAPSSVAMVSGLHPSKMNCARIFNLFCLYGNIEKVKFMKSVPGTALVEMGDDYAVDRAITHLNSIKVFGKRLSVCVSKQHAVIPSQFFELEDGSSSYKDFAMTRNNRFSSAGQASKNIIQPPSAVLHYYNVPSCISQDHLLRVKFMKSVPGTALVEMGDDYAVDRAITHLNSIKVFGKRLSVCVSKQHAVIPSQFFELEDGSSSYKDFAMTRNNRFSSAGQASKNIIQPPSAVLHYYNVPSCISQDHLLRLCNEHDVPGFVKFKMFDAKSSSKTISGLLQFDSKTEAVEVLTVLNHYQIRIPTMDDYLRIEKIGEGTYGVVYRGKHKATGQIVAMKKIRLESEEEGVPSTAVREVSLLQELKHPNVVRLLDVLMQESRLYLIFEFLSMDLKKYLDSIPSGQYMEPMLVKSYLYQILEGIYFCHCRRVLHRDLKPQNLLIDNKGVIKLADFGLARAFGVPVRVYTHEILEGIYFCHCRRVLHRDLKPQNLLIDNKGVIKLADFGLARAFGVPVRVYTHEVVTLWYRAPEVLLGSPRYSTPVDVWSAATIFAELATKKPLFQGDSEIDQLFRIFRTLGTPDNVVWPDVESLPDYKSTFPKWKPGNLASTVKNLDKNGLDLLKVDEAYHDDLEQEKVSRVSKKCVKCKETIAVVVIRAGDQYCRDCFEQYFLHKFRATLGKNRVIFPGEKGLSQNAHKKLRFVPGIVYIDEGGALNRSMEERRQTVAKLKDIFIATGFPFHIVPLEEVLDVPGSVLATAPSSSPTQASSDKASVDGFVCNNSCTCLTPAQQQQHETSSPYVEESQTQLLQQLLDSSKTVTAKAELVSVLRQHLLVHTARREGYSKLMFGDNCTRLAVKLLISISLGRGAQLAQDTSFSDSRYGDIISLRPMRNYSAKEIAYYNHIFNVPSVFTPNLHTKSKEKASIQCLTESFVTKLQVDFPSTVSTIYRLVYFRWPMIQYLILAQPLEIFVIITKFCVFTSTPDENFAVS

>Transfe3

MSGFVTLQLHKCDAQANLRADLPTSYWHLIKFTRMRVSREPRGKKNVIMATWSTTAGALLLILHAVCGQSSIRWCTISDAEQRKCEAMSRAFDQVSIRPSLSCVRGTTVENCVQKLQKNEADALSMFASDIYNLGKSASFKIAASESKADHTGASYFAVAVAKKVNSGININNLAGKRSCHTGKGRTAGWKMPIGHFIDQGYMSVMGCDFSKGVAQFFNSSCIPGANEAGDPESLCKLCVGDGKGQHKCEASNNEQYYSYEGAFRCLAEGAGDVAFIKHTTVEDNTDGHGPDWAHKYKSADYELLCRDGRRRPIDQWESCNLVRIPSRGIVVGNHVTPSVVFNMLSQGLESGFNMFSSKPYGEGTLLFSESSVTFQSTEYDDPKKWMGQLYHNAMSAMDCKPPESPLRWCVLSTGEQQKCAAMGSAFQSKGLTPLVNCIYGDSVTDCMEKIKNKEADAITLDGGYIFTAGKDYGLVPATGESYTADRDGSNYYAVAVVKKSSYGIRSLDDLKGLQSCHTGLGRTAGWNVPVSTLIEKGLISPQHCQIAQAVGGFFKQSCVPGANQPGFPTNLCGLCTGDILGQNKCEKGKDLYDGYDGAFRCLAAGGGDVAFIKHSTVFQNTDGNSDEIWARDLQSKDFQLLCPHSTKAEVTQYKYCHLARVPSHAVMVRPDTNIHAIYGLLDNAQTYFGSDTGTEFRMFDSQEYRGTDLIFKDSTVRLVGVGDRKIYQEWLGQNYLDSLINMECNASCAVISSLWLVTVALLCSVLTAI

>Lectin13

MTDKGCSHGNFYYGVAPELPSVERSHVRSYTGVAPGNILNRSKFNPAQESVCNILEGNLVSIHSFLENAFVREMAAAGGNVDQIWIGLTDVLLETDYIWTDGTVNDFQNFDLPPPANNNNNCVQLRQSDGEWVTAQCIDAEQYVCIRDVRH

>Histone47

MARTKQTARKSTGGKAPRKQLATKAARKSAPSTGGVKKPHRYRPGTVALREIRRYQKSTELLIRKLPFQRLVREIAQDFKTDLRFQSAAIGALQEASEAYLVGLFEDTNLCAIHAKRVTIMPKDIQLARRIRGERA

>Neuropep10

MLKPCMMFAALLLCLLVFWGSLADAYPPKPESPGSNASPEDWAKYQAAVRHYVNLITRQRYGKRSSPEQAVAWLLYGAEPSQDSEPRITLKKRGGLNPQEHVDAMPAGDDAMLIEQFAVGVTEHRKVCEKDE

>Throm45

MAAALRVVCLLMLPLLSEAAFTYMPGKQHKVLHLDWPRDCGMAYFNPNMAERIVSGNEARPHSWPWQVSLQVRPRGSRHHIHVCGGTLIHKNWILTAAHCFQKGKAEDASSWRIVLGKHQLRRSETAERVFPVKRIYRHENFRYPAHSELDYDIALVKATTDILPSNFIRYACLPRKKTILKPGQYCWVTGWGDTRGGKENVSLAEALNQARLPIIDFKTCRQKKFWGDRVRDSMICAGFRDTEDPPAACQGDSGGPLLCQLGRERWEVHGVVSFGPIGCTVENKPSVFTRTGAYIPWIEATRIRDFFLH

>Ubiq7

VTLEVEGSDTVENVKAKIQDKEGIPPDQQRLIFAGKQLEDGRTLSDYNIQKESTLHLVLRLRGGMQ

>Ubiq8

VTLEVEGSDTVENVKAKIQDKEGIPPDQQRLIFAGKQLEDGRTLSDYNIQKESTLHLVLRLRGGMQ

>Ubiq12

ITLEVEGSDTVENVKAKIKDKEGIPPHHQRLIFAGKQLGCDRTLSDYNIQKEATLHLVLRLRGGMQ

>Ubiq10

VTLEVEGSDTVENVKAKIQDKEGIPPDQQRLIFAGKQLEDGRTLSDYNIQKESTLHLVLRLRGGMQ

>Ubiq23

TLTGKTITLEVEANDTVENVKAKIQDKEGIPPDQQRLIFAGKQLEDGRTLSDYNIQKEATLHLVLRLRGGMQIFVKTLTGKTITLEVEDSDTVENVKAKIQDKEGIPPDQQRLIFAGKQLEDGRTLSDYNIQKESTLHLVLRLRGG

>Ubiq6

EGSDTVENVKAKIQDKEGIPPDQQRLIFAGKQLEDGRTLSDYNIQKESTLHLVLRLRGGMQIFVKTLTGKTVTLEVEGSDTVENVKAKIQDKEGIPPDQQRLIFAGKQLEDGRTLSDYNIQKESTLHLVLRLRGGMQIFVKTLTGKTITLEVEDSDTVENVKAKIQDKEGIPPDQQRLIFAGKQLEDGRTLSDYNIQKESTLHLVLRLRGGMQIFVKTLTGKPVTLEVETSDTVENVKAQIQDKEGIPPDQQRLIFAGKPLEDDQTLSDYNIQKESTLH

>Ubiq3

EGSDTVENVKAKIQDKEGIPPDQQRLIFAGKQLEDGRTLSDYNIQKESTLHLVLRLRGGMQIFVKTLTGKTITLEVEDSDTVENVKAKIQDKEGIPPDQQRLIFAGKQLEDGRTLSDYNIQKESTLHLVLRLRGGMQIFVKTLTGKTVTLEVEGSDTVENVKAKIQDKEGIPPDQQRLIFAGKQLEDGRTLSDYNIQKESTLHLVLRLRGGMQIFVKTLTGKTITLEVEDSDTVENVKVKIQDKEGIPPDQQRLIFAGKQLEDGRTLSDYNIQKESTLH

>Ubiq1

MQIFVKTLTGKTVTLEVEGSDTVENVKAKIQDKEGIPPDQQRLIFAGKQLEDGRTLSDYNIQKESTLHLVLRLRGGMQIFVKTLTGKTITLEVEDSDTVENVKVKIQDKEGIPPDQQRLIFAGKQLEDGRTLSDYNIQKESTLHLVLRLRGGMQIFVKTLTGKTITLEVEDSDTVENVKAKIQDKEGIPPDQQRLIFAGKQLEDGRTLSDYNIQKESTLHLVLRLRGGMQIFVKTLTGKTITLEVEDSDTVENVKAKIQDKEGIPPDQQRLIFAGKQLENGRTLSDYNIQKESTLHLVLRLRGGMQIFVKTLTGKTVTLEVEGSDTVENVKAKIQDKEGIPPDQQRLIFAGKQLEDGRTLSDYNIQKESTLHLVLRLRGGMQIFVKTLTGKTVTLEVEGSDTVENVKAKIQDKEGIPPDQQRLIFAGKQLEDGRTLSDYNIQKESTLHLVLRLRGGMQIFVKTLTGKTVTLEVEGSDTVENVKAKIQDKEGIPPDQQRLIFAGKQLEDGRTLSDYNIQKESTLHLVLRLRGGMQIFVKTLTGKTITLEVEDSDTVENVKAKIQDKEGIPPDQQRLIFAGKQLEDGRTLSDYNIQKESTLHLVLRLRGG

>Throm40

MEKIARQPFGRLEGRRNALQDYQRTDGVRLFGTSPSSSHLTKSRKVSMGKCARICSRNKKLPFTCRAFLYDQQNRKCQWLSFDRNSPVAQMQHDLNVQLYQKKDYIRECIIGTGESYRGRRSMTVTEILCQSWASPIPHEHKFMSKRFSKKDLRENYCRNPDNSTVGPWCFTTDPRHRHQECGIPQCSQVECIHCNGEEYRGPMDHTESGKECQRWDLEEPHKHLYHPDRYPNKGLDDNYCRNPDGRHRPWCFTTDPNTNWEYCAIKVCESPPKRTTVETSECYQDKGEAYRGTVDMTPTGLTCQRWDSQYPHNHSFIPQAYTCKDLKENYCRNPDGQEFPWCFTTDPRVRTMLCTNIPQCGSQNKPDCYEGFGEKYRGEQSRTRYKLLCAPWGEHSNSGERGVTVAGLERNYCRNPDKDKHGPWCYTNNSAIRWDYCNVKPCDASQNTISLGELSSVGCFVHKRPRIVGGGPLSISAGSWMVSIQKGSAHWCGGSLVREEWVLTDRQCFSSCVPDLREYRVWLGVSNIAANTSDWATRQEVRIAQVICGPDGSSLALLRLSKPALPADNVHTIQLPVDGCSIPEGTLCKMYGWGETKGTGHDNVLKAVNLPIVSNERCRELHRGSLHITNSKICAGGRRNEGVCERDYGGPLVCQDGEIKVVVGVSVHGRGCARFNRPAIFINVPFYTQWIYKVFRHYPEPDLSTA

>Ap1

MSNNYGEEPQYYQAADFGGGEEEDEVPATEKDLAEDAPWKKIQQNTFTRWCNEHLKCVNKTIGDLQRDFSDGLKLIWLLEVLSQKKMYRKYHSRPNFRQMKLENVSVALEFLDRERIKLVSIDSKAIVDGNLKLILGLIWTLILHYSISMPMWDDEDDEETKKLTPKQRLLGWIQNKVPQLPINNFNRDWRDGKALGALVDNCAPGLCPDWAEWDPNQPVENAREAMQQADDWLGVPQVIAPEEIVDPDVDEHSVMTYLSQFPKAKLKPGAPLRHKQLFPDKAKAYGPGIEPRGNKVLHPAVFTVETVEAGSGEVLVYVEDPEGHKEEAKVKPNKDKNRTYTVTYVPKVEGLHKVKVLFAGQDIDKSPYAVNVAKDMGDPSKVHARGPGLERTGNVANKATYFDIYTAGAGNGDVSVVIVDPQGRKDTVEIILENKGDSVFRCTYRPVVEGPHAVHVLFGGQEIPKSPFPVNIAEAPPVAPSAGAPLQIIPQSVLTPPATKGAPPKKPVRPTINPNACRATGRGLQPKGVRVKEVADFKVFTKGAGSGTLNVSVKGPSGAQEQVKVRDAANGVYDCEYYPLKPGKYTVVITWGGQPIPRSPFEVEVGAEAGFQKVRAWGPGLRTGTVGKSADFVVEAIGADVGTLGFSIEGPSQAKIECDDKGDGSCDVRYWPTEPGDYAVHVICDDEDIKDSPFMAHILPAANDAFPEKVKAFGPGLQPTGVTVNKATEFTIDARAAGKGPLTIYAQDAEGCAINIKITDKGDGTYLCTYTPTKPIKHTVIIAWSGVNVPNSPFRVLVGEGCHPDKVKVYGPGVEKTGLKANEPTYFTVDCGEAGQGDISIGIKCAPGVVGPAEADVDFDIIKNDNDTFTVKYTPPAAGRYTIMVLFADQEIPISPFKVKVEPSHDAGKVRAEGPGLNKTGVEVGMPTHFTIYTKGAGKAKPEVRFAASGPGEAVRDFEIIDNHDYSYTVKYSALQQGKMAITVTYGGDPIPKTPFHITVAPQLDIAKVKVEGLDTKVEVGRDQEFTVNAKGAGGQGNVGVKMTSPTGRPIPCKLESDKAKGLHGVKYIPPEEGQYKVDVSYDGNPVMGSPFGVEAVMPADPSKVRAFGPGLQGGVAGKPAPFTIDTKGAGAGGLGLTVEGPCEAKIECQDNGDGTCSVAYLPTEAGEYAINILFAERHVPGSPFKAAVRPAFDPSKVTASGPGLERAKAGETATFAVDCTRAGDAELTIEIVSETGAKAEVRILKTAEGTFSVTYVPPFHGAHTITIKYGGHAIPHFPKVLQVEPSVDTSGVRVYGPGVEPRAAPKRERVRAARDIARPCRRAPEDDRPEGFRGEPQITRRSALFPGVLREVTTHFIVDARALNKVGGNHVKARIVSPSGTATDGYATDKGDGTYRVEYTPFEDGTHLIEVLYDDAPVPESPFRVSVAEGCDPTRVRAYGPGLEGGVTNKPNCFTVETRGAGTGGLGLTIEGASEAKISCKDNKDGSCSVEYVPFTPGDYDVNINYGGHPIPGSPFKVPVKDPVDASKVKCSGPGLGSGVRAHIPQTFAVDCTRAGQAALDVKLFGPTGTMEPVGVKNNGDGTHTVHYTPAQDGPYTVAVKYADQQVPHSPFKVMSQPGHDASKVRASGPGLDSKGVSASLPVEFTIDARDAGEGLLTVQILDPEGKPKNASIQDNRDGTYTVSYVPDSIGPYTITIKYGGDEIPYSPYRIQSNPTGDASKCRLTVSIGGHGVSGLQKLQTSEDTVITVDAKAAGKGKVTCKVLTPQGMELDMDVVENRDGTFDIYYTAPEPGKYVITIRFGGQNIPKSPFHVLASNEPVAPRDTVDPLFRPVNFLVPFMPQQGEISGEVRMPSGKTARPHITDNKDGTITIKYQPTERGLHEMDIKYDGNHIPGSPLQFFVDAVNSGVVAAYGPGLSYGMVNKAATFTVVTKNAGQGGLSLAVEGPSKAEITCKDNKDGTCTVSYLPTAPGDYNIIVKFDNKHIPGSPFVAKITGDDTITRTSQLNVGTAADVSLKIAETDLSSLSASIRAPSGNEEPCLLKRLPNRHLGISFTPKEVGEHEVSVRKNGVHVANSPFKIMVGQSEIGEASRVKAFGKGLVEARTFEMAEFFVDTRNAGYGGLALSIEGPSKVDINCEDMEDGTCRVTYCPTEPGNYTVNIKFAEKHIPGSPFTVKVTGEGRMKESITRKRQASSIASVGSTCGLNLKIPGNWFQMVSAQERLTRTFTRSSHTYTRTERTEISKTRGGETKREVRVEESTQVGGGASPFRDVFGDFLGRESLGSFAGIAARPEAAELGSQAMTAQVTSPGGKTVDADIMDGGNSTYSVRFIPQEMGPHTVNVKYRGQHVPGSPFQFTVGPMGEGGPHKVRAGGPGLERGVAAAPSEFSIWTREAGAGGLSIAVEGPSKAEISFEDRKDGSCGVSYIVKEPGDYEVSIKFNNEHIPDSPFIVPIATLSDEARRLTFTSLQEKDLKVNQEASFLVQRNGTRGVVDAKVHTPSGCSEECYVTELDGGKPLFPLYRFPTSTDEPQRKQDKTAVRFIPRENGVHSIDVKFNGCHIPGSPFNVRVGDPGLVGDPGMVAAHGPGLLGGNTGAPADFVVNTCNAGPGTLSVNIDGPSKVKMDCCECPEGYKVTYVPMAPGNYLIAIKYGGPQHIVGSPFKAKVTGARLSGGHSLHETSSVLVETVTKSSQFGASASAASSKLTSDAGMVVCRGMGLSKAAVGQKNNFTVDCGKAGSNMLMVGVHGPHAPCEEVYVKHVGNKLYNVTYTVKDKGSYTVIVKWGDDNVPGSPYKVAVL

>Throm3

MSTKNVVFKKICKDKSVGVYMGRRDFVDRVDSVDPVDGVIVIEREALQGRKVFVTLSCTFRYGRDDMDVMGVAFRRELYLSTRQVYPPLQDREQGVHTRMQAKLLRKLGDDAYPFFFEIFYHGETMRLHLNVVNNSTRNVKNMVISVDQVATVVLYSNDSYVKCVAIEEGETVSAGATFQKDILLLPLLANNRERRGIALDGKLKHEDTNLASSSIIKPGVLKEVLGIMVSYRVSVKLIIGGEVSLEIPFKLMHPKPDKAVCLQIFYHGETMRLHLNVVNNSTRNVKNMVISVDQVATVVLYSNDSYVKCVAIEEGETVSAGATFQKDILLLPLLANNRERRGIALDGKLKHEDTNLASSSIIKPGVLKEVLGIMVSYRVSVKLIIGGEVSLELPFKLMHPKPDKVRALSAWCDRSQILTDMRRRAVCICLTLAAWAVSALNLSVFSRPPEAHMLLRSRRANSFLEELKPPSMERECVEETCEFEEAREIFKVREATLEFWTVYRDGNQCESNQCVHGRCVDMLQDYVCSCNPGYEGKYCEHAQSATNCSVNNGDCDHECTESEDGLRRTCSCVDGYKLHDDAKKCVAKGSSSCGQLLIGRSSYTKPMDGLMPWMVGGEVGKKGESPWQVLLQNAKGRFHCGGVLIDQNWVLTAAHCLENNLRFRVRLGDYERFRDEGTEVMLKVSKAIQHPDYDSTTVDNDIALLRLERPAPFSEYILPACLPSRKMAEAVLHRNGTQTVVTGWGKESQESQRFSSALNVIRVPLVSRDVCATHMLHNISENVLCGGILGQSIDACEGDSGGPMVTLYSDTWFLVGLVSWGEGCGRQDKLGIYTKVSNYNEWIDSVRQDWDKSPQPPEA

>Lectin19

MKDAAGLFVAALAVLVGRLGGTEPDGGLCVDGSCFAVFRHSGDFTSAQSHCEERNGHLMAVGSFASRDVLAQLLRNSSGRFWIGLHRSTGCPNATSPLKGYEWTAKDAQGDLADWAPSFDGSCSSPRCVAVSEADRLRWVQEPCDAPVTGFLCEYHFAYSCESITIDGEAPAYDTPLGFGGGDLHSLPPGSIAALTPSKRKFVCSDSWVEAPWSCEILEGGCEHKCAMNDDHEPSCSCPPGLSVNPANEITCEVDALDPCARLLCEYACFEGDDGTHACACDHGFTLAPDGRSCVDFNDCSDERQCPGDNSRCVNTVGGFECECEGGFKLTDGSCVDRDECASAPCEHMCDNTPGSYKCSCYGGYRVHPLDANKCELHCGKKECVAECDPNDKYQCYCPNGYIAEEREDHTVCIDIDECGNSYCDHGCRNTYGGFVCSCRKGFTLVDEVFCDRNEDEDEEAVGSGEAATTPSAPTVTPHEVAPTRQPSAVTVGGLVAIIVCCVLVVVVVVFLSQHILNKKGKVERGAASKAADGGETHSLHGMGSEA

>Lectin2

MLSSKTSLSSSSQPTKNLGAPHRLSPGLLGSDITVSRGSPQRPTAPSYTTAPLEQAADGRARGQRTQRVPLCLSWTAGLDHTVIDPGAPNIAPLSHRIKWSFITKDKVSTILVALEGEVRISESYLDRVHLVGYPLTPTDANIMISELRSSDSGVYRCEVQHGIEDNHDDVRVKVQGIVFHYRPIIGRYMMTFEKARDACTQNSAVMASPEQLQAAFDDGYHQCDAGWLSDQTVRYPIHDPRVNCQGDKENVPGVRTYGMRQLNETYDVYCFAEKMTGRVFHITSAEKFTFSEAELACSTKGAILATTGQLYLAWQGGMDICNAGWLGDRSVRYPINIRRPQCGGGLLGVQTVYLHTNQTGYPLLQTPYDAFCYTEFPEDEGSGLIEEESSLLSVTPVVQSHEVFFGKRTTESEVVGGVETQQPTIVDLSYTLSPSELLLPQPPNVTDIPTDIIEQVTLPSNLGREPGKVGVIAPNADILFIANETGLNITDIQDVLPNLTSITELVRLVITPIVTPIPVESPGSGSGSTNFGSGSSIDNTSGDLPGSGNQVISGDMSRSGVRLTSVDLSVSGDRGFSEDLSGYGDKLISGDVLDFGSLSDSVNQSTSGDRSGFLSFSNSGDKVISKELDGSGDMSGLGDQFMSRDLSGSGNTVGSTSTDKTHGTSGVTSADSSRSRISGEQVISAVLYSNNDTDLSGEDTISERFQKAEEAGTEIYINIKSSEIGSGRLSGSRDQPGSGLDPGFTSEEIDLSMETSGENGQFSGMSSGLIKSLDFSGGLPSGLSYSGSASGHITGAGDAKILLIDNELNDTFAPIRQKEYELGGGLLVFSGSGDSYGMPTNDLSGSASGSGSMFFSDVTFDEAAFTDLTESSFGEQESSGFSLYNSGGAIPEHLSDVRSLEPLSGAAMSGEGDSVTLLTEYVMTGVSRNLTVSEEVQQGTVQYSGEEINNSGRGFHTESRGVIMSIPSETQNEGPSQVMTSPSTQWELIETATVTNVPKSLSRALASKLAPAGLSALPNVIPPVNGSQEDAEHIQELDACHSNPCANGATCVENADSYKCVCLPSYGGERCEIDEERCEEGWTKFQGNCYLHYSDRETWLDAEQCCRDLNAHLVSIITPEEQHFVNSNAQDYQWIGLNDKTIEKDFRWTDGTPLQYENWKANQPDNYVNSGEDCVVMIWHAKGQWNDVPCNYHLPFTCKKGPVFCGAPPLVENAHMFGNKMEEYPINSIIRYQCNPGFRQRHLPVVRCKTDGQWETPQVECTDVPPGFAVGPSMSCKGRLGLWWVGRALRVGELLRREGQPEWVQKFETDPLTPSDLTMTMESIHPTDSTKEERAPTQIAENKDFPWRQAVWISFACQVQSKAAHNVAVQCMCAEMCVGICVSLCLRVCHEIQPGTELLLGDETDGAKDARKRLDAQNISEGQQRDYPVEQLRDPEVAVPNPQTTEEIKIHDKTDKEERQNKDPHDRSFGPNRGKQKERSTAVNTAAKVDNIVLTSNGPNISTGLVMDAVDTLSMLNSPDKQRILSLPARPVRCSSRLAAKPRQVHCQTSHVKKLSVHSHPDRQTDMQESLTQVSEYSAEAEVSMATSHSDAVAVRAENGTVEARTWCPEVRERRYRCSECGKKFFQIAALRKHQFSHTETKPFTFLDGGQNCTSAETFLAHQLSQHRECPFSCPHCDETYDLKQDLHKHLVVHTGEKSYVCEHCGKAYERRSSLRNHRLLHCSRLIYPQLPKLQCTLCPKLLANSGSLRNHMKLHTGEKSHVCQYCGKSFNQKGNLSSHLRIHNGERPYPCPDCDQRFAQKPELSRHRLSHTGGVFLCSYCGKSLRDPHTLKSHERLHTGDRPHRCPICKKGYTMATKLRRHIKSSHVTEKPHSCHCGASYTLRQSLLRHQAQHHSREETQEGIKEGLRQKAKPAMQELAAANSHHTKPVKGRPKKGDDRVQSRGRRKGEESGTGKTLSRSETTAATGADGKASSDTQHTVVLVHTDDTSIPGYGPLLLTSDDLHSRTGEELVEVVISGGAEQCIVVQGQQTVGDLMIIQEKDVCSVAQTVEINTVLL

>Chem12

MKFQGLLFLVLLICTTVCVAQGSYGNCCLGHVQRMGEKAKKNIESYRMQETDGDCNIRAVVFTMKKRSGRKRQRTVCTDPDQLWVQDLKAVVRLRMGLQN

>GAPDH

MLIAKSLSCPSILKLIGLIPDHAERARIENLPFELQRNFNLMRDLDQRTEDLKGQIDSLAKEYTSNARTLSSEQKLSILRQIQQSYSKCKEFGDDKVQLAMQTYEMVDKHIRRLDTDLARFEADLKEKQIESTDYDSTSSKGKKVDTRHKEKKTAKTRSKVKSSDEDGSPKSSQKKVKLLQTGEFNSPSANFGNVHPSDVLDMPVDPNEPTYCLCHQVSYGEMIGCDNTDCSIEWFHFACVGLTTKPRGKCSCFYAMENKFDPYFPGMSWDFFVPVCVGDLVKTGGINQYVVQDVLSPKQLPSLLHKFKTALRSQGPLCILEHFDTAYSVLQHANSVELGVKENTLDLLVQVVSSLSTSLPSLLISTSVSAAERKENLNAVKMSVFLLCKITESLESDSYRQSFIAAPGKGGKKAKTSEGLQQWDNERERVLQALGQLIQLDIRSLWNLSLVEEEFTSCMTCCCYKLLENPTISHVKSKPTREGIIHLLGLLIKKYNHLLGASVKVIQLLQHFEQLSSVFAQAVSVWSTEYGVRAIIGEVMREIGQKSSEELAREGSGVKAFSSFLSELSALVPELMIPNISVLITHLEGESHTMRVAVCEVLGDILVRVLCGDGLEESGKADRDRFFDTLQEHLHDTHSHVRARVLQVYTRIVNSKALPLCRYSEVMGLAVGRLMDKSVNVVKSAIQLLAAFIAHNPYSCKLSSADLKKPLEKETAKLREMKEKLRGGAPVIKASELWAAMEPELRITVDTELERSGDSEKGQHEEEEEADVEKVNEMEDCRATAVMIAQYLRVNKYRNAVRLCIKALSRFPESEMFAALSALTAETLMDTLARLFKGSDEDTPEHSPNLPPTPQKEGEKEADDAELKKQEMLVQYLRDTETFALQVERAISVINAMLYWKTTSVVQEAVQFCVTVCEFGIANSLSGVRKMLPLVWSTDSAIKDAVVQAYRRLYLNPKGDTIRMKAQTLVDSLSELMVDASLGTIQCLEEIVQEFFGSGNSLESTVVQVLWGRFTGKTESSPLYRRAAVLLLGMAARAEREVVLSNLDTLCSVALGEKVTEDFLLARDTVIAICSITDHVRQSRGAPFRLPQEHQLFTRLTQAIAEGAMMEDAHWQSFMEQAVRLTYFLAESPDQLCSRLIQRTARLLLDQISEGAEVNKDVDQPQEGSQESGEQVNCVCLAQLLALCGSVAFWQVSHLERSVSSELRRRRLETEEREEKEKGPSSKAKLTANESAMEEELGLVGASAEDTEAELIRKICETELVAEENLLSAFLPLLVKVCSSPGHYSHPQLTTAACLALSQYMMISPSVCEENARLMFTVLERSTLPVVRANAIIALGDLTVRFPNILEPWTQNLYARLSDEVPSVRQTAVTVLTQLVLKDVLKVKGQVSEVAVLLIDPEPHIASLALNFFNELASKDNAIYNLLPDIISRLSDPDRGMSPEDFNTVMKQLFSYITKERQTESLVEKLCQRFRTARTERQWCDLAVSLSLLSMCERGFKKLQECWECYSDKLTEPGVYQPLLAITAKLRRGAKIQLKGQVDDFEKRLTAVHTRGLENVESPEMEDENQKEGQATDKAAARTPMPARGRARSKRGQSKSSVSSQCDDSFVTPQRTRKSKKKPVITFSSDEEEEEEDAVLTESETPKPLRFPVVQLVLFTPAAMLLCPVLLGASLLILAPIRTPQARALHPSPGSAQLMEQLLDRYNDLLTFDDLENLLSTPAEEQSTLSSGAKASEFPGKWADVQPETPWLRLLRGALANQKPVESERSRRGWNRGCFGLKLDRIGSMSGLEDSLNSGKMVKVGINGFGRIGRLVTRAAFHSKKVEVVAINDPFIDLEYMVYMFKYDSTHGRFHGEVKAEGDKLVIDGHKISVFHERDPANIKWGDAGAEYVVESTGVFTTIEKASSHLKGGAKRVIISAPSADAPMFVMGVNHEKYDNSLKVVSNASCTTNCLAPLAKVINDNFGIIEGLMVSFPTCDDFDNTVAGCLIGDLMLHNEKSTVHAITATQKTVDGPSGKLWRDGRGASQNIIPASTGAAKAVGKVIPELNGKLTGMAFRVPTPNVSVVDLTVRLEKPAKYDDIKKVVKAAADGPMKGFLGYTDHQVVSTDFNGDTHSSIFDAGAGIALNDHFVKLVSWYDNEFGYSNRVCDLMAHMASKE

>Chem4

MMSKVILGALLVLLASVAISEGMTLRSLGVELHCRCIQTESRPIGRHIEKVELIPANSHCEETEIIATLKQTGQEVCLDPAARWVKRVIERILNKGAFSVVRRCVKISSGQEYAAKIINTKKLSARDHQKLEREARICRLLKHPNIVRLHDSISEEGFHYLVFDLVTGGELFEDIVAREYYSEADASHCIQQILESVHHCHVNGIVHRDLKPENLLLASKLKGAAVKLADFGLAIEVQGDQQAWFGFAGTPGYLSPEVLRKDPYGKPVDMWACGVILYILLVGYPPFWDEDQHRLYQQIKAGAYDFPSPEWDTVTPEAKDLINKMLTINPSKRITASEALKHPWICQRSTVASMMHRQETVECLKKFNARRKLKGAILTTLLVTRNFSAAKSLLNKKSDGVKVNNKANTATSPKDTGPAPALEPQTTVIHNPVDGNKESIESANTTIEDEDVKALRLGSLVSGILSSKSRSSQMSESRQTPTYSFQTRKQEIIKVTEQLIESINNGDFEAYAKICDPGLTSFEPEALGNLVEGHDFHRFYFENALSKGNKPVHTILLNPHVHLIGENAACIAYIRLTQYMDGGGMPRTMQSEETRVWHRRDGKWQNIHFHRSGSPSIPSQ

>Chem6

MFGVFTAKPISLVERCWCRSTINTVPQRSIKELKFLHTPNCPFQVIAKLKSNREVCINPETKWLQQYLKNAINKCNPFCTAPPLLILPKSGERAIEGAAVISGARYGNNGDVPLERHSSSVLYYYLAMEEEIEKNKDHPQIAPIYFPAELHRCEALARDLEYFYGAKWQTQISLSAGTKPYVDRIHNVGTQDPTLLVAHVYTRYMGDLSGGQILKKVAQRALKLPSSGEGLNFYQFEGIHSHSGFTGVFTELEEIGKRIPEQAQEEAFGHGHAELMQGEDINKCPYYAAKMAASGNPTYICQFAKALLQHVPSQVVLTAWIALFAGFTAWYLL

>Histone3

MSGRGKKAAAKPKLPVSSSVRAGITFPVSRIYRFLKSGKYAQRVGNGAAVYLAAILEYLCAEILELAGNACRDNKKQRIAPRHILLAVKGDEELNKMFAGVTISDGGVVPNIHAALIPKRSKPIREEITAQDTQSQDF

>Chem8

MASHLPLSVLVLLLVAVTFSEGLRGFGPKNCCYRFNENPIASQRVVSYVKTPQQCPIPAVRLRTVMGRYMCARSSASWVKNLINELDDKIIPGMTNQL

>Ubiq17

MWIQVRTMDGKVTHRVDSLSKLTKVDELRLKISELFKVEPERQRLFYRGKQMEDGHTIFDYNVGLNDIVQLLIRQNMPCVEMTKNKDKEAELSDSDSGCGSTQSESDKSSTHGEMEVQTAGTSVQVSPSQVLEPGFGFYKVNEMVDARDLNMGAWFEAQIVNVTRSPKTPKVDVVDVEHEQEEILYHVKYEDYPENGVIPLLAKDVRPRARTVYQWHQLEPGMIVMLNYNPDDPKERGYWYDAEIQRKRETRTLREIYAKIILGDAGDSLNDCRIMFVTEIYKIEEPGSQSDMPAGSESPLKRSNGPECKQCKDDPDKICRWCNCNICGIKQDPDKQLLCDECDMAYHTYCLNPPLTAIPEDEDWYCPNCRNDSNEVVRAGEKLKESKKKAKMASASSSSQRDWGKGMACVGRTKQCTIVPSNHYGPIPGVPVGSLWKFRVQVSESGVHRPHVAGIHGRNNDGAYSLVLAGGYEDDVDDGNEFTYTGSGGRDLSGNKRTAEQSCDQTLTHMNRALALNCNVPVNDKNGAQAKNWKDGKPVRVVRSWKGRKHSKYCPEEGNRYDGIYKIVKYWPAKGKSGFLVWRYLLKRDDDEPAPWTRDGKERIKKLGLALQVSSYTSGSLFSDGVHQQVSACSKLELLVPIRQEAIALAHNLPDQSGVLWAEEPWLTTGGVQVGVGPEEGLYPAGYQKEKENKNEVEEEETPSKAKRKRESQSIESTKNSPAKSPKKVKVEVYKLSGEQKALIKDDTQNKKVWDEAMESLSLGPKFLNKVEEVFLCICCQEVVYRPITTECHHNVCKECLQRSFRADVYTCPACRHDLGKNYSMTVNKSLQEILNQFFPGYSNGR

>Lectin14

MLTEQEFGGVRKSAEWQEPQAPAVTGQPELRRINGLDGTLRVSIPSAVPLRPLLGGKVVVPCYFQDNTASDPGAPTIAPLSHRIKWSFVTKENVTTILVASEGKVHIEREYLDRVTMVNYPLVSTDASLEITELRSKDSGTYRCEIMHGIEDNYDTVDIQVQGIVFHYRAITTRYTLTFEKAKAACIQNSATIASPAQLQAAYDDGYHQCDAGWLSDQTVRYPIHEPRKECYGDKENFPGVRTYGVRDVNETYDVYCFAEKMAGRVFYSMSVEKFTFHEAKDQCNKLGARLATTGELYLAWKGGMDVCNAGWLGDRSVRYPINIPRPQCGGGLLGVRTVYLFPNQTGYPITSSRYDAICFQGSDDEGAVPGRITPFPDIMSITPAPGVYAGLTTPPGGQEIKHGGVDILTKPISPPFTDAAMGPTGVVFHYRPITGRYTLTFLEAQQACKSIGAVIASPLQLQAAFEKGLHQCDAGWLGDQSVRYPIVSPRDNCAGNLLHLPGVRSYGLRPDTERYDVFCYVERLEGEVFFTSDYDSFSFEEAVQHCEKLNTTLATTGQMYAAWNQGLDKCRPGWLMDRSVRYPITTSRSYCGGGQVGVHIIYAFPNQTGFPDEHSRYDAYCFKAEIRAIEIDSKTTIDTVSETIFDKLLTVDVVSKTTVATDHATTTIEPPVGYTDTETMANITEIEVVIQKTNITTQLTIPTVDGSASGSGDHLASGDLQISGDHSASGDLQISGDHSASGGLQISGESSTTSGSGASGSGLDITFSGHTDILSGDHSASSDPQGAEGGSAIFTSGDSYSGSGSGSGFLVSGSGSASGSEDISGSGGEILINILDGKERELLIPTLTEQELGSGALWTSGMSGSGSMSGSGVMSGSGSGFSGITFTDNNIIDLTVQQSREQELYGSAEMSGSGSISGSGFLSGSGSGEFSGITFTDNGAVDLTVHQSGEQELYGSAEMSGSGSISGSGFLSGSGSGEFSGITFTDNGAVDLTVHQSGEQELYGSAEMSGSGSISGSGFLSGSGSGEFSGITFTDNGAVDLTVHQSGEQELYGSAEMSGSGSISGSGFLSGSGSGEFSGITFTDNGAVDLTVHQSGEQELYGSAEMSGSGSISGSGFLSGSGSGEFSGITFTDNGAVDLTVHQSGEQELYGSAEMSGSGPISGSGFLSGSGSGEFSGITFTDNGAVDLTVHQSGEQELYGSAEMSGSGSTSGSGSMSGSGIDGFSGISFTDNSAIDLTIQPSGDQELSGFPPYASGFPSGAPSGFPSGVSGSSSASGQPSQTEGDIIFLTDDGIKEVIVSPQEHHPEEGRGVVEISGQESGSGTHLQISGSDKPSTAGLSIAFPPGSTMTYPDYNGPSGHEEATVIVMTPDPAYVSPTTAPTISLATAPVVEEPEVEEAVNTCPEGWLEFMGSCYLHFADRHTWSDAEQRCRELNAHLVSVRSEEEQQFLNSKGQDYEWIGLNDKEVQNEFRWTDGSALTYENWRPNQPDNYFNSGEDCVVMIGHEGGQWNDVPCNYYLPFSCKTQPAGCGAPPEVAHGSPMGVTRDRYAVNSKVRYQCQAGYTQRHLPVIRCMENGQWESPQVECTETAGNRLHKRSVRSRSRGVSSRREQSKTL

>Neuropep8

MDALMKGFSMAKEGVVAAAEKTKAGMEEAAAKTKEGVMYVGNKTKEGVVSSVNTVANRTVDQANIVADTAISGANEVSQAATEGVENVAASTGLLNQVGYRGNRLPVRGPFPLSLFLSTFLCLCPAPPRRSP

>Histone41

MKVFFLFRPGTVALREIRRYQKSTELLIRKLPFQRLVREIAQDFKTDLRFQSAAIGALQEASEAYLVGLFEDTNLCAIHAKRVTIMPKDIQLARRIRGERA

>Throm23

MSLKSRLIATFLGVTGFLFSYAEECGRPTLQENRIVGGMDASDGVWPWQVSVQKDNGHVCGGSIITENWILSAAHCFPNTSDVSSYTIYAGWYFLNSFNPHQSAHRVNYVVIPSGYVEPHSGKDVALVRLATPLTWSDWIRPVCLPASGTLFPGGMLCYVTGWGDIRNSVPLPGVGPLQEVQVPIISEGTCRDMHQMEQVDILYDMICAGYQEGGRDSCQGDSGGPLVCQMVNGTWVQAGVVSFGLGCAEANQPGVYARLTSYSSFIRDNIPEVRLYGRAQRHRLEGAAAALLCCLSAILVLRWL

>Throm18

MNFVFVCFPSVSCSAGCPPLNSKLVGGGDAQEGSWPWQASLQNFGSHVCGGSLINKEWILSAAHCFYGTSAYGWSVSLGRQNLQGNNSNEVTKTVATIVLHPGFNIFTLDNDIALLRLSSPVTFSDYVRPVCLAANGSVFNNATRSWVTGWGTVQGGGTLREVEVPVLGNRQCSCLIGVDLVTDNIICAGFPEGGKDACQGDAGGPMVSEQDSIWIQSGIVRFGFGCARPNNPGVHTRVSRYQSWINSHVGSDKPGFVQFNSSGLDADSNFTCPGPPPPATEGPAEAEDLVPSLSSAELCGIPSQRNKIVGGDVVAAGSWPWQASLQTFGSHVCGGTLINSEWVLSAAHCFTSTSTTGWSVSLGRRNLEGIEPNEVALPVAEIIPHPLFDNQIFDNDIALLRLSSAVEFNAYVRPVCLAANTSVFNNGTESWVTGWGSVQQGVPLPSPQTLREVQVPVVGNRQCNCLNGEGVVTDNMICAGFLGGGKDTCQGDSGGPMMSEQDGIWVQSGIVSFGFGCAQQNNPGIYTRVSRYQPWIASRIGSDKPGFVHFDSRGPDADSNYSCPRPPPEDLSSAECAFHCNHNNMTSTREFSVLDMTPKPPSVCVQASPCGTVGEVCGSPALSTKIVGGEDAQEGSWPWQASVQIFATHVCGGSLINREWVMSAAHCFSSTSTFGWEVSLGRQNLLASNPNEVTKTVATIVLHPGFNIFTLDNDISLLRLSSPVTFSDYIRPVCLAANGSVINNGTHSWVTGWGTIQSGVLLPFPATLQEVNVPVVGNRQCNCLNGVGLITDNMICAGFPEGGKDACQGDSGGPMVSQHDDVWIQSGIVSFGFGCAQPNNPGVYVRVSRYQSWINAHIGSDKPGFVRIDSSRRDADSDYTCPGLPPPVTEGPATTTEAVPSISSVGCGIPLPKTKIVRGGAAQEGSWPWQASLQKFGTHVCGGSLINREWILSAAQCFSSTSVLGWSASLGLQNLKGNDSNKVSRNVHAIILHPGFDIFSLDNDIALLRLSSPVTFSEYVRPVCLAANSSVFNNGTRSWVTGWGTVQRRVSPSFRGILQEVQVPVLGNRQCNCLNGVGLVTDNMICAGFLEGGKDTCLGDSGGPMVSEQDSIWIQSGIVSFGFGCARPNNPGVHTRVSRYQSWINSHVGSDKPGFVQFNFSGTDADSNYTCPGLTRPVTEGPLSTFTTTAAVTEDTVSIMSSAELCGNRTVNSKIVGGLNASEGVWPWQASLQIFAAHVCGGSLINREWVMSAAHCFTSASTFGWSVSLGRRNLQGDDSNKVTKTVATIVLHPDFNIFTLDNDIALLRLSSPVTFSDYVRPVCLAANGSVFNNATRSWVTGWGTVQGGVPIPFPGALQEVEVPVLGNRQCSCLIGVDLVTDNIICAGFPDGKKDACLGDSGGPMVSKQDGIWIQSGIIRVGSGCAGPNDPGVNARVSRYQSWISSHVGSDKPGFVHFDSSGPDADSNYSCPLPPPVVVPSAELCGSPALSAKIVGGEDAQEGSWPWQASLQAFGHHICGGSLINREWVMSAAHCFTSTSTFGWSVSLGCQNLQGDNSNEVTKTVATIVLHPNHNRSSHDNDIALLRLSSAVTFTDYIRPVCLAANGSVFNSGTDSWVTGWGRVQEGGGPMVNEQDGVWIQSGIGSFGFGCARPDLPGVYSRVSRYQSWISSHIGSDEPGFVRFNSGGPDVDSNSACPPPAPEVAPSEEPDPISKTPSPTLPSPTGHVCGTAPLNNRVKSDRGLVAGGTWPWLVSLRKNGAYTCAGTLISSSFVLTSAECFSSPAPNASDWTAYLGQKVVNGVEEFQVFSAIVSIAVSELQGSNVAVLQLREDVRFGDYLQPACLDISSAIFFPFGTRCWTAGWGQANEYPSVEKSASLRELETVVVSCGSAASDQENICTSSLDLQQGDVGGPLICKADSSWFLAAVVATSGRPPLADIQVFSKMVRFGCFLRETVGELPSPAAPSSAGAPSSYGRSFLSFSVAWTSVCLALRVCHGAA

>Throm55

MNCHFGDCVLNSAGGPECKCTYPYLGPNCLQQQAVPCSPNPCHNGGVCMEQNRQANCSCRKGYRGRYCQVAPNDCYVGNGKSYVGTVSTTVHSEECLNWHSVSILLNEGRSPFDMYPNFSGLGQNNHCRNPDGENKPWCYVKRAKVEWDYCDVKSCHRVRYCPAKGVPPDLLGYQVMAHPWMVSLQERPKGSNVSFNPICGGALLSSCWVITAAHCIETANEYQVMVGAVKLDSTELTYQLIPVLRTVLYPRPRDVDLALLQLQVTHDQHCARESRFVRTLCLPKQNFPAGKECVVSGWGSDGNSSRSNDLLDAYVLLISEQTCKQPQAYEGLLRDGELCAGNMEGGADSCNGDSGGPLVCQRDGIHYLAGVLSWGDGCGLPNKPGVYVDVYKFVSWIRHEIG

>BPTI2

MLFPRWIWFVASVWMELQVGVLPHLAYSHVGMCPNDMNPNLWVDAMSTCTRECGSDQECESFEKCCQNVCGNRSCVAARYTVGEKGPVGIPKEVTCASFMCTQQGAECDIWEGQPVCKCRDRCEREPHFTCASDGMTYYNKCYMDAEACSKGISLSVVMCRFHLTWLSTSPALPPVTTVHPTTAPLQVTVPPPAAPQAPLILSTPVRQTVNAGDAASFLCDVTGGSPPEITWEKQLSEGVNKVVMGRNHVRGNMVVTNAGQLVIYSAKMHDSGVYICTARNPSGSVQVHHALMVLPVEPSKSPVAMDLTRCSPEECLKPPDNPDLCGSDLEKVSWFYDSLSNNCISFTHCHSNGQQPRKLFETHDKCMQCCGPELSGPCDLPSLQGPCKAYEPRWAYSSSLRQCQSFIYGGCGGNDNNFESKEACEEMCPYPQNHNCKACKPRGKIVTSFCRSDFVVLGRMTELAEEKDSGRALVTVEEILKDEKMGLRFFGKEPLEVNFINMDWNCPCPNITGAAAEGQVIIMGNVSEGMAVLEPHSYVGASSPRRVRKLREVISKNTCDILKAITNSPQ

>Histone6

MTRTQASDSHFADGRSDGIRGPKSDERTLREWCRASPPDPPTPALPPPPLSLPTPPPPEEPAATALARSEPSPVPPSHVTGYRPASPKQASVCPPPASRPAGQLARSLLREAVNHPSSQAPKLTPHAAPPTSPPNNTATMQAAQQQYDFFSDDNAAKWRGLLVPALDKVLKQVHPNLVSQQEALQYIEELILLLLSMLCQAQPRSVQDVEERVQKTFPHPIDKWAIADAQAAIEKRKRRNPLALPVDKIHPLLKEVLGYKVDHQVSLYMAAVLEYISADILKLAGNYVRNIRHYEISRQDIAVAMCADKVLMDMFHQDEDDVPPTDEEPSADEEQSYHELVRSFMADGRHFLRQLELLIKVFREPFSANAMLFSQHDVEGIFSRVADVHEATAKMLGLLEDAVEMTDDGSPHPLVGSCFEDLAEELAFDPYETYAQDVLRSGFQQHFLSQVTKPGAAFHLQSICEGFKEAVQYVLPRLLLTPVYYCLHLFDIIKQLEEKSEDEEDKECLKQAITALLNLQSSVERICSRGLAKRRLRSLIDSPIGYRLGSRIRGRARIGAGIGTELRDSESACRLSGRQMKGKHSAVKKMNEIQKNIDGWEGKDIGQCCNHFIMEGTLTRVGAKHERRIFLFDGLMICCKSNHGPPRLPGASSAAEYRLKEKFFMRKVRINDKDDKEGEYRHAFEIIPKDGNGAVFAAKSAEEKNGWMAALVSLQYRSTLERMLDAAVLREDEDERLRLPPPRLYRFARPDSEENVVFEDGVQSKSGIPAVKAGTVLKLIERLTFHMYADPNFVRTFLTTYRSFCKPQELLDLLMERFDIPEPRPDDADHPDGGEQPLGAELKRFSKEFVQPVQLRVLNVCRHWVEHHFYDFERDVALLGRLEDFIASVRGKAMRKWVESITKIIRRKKQAAVSGPSHSITFQNSPPAVEWHICKPGHPEHFDLMTLHPIEVARQLTLLESDLYSVWTKEDKEIHSPNLLRMIRHTTNLTLWFEKCIVETENLEERVAVVSRIIEILQVFQELNNFNGVLAVVSAMDSSPVYRLDHTFEVPPPARAPPPPSLASERLSRKATPLSALFITRHCHPCCIYLTNILKTEEGNPDFLLRHGKALINFSKRRKVAEITGEIQQYQNQPYCLRVESDIRKFFESLNPTEELCENDFGDRLFNGSLLIEPRGARTLPRFAKRYTCPLKSPGIRPTSARPGAACHPTPLQNEPRKISYSRIPDGEADGGPDAAVSAPGSPCTPLTPPPASGSADGVFDSPRAPGSPAHSGSSSASSVVSFGRLPDDVPPPPPPVPPRRRPGTPKVLASMDGPDVTSAPKQVTGRRAFFRSGLRYLCCLLLLKAGLKKYFVNMDDYLASLGLYRKMVARDASSLFRAVSEQLYYSQNYHHTIRQACADFMRANAYVFEPFVEGSFDKYLERLEDPKVSLVNFQPIRAQYLGHVKTECARRRRFLIYRYPGKAATLISEDDFVDKVTLSCSINGHYDIVYPRSYPASAGLCQAVVYELLYTKVFACEEAELSQAVEAFRVGGRRYRNSLSACSDVDFGYDIPEDRGHREEAESGEEKLRVWSDDVKPASEAPPHGRLSLPYKVLKSLDGDVYRNVEFDVWQDTCKEMQKTDYMVFAGRQYFLGDKCQVRLEPKGKYFNAFIQEVGTHSAAVTVFIEELGEKHLVPVADVKAVNPVPAWNAXPTTTSSSSMATPSPPPDRPDTAPPGGCGRSAARSSSPGRTHRRARLFGFCVRSSSSRLVNRHLIGPQLTYYPPTRRYYDYDGYAFRSRRSRRHLAAALAKGCAYFNEDDAADPLVEMDGAVAYYHLDDAKHEVFAAVPPAAQSASAAAPPPPSVSYRVQGDSGGPVPLTPPAGGAPVCSSEEEQDDANAVGDRVVAPPPYSYDPSGSDLPRGGLRCRHRCSWYHQSCWQQQQQTVAPYAPTYTPDAAYAYQHYPPYLGQDAPPHCESVEAQTDAHGGGPAHPPEGSSLGAPSGAALYPEQVALPSVLHLPYEAPPPTTYLSSAPPPPLGHSAHHPYHTHHACLPASAHWGGHASRVYCPAPNAAHVVGYVAAPPAHFIPPSA

>BPTI6

MAERDVVLGAGLVSSCVARLGRFNTGLQHPYYHVALPGRKLRDISVLSNYVHLQKLELSNNRITDLSCVSHMPFLVILDVSHNEISDFFGFDPPKNLKEVNFSYNILTQMKDMSAYEALVKLELDHNRLKRIHGLEKCCKLTYLSVAHNSITSICSLDDVPLKDLNLRGNHLTSTEGLENLKRIHTLDLSVNRISSLAGLNNLHLLGSMNLERNQEKPEYRLSVVFLIQHLTLLDQEKVTIEEKVLSINKYDPPLDVVAARDHMTQLMYQLMQPQHLYDTTLPSDDAPYPMLVLTGPQGCGKREMAHRLCEDFEDFFAYGVSHTTREPYFGEVNGSDYHFVSEEEFELLVQRGAFLQTVQYGGHKYGLSRDAIEDVAREGLACCVQMELEGVLSLKKSIFEPRYILLIPTIAENYKAMLRSRNLYTDAQIEAALSRIELYDNFNRERPGFFDNVIRCDYWDRAYRMLERVVKNYLLLEDQEEEASGEDKEEEPPTEQQRSDSSAPPDPATSSIDPSEPLDINSCTHGQQQLAPSKTPAELASIRRREQPVREAVVGKSPGVYSWLIRSSDESPGHSADTIKGDKQPHQAPAGITPLSDRRPGSGVKPVLPPIPPERKTPQLLASGVLWRWAKGRDDLRLESREQGHQLGCAGGEVENAIMEKELTYGYTRKAAAVCESRREREKERKRVSEKEADNTETVVQADIVFLVDESWSVGAGGFSAMKDFISEMLSSFKDSPVGAEGVRFGVTVFGDVPRMRIALTDYSSLEEVLGAVRMLPYEGGARKMGAALQFLVHHVFSASISRDHAPKVAILITNGPSDDPFDAAAKEVADNGISLYAVGVRGAEEAELRTTVSQPHEEHLLMGADFPLLEAVLPKLSRRVCFTASEPPRPVKMFQPVKEPILGPRDLRVSELAYGSLRLTWTAATGDVTGYRLTVIPLSSRGQPVNAQQRQIGLKADVTTIVVTELNPKTSYSLTVSAVYPSLIGESATTTAQTTPLPQVLNFRVIEEGLFSLRLGWTPPLKKLDGFRVLIPRPDRPGFVYEQLLPGDASSHVIDSLEEDKKYNISISAVYPQGLSEPVSVVGKTLKLVAIQELLVQNATTDTVQAKWTSVKGATGYRLTWASPEGHIENINLRDTFDFYMIQGLHAGADYTITMNPIFGDIEGPVTSAKVKTLESSAVQTLKVSAVSTSTAVITWNSVPGATGYRLAWGPTIEFVGRDRPRQVALNGTTTEYQLKNVAHDTEYALTLYVLFGSVVGPGITATFRTSPLGYVSSFEVTAYTSTTVEVQWSPIVGATEYKLSWNTDSGSPQSRYLDHSVLSYHITDLSPHTTYTITVRALYGNTEGPEISLTQVTAAALDSEPPQLVKEVRVVDTGVNSVTLSWKQTPGATGYKVSWIHFMGGEEKSHMVPAGVTTFTIQNLHEGSAYKIQVSSMAGSREGSSVLVTARTSDLPKVTGFSAINTTDSSTVLSWNRVAGVSGYRLSWRHISVFETKKETLGLSFTSFKVGDLLYGRTYIFSIRPLYGQVEGPVSTVYQRIVHPTAASPQFTPPRAVTPAPSRAECGRSKVDIVFLVDESSSIGANNFDKMKDFIFRVVTYFPVIGPEGTQIAVVHFSDEPRIEFRLNDHKDRNAVLWALTGLRYGGGNTKTGKGISYVLQEMFQESLGMRQDVPHVLVLITDGRAQDNVEPPSRIARALGVSILAVGVANADLQELRKVAAPGRYKNVFYSPTFDDFPSVEREFIGSVCGEELRSEFKLRLDEELDTPTDDPTEVAKPQAPCRCIKGQKGQRGDGVGGGLGPPGTDGIPGLPGRPGRTGPPGSAGQRGTPGIPGDTGPGGLTGPKGQRGERGEPGYVIAAPDANFVPGQKGEPGSSGPQGPAGIPGLIGVPGLPGPAGPPGISIKGKKGFKGEQGDKGEQGERGPSGPQGPTGPTGPIGLKGDAGPRGAPGDTAMGVIGPTGKKGIRGDVGPIGPVGPLGKKGEQGDKGEKGSPGFGIPGQGGPKGENGERGQDGSKGDKGDAGSPGNPGEAGLRGKDGGVGAKGEQGMKVPYQPHPHKKRKNCNMPKHNIFLNSFCPSQGERGPQGASGDPGQRGPLGLPGQLGAQGEKGNIGSAGLPGLDGAKGDKGEPGEPGEPGLRGEIGLPGPSGPEGKPGQPGSSLTGERGEQGRIGIAGMPGLPGTPVKPGRDGNKGEPGPPGLPGKQVLVTAEGATIDEIRQAFPVPMGPPGATGSPGMKGEKGDIGMKGEKDIEAMFDAYGIKLPLLKVLIDRLLQGGTEELLRTLGSAATTSQSAGTRNEETQTSNAITEYTVCHANSALFKRTLNNLLASFRKSSVKIDLSSRTRLDTFEEFELDQPTRPDFWNESTEGPTLWTPQKQKEEGLDGPSKAKTYPTQVSSTFNDTSNQTVSAQPASDAQAVPLTLPRAGKKGAGHKRQNRERGKKSRQSEEEFYDARHYDEQEERSASNIGGGSRRRAVTDPVVQNPAEGPGPDDEDWSGDWETRGEDEAELARRRQLEEWKRRRADMERTGEGEGGRPSPYHPDYFYFKGQPGEDGMRGPKGDVGEKGQKGEPGIGHRGPEGQAGPPGNKGEPGDAGPPGAQGIQGIRGSSGIQGHPGIKGPPGDPGEPGREGERGRRGKNGTPGGTGPPGAPGPDGSLGVPGLKGEKGDSIPGEAGPRGFTGFPGKRGDQGAPGMKGGPGLIGPKGLRGFKGVKGERGMQGIRGERGSALQVHGPRGLKGAKGDAGERGSPGFDGDKGEKGEDGPPGVKGLKGDAGTKGTLGRFGTRGPGGQKGDPGEPGDPGSMVKTVRNVPYASKMGCQRVVSIFKGRPGNDGVNGTKGDKGDAGRQGQRGNQGGSGETGSPGDQGAKGEKGFRGVPGYFGSPGLNGEKGDGGSPGTPGVPGLNGLAGKKGDKGNAGINGVGGGWGGKGQKGAGGFPGFPGFKGSVGRPGRDGGRGPPGGPGPHGNPGAKGARGRRGKTQPCQRGAPGRSGLRGQSGVLGVGGAKGDKGEPGLSAHEVKKLIIQEVMDKCGLEYKLMVKSVDPDGSSAPAGPHEDDDDDGGGDDAEEHPKRQPGLPANGVGSEERCLEPMSEGSCSDYGLLWYFHARAGECRPFVYGGCGGNGNRFASLLECRACCVLTPERGRGAARRNRVRNLLGKNIYYGLESVKRCLQMAAKRNLCLSEAPQFIFPHQARHGGKKMSEITAKLIERKLLN

>Lectin3

MRATGWLLGQALLTLGAWTASDSYCDHGDVLYGSFCYYFGSWTASWKDAEDYCFNVWKGHLASFHSIEEVNFLKACAKGYWIWAGFRRTNGYNAMFTDGTPSDYLPWGENQPRAWEDASCVRIGSDGRLYYYLCHIDIRFICKKAANRGSNPLKLSEPQSVWTDACGWWAHNPSNDYCYLLMTEYQISWDDAKDYCTRNGGNLLSITDFNEQDFVRDYFQVRANGISLWIGGYSLNAEEKWMWSDGSKLRYIRWSTGEPEDDKPVQRCLSWNLDSDRWKEDTCGSDRGFICKIRKYEPDPTMTSRFGKTYSRKGGEGTSKFDEVLSTKRGTLSTKWGDTTYKAKVGSKRAGPSKGSSFLDTHKRARLGGGGSDDPFGFDSDEESKPVSSRGGGKSSPAKVASAEAPMVEKVGLSPDPRNWSSFREGLHGSVQGISRPGGTERCGPFFNSGHASKGIPESSSPSWEKQHSYAWYQNASESDRKPLAQTATLKTSAKAESTYSSWDALMGFRPPSPSQELRAPALSWTSAVAGDKLPSPPRLQSPVESRDPDGDFGFETADYCQTTSSPSAHVRNSKCRTYRRPGKRNASKAPDCSDTARGPLTSASGGAGAMEATRSSRTRDYTVLHPSSASMCNVTNQAGGVKEFTSEAAPASDKSAAGFSGAAEQLAATRQRKRTEQQHSSQNQSRLKKGTKTDLFGFDEVDACQENDNDGSSKYKMKYFGFDDMSDSEAVGGDDDDGGDRRMKVRARFKAVAEETPPAALEDSHDEPVRNCRALQGRATGRSQNYLHRQDGSLQAESAGQLLDSLPADVLSFSEDDLRVVASGPRRPPQGKPVSKNHDTHRRIFSAQKKSPTKAVYNARHWTLESKEEPIPSFMCRPPATPAPQLSGGASTDVASDAQKDNGVFKAPPPPPKVTKCVHVPSDPYQDTVTTLKCRKEHKELYTVVQHVKHFNDVVEFGENQEFTDDFEYLAAGLKSGQPLNTRCLSVISLAARCAMPSFRMHLRARGKVAQVFKTLNDAPQHHNLAMCTASLMYILSRDRLNMDLDRSCLDLMIRLLELELDQDHGGCDPASQLSAREICKMKEKIRKLCDTVHNKHLDLQNITTGHLAMETLLSLTSKRAGDWFKEELRLLGGLDHVVDKVKECVDNLKTEDDKEKLVSSLWGAERCLRVLDSVTVHNAENQSYLIAYKDSSLIVSCAKALRRCEEMIRRYSREVGGGTQVSADGGSVVAKAVENCMRAIICVLLNLTHDNEWGSTKTGEQDDLIGTSLNCVLRVPQYFPQEKRFDIRVLGLGLLINLVEYSARNRYCLMEMETEGGQGPCDSTVLLLPPHQPDVVIAGGPLGAVAALVQLFLQRERAAVQAEAQTDDLIKEPAKPADKSGEWQETGGEIQWVANDNHCDDAQEKDDKDEKKEEEDRELDLNKALQHAGKHMEDSIVASYTALLLGCLCQGSPMNVTTVRENLPQGDFSIMTEMLKKFLNFMNLTGVSSSCVLESS

>Throm27

MRRAVALVLLTLTFCSSSAGGAIFSGQAGGREQPRRRPRRQTNLSSLWSSGEPASSWDTFEHRNEPDRLWFTVACCDPLAEICQNGGRSVPSLTTGQHMFCLCPNGFEGTFCQTGVKAITAPQGPSLVHQVSGADCYEGVGIYYRGFRSQSESGRRCEPWDADTRLRYLSLDVYGGRHNYCRNVRFKRRPWCHVWKEQYLVWEYCDIPRCASLPPTTQPPRTTPPAVTNTCGRRSRRKQMRIVGGSVAPVESHPWVAAILWRGRSKDVVFRCGGSLISSCWVMTAAHCFPDGSRVNERRFLVSLGKSALNQSDALEQKFRVDKIILHPEFNNELGNYDNDIALLKLKSKKDGRCARDSVGVRRVCLPGEGHHVPVGFPCEIAGYGKEKHGLWYHSQFLQQARVNLLAADVCGADDYYGDKITSNMLCAGRDDWTQDACEGDSGGPLVCQVDGRFVAVGVVSWGEGCARERRPGVYTKVHNYRQWIRQHTGV

>BPTI14

MQSGRSLITRSALLQYSSHVIVEQTFNQWQGPDSFKAHIAPMTYIGHGTYITYAITNLTRIYLEESAPSSIKVAILLFDGISHPRNPDIASAVADAKNQGVRFFTVGITPEAKESANIAQLRLIASSPASRYLHNLQDGGIVEKVVREITTLADEGSLCACEKGERGPSGPAVRQEGPSWGGWNPRTKGSQGAAHPLTSLLFLSVHTESLITVIPIPNLSCSKGEAGLSGLPGREGAEGLPGPPGDIGPEGVTGKKGERGLPGPAGIQGETGIGLPGQKGNVGHQGQPGPVGPVGLGEPGPPGERGLEGPRGPRGLSGFGFKGDKGDVGPSGPPGPIGESGYGLPGPKVKPYLLRLYASQQQAGSIHTLLVERAALFIPTSMCHRVTMEIQALQVHLAQKEKAIPAPWGTLVSEGCLVYRDLLVKDCQDLRVNRESRGDRGSAGERGLKGFKGDMGDPGNAGQPGRSGVKGEAGLTREDVIRLIKEICGCGIKCKERPMELVFVIDSSESVGPENFEIIKDFVNALVDRVTVGRNSTRIGLVLYSLEVRLVFNLARYATKQDIKRAIRSIQYMGEGTYTGTAIRKATQEAFHSSRLGVSKVAIVITDGQADKREPVKLDIAVREAHAANIEMFALGIVNTSDPTQAEFLRELNLIASDPDPEHMFLIDDFNTLPALESKLVSQFCEDENGDLIYNRLTNGNNGHSNSRNGNNGRYAEGVNRYQPAPDWELLNTRQPNNQNHGDSVATFQYDQKGSSTGQQVHAETTHRGTERGDTFNRGTAFQRIPPSREDSSSVNLTLSSSGVSVKSIPTPRPALPKATVPIDPRCALALDQGLCREYTIRWYYDKQANACAQFWFGGCSGNRNRFDTEEECKKTCVLTTSNELQKTIQVIREAFKGKLPEKPKKREGVTKEGSEDEESPFPGFLNDDRRHGMYTRDFILMVNDSAAVAYDVNENECFLAAMSEQVPRNHVSLTSQKNQLYIIGGLFVDDENKDVPLQCYFYMLDPLASDWVALPPMPSPRCLFSIGESDNLLFAVAGKDLQTNESLDTVMCYDIEKMRWSESKKLPLKIHGHAVVSHKGLVYCIGGKTDDNKALGKMFAYNHKQSEWRELAAMKTPRAMFGTVIHDGKIIVAGGVNEGGLTASCETYNFATNKWEPFAEFPQERSSVNLVSTGGSLYALGGFAMIEMENKEVAPTEVTDVWQYENDKKQWSGMLREMRYAAGASCVSMRLNAARMPKL

>Lectin12

MIFNEADQEIRDAGYRQHAFNVLISRRIGPRRQLPDTRDRRCGAEAYPEDLPAASVVICFFNEAISALTRTVRSVLDRTPARLLHEIILVDDHSELAELSEDLERLVREQLPPNVKLLRNRRREGLIRGRMIGAQHATGEVLVFLDSHCEVNRAWLQPLLAAIRAQRHAVVCPVIDIISADTLAYSPSPVVRGGFNWGLHFKWDPVPAAQLAGPRGAIAPIRSPTMAGGLFAMDRKYFGELGQYDAGMDIWGGENLEISFRIWMCGGQLLIIPCSRVGHIFRKRRPYGSPGGHDTMARNSLRLAHVWMDDYKERFLSLRPELRERDFGDVSERLALRERLQCHSFRWYLDNVYPEINNKLQAPPVLGDGKAARPRVLRRGQVTFDPRPPPLRGGRPSQSGFPFQLRNVAGGRCLVAQTRVSQKGGAVVLRPCDPGDPEQEWTYDDEGQLILSGLLCLDVSEVRTSDPPRLMKCHGSGGSQQWSLGKSARVYQVSVGQCLSAAQPPGVKGHVAMAICDASRPQQWLLEKSGKMTFDLPRPQALALVALVTAVGGGRVLSGQRLCLGGARPACYKMAYFHHVSSRVDFREAELACRVDGGHLVSIRTPQEQKHIQTLLEKLRLGSGISDGDFWIGLRRADHDERHPSDGVAPCPQRYRWTDGSTAAFREWHSDEPSCGAESCVAMYHQPGVGGAYLYQWNDDRCSMKHNFICKYQPERRPEEERGTTAAGRGAGPLALYILLPTVPLLLILAAAAARYRLVLGKRSAICSGAERHPCGSSHSPAVRRGHLWISETHKGGGAEP

>Throm7

MKKRVRFRSGWWGAEGVSPHSLIASPEGTGDSAMRREGCQVYCVFGQGRSGSSGVPKEEGVLKEPSQLPSSPTKTKEEPGPPPEFGCRRRWAFGLLPVLSCVAAMALAAGYLTSAPPSSVFFLGCSVEFPNLSFSAELADCSSPPFHLRARSLKPYFSELYKSSPWSSYYLRSGIAAFSEGTEGLNVFYWSQFSAPGHIATAIQLSSPERLQRLLPGSNKVVRDGRREQRYYLKQDDVTLHLLGLDPDDWEEKSDKFMNPTGLQSGKWQFGFQAVSFDLYAKHGVNRTLVLVSPKKLYYQWRLRVPSGHAVRLVVLALHGGAAAASPSCSAHMMSAYDFLLPLQNKIIARWCGPHPTPGSSPTMRLTSSGNVMLLTFSFSRQREGAVFKAYFQAVPKAGCGGSLFSWNGSVSSPYHPYHYPPNIDCRWTLRAPLPGYLIAVTIVTVDIQQSSDGCEKDWLDVGGVKFCNQVSDDGRSRVYSSPLSIHFHSDEAVTHRGFYLRHRAFWPQGSCPPRHFSCSDDRCIPLRRVCDGVRDCEDGRDEAKCSSCRPGQVLCANGRCHAQSSQCGVCADSNEESGCRSKCYHTCPNKLCLPKSSVCDGVVDCQDSSDEMNCTRAYVKACSSSLYKCANGKCVTKLNPECDGFKDCQDGSDELRCGCGIRPRKRPKIVGGTDASPGSWPWQVSLQMERYGHICGATLLATRWLVSAAHCFQDSDSIKYSDPRSWRAYMGTRLMAAGKSALSIASRPVRRIVPHPKYEQLTSDYDIALVELAAPVFFNELVQPVCVPAPSHAFAIGTSCFVTGWGVLAEDGELASRLQEASVRIINRNTCNKFYDDAVTPRMLCAGNLQGGVDACQGDSGGPLVCQERNRRWFLAGIVSWGEGCARQNRPGVYTQVVKFADWIHQQTRGADVEGNGKYGVPVLEGTQGQLVLELNQTLELSCRGRWELSWSFPPNLPHDRIRVEESRCGKSQRLHCSRVTLLGGARPHHTGSFLCRYRHRVERQASVYVYVADSQQPFVDHPEMSPDVLYMKEKQPLVIPCRVTHPNVTSTLVKFPNHSLSPDQRNIVWSTRRGFTIRTPTFFYIGLFFCQTLVDGVAHKSRIYIVHRPVSNILDVYLNSSGPVRALKGERLVLNCTATGELNTRVNISWDYPGKMSNSGSTSKRLLKHRTHMLFYNILTIPKLRRSDRGLYTCRVSSGNKTKQQKVTVTVYDRPFIRLKPRRGSVMEVQAGQKAYRISPKLRAFPAPEVIWLKDGMLAAEQCSRYHLDGHSLVIRDVAEEDAGKYTVRVRIQEHGLYQNLTLILVVNVSPQIAEKAVSSQSAAAVPRGSRQSVHCTSHGVPAPHVHWLWHPCPPKGLRCTEGRDYRNVDAGFVRLSLAAFIPGCTASLTASQSRPGVLNGPGRVRRAQELLSFGLGSPLVAGSAGAAPNHGNRSASYQARAGGALGFLFENQSAFPLVVWLPLPTCAPPTSSSGWTPLNEGLPATSTHNHILSMTRNQEVLQGKTKTVGVVTVARAVVSGVFRCVASNSVGLDHLDINFFVTDIPGGFAVSQHEDPREGGELHLTCSANKHLYSSLSWRRLHPAGGGGGTGWGGHTFLHGHSSMGDFSRSLRLSLSNLSTQDSGAYMCAAHHRLTGQEAHLVVQVRVSVLEPAVLLNNLTDWTVNVSSSVTLRCPTKGVPRPTVTWYKNKRLLSAGSGIVVSPEEDSLHIDRITVEDQGLYTCRASNQKGSAESSAYIWVNGAAESPSMEIPTLACTCVVATFLWILLTLLIRKLRQPDSSQNKAEYLSIILDSGDSPAEQRCERLQYDPKRWEFPRERLKLGKNLGRGAFGKVVQASAFGMGNASGHRTVAVKMLKEGATVSEHKALMTELKILNHIGHHLNVVNLLGACTKPGGPLMVIVEYCRHGNLSSFLKSKRGVFGVSRRKSGETSCDIQPARNNTAGDADTNSPLFLEDLISFSFQVARGMDFLASRKCIHRDLAARNVLLSDNKVVKICDFGLARDIYKDPDYVRKGDARLPLKWMSPESIFDKVFTTQSDVWAFGVLLWEIFSLGASPYPGLHIDEEFCRRLKAGTRMRAPEYSTADIYSTMLACWKAEPTERPSFSDLAHTLGDLLESTVHRDGKDYIPLGSHVESWKSSKTFREKILAVTNLSYMHAVLGTLDMFEEMPCEDASHGSEEGDSGMVLPSEELQRLSWRHDMKEGSRLHGNGPSEWDSDQGGCSASDYNWTFFYPSL

>Throm26

MGPECKESGDVDASQQDAVSFTDVSVEVSTVDHTLQKLCRMYRKTRRKPKGLSRLWAYLLSIRHLAVIITAVVFVLVVVLWSLLWVFIFRREGNSVVYFAGMFRVANVEFIPEYRQAGSHEFVSMATRIQHVVSGVYKMSSVTRLYKHAIISDLSTNNKGGVLVHFWMVFEVPRLKSAAVCEECVAVILRDSVHTSLKNRSSVGYLLDLPVDIDSILVNAVRRSDYTSNAAGSQCIDKLYASLPGARVPLNVFSSWGGVRCHVKLTAVPGFLIRLTVASFQIEPSDCVHDALTVYDALLPMRGRVLHRLCAPLSSSISLVSTSNVMLLSFTMSSGTKSFRGHFEAIAEELCVSRITAQPHATGQVYSPFHPSLMPPHCSCTWIFQTPDAALGVALQLQNYVLKPKDMKGYCGSYVGYESIFRIGECAAKVEFRCTSRSATQPFHVTYSSYNISQPCPESHFLCSTGLCVEKSRRCDGLDDCQDESDEVFCSRPTKNCGGSSPLHPLFVCNGETDCANGLDETNCTQETSCSAVSYHCNSGSCIRKKNARCDDIADCQDGSDELDCACGLPNHSVAHRIVGGTDSEEGEWPWQVSLHFSGHLICGASVLSPDWLVSAAHCFSKDRLSDPRRWRAHLGALTQGSAGHVAEIQRIVVHEYYDARTLDYDVALLRLKRTWPPSLGPLVQPVCLPAASHAVTARHRCWVTGWGHRFEEDKSLPSVLQKAQVSVMSQTECKKTYGPVSPRMLCAGVPSGERDACRGDSGGPLSCQAPGGGRWFLIGIVSWGAGCGRPGLPGVYVRVTKFTSWIYAHIS

>BPTI3

MGWWPLVLRLVFLACAFSQETGEECLAKFKKGREDFVLDVDESVKDGAIFISSPKLDRSRDCVAACCKEEKCNVAFMQGGAEENSIKSCFLFNCLYKKKYACRFVRKKGYYNYILDSVYESYLEVDLPPKETDRPPVANGGPDRVVQPQDSVTLNGLESKDDEGIVSYLWQMLTEYPYAVIEKTGFNDQIIVSNLTSGMYKFQLTVTDTIGQSDSTKVTVLVLTPEQSEHHCMAPKKIGPCRGAFPRWHYNAASQKCEGFVFGGCRENLNNYLSKDECTNACYGTEKRGSGRGLPVPDQREKCGVACTPEQFTCANGCCLEPGLECDSTAQCSDGSDEQKCKDLDNTFQILLKIPVDEQKVRCTEPPQTGSCRDTLSKWYYNPLQQDCSRFNYGGCQGNENRFDSKAGCLKVCRGVTGVIAIAAVLAIAIVIILGILLYCFMKGKKKSSQHHRVPVNNAPLTSIEDREHLVYNSTTKPI

>Ubiq19

MGGCVGRYWERWDDTQSRGSSRNGGRGARNEPLKKDRPKWKSDYPMTEGQLRSKRDEFWDTAPAFDGRKEIWDALKAAAVALECNDHELAQAIVDGASITLPHGTLTECYDELGNRYQLPVYCLAPPVNLISERSDDDHSDSPQLSVAPKKEFQLKVRLSTGKDLRLSASMTDTIGQLKKQLHAQQDIEAAHQRWFFSGKLLTDKTRLQDTKIQKDFVIQVIVNPQAQPKMSPTTTTPTPTPTAATTSSSSSPESE

>Throm46

MCRGCCEYDVIRCKCPLKGTSVGYAVPCCRNAIDECDPCIIHPGCSLFENCKRCNNGTWGPKDDFFIRGQYCAECRPGWSGGDCMKCGGVIRKRQGHIVLESYPNNARCEWTIQVDQPFTIELRFMMLSLEVHHSCRYDFVEVRDGDSINSRVIGRFCGNNRPAPIRSSGNSLHIHFESDGYKNYDGFFATFQESSVPVDRLCALPPKPRHGDHFLVYGPNDVLIALQYLCHQPFELSGVSQRTCLPNNTWSGTQPVCTQVNNTLTEAEKDKETAKETAKGTDTAKEDIDSEKEDSKSKEKNQENTGVKKESDHWSTTVDNTTAGTIPGKTLAEDRNEDEKSIGREKTTSGGGDKMGNVGPDNGLDTAENKEADIVKPGKKDDRQSSKSETATTDVVVIKDKGQGEKERKEEQVNGKKDSGKVGPNDTKLRTVVSVDINAVEISEQEMAKNNTVTDDLNDLSKNNTTALPGRKIIPSRVNITQYTLYRAGGEESGMPKESPTIKEDKAETSPTQKPKEPDTDRKAKEKEKDVNQSFFEGSCPPPPRLYHGYHVMMPGAKPETVEFSCNHSYALSGDPLRSCQPDGTWSGKQPQCVRACREPKVSELVRQRILPPQAPFRKTPVHKLYSTAVGRQIQSDAPTKGPPVPSQLAAGFHHLYTHIEYDCVSAFYHHNGSPRRTCLKTGKWSGRHVSCSPVCGKHPTFDPERPGEAHWPWLVAIYRRSTNGAETKLTKADSHTGSVKKDGSARASNHIRASVWQLVCSGALVNQRSVVVAAHCVTQLGKVYPLDTAKIKVVVGKHYRDDHRENKGLQHLRVATIVIHPNYDPHILDSDIAVIKLLDKAKIGEKVQPLCLSDIPGEGEMSGQGLVTGWSPLPDSGLGAEEKARVGLVHLADIVPCEQQYARNGVPVSVTDNMLCASQKPNYGPSTICPSDTGGILILPALTDNKENPSVGFTPKGNKKRLWRLLGLVSFGYDQGECDPELYTVYTHVANFKIWIESSMK

>Throm44

ATDPDNFLGPEECLSRKPTRASCDLVFCAPWERCIEGRCSCKPPYLCPSEDVTPVCGRDGRLYRSYCQAMALSCRGGKSAMSHFDQTCSEEQPKFRSFLDPRTGALSLFVPDRASVGGGKRLLVCGQRWDAAAANVACKDLGHPLGAASAGSLASGDLRRGHAPSPLPDRCVVVRCRGFETSLAECVIHDGVRVGEGRVATATCYDHKEVPRECGFACANGKCVSANRTCDGIDDCGDRSDEMCCKRCRRGAFRCDSGVCVQADALADGQRDCLAGEDEAPTHASALARLPFEILNWDKIEPKLHLVQRSAAQDERAVIHLSCRAEPAGTEMNGTEYVSPRKETRARRAHLESKLQCGIANMTAALDDSVGDRAGRVKRVVGGVAANRTQIQWQVGLEDDGRINCGGTYIGGCWVVTAAHCVRPNPSAYRVKFSIWKKSTPQDTTDIIRVGKVIIHPRYNASTYENDIALVRLKELPEAPGKCMVDNPAVKPACVPWSPRLFGANHTCSISGWGRTSVGKGSQVLQWANVSLIHDCQRFYKDRFRPGMMCAGDLDGNVDSCQGDSGGPLVCQDHLGVSYLWGIVSWGDQCGQPGLPGVYTQVAHYFEWIRLQTGWPAVTKFNS

>Chem3

MELRALLAFGSLLAVGGGLPPISRDYNTRCGCLRTETRIIPPDSLRSIKLVPEGPHCTQTEIIATLVSGERVCLDPRSWWVKKLVHFVLEKQLHRRAAVISRRPA

>Histone4

MRACLPVCSHPQAGGKAGKDSGKTKTKAISRSQRAGLQFPVGRIHRHLKSRTTSHGRVGATAAVYSAAILEYLTAEVLELAGNASKDLKVKRITPRHLQLAIRGDEELDSLIKATIAGGGVIPHIHKSLIGKKGQQKTV

>Lectin7

MLKSKGQSLAWLPLLLLEMTCNASLVKVNKALKVRRGQSSYLQEGDLHFLLPLPKDACQVEVVLNEPITQRVGKFLPQVFNCHYRENEVKYVHNGSPLLMEDTVKLRLYRFTETDTYTEVFSLHVDIVEPDCSIIKLGPQFLQVPFYGISADVDGNVLSLHYEKRSSLECSIHLNSHDTNLPAHGQLVTGKPKEAVKRGDEPLRYIEDPVRCNSDVCLKGVKLVTFLKVPCEDFLLMGLKYQHMTPPSPDIDYIAIRLDLKDTRSGNLYQAMGHYSMEIDVHDFFFEKSPPMTVQMSVRNADTNAPRVSWNLGLSLLEGQSRPITWEQLQIVDNNDIGAVRIITVKGLLHGRLTVRGGKGFMFTVRDIKAGIVCYHHDDSDSTIDFIIFRITDGHQQTRHKFPIKILPKDDSPPFLIANMLLEVSQGQTTLLKGTALQASDVDSSSYVVSKFLQRDLSQSMVYYRHKGNEVLDDSFEFVLSDFQDPPNLSEPQVVMLHVEPVPRQAPREVPTARRCLVVKETDVVHVTQQHLHFVDKDSPDSDLTYTVTTPPFDRDRYSSEDAGRLFLVDSIPTFTKDTNAPVLRLFTQHAVNFMKVAYMPPTADIGPFPRYIQFGLSVTNQHGNTLDGICFNITVIPVDNQPPQVITKPLMVDEGGECLLGSEHLQLSDADSVEEALQLELLGQPQHGALQLNNFPLKPGQTFTVQDLTSHRVRYRHDNSETLDDRINLAATDGANEISFVLQVDVSPINDEVPVLVAGLKPVLPCAEGQTTIISAEYISAIDADTENNSLAFLIARQPRHGLVLRNDIAVDHFVQADVRAGMISYKHTGQEIGLTPHGDMITFVISDGETEKSVLCCGGRKPKIGSRETSLPVYDLRITISPVNSQPPSLKTGDIFVVDEGGRAQVNASHLKVSDADTVVDELVVSLVTPPRFGYLENVLPRPGFEKSNVGVSIDSCSYRDIIAGQINYVQSRHQKLEPTSDQLILRVSDGKFSSARVTFNIVVSPTNDELPELVAQNMTVQEGHKRNLDLSVLNATDMDIPKDVLRFHVVKPPRYGNIIRHLGTDTHRGANSESSVADFTMAEVTNGHAVAYVHDDSEHLEDDFTIQLMDGRHKIQRQVLVEVLAVNDEKPHLIRNNGLELEPGEARLISNVALFAQDHDTPSSQVIYIFERVPAQGLLQLKRVQNWLLLTVGENCTQEMVDTNRLRYVHKDPFGMTTQDLFVFHLHDGQNQSPSQQFIISIKHVEKGTIAMFVTPVSVNRGGRVVLTTDVLFATDHADRPDELLFVVTAPAAHGYLENTKHPGVSISAFSQMDIAANCVAYVHNNQASTPTETIHFVVSNNQTSRKGILELSVKMLDQIPPSLTNKGLRVPQGLAMTLSRDCLAMTDPDTPRGALVFKLRQPPRHGELLLRGTALMTGSTFTQQHIEELEVTYKHNGAQSLIDRFEFTATDSTNRGVRLDGKLHTEPLSFIIQIKPLERSHPEVVKLLPLWKAELLGGFARQRVSQFDLNRRTIVYIVNPEMESLSDSLEFRVSDRHGNAGPSHILQLSWSSVELSQPEYCANEEEKRVSVEIIRKGNVAESSYVKVKVNEGTATAGKDFLSSSSSLLQFDPGVSLKIWNIEVIQDQLEEADEVFEVLLVFPEGTVIASVKKAQVTIKDTGRGQCKQNQRNAASLLTGKSGPSDTRNQNSSGRLKKPSLASQSPIHQEGAFSRKNKSKSMAKTPPCPPGWTFHSGRCYILNKRHKTSWSRANRSCKESYKGNLASVFSKGDMDWLWDFGGRKPFWIGLNDRDAQGSWRWEDGESVTYMNWNRAPSPSIAEGNKMCALVWRRAKWQRRDCKTGRGHGYICSIKI

>Chem9

MRSIRVFLLCALAAATISAVVGNNSSGPEECCFRHYPRRLNPKIFESYFMTDSRCPLMGVILVTKKNNHICANPTLSWVKKVLKSLDEAIL

>CDK-like2

MDVSSNPKGCPASGTRVGDPRMVDIRMGEREPPLRMSPFRMLRVLDSCRPPGSRIGIVHENVKMGSDGESDQASGTSSDEVQSPVRVRMRNNHHRRISNEDINKRLSLPADIRLPEGYLEKFAMNSPPFDKPMSRRLRRASLSEIGFGKLETYIKLDKLGEGTYATVFKGRSKLTDNLVALKEIRLEHEEGAPCTAIREVSLLKDLKHANIVTLHDIIHTDKCLTLVFEYLEKDLKQYMDDCGSIMSVHNVKIFLFQLLRGLAYCHRRKVLHRDLKPQNLLINEKGELKLADFGLARAKSVPTKTYSNEVVTLWYRPPDVLLGSTEGVGCIFYEMITGRPLFPGSTVEDELHLIFRILGTPTEETWPGISTSEEFKTHNFPKYNAEPLVNHAPRIDNDGHDLLSVLLQFEAKKRVSAEDALRHAYFRTFGEQVQTLADTASIFSIKGIQLQKDPGKRSSAYQESSKSLEMAN

>BPTI8

MEVCKLRGLAWILLLLWSLPHWTTAQRRNGRRNNYKLYEDGPNGGRPCSLEVVFILDSSESAKISLFEKQKAFVLGFSTRLSMLQVAGWTLSVRMAALQYSSSVSMEQRFSAWTDLDAFHGRVSVMSYIGHGTYTTYAIGNATELLVKETPDDSVRVAVLMTDGADHPRNPDVLAAAIKAKEHGVKMFAVGLSHIAQQGANSAKLRAVASAPAQQFVQSLQDPQLEQRLLKEMGDRGECGAPGVKGDQEWKQNLSVFTSALMECEYRLLVNPHPKAGNPDSQDIFLGLCVAGLAQRPGRWLVRIKSILLWKAMEHESREREKKATREGLDQLVSLVSENRDYQEIEDLKDPGAIVGYLASGSKVTRVIKAHLDPQASEEVRAWDLRALRGTWGLRDYQAYLVSREKTEPRDKRESEGIEGPEVSQVLLVLWDQWEPRAIRDLSGHPVHLVNLGGGYLVRRCRCIKQYLFFSVQGDPGPQGLAGAVGEPGRGITGPKGPPGVPGLPGETGADGVGLPGAKGSIGIIGPPGPQGSPGEGIQGQKGDRGSAGEKGKKGDRGAIGEPGLTGPLGRVGQKGEPGLTREEVIKIVRSMCNCGVTCRQSPLELVFVIDSSESVGPDNFEVVKDFVSALVDRASVSRETTRVGVVLYSHINVVVVSLSQDASRDQVKAAVRSMTYLGEGTFTGSAIHQANRLFRVARPGVRKVAVVITDGQADKRDSVSLENVVREARVGDIEMFVIGVVNASDPFFSEFKKELDLMASEPDADHVYLIDDFKTLPALEKKLLSHICEDSSSLLFRSIPGGGLPPSRPEVSGSDIRLPQQTRTDMDTPTFSGDFRRIQVTPRPPPYDPDRQTTTPRPKTEDWSSSEAQRLPFLDRQSLRPDGRAPTRHTVKVAPTTPTVKVPVGAPQITPELCRAPLDPGPCRDYVVRWYYDSEANACAQFWFGGCQGNANQFDNERRCKQTCVRV

>Antipro2

MHTALRVWILSVVVCLGRGHHHLGHTDDDSLSQVSSGNKEFAFHLYRRLAAHPDNQGKNIFYSPSSVSVALAALSVGARGDTHEQLLSGLGYNSSEVTQSAINQAFSMLLKRQDNSSDISEGTAVFLDNTFEPNPEFLKDLKEAFHTDVSNVNFHQTTESADTINKYVSDKTHGKIDKLVEDLDPGTIMYLISYIYFKGMWDSPFEAKLTKEDTFMVDENTEVPVQMMNKEDGFDTYRDMAINTTILRLPFNSSYSMFLLLPENMAVLEKEICPQHLTKWSKWMKKRTYDIFVPKISMKTEYSLKELLTEMGMTNMFDVRADLSGISEGKKLLVSGVVHQAALDIDEAGATAAASTGIRITLMSLQHVPVLKFNRPFMLLITENTTDDILFMGKIMNPKI

>Ubiq21

MSRLRGNLAARHSKSQGTLRHLPGSNTPPLKCSSQEDHAVTFGFVKKSQSCRLALFKLSSRSLTRNGPEFEARIRQNEINNPKFNFLNPNDPYHAYYRHKVTEFKEGKALEPSAAVPKVMQQTMQQTQQLPQKVQVIQEAIIPKEPPPEFEFIADPPSIAAFDLDVVKLTAQFVARNGRQFLTQLMQKEQRNYQFDFLRPQHSLFNYFTKLVEQYTKILIPPKGLLVKLKKESENPKEVMDQVRYRVEWAKYQERERKKEEEEREKERVAYAQIDWHDFVVVETVDFQPNEQGHFPPPTTPEELGARILIQERYEKYGESEEVEMDVESDDENDEREVRNAGHAVQPDQDTQVQDMDEGSDEEDEGMKIPAPPSNPMPPPLPPTPDQVIIRKDYDPKASKAPAPAAVSDEYLISPITGEKIPASKIPEHMRIGLLDPRWLEQRDRSIRDRQTEDEVYAPGMDIDSSLKQLAERRTDIFGVEETAIGKKIGEEEIQKPEEKVTWDGHSGSMARTQQAAQANITLQEQIEAIHKAKGLVGEDDTKEKIGPSKPSDTHHMPPMPSSAPIVPKPSPPVAAMPRPPSNVGPPVRTTLLSAMPVIPRPPVAPVVRLAPGQVLTSMPPMIPGPRINVVPMPPSAPHMMAPRPPPMVVPATFVPAPPVPQPPNIVPAPPSHPPSHHDDEPMNKKMKTEDNLIPEDEFIRRHKGPVAVKVQVPNMQDKTEWKLNGQVLNFTVPLTDQVSVIKVKIHEATGMPAGKQKLQYEGIFIKDSNSMAYYNMSTGSIIHLALKERASLTLANFASLLAVDANMANTENGRRSVDTRSIRTLSSSHIDDESSLGSDSEINGFTSDKQTDKYGFIGGAQQYSEESAQDVPTAVLRQREVKWLDMLNHWDKWMIKRFNKVRLRSQKGIPPSLRGRAWLYLSGGKGKKEQNQGKFQELDSQPGDPKWSDVIEKDLHRQFPFHEMFVSRGGHGQKDLFRVLKAYTLYRPEEGYCQAQAPIAAVLLMHMPAEDAFWGLVQICEKYLPGYYSPGLEAIQLDGEILFALLRRVSPLAFRHLEKHKIDPILYMTEWFMCAFSRTLPWASVLRVWDMFLCDGVKIIFRVGLVLLKCMLGSREKLKACQGQYETMELLRAVEPRYMQEGFLVREILEVPITARDVEREHHTQLKRWRKSHGELNFKSPPRMHGARLIMLADPPRRQDLQQHPTILLEEAELSPLLKKGKEHKSRKKTLKKSQFPEVIPNPYPLPNATPPPPSYPPAPPPLLSKAPETVPQTQADPIHQGHAPSADLPPAKEAPLQQSTHSLSSTDQDTYL

>Throm29

MFCFGELSLGVLFLHLATAHVFLGTEEASQVLIRPRRANKFFEELKQGDMERECREERCSWEEAREIFEDAAKTDKFWATYFDGDACLSTPCAHRGRCEDGIGSYTCYCYEGYQGFNCEIAIPQLCENQNGGCQHFCKVEQGNIECSCADGYFLANDDKSCESNEPFKCGVTITGKTRTIFRYQRPNITEGNVTEFNQTHVESNDAAAASLPESSPHGNLTSEEIVQESVEEDEVFSDVVGMTRIVNGENCPSEECPWQALLLDENDKGFCGGTILNEYVILTAAHCMNHTSYISVRLGEFDLLADDGNEATYYLETALTHKLYRSDTFHNDIALLKLASPIKFSRHILPACLPEPDFAEKVLMKQQDGMVSGFGRLGQNLPASTILQRLSVPYVPQQTCKESSSFRISAHMFCAGYDKKAMDSCRGDSGGPHVTAYKNTHFITGIVSWGEGCAKEGKYGVYTQVSKFIKWIRTAMKVLLRKEKSGGRARRQGQPFRRMYF

>Throm51

MMRSCIVLMVFGTATAAVFVEKQEADAVLSRWRRANSGFLEELKQGNLERECREEICDLEEAREVFEDDALTRQFWDTYERRDPCRINPCRNNGVCVTVESGFQCQCAEGFEGHFCQTGEQNLDADDGTEQRILVSIVITHEDYEPATGDNDIALLHLSGGVSLSRYAIPICLPTKDLAERELLLEPTHVVSGWGRRTSGGNEDVYSSTTASGEVLAFPVLRSKSVPIIEHFVCSQIVGFNITERILCAGYPAGLRESCLGDDGSPLTTLYGSTHFLTGVVAWGRGCSVRGYFGVYTNVAKFVDWVESKNLLLTAMSPDMLEEKDQHSPRQRFRL

>Throm41

MRTWNLILGIKPLDIMLARSCIVLILLIDIAAAAVFVKKQEADAVLSRWRRANSGFLEELKQGNLERECREEICDHEEAREVFEDDALTKQFWDTYERRDPCRINPCLNNGVCVTVWSGFQCQCAEGFEGRFCQRVFEDSLKCNFHNGQCEHFCDGSGKRRKCSCADGYILGEDGRQCIAQGEYNLDADDGMEQRIPVSTVIVHKDYEPMTGHSDIALLHLSRGVSLNSHVLPICLPTRDLAERELLLLRYHTVSGWGKRISGGNDDGTVSAGVPISPILRKMSVPIIQNSLCSERGRYNFTDNMLCAGYLEGQQESCRGDDGSPLTTLYGSTHFLTGVVAWGRGCSVRGYFGVYTNVAKFVDWVNSENLPLTTMSPGMLQQKVTEH

>Throm14

RVVNGEICPKGHCPWQALLTENHQQICGGIVLSDQWILTAAHCVWKKQNSILHVVVGEHDLETDEKTEQKRRVSKVLIHRSYNHSSYDSDLAVLKLHRPIKLGLYVVPICLPAKNSSFTRTLAAIRHNTVSGWGRLSLHGSTARLLQRLALPRVPLQECRLHTKLNITRNMLCAGLQRGGQDACKGDSGGPLVTRYKKTWFLSGVVSWGNGCADNNMYGIYTKVSNFLDWIQHQMTRCCESSPCLNGGSCLVLPASYNCLCPLPFSGLNCELDDHVRPETCLLENGGCEHFCDEDERGERVNCSCADGYFLNADGQSCMPKDSITCGMTPVLQDPNKAKELDPRARIVGGQECPKGECPWQVLLLYHGKGFCGGIIIKPMWILTASHCLEDADVQFLNVVAGEHNTMVEEGTEQVIQVAQILMHERYEKKTADNDIALLRLASPVVYTPYAVPACLPTKSLAERDLWAVSIHTVSGWGRRAENGPTSHLLRRLRVPRIRTQQCIQESGVQLTENMFCAGYLTGREDSCKGDSGGPLVTQYKKTTFLLGIVSWGKGCARPGHYGIYARVSNYLQWIHNHTATPVQLTNDTQLPPHNLTT

>Throm63

MPNIVLFSGSSHHDLSQKVADRLGLDLGKVITKKFSNQETCVEIGESVRGEDVYIVQSGCGEINDNLMELLIMINACKIASSSRVTAVIPCFPYARQDKKDKSRAPISAKLVANMLSVAGADHIITMDLHASQIQGFFDIAVDNLYAEPAVLQWIRENIPEWKNCIIVSPDAGGAKRVTSIADRLNVDFALIHKERKKANEVDRMVLVGDVKDRVAILVDDMADTCGTICHAADKLIDAGAIKVYAILTHGIFSGPAISRINNAPFEAVVVTNTIPQEEKMKACPKIQVIDISMILAEAIRRTHNGESVSYLFSHVPLPAKMSDKPDISEVTSFDKNKLKKTETREKNVLPTKETIEQEKAATTS

>Lectin20

MALALRSVFLLCGLLAGVLICQLKGGNLASILDATENALVIELIREALNGLRDTWIGLHDGVQEGTNLWTDGSVVDFTAYTSPQPDNFQGLEDCTEIDDGIPSCPKGWVRLEDRCFYVVDQQRDFGDAELACQALGGNLASILNAREHVVVRALIDLRIAVHQVAWIGYNDIDNEGTFVWTDNSPTGFDAFNGPDPTNPNEDCVQTNAGTNSMFWEDHICTTTVPFVCVTNVH

>Lectin23

YIKTARRRRQHQASHTATKMEFALRSLFLLCGISGLLTGVWSKPTVIVKGNNCPIGWTQLDCYCYIYEDEGRSFADAESVCNILGGNLVSIHSALENAFILELIRAGDNDDEAWIGLHDAIENDDFVWTDGSDTQFTNFDVSGSEPDDTGDCVHMDESAGLWEDALCSDEIPYVCIRDMLHH

>Amyloid2

MGDRVALMLLAIAAVTLAAEVPTDGLTGLLAEPQVAMFCGKLNMHVNVQNGKWEPDPTGTKSCVGTKEGILQYCQEVYPELQITNVVEANQPVSIQNWCKKDRKQCHSHMHIVVPYRCLDRTMNLHDYGMLLPCGIDRFRGVEFVCCPTEAERDADSAEQDGDDSDVWWGGAETDYSDNSMVREPEPAEQQEETKQSVAEEETAEGNDEAEDALDNDQDGDGEEDEEEEEDMAADTLDDDDDDDDDDEAATNVAMTTTTTTTTESVEEVVRDVCWASAETGPCRAMLPRWYFDRQEGRCAQFIYGGCGGNRNNFESEEYCLSVCRSVMPTATPGSPDAVDHYLETPADENEHTHFQKAKESLEAKHRERMSQVMREWEEAEREAKNLPRADKKAVIQRFQEKVETLELEAASERQQLVETHMARVEALHNDRRRLALESYLTALQQDPPRPRHVFSLLKKYVRAEQKDRQHTLKHFEHVRMVDPKKAAQIRPQVLTHLRVIEERMNQSLGLLYKVPGVADDIQDQVELLQREQTEMAQQLANLQTDVRVSYGNDALMPDQELGDGQTELLTQEDTLGLGGVGFIHPESFNQPNTDNQVEPVDSRPSLERGVPTRPVTGIKMEAIPELRMEKGDRQSTEYEVHHQKLVFFAEDVGSNKGAIIGLMVGGVVIATVIVITLVMLRKKQYTSIHHGVIEVDAAVTPEERHLSKMQQNGYENPTYKFFEQMQN

>Throm9

AYLIQEQHDFMETVELKGLKHDPSLQDPNSGFSLVLTSVLKSKIKNVFASSSISHHYVDCNIIAFGNMNGDVMATFRLVFAVSKFQQYSENFIQELLRAGLTSVMHGNPLEVPQFGHINAIVLLGASGKSFYNIGDDTLGEESCPESAFTCDNGECITKLNPECDFVSDCADGSDEARCACGTRSATGNRIVGGEDARQGELPWQVSLRLHGQHTCGASIISERWLVSAAHCFETANNPKQWTALVGASLVSGEESESKTINIKTLAVSPDYNPTTTDVDVAVLELESPLTFGPYVQPACLPAPSHVFAPGQKCVVSGWGSLHQFSSEMPSTLQKALVKIIDSKVCSKLPVYRGSITSNMMCAGFLQGKVDSCQGDSGGPLVCEGAPGRFFLAGVVSWGIGCAQIYRPGVYSRVTKLRSWILSHVDPSSKHYYPNHEITLPVTAPGASASNSPFPKIVAMDVESAPAPPVSNCSGNFQCSATSCINKLNPECDGLPDCPNQADERNCDCGVRPALGGNRIVGGVTARRGEWPWIGSLHYQRLHHCGATLVHSKWLLTAAHCFKSDPSPTHWAVSLGSVLRSGVGAVVIPIQRVVIHPAFNGTSMDHDVALLELLIPAPMSHTIQSVCLPSPAHRFLEDAECYISGWGSMREGGSLTNLLQKAPVSTIEQADCRQSYGDNLTPHMMCAGYMEGGRDTCLGDSGGPLACREGSGQWFVAGVTSWGHGCGRVGFPGIYTRVTSVRNWISSYLPF

>CDK-like17

MPGSDRHHGTKRKPESIIGGLAHAAFQTAAGDMTSKDGKLHCKSLMASSKRRRHKSAKVRESPVVPPTIPEYISLSRTAPPSVLMKPLVEYDDISSDSDTFLDPPSTRLPERVVVMERLEPSPEFIREEPIRESKSHRHSRKKSKDPNKVRDPGNGARGYKKKSSKDRDKSASGVGKEKLLSSGPSKRHIQQELDFKRGDPMFSQMHTMVSIGSTSASSSRSKESGRSGKSRKDKQQRREARLEGAHSDSQKVRGERSHRKSSKSNKSSPKGKSSRGSPRRKAATAALSPSPRRGESPLGAPMLDDSYSRRKATQQCPSPYGHCRQRSDSPYGSRHRSSSFERDSSPYSRRRSISPYAARRSSSTSPSPSPASRRSGRSRSRSPVFFSSHRSSSRSRSKRHGPPAGVLGAGSYSSRPASRSPPATRLPLNSSLGAELSRRKKERQAAEAAAARACGSGAASPATQKPIAPSASSSSSSSRPKAEDPQQEKDSGEKESQSLPSVQSPQEAPMLEEPVADPLPSASTTFSPAAPPEPSPTEAPGLTSSTPPPPPPPKPQQADAKVLPPAPSAPAKSPAPVPARSPARQTPLHKTSTLPPLPLPPLLLGNSQHSPKKSTYQRGSRKEKETRSRPSIIDLPLPPTLPGGDSSPLHLASHQGLSLTLPALKKRPKICCPRYGERKHTKSDWGKRCVDKFDIIGIIGEGTYGQVYKAKDKDTGELVALKKVRLDNEKEGFPITAIREIKILRQLKHCSVVNMKEIVTDKQDALDFKKDKGAFYLVFEYMDHDLMGLLESGMVQFSHEHIRSFMRQLMEGLDYCHKNNFLHRDIKCSNILLNNRGQIKLADFGLARLYNSEESRPYTNKVITLWYRPPELLLGEERYSPAIDVWSCGCILGELFTKKPIFQANQELLQLELISRLCGSPCPAAWPDVIKLPLFNTMKPKKQYRRRLREEFAFLPTSALDLLDRMLTLDPSRRCTSEGALNSDFLCDVDPNNMPPPDLPHHQDCHELWSKKRRRARQSGLPEDVPKAPRKDISSTSSGDNSRPQTSPAGAPAPPPPPPPPPPPPTQGKPPSAAAQDNDLSALGAAAEQLNQTEMTLLLNLLQGQTDLSLQQVSQLLNQSNAETLSQSLSALSEASDHHQGGASASEPPELSPASGDAAQLPEEQANLLALILGHIMKPQGEEQADESSNGVQSGEALTRAASTSTTERKGKSGFNVSKRNPLSTCPALPRGDQRPPSPPGLPPAASSPCLSQDISTSEMAAALLQLISDVGASSDAEGMSPNTEGSDKAPPPAAEAPTTLSDSSNAAQFLKDQDLRFSHGTTH

>Lectin5

MHVDDERHRDSTTTATKWTARQRLLQRCTLSSLLLLLLLGVDVTWSATAPLDANDFAFFHEGTRGCLGVRERSLVLSTSCREAGQRWKWVTGGRLFNLGSSLCLGVTAGNDTASSSSGPGRSPLSVYTCDRQPPMVRWTWSCGQVLEDLRNYLPWPSFSNASAPSTPSASSSKWSVHGEKQDLCGKTYRDIYTIQGNSNGRPCYLPFLYDGQWFHNCTSIGREDGHLWCATTYDYGQDERWGFCPVNSGGCETFWETDPLTDSCYQFNFQASLSWSQARESCQKQGADLLSVTKLHEQTYINGLLTGYSAALWIGLNDLDINGGWQWADSSPLNYLNWEQEQPNHEDEDNCVVIRTESSGRWQNRACSDALPYVCKKRPNATLDPFTTDSWADDEKYECEVGWKAFQAGCYKLVPEKGDWNAGLKTCQKMEANLVSIHTLPEMEFILRNIKKDTEQLWLGLHDIAMQMDFHWSDHTPVTFTHWHPYEPNNFRNTQEDCVSMWGAGWKWHSPSCYWVSEDLLTFSEASKSCEENKASLLTITDRFVQAFVNSLVFGRVDDSFWIGLHDQGSPGSFHWQSGDRVSFTHWNLNQPVSVQGGCVSLETGLVTGLWEVRECASWKAKYICQQNQDATLSPEPSLPAPPPTPSLSGSCPNGWKSNSNLRHCYKVFHWSQMDQKLSWLQAHLFCRKHGADLLSIGSSDEEHFVLQVLHETFGESEDHDQQWFWIGLNRRNPMDNGSWKWSDGLVFTYQNFGRYYYNVRQCAAADLGSMTWLAMHCDSPLDWICKIPRGSVEKEPEAAEGTTSPEWIGFQEAEYKFFDHRTTWDQAQRICSWFDSSLASVHSAQEEKFLSSTMVKVEGDNWWLGLHTFDNDGRFRWSDHSVLNYVSWAANRPRPLSRDRRCVHLIASKAEWAEQRCHSDLPYICKRVNVTGTAPPTPSSPHLPSGCPEGWSSYRRKCFKVFQRELPSQRATWSAAKIKCELQGGVLAVVSNHLEQAFVTTLLSNVTVDLWLGLSSDAKGHFQWAQPGLLSYTNWAPGQPVDNNGPHHDKNPGNCVSAIHGNPQKHKGMWASRACETESLGYLCQRQQDPGMPAAPTLIPATLPKRLELGGITYQVVQKRLDWTGALHLCNSLNGTLAAPKDAVQQAYLTLLINSLRLPAWIALYNFGDRSYTWLGEEDLPYSNWVDGQPNQLSGCGHMTTSGLWTMTPCHAKLDAAICQIGDAPDTHQWLYPGSCPRSVGDWAWLSFRNHCYAFNLQRLKLQQDARLSCQKVGAELLSILDETENAFVWEHIQSSAEQAHGAWLGVSVKGRGLVWGEETQMSYTNWESHEVAFAVLSPNSCFWIQSNSGLWKPGSCRNRTHGVICKRPRIIESSNELLSGDHLPTLIVVLVTGVVLVALIVGVIYLYRRRGGVGVRGSYDGARYSRTHSGQGEQVEKNILVSDMELNEQPE

>Hemo1

MTSLTKRDKATVKAFWDKVSPKVEEIGVQALSRLLVVYPQTKTYFAHWSDLSPSSEPVRKHGKTIMAGVGSAVSMLDDLKGGLLPLSELHAFTLRVDPANFKDLLNRKVDLSDTFFKDGSRLFNSPLMPSSFLRMGDRDEAEEEETQHQVRQDQVGQTSPHDDLLVTLDARGYAPGDISVKLEGRSLLVMAAKQAGAQEAHSCSSPSSQASFASSAASRVGFKQRIHLSPHLDLSGLSCSLMEDGQLRIHAPVARQPIAAGSSEPIAEERSELVAEERKEPIAEDKEEAPPLFRTSLEFPITKEETEAQHTH

>Hemo2

MSLTRRDKATVKAFWALIAGKTDVIGSEALSRLLYVYPQTKTYFDHWKDLSLNSAPVRKHGRIIMAGISDAIGKMDDLTGGLLTLSELHAFTLRVDPANFKLLAHCILVVMATQFPVEFTPEVHVCVDKFLAALTLAIAEKYR

>PLA2-2

RSKRGLLELAGMIKCTTERTPLDYAFYGCYCGLGGSGWPRDRTDWCCHKHDCCYDSAERQGCSTKTDRYDWSCDDRTPNCGDLTDTCEKLLCKCDRDAAKCLRNSPFQRKFTLWPNWRCGPHQPMCSIY

>Throm21

MDELTLTNAMLLVLLVTVCGRAVLNNRIVGGQEASQGDWPWQASLQFDTGKYFCGASLINSEWLLSAAHCFISHNNDVALVRLSSPVPFNDFIGPVCLAANGSTFFTGIESWVTGWGKIGEGVPLPSPKNLMEVKVPVVGNRQCGCDFKSLRKITDNMICAGLREGGKDACQGDSGSPMVTKQDSKWIQSGVVSFGQGCARPKLPGVYARVSQYQDWISNHTGNDNLPGFVTFTSPGTDPDLEVSCDDAMPTTSTGTPISPGMPPPVLCGFAPLNECQSGSGSASVAVGDWPWLARLYWNGSYACSGTLVAQDTVLTSAECIARSSTVSNWTVFLGPMNQSDSNSSETAVGVIAIRTSARSVVQSLKEVPTSLQDCGNVSSAENICINHLPLDQRNFSGPLMCQLGGFWYQVAVLKPTNHTVQNDQLTVLPRLSGFSNFLMTEVGEVLSPTNSSSATPPLLSQLLVFGTGLQVVLLMLCH

>Throm17

MVEGDNKSANMLAQETAQLEEQLQAWGEVILAGDRVLRWEKPWFPGVLVAATTALFLLIYYLDPSVLTGVSSGVMLLCLADYLVPTLAPRVFGSNKWTTEQQQRFHEICGNMVKMQRRLLGWWRRLCTLKEEKPKMYFTSVISSLLALAWIGQQVHNLLLTYLIVTLLLLLPGLNQHSVFSKYTAMAKREINKLIKNKEKKNEAGTVTAFVGRHSQTGSNPNEQTRNVSLIIFHPAYNSTTFDNDVALLKLFSPVPFNDFIRPICLAASGSTFFDGTQSWFTGWWDVTSGVPPPPSGDLTEVEASVVGNRQCDCDRTQITITDNMICAGLKAGGHDACQGDGGGPLVSKQGSTWVLSGLLSFGFDCANPNIPGVSTRVSRYQAWISSHTTNSNSLPGFVSFTSVGTDPDLQFSCNANLPLPSASPRITYTGPYITHTGPYITHTGPYITHTGPHSTHTGPYITHTGPHSTHTGPHSTHTGPHSTHTGPHSTHTGRPYITHTGPHSTHTGPHSTHTGPHSTHTDPYSSTTGPTPCPVITCPSCSCTTPITIWSSSTSSDPLSNVCGVAPLNSRIVGGQAASPGAWPWQSQLAFNPNQVSRLVVQTIIHPAYNPATFDNDVALLKLSSPVPFNDFIRPICLAASGSTFFDGTDSWVTGWGTIGSGVPLPPPQDLMEVQVPVVGNRQCDCKYGGGLITDNMICAGLSEGGKDACQGDSGGPMMSKQGSVWVLSGLVSFGEGCALPDFPGVYTRVSRYEAWIRSHTSDGNLPGFVGFFSAGIDPDLSFNCPITTTMSPSTIQSST

>Defensin4

MPSLAAFCWAILVLVQGPLGGTCQEDHRPTSSFQCGPSDQGLACANGMRCCQEGHRCSADSMWCFKPESVVLCGDQVSECPNGSTCCETPQGTWGCCPLPKAVCCEDKEHCCPGGSQCDVRRSVCVSPEDGEDMPMWNKLPARRRADWENQRANMVPCDNKSYCRDGTTCCKTTRGDWSCCPLPQAVCCSDFLHCCPQGYTCNLVAQTCDLEWRSVPWLEKEAAESRQQEKEVRCDDTHACPDGSTCCKTQTGLWGCCPVPQAVCCPDGNHCCPKDYTCDEQESKCVKGQVAIPWYPKLPASISSLLPPRTTVVAMKPGFVQCDDQSLCSDEQTCCKISPTDWGCCPFAHAVCCSDMKHCCQAGYRCTGGGQCTSNGDLPWL

>Throm30

MFPPHLSHPFLPAASLGRGHRELRSLKDLPKTMTRIQFLVLSALFCCLADSAELLRSKRGTRFYRGEPQRKISRRPLFIVHIQKPSPSFDTSPRCVDSTTSAVHSYGDTWLRWKGQRVEYCRCAFRGRELCHIVPVINCYTSHCYNGGTCKEAVYSSDYICQCPTGFSGSQCEINTKEKCVVGSGDAYRGTWSLSKSGAECINWNSTALREKKFTARKVDASSLGLGNHNFCRNPDRDSTPWCYTYKGTQIIWEFCSLPKCSQDKYEECTRGIGLTYRGTKSATKSGARCLPWDSPAIKHKSNNAWRSDALEVGLGSHSYCRNPDGDEGPWCHVYKNLHLTWELCDVPKCTRRPSTITPQGPRGPMINNNQGKCGQRLDNTLSQPTFRMFGGRESDITEQPWQAAINVYQARLRQHFHRCGGILIDSCWVLSAAHCFEPTDKPSKLEVILGRTFRKQNSSSEQIFKVEKYWLHEKFDNETFDNDIALLKLKSDIGVCAVNSPEVLPACLPEAGLVLPDWTQCEISGYGKDSEFSAHYSERVKRGFVRLWPRERCVPAVLSGRTVTANMLCAGDTRGLDDACKGDSGGPLVCRNNDKMTLMGVVSWGDGCGQRDKPGVYTRVTRYVDWIHNKMTANPI

>Throm35

METSKAALLLAAIVCTTVSGSEIMESYAKTGGAWLTAINKRMYSTNTVEECAAKCDVETSFTCSLTPENKPKADLESNFCRNPDDDAGGPWCYTTDPDTRWEHCNVPSCTEDCIHCNGEDYRGKVSITESGVACQRWDSQEPHNHGYNPNALPQKYLDNNYCRNPDGDPKPWCFTTNPNKRWEFCSIPRCTSETPTVAPETTCATGEGSAYRGTIAVTESGKTCQSWSTQSPHKHNRSPENYPCKGLDNNFCRNPDNERKPWCYTTDPETRWEYCRVPTCGDAPGPDEPVIPTEEDDCYDGDGSSYRGVTSETISGKKCQAWASMSPHRHGKTPQQYPTADLRRNLCRNPDGDRAPWCYTTDPRVRWEYCNLEKCSSRPVESTPTTEPTTPEKPSSQDPIQRDCKVGNGATYRGSTSITIMGVTCQAWSAQSPHQHNSFTPDSHPDKGLDGNSCRNPDNDVNGPWCYTTDRNKKWDYCQIPDCTGVTCGTPATKPKRCYGRIVGGCVSKAHSWPWQISLRTSSGIHFCGGTLIHPQWVLTAAHCLERSKRPSSYMVILGSHRERTLETSAQQRALEKLVLGPNGADIALLKLQRPAVINDKVLPACLPEKDYVVPSGTECYVTGWGETQGTGGEGVLKETGFPVIENKICNRPSYLNGRVKDHEMCAGNIDGGTDSCQGDSGGPLVCNAHNKFILQGVTSWGLGCANAMKPGVYARVSKFVDWIDRTIKAN

>Throm56

MLQTLGLLILQVALVQPDTNRVVNGEDARPHSWPWQISLQVKHGSRYHHTCGGTLIAPRWVLTAGHCIWPGDIYRVVLGEHDMSQQEGTEQILDILRIVVHPEWDINHVDDGNDLALLKLVKSPIMTDSIGLACLPPPGEILAHGTACYITGWGNLYSHGPMPAKLQQALLPVVQHSVCSQSGWWGNTVKTSMICAGGDDVSGCHGDSGGPLNCQGEDGRWYVHGVTSFVSSQVCNEAKKPTVFTRTSAFAQWLSEVRRRIRSPPP

>Throm28

MMLQRAVGQGMAAVTGELFPQQQVGVVVFTGTGSKTPGAPHLELGIIDTAGSTTVKPRVTVDTELTCGETSERRMHKIVGGSFITAQSQPWVAAIFHQRRRFFCGGSLIAPCWVLTAAHCFSDGKGTNLQRLSVFLGKSAINETDEVNEQKFTVEKMILHQAYNKTNYNNDIALLKIKSSDGRCAVRSESVRTVCLPPDHTQLPVGFQCVIAGFGREKSGALHYSQYLKEARVNLLSQSQCKKNSAYANLLTENMFCAAEPSWSMDSCQGDSGGPLVCEASGRLFQFGIVSWGDGCAEENNPGVYTQLTNYNKWIAEKIKLPQYTVGVMYPPK

>Chem7

MDVKLLAVVAALTLLAHVPLSQAKPISLMERCYCRSTVNSLPRGYIRELRFIHTPNCPFQVIAKLKTNKEVCVNPEIRWLQQYLKNAIKNAKLKTNKEVCLNPEIRWLQQYLKNAIKSLIMPVDGPEDQVPSSHSGLANVALCRSNSEENGGSAVLSQDPEAASPCTAVNEAGEGNSGGKRSGALVHIDRQRIQSASGADELLGLGVAIYDQDVLEQGVLQQVDEAIQEASQAAAKAEAEKEYESVLDDVRSVTVSLKHINKIIEQLSPYASSSKDISRKIESVKRQKENKEKQLKKVRAKQKRLQAILGGEIIQRFEDELLAEDAEDTEPGPSTLGSMLMPAQETEWEELIRTGHMTPFGTRIPQTEEKKEPRKLMLAENSAFDQYLADQAKQATERKRTPHAKKRRSSATHQQGSKGTASSPKDKKMRKRMRKLQITALKAHPKARTKSEPQAPKPKRKTPIEGDEVDSDGSEYLPSDEGFDPEREERDAVEEGFGEDVDDDEYELKAYKRKNEAKGKRPVKKQQSDDQYSDEEDDGKSKEKKCRDDGDVEYYRQRIRRWKRQRLLEREERRERGEELTDDSDAEFDEGFKVPGFLWKKLFKYQQTGVRWLWELHCQQAGGILGDEMGLGKTIQIIGFLAGLSYSKLRTRGSNYRRWKRQRLLEREERRERGEELTDDSDAEFDEGFKVPGFLWKKLFKYQQTGVRWLWELHCQQAGGILGDEMGLGKTIQIIGFLAGLSYSKLRTRGSNYRYAGLGPTVLVCPATVMHQWVKEFHIWWPPFRVAVLHGTGSFSSNKEKLIAEIASCHGILITSYSAVRILQDTLHRYDWHYVILDEGHKIRNPNAGITLACKQFRTPHRFILSGSPMQNNLKELWSLFDFIFPGKLGTLPIFMEQFSVPITMGGYSNASPVQVQTAFKCACVLRDTINPYLLRRMKADVKANLALPDKNEQVLFCRLTQEQRQVYQSFLNSKEVFQILNGDMQVFAGLIALRKMCNHPDLFSGGPRLLRGITEEELTEEEHFGYWKRSGKLIVVDSLLRLWFKQGHRVLLFTQSRQVFAGLIALRKMCNHPDLFSGGPRLLRGITEEELTEEEHFGYWKRSGKLIVVDSLLRLWFKQGHRVLLFTQSRQMLHILEVFVRQNHYTHLKMDGTTPIASRQPLIARYNEDTSIFVFLLTTKVGGLGVNLTGANRVIIYDPDWNPSTDTQARERAWRIGQNKQVTVYRLLTAGTIEEKIYHRQIFKQFLTNRVLKDPKQRRFFKSNDIYELFTLVDPDEKQGTETSAIFAGTGSDVKAPQKSDRTHHSKHSKPQPNEIYANNRHSLLNSSESTCTLSQGTINGTTNAENHPNSTDAEPHLSCAQLKIDTGLKRSEKRKHCDSNKVKRHKSKKRKREKDARFEGQRISHLVKKRDYKRMDAEDQNKSDDYVLSKLFKKSVKRHKSKKRKREKDARFEGQRISHLVKKREYKRMDAEDQNKSDDYVLSKLFKKSGIHSVMQHDSIIESSNPDYVLVEAEANKVAKDALKALKVSRQRCRLASSRAPPPPARKRFGQKKNSLLVGTSVHTLPNSNKCKDAAIITKTQSKKLDSGAHFSGEVAESDLSSAPPSSCTLLARMRERNHLSLPSRSQREEQEDNDGEEDGAGSSRPAPSTEFDELLADLRNFVAFQARVDGQASTQELLEYFTPRLSVKQSPVFRELLRSICDFQRGSGEEGLWRLKENLR

>Chem5

MRVNPQLVCRIILLAACCVLNTDSTFVPGRCLCPETQAGVRGKLQELSVYPKNPNCDKVTVIVTLKSTNEPVCLSPAAPMGKQLIRCWNR

>Throm43

MCARRTTESEEKLCKAKENKWKLQQLDAICQKQPRDVVLRADIREGIHPEQQELKSFHIKEEVEDKHSPELLDIKGEDEPEPLHIKEKAHESPLIKEENNFMESPVTDCIRSSAQCDGEVHCDNGEDELGCECGFRPKYLTRIVGGNISRPGQFPWQVSLHFSSEHLCGGSIITSHWILTAAHCVFGFADPSLWVVHAGEVEQPVHGAQSLAVEQIIHHARYHPKGLDYDIALVKLTKPLIFNGFVEPICLPNYGEEFEEGTMCWISGWGATEDKGETSVVLRSAMVPLLSTKTCNKPQVYQGFISSWMICAGYLGGGIDSCQGDSGGPLACEDSSRWKLVGTTSWGIGCAIRNKPGVYTRITHSLHWIRQHMEVSV

>CDK-like7

MDVPGEEELGPIRLKSLKNKAFTVPKNDRFGMCRSVRGFEKLNRIGEGTYGIVYRARDTKSDEIVALKKVRMDKEKDANTDGAFLFVCVCVCXRHPNIVELKEVVVGSHLERATAKDCLESSYFKEKPLPCEPDLMPTFPHHRNKRAAPQPEGHSKRSKV

>BPTI7

MRGMMGSQSSPVKSYDYLLKFLLVGDSDVGKGEILESLQDGSVESPYAYTSGIDYKTTTILLDGRRVKLELWDTSGQGRFCTIFRSYSRGAQGILLVYDITNGWSFDGIDRWIREIDEHAPGVPRILVGNRLHLAFKRQVPTEQARAYAEKNSMTFFEVSPLCNFNVIESFTELSRIVLMRHGMEKFWRPNRVFSLQDLCCRSIVSCTPVHLIDKLPLPVAIKSHLKSFSMANGMNAVMMHGRSYSVANSAALGVTSCGSKANSIKRSKSFRPPQSPPKTSSSSSKGNLEHEGLCPNKLNTNLWVDAQSTCERECNVDEVQSEYFYHLPVAVVRFVLCEQDCADFEKCCTNVCGLNSCVAARFSDGTTAQPGDLGRGKGAAAPGSTATCDEFICSQQGATCDIWDGQPICKCRDRCEKEPNFTCASDGLTYFNRCYMDAEACIRGVTLTVVTCRFYLAGPNTSPLPQDTTAYPTPTSSQEDPMPPTLYSNPHHQSIYVGGTVSFHCDVIGIPRPDVTWEKQSERREKLVMRPDQMYGNVVITNIGQLVIYNAQVWDTGIYTCIARNSAGVLRADYPLSVIRRADDDFSEDPEMPMGRPFSPADCLADVDTRVCSGERHVDWYYDSKLGFCMAFSNGGCEDSRNRFETYEECKASCQREGVGICSLPLVQGPCKAWEVRWAWNSVIKRCQAFAYGGCHGNANSFGTLKECEANCPTPKKKPCKTCRVKGKMVPSLCRSDFAIVGRLTELVEDLDSGLARFSLEEVLRDEKMGLTFFNTKHLEVTIDKIDWSCPCPNITVEENPLLVMGVVQDGMAIIQSDSYVRAITDRRLKKLREVLVKKTCQTL

>PLA2-1

MTGSGSRKLATGLRQGRPQQGACKEGWVAFQTSCYLLSSTTATWSKAEQQCLSHAAHLVVVNGVEELDYLSEISEITYKYWIGLVERAHEGEWSWVDGTDYDKTPKFWDVDQPDNWAFRKNACVVSGARLPKALWQFADMIECAQPGVNPLKYSNYGCWCGFGGTGSPRDDLDMCCYVHDKCYERSRKIPGCTAIIDLPYVLVYDFTCSDNQVTCSAMNDKCQAAVCECDRIAAHCFERADFNPENKNLDPKVHCIN

>Neuropep6

MGDVAKIQQNVFTFPLSVSFQLTMGDVAKGKKAFVQKCAQCHTVEEGGKHKLTMGDVAKGKKAFVQKCAQCHTVEEGGKHKVGPNLWGLFGRKTGQAEGYSYTDANKSKGPKSFKVRRIEREGNPQNIRTHARRLKEGGGFQDEQSRRGRDSRGSASRRQRSSSSIMDVLKKGFSIAKDGVVAAAEKTKAGVEEAAAKTKEGVMYVGNKTMEGVVTGVNTVSQKTTEQANIVADAAVTGANEVAQATVDSVENAALSTGFVTVVSHDSFWQC

>Throm31

MTAVFLALTAALLWPSGGRSSFIQPFGDCARSRGSGASYRGAIDVTESGARCMNWSEVGGFTERHPGKGVGEHNQCRNPDGRIRAWCFFRNQRGRVDWGYCDCKQADAILAACEPPGSKRQIPGMTAVFLALTAALLWPSGGRSSFIQPFGDCARSRGSGASYRGAIDVTESGARCMNWSEVGGFTERHPGKGVGEHNQCRNPDGRIRAWCFFRNQRGRVDWGYCDCKQGSVRLQGGRSKLDGRVEVYLGGVWGSVCSDDWRDEDATVVCRQLGRGLSGRAHALPLSRPSMAKFHWRAVRCQGDEPDLLQCPKTMWNGGECSMSAAVTCTKEHVAVMLPIRLVGGQAMSEGTVEVFHAGQWGSICDDQWDDSDAEVVCRQLGLSGAARAWRQAHYGKSSGRVWLDEVRCTGNELTVEQCLKSAWGEHNCLHSEDAGVSCSPLTDGTARLVGGTGSNEGRLEVFYRGQWGTVCDDGWTDSNTQVVCRQLGYRLGQTLVPEGLDITPVPRFGVASGPILLDDVSCTGKEPSLLLCKREEWLRHDCTHHEDVNIACNAERLGDGLPTSIPVRLVGGESPREGRVEIYLSGQWGTVCDDGWNDRDAEVVCRQMGYSGLAKARVMAYFGEGTGPIHVDNVKCSGDERSIADCIKQAPGIHNCRHSEDAGVICDYGELQPGHREAKAPVNQICGLRLIHTRQRRIIGGENSLRYGNSTKQYKVRVGDYHSLVPEEYEEEYGVAQIVPHPSYRSHSNDYDLALVRLSPGAVGHTPGECVSFSRHVLPACLPLRKERALKQASNCHITGWGDTGRSYSKTLQQAPLSLLPRRQCQQYFQGAFTSRMLCAGSLQQERRVDSCRGDSGGPLVCERPGGGWVVYGVTSWGHACRTQQSPGVYTKVSAFISWIHKVMAHNEGKQRSQ

>CDK-like22

MGDEKETWKVKTLEEILLEKKRRKELEERTDTKHPNNISQSIVPQADARESKRETPEEGELRDQRMEITIRNSPYTRQDSTEDRGEEDDSLAIKPPQQVARKEKSHHRKEEKRKDKRHHRSHSAEGAGKHARPKDKERDRDRRKRQWEEDKARQDWERQKRREQARAHSRRERDRLEQQERQRERDRKLREQQKEQRELKDRERRADERRKEREGRRDVSSHHRMLPEEYGEKLKQSHRSRSPAHVPREKTEPGELRKLTSKEERPEVQDLLADLQDISDSERKTSSAESSAASRSGSEEEEGEEEESSSKTDGEEEEGEEEEEEDEEGEEEEEGESVSVSGSERSEQSAEEVSEEEQSEEDFAEEQENGNHILPVPESRFDHDTEESGEEMEDEEEEEAGDADPTPQSQTHSRTPTPEGNYIPDSPPISPVELKKELPKYLPALQGCRSVEEFQCLNRIEEGTYGVVYRAKDKKTDEIVALKRLKMEKEKEGFPITSLREINTIMKAQHPNIVTVREIVVGSNMDKIYIVMNYVEHDLKSLMETMKQPFLPGEVKTLMIQLLRGVRHLHDNWILHRDLKTSNLLLSHKGILKVGDFGLAREYGSPLKPYTPVVVTLWYRSPELLLGAKEYSTAVDMWSVGCIFGELLTQKPLFPGKSEIDQINKIFKDLGSPSEKIWPGYNELPAVKKMSFTEHPYNNLRKRFGALLSDQGFDLMNKFLTYCPSKRILSDEGLKHEYFRETPLPIEPSMFPTWPAKSEQQRVKRGTSPRPPEGGLGYSQLGGDDDLKDTGFHLTTSNQGASAVGPGFSLKF

>Throm38

MVAAVISDDSINRVFCVTVACSHRNVKKIASIDVSKKFSKLFDEHHGPRINHVKIAQCQCANGELYDIDDMDMSVFQVLNTLPDLRLIRFTVDVSKKHKKQWQNIKCRRIRCHDESSTFRKGEKQRISEENSKCRKGTLNCVADIFLVCGCGLPAYPPVPARVVAGVDVKPHSWPWQQVVHNRLEYELHEQNKPRQPFSLSTPPCHEQSHSGRHNYRVEVGKHSLKASEENSAARRAARIITHEGYNILLSRNDIALIKLSFPVVFSDTIMPSCLPPRHAVLPHDAACYITGWGRLSTDGALADILQQALLPVVDHDTCSHSDWWGVLATDKMVCAGGDGITAGCNGDSGGPLNCQNPDGSWTVHGVVSFGSGQGCNFFQKPTVFTQISSYLDWIDRVSNKYFKVNHFHQHSRRRLGLTKSLGFAKTSYWLVC

>Antipro1

CPRLLKVVPSHKGCACDQDCPENHKCCVFDCGAACVPPSFNKPGVCPRRKWGFGQCAEFCYDDSDCPNEEKCCSNGCGHECTVPYKVKPGRCALPQGTPMCAEYCYHDGQCPEEQKCCRTTCGHACSKPC

>BPTI16

MSVAKVVYSHAGLCPNELNPNLWVDAMSTCTRECESDQDCETFEKCCLNVCGNKSCVAARYIDIKGNKGPIGMPKGATCDKFMCSQQGSECDIWDGQPVCKCRDRCEREPHFTCASDGMTYYNKCYMDAEACSKGISISEVTCRYHLTWPNTSPLPAETTLHPTTALQTTLPVDVQSPAIHSSPAQQAVFVGETASFLCEVTGKPHPQITWEKQLKGKDNIIMRPNHVRGNVVVTNIGQLVIYNAQLQDAGIYTCTAKNVGGSVSSHFPLAVIKREARGKEAQGNNTNRPFPPAECLKSPDTDDCGEESISWYYESSRNNCFTFTYSQCNKNRNHFDAYDICMLSCGGELAAPCSLPSVQGPCKAYEHRWAYSNALKKCQSFVYGGCGGNENNFESKEACEGMCPFPKNHNCKECKPRSKMVASFCRSDFVILARVTELTEEQESGHALMTVEEILKDEKMGLKFFGKEPLEVTLLNMDWNCPCPNITVAENQLIIMGEVHNGMAVLQPDSFVGSSSARKVRKLREVSSKKSCNFFKELPGTQ

>CDK-like8

MAHGTSLQYERVAEIGGGAYGTVYKARDTESGRFVALKSVRVQTDHNGLPISTVREVALLKRLEQFDHPNVVRLMDVCATQRTDQETKVTLVFEHVEQDLKTYLEKAPAPGLAPERVKDLMKQLLCGLAFLHSNRVMHRDLKPENILVTSQGQVKLADFGLARMYSCHMALTPVVVTLWYRPPEVLLQSSYATPVDIWSSGCIFAEMFKRKPLFCGESEVDQLGKIFEVIGLPPEEEWPTGVTISRKHFPPFSARPITDFVPEINEQGAQLLLYSYWLLHCFDEIVCFMLWLVFLCSMFTGCLLVWFLAPSVLITTIPCVNQLASSSHSSCPVRSRCHDGVLHVWTGRREGATVLRSCVPMSQADNPQRRTTYLISLTLVKVEALPEDVAGKQVEKEGEGSPKESEEVRHAPKEEEVRAGPQASDKESTQAGEEVEQVQVKYGSPNEKWIKAERRDQKDVPIAKSKDAAPVHPSLRQMVKVYSGGSFETTVRREGPRSDASRPKTELYREPPRLFPKGENGAELNNRSRCSLPFSVPPQVSQRPEVLMRPHTGANTACWKEAREANNSLYHSAKTLERRDNRIGDQSPLMAATTLGPYRASWADSDGRGTLIRPAAPLSGGYSGIMNRENSHEDRFKVSLVTPVTCDVNRPPRKTTSRTLDNSDLHCQGGTIHRAPVRDRKMLKFISGIFSKSAAVPPNVSTASALYPAADRGSSEEEAACTSSQEWTITQTVQELHLAVLGGLCSGKSALVHRHLTGSYLSLENAEGRQYIKDVLIDGQSHLLIIREETELPGAQFASWLDAVILVFSLENEASFQEVYKIYHQLAVYRPVTEIPFVVVGTQDKISSSNPRVIDDARARQLCSDVRRCTYYETCATYGLNVNRVFTDAAQKIMAAKKQAALLASCKSLPNSPSHSGGSTPVSGVYPGQASNGGQSSDYSSSLPSTPVISHKEIGRSARGEKQDSGTPGSVRSATRRRTARFSGRRGSDSARRSADCKGDVGSGRFIPIKQGFLLKRSGNSLNKEWKKKYVTLSSDGILSYHSNVNDYMLNTPGKEMDLLRVTVKVPGKRPPRAVPTGGPPVGLKDVQGPEGASLVPVSASSLLQADDMEGLGGALFLRSNRGVQRCSSNVSNHAQSVDSAVEGVSSSSSTKDVGSTSPVEDRKKHRRKKSMNQKGDATIGQADDEENNDFVIVSSSGQTWHFEAQSVEERDSWVQAIESQILASLQLCESSKNKARKNSQSEAVALQAIRNAKGNGFCVDCDAPNPTWASLNLGALICIECSGIHRNLGTHVSRVRSLDLDDLPRELTLVLSAIGNHMVNCIWEARTMGHRKPPPDATREERESWIRAKYEQKLFVAPLPPPTPNEGPDITLSGRLLLAAMEHNLPKLLLLLAHCTKEDINGPPSMALSSRSLLALRLPGSALHAACQQANVVMTQLLVWYGCDVRYKDAQGQTALTLARMAGSQECADILLQYGCPNESAPSAATTAGFTSAANPGFPVAMTPNLTVATTLRISHRGGGGGGAASFSYSTSRKAVS

>CDK-like1

MDSFQKVEKIGEGTYGVVYKAKNKNTGETVALKKIRLDTETEGVPSTAIREISLLKELSHPNIVKLHDVIHTENKLYLVFEFLNQDLKKFMDSSTVTGIPLALIKSYLFQLLQGLAFCHSHRVLHRDLKPQNLLINAQGEIKLADFGLARAFGVPVRTYTHEVVTLWYRAPEILLGCKFYSTAVDIWSLGCIFAEMVTRRALFPGDSEIDQLFRIFRTLGTPDEATWPGVTSMPDYKPSFPNWARQDLSKVFPLLDEDGRDLLREMLCYDPNKRLSAKNALVHRFFRDVTMPLPHLRL

>Throm4

MMSLSCVFVNSTPTFTLRIFLGRQTQEGTNDNEQARGVAQIILHEDYNPSTNDNDIALMRLSSPVEFTTFIRPVCLAAQGSDVPAGTEAWVTGWGTVDSGIPLPSPQALQEVTVPVVSNSDCNDVYGIITSNMICAGTESGGRDSCQGDSGGPLVSKNATRWVQLGVVSFGRGCALPDIPGVYARVSQYQTWIRDRITGDQPGFVDFVPGTTTTGGATLVFLSAPLLLTLLPTIFTSLVLM

>CDK-like13

MEKYEKLAKIGEGSYGVVFKCRNRDTDQIVAIKKFVESEDDPMIKKIALREIRMLKQLKHVNLVNLLEVFRRKRHLHLVFEFCEQTVLNELEKHPRGVPEAQLKSIVWQTLQAVNFCHKHNCIHRDVKPENILLTKTGVIKLCDFGFARILTGPEDDYTDYVATRWYRAPELLVGDTQYGPPVDVWALGCVFAELLSGSPLWPGKSDVDQLYLIRKTLGDLIPRHQQVFRSNVFFSGETLEKRFSGSSPLALQVMKAQYLPQLTNSNMSPAPDVKKPAKHKYHLPNI

>CDK-like19

MQKYEKLEKIGEGTYGTVFKAKNRETHEIVALKRVRLDDDDEGVPSSALREICLLKELKHKNIVRLHDVLHSDKKLTLVFEYCDQDLKKYFDSCNGDLDPETVKSFMFQLLKGLAFCHSRNVLHRDLKPQNLLINRNGELKLADFGLARAFGIPVRCYSAEVVTLWYRPPDVLFGAKLYSTSIDMWSAGCIFAEEQWQTMTKLPDYKPYPLYPATTSLVNVVPKLSSTGRDLLQNLLKCNPVQRISAEEALQHPYFADFCPP

>CDK-like20

MQKYEKLEKIGEGTYGTVFKAKNRETHEIVALKRVRLDDDDEGVPSSALREICLLKELKHKNIVRLHDVLHSDKKLTLVFEYCDQDLKKYFDSCNGDLDPETVKSFMFQLLKGLAFCHSRNVLHRDLKPQNLLINRNGELKLADFGLARAFGIPVRCYSAEVVTLWYRPPDVLFGAKLYSTSIDMWSAGCIFAEEQWQTMTKLPDYKPYPLYPATTSLVNVVPKLSSTGRDLLQNLLKCNPVQRISAEEALQHPYFADFCPP

>Histone38

MSNDISKKRGKSSGEKRAKKKVKRKETYAMYIYKVLKQVHPDTGISSRAMSIMNSFVNDLFERIATEASRLAQYNKRATITSREVQTAVRLLLPGELAKHAVSEGTKAVTKYTSSK

>Transfe1

MAKKAPVFTCVKRSDSLGCIAAIHEGIADAITLDGGDIYTAGLENYKLHPIIAEDYGAGSESCYYAVAVVKKGGAFGLRDLRGKRSCHTGLGKSAGWNIPIGTLVKLDVLKWGGIEDESIESAVGKFFLKSCAPGAEKGTSLCQACKGDCSRSHNEPYYDYGGAFQCLVEGAGDVAFVKHLTVPAVVLSAPPENVRWCVKSEKEHEKCVAMAKKAPVFTCVKRSDSLGCIAAIHEGIADAITLDGGDIYTAGLENYKLHPIIAEDYGAGSESCYYAVAVVKKGGAFGLRDLRGKRSCHTGLGKSAGWNIPIGTLVKLDVLKWGGIEDESIESAVGKFFLKSCAPGAEKGTSLCQACKGDCSRSHNEPYYDYGGAFQCLVEGAGDVAFVKHLTVPDNMKADYELLCLDNTRAPIDDYKNCHLARVPAHAVVTRNDPELTELIWNSLNSVQGFDLFSSEAYAPAKNLLFKDSTQKLVRLPANTNSFLYLGASYLGIVRSLSKDVTAARSNAITWCAVSKIELRKCDKWSVNSLEDGENIILCQSAPTVEECMKLIMSKNADAMAVDGGEVYTAGKCGLVPAMVEQYDEAKCSQSGVSASSYYAVAVVKRGSGVTWDNLRGKRSCHTGFGRTAGWNIPMGKIHKQTGDCDFSKFFSSGCAPGSPPDSPFCSQCVGSGKIVDDKAKCRASAEEKYYGYAGAFRCLVEDAGDVAFIKHTIVAENSDGNGAAWAANVLSGDYELICPNKDPVPVSDFESCHLALTPAHAVVTRPESRKAVVDILLKQQERFGKDGSDSNFRMFESAPDKNLLFKDSTKCLQKVPSTNDYKSFLGPDYVDAMISLRECSDATPGKFFSSGCAPGSPPDSPFCSQCVGSGKIVDDKAKCRASAEEKYYGYAGAFRCLVEDAGDVAFIKHTIVAENSDGNGAAWAANVLSGDYELICPNKDPVPVSDFESCHLALTPAHAVVTRPESRKAVVDILLKQQERFGKDGSDSNFRMFESAPDKNLLFKDSTKCLQKATYGPGQPQSLVFATPPPPQMNSAPQPRQGYYSNRPTMATSAPRVQTSSVSRPVGPPHVYPPSSQMMMIPQQQLSFPGSPQGYFLPPGQYRAPFMTPTQQYPVTSGTAGFYTGTSPAEYSGYEPSLAARERRGGGGRGGGRENGRLSLHGAPLTSQRYPAAAYYPAQPQYSPSVQPAQVIINPAQQQQPAPPPQQQPAPTQGPPKRERKPIRIRDPTQGGRDITEEIMSGGRSTTTPTPPQANVTEAGLAQTNGEVIPPVAAVSSRDDIVEHVASVETPPPPPTTNPEPALESAQEADNQNVPAAQAEPVAPDLVPEVLAQVVEEQPVPAVLPAVTSASSTVEVEMDTNVADAVDAPVCPSPSTAQEEEPQAAPAPCENVPENQVEEMQLAEETEEPVEVAAVEPIAELAKVIAPVTTETEEIPTKMATEAPPVSVQEPIAPLTPNAAAEPEPEPKPQPTPAEPAEPPLSNGLPQDVQELGEEDITPCDQPDAFQSQESPPVAKTAASVVEDVDVKEKEVVAMKEKKEEVVKVAKSEEVPPVAVNCPTEESAMHAATSVPKKRRNMKEINKKEAIGDLLDAFTEEQDAKPAAEPSSAQADPVPVAPAEPPAEVVDETWEEKEDKQNAEPESKAESADKKYQYKEEQWKPINPEDKKRYDRKFLLGFQFISASMNKPEGLPVISDVVLDKVNKTPLRPADPVRVQDFGPDFTPSYLGNLGSRSVGGPRGPPPGPRRSQQGQRKEPRKIITSMSLNDDVQLNKAEKAWKPSAKKGPRNRGEEEVTAVTDEDLTPEQAQTQEILKRLRSILNKLTPQKFQELMNQVKELTIDSEERLKGAIDLIFEKAILEPSFSVAYANMCRCLSGLKVPTSDKPGYIVTFRKLLLNRCQKEFEKDQDDDEFFDKKQKEMEAAKDDEVRERMRVELEEARDKARRRSLGNIKFIGELFKLKMLTEAIMHDCVVKLLKNHDEESLECLCRLLSTIGKDLDFEKAKPRMDQYFNQMEKIIKERKTSSRIRFMLQDVLDLRRCNWVPRRGDQGPKTIDQIHKEAEMEEHREHIKVQQQLMSKKDTGGGGGGRMGGGPGGRGPHTPGGGRGSVPQDEGWNTVPITKRPYDTARLKITKPDNPDLNNQRLAPSGKGWGKGSSGGMGVKTATGDQDSGRSTTSTVNRFGALQQSSSLSSDSDRRAPQRSSSSRERGGDRDRADQDRDRFDRFDRDDRNSRSQNTKRSFSRETEERGGRGGDNKPTNEPVRRVASMTDSRDRGSRDRGSRDRGSRDRGSRDRGPSKDLPAKRETAPAPSLPKPALTEEEVEKKSTAIIEEYLHINDLKEALQCVAELNSTALLYVFVRQGMESTLERSTSAREHMGMLLHKLTSSGTMPAPQYFKGLLQILEVAEDMAIDIPHIWLYLAEIITPMLLEGGIPMRQLFGEIAKPLLPLGKASVLLVHILHLLCKGMTRSKVGALWKEAGLNWSDFLSKNEDVNKFVTDQKVEFTTGEEQESKESTKKSLSADELRQELDRLLRDKASDQRIKDWVEANLDEQQSSSNQFLRALMTAVCQLAITGDPYKVDVPLINNRAALLQSFLSDEQKELQGLYALQALMVHMEQPAYLLRMFFDVLYDADVIKEEAFYKWESSKDAAEQNGKGVALKSVTAFFTWLRESEEESDKE

>Lectin17

MTVKYQTSTETEMDIDDGNGGYKHLIMNGGKLKYSVYALRNSPFRAATLILGLMCVALVTGVIGQSVHYWKVGKDNQRKLEVVGGEKDKLQSQLKTVQNEKKNLEVNHEHLQQSYNFISKRTNQIQTNNNLLKSEANQLKDSQSKLQASNAALKKDLEKLTSSQEQLKTNNDALSTARDLLQKKYESMGQSKNVLQANYDSVIKERDNLQNKFNNVTRSKEKLQMSYNVLIKDIEHLQERYTSSSNERDTIASSHQNITLEKENLQAIYTTLAKATDELMASYNSTVEEKKFLESRLKNVTAERDLQRVEISNMSAEVDQLRATVTRLNATIKDKVCQSGWKKFENSCYFSSTSKKTWYLSRNYCQGKGADLVIINSQAEMVFINGLFSSNKEVWMGLTDEGVEGQWKWVDGTELTLKYWADGQPNSYNGNQDCGEFWYRSSGNAEWNDEKCSSQRYWVCEM

>Throm42

MAVLWILSCFALVSAAYGCGTPAIPPEVTGYARIVNGEEAVPHSWPWQVSLQQSNGFHFCGGSLINENWVVTAAHCNVKTYHRVVAGEHDKGYGSNEAVQVLRPAKVFTHPRWNPRTINNDIALIKLSSPARLGTNVSPVCLAEAADDFAPGRTCVTSGWGLTRYNAPSTPNKLQQAALPLLSNEQCKRHWGSNISDVMICAGGDGATSCMGDSGGPLVCEKDNAWTLVGIVSWGSSRCSTTTPAVYARVTELRAWVDQTLASN

>Throm54

MTRCFLPAADFPEFHVSKLSRPLIACASVATPRPPLSSNRAQMRPDKRPAFVGRRHAVAMAFLWILSCLAFVGAAYGCGSPDIPPVITGYSRIVNGEEAVPHSWPWQVSLQDYTGFHFCGGSLVNENWVVTAAHCNVRTSHRVVLGEHDRSSAEEDAQVLRVGKVFKHPRYNGFTINNDILLIKLASPAQMNMRVSPVCVARAGDDFPGGMKCVTTGWGLTRHNAPDTPALLQQASLPLLTNEQCRRFWGSKISNLMICAGASGASSCMGDSGGPLVCQKEGAWTLVGIVSWGSGTCTPTMPGVYARVTELRAWMDQTMAAN

>Defensin2

MKGLGLVLLVLLLLFAVVEGNDPEPQYWTCGHRGLCRRFCYAQEYMIGHHGCPRRYRCCAIRF

>Histone10

MSGRGKGASKTRAKARTRSNRAGLQFPVGRVHRLLRRGNYAERVGAGAPVYLAAVLEYLTAEILELAGNAARDNKKTRIIPRHLQLAVRNDEELNKLLGGVTIAQGGVLPNIQAVLLPKKTGQSAPSSGKGGKKASSQSQEY

>Lectin1

MPENRYTPGGSRCVSEQGRMFGVHCRNKQARKEELGSVVGPGRTCDVMPPCGLQNLLALLLLLHAVSHGLSDADPVVSMHKVTYHSISEEMSKTVVLPCLFSLRPGAIPSHEPPRVKWSKVWGQQGSDGSRKEQSILVAKDNVVKVKKAFQGRVSLPGYSDNRYNASLALTGLRSSDSGLYRCEVVMGINDERDTVPLEVTGVLFHYRAPPDRYALSFADAKRVCAENSAAIATPGQLQAAFADGYENCDAGWLSDQTVRYPIQSPRPGCYGDRDDSPGIRNYGNRFPDELFDVYCFAKRLQGEVFHSTVEEKLNLATASTHCHSLGAQLATVGQLYLAWQAGLDQCDPGWLADGSVRYPINVPRRNCGGDEPGVRTVYSNPNRTGFPDTTAMFDAYCYRAHQPADVQAAEALAPLHAKIPSAQTGSPLSRTQMGAPLPKTATRTDLTDLNEAITNDSSDISDEHVVIHLSPSQGWDNQQDGAPTGRSRPAEPDEMKTSNAPTEQESHGGSAQEEVDASFDTLPSAASTTSQPHTSKSMLTDFVNTLMKPFKYWAGSGMGQTSNRSQTLSSQASENKGGSDNAITEHGSHDSPSRLLHASGLQSAEDGLSEQEKEEMPLFPVLSAVHNGRKIESTTQQEGKFANSGITAVDPESHTSPNERVKFPKGSPAWAIKSLRRFRVGFPRNVARSHTWDPLEAKSGLLEIITLPDSFRQDTVENYSGEGRDQVGDEAQTTTATGTDQKKEVEGSSEGNSFLSNFANTVPSSPESEPSLHQSQSELVTVAELTTSSRWQLVAQPTTATLPQTAEEARGEIIYVHRPADNLSSASSLSQRGESGSNGGFTPVIKKQRKGAGRKPGRGAAKLVTSTPESLMDVLTTTEITTAHFLTTTDTPSTDPATAAVTTELSSKSYSAQEPSISVSWIEETTSNAEGNFSMESSTTTASQLTSVVSDSKDAVTEVESRLAVSESFVFGSHWIPSKGSSPKFEDHKTPTVTDSKGAKHPFGVLVPNWAFVPPVESNPCQTNPCLHGGSCLQEGDGYSCYCPQGFSGESCEIDTEGCEHAWRKFHGHCYRYFSRRHTWEDAEKDCREHSGHLASIHSLAEQNFIGGISHDNTWIGLNDRTVEDDFQWTDKMDLQYENWRSNQPDNFFAGGEDCVVMIAHENSKWNDVPCNYNLPYVCKKGTVLCGAPPQVDNAFVVGRKRSHYDIHSTVRYQCVDGFQQRHVPTTKCRANGNSDRKKHSHVHTSDKPYNCKVRGCDKSYTHPSSLRKHMKVHCKSPPPPSSGYESSTPSLVSPSSDPGREPPPPPAPPPPSGGGPTPTPSSHPANLSEWYVCHSSGASGAHSGPSTPDSATEDDGPPYRERDPRDNL

>CDK-like23

MQREKNNNSGGAEKPDREAAIMSKYYDGVEFPFCDEFSKYEKLAKIGQGTFGEVFKAKHRQTGKKVALKKVLMENEKEGFPITALREIKILQLLKHENVVNLIEICRTKATQYNRYKGSIYLVFDFCEHDLAGLLSNTNVKFTLAEIKKVMQMLLNGLYYIHRNKILHRDMKAANVLITRDGVLKLADFGLARAFSLAKNSQGNRYTNRVVTLWYRPPELLLGERDYGPPIDLWGAGCIMAEMWTRSPIMQGNTEQHQLTLISQLCGSITAEVWPGVDKKYELYQKMELPKGQKRKVKDRLKAYVKDPYALDLIDKLLVLDPAQRTDSDDALNHDFFWSDPMPSDLKNMLSTHNTSMFEYLAPPRRRGHMPQQPPNQNRNPATTSQTEFDRVF

>Throm58

MARTSFCDSKPLLDLHMADERRGLIRTLPTVFGPADCWQPVEVSESRLVPAKREPGPRWVSALPSLYTDRILSAKMNSIFAGFSDEVWTLSSASISPAKADGFLEEPLRLYPRSLERFPGLQALVPSLLRHEVEIALEKLDLTWDRTFAWEMFLDMMRSQTWHSLPNAELPRHFTDATLAVLVDWLVQVHELMHFQEETLYLAIHLLNRSLRQVKVTTATLQLLGIVCLFLAAKKEECLLPEVSGLCYLMDHAYTKHQLLRMERKVLTGLKFNLSYCPPLHFLLLLASISRCSAKMVWMARYLLELSLLEGRCVVFLPMQLAGAALCLARQVLQEPLTPEGEAAWCLASGIHAGSETVLLRIMQILAAAAARALAQETCATFIKFSSPETMHMVWVALLKVVSPGEEKEASMVKEELVMGERSSPPASSKYAKEKSNNSTLRAMRQNKDSDDCSERGCRCSSEKDSGYSDNGADWQQTDADQQSNKHQAREAAGVLRFGQSQDCGKGKPGDPKWMPRGQAIQPIYILNHRALKQTEAIPKSGWRFWNKSSSDVGVSDGAHRIFFQQPSLMPAALQIHKPFSQKNSTSGNKVTPYRPILDSYPRLAPHPSKKPPDKSALSDGVQNFSKRVCTEQKGDLEPIARSQQPAISTLRQPASSSSSPTREPSNSAHRSPPTSALTNGGPQRSNRHRRFFNTADVLRQSGLLDITLRTKELLRQSNATEREIAELRWHTELLCQAACSSPNGPSTWQRVYRTMADSNRYADLQHLRLPAHWESACRLESAANEASQRRDPSARLSETQRQNCVCVEPRREFLSRETSPENVTFVPPDSSTAEAEHVVSLTQTFGSFQSPNFPATYPDNMRHRWDIRVPDGFRVRLDFSHFDLEPSDMCQCDYIRVEAGGGASDVGGSSIMGVFCGGKSQDPDSAPGPEPLGASGNIVSVVFSSDFSNQQNFSGFQAHYSAVDVDECVEGVDENDACDHLCHNYVVECSGLVFSERVGSLSSPDFPSPYPKRSECSYVIQVSPGLKLRLHFDDPFDVEDHPQTLCPYDYIKIRAASKEFGPFCGGKSPGVIQTDSNIVAILFHSDHLGENVGWRMTYTSEESQCTSPVHGANATVIPLQPEYSAGDRVRVSCLPGFMLIKDGERMDADFEMHCQQDGTWDTALPVCQSKREYGCSPDVPRGAELPSCMHACGRPSRPLAVQVKRIVGGRAAEPGNFPWQVLLSVEDSSRVPSERWFGSGALLSDTWILTAAHVVRSRRRGTHVVPVAPDHIKVLAGTVDVRDKWASAVLAVSRVLVHPDFCPSDFDNDIALLKLSTKAELNRVVRPVCLPPPPQSDASAPPPRSLGVVAGWGVSHPDTTSDLLQFVRLPVVPQDECRASYAARAGGYNVSDNMFCAGFYEGGRDTCLGDSGGAFVAQDPVSRQWVVFGLVSWAGPEECGSRRVYGVYTRVNKYLKWIQRHLAVVP

>Histone44

MHLNAAMYAANASVHSYPDVMAEVAPAPATPAKATKKKASKPAKKRVGPSISELIVKAVAASKERNGVSLASLKKSLVASGYDVDKNRYRVKIAVVGLVNKGTLLQTKGTGASGSFKMNKKAEGKTKAPAKAPKKTPTKAKKPAAKKATPSKKPKTTPAKKAAAKKSPKKPAKASTPKKAAKKPTKSPKKATPTKKAPAAKKTPAAKKSPAKKKYGLDVGQHAALSDGHSAQKFVELLVVSYGQLQKSCTMARTKQTARKSTGGKAPRKQLATKAARKSAPATGGVKKPHRYRPGTVALREIRRYQKSTELLIRKLPFQRLVREIAQDFKTDLRFQSSAVMALQEASEAYLVGLFEDTNLCAIHAKRVTIMPKDIQLARRIRGERA

>Histone36

MPEPAKSAPKKGSKKAVAKSVSKSGKKKKRTRKESYAIYVYKVLKQVHPDTGISSKAMSIMNSFVNDIFERIASEASRLAHYNKRSTITSREIQTAVRLLLPGELAKHAVSEGTKAVTKYTSSK

>Histone1

MSGRGKTGGKARAKAKTRSSRAGLQFPVGRVHRLLRKGNYAQRVGAGAPVYLAAVLEYLTAEILELAGNAARDNKKTRIIPRHLQLAVRNDEELNKLLGGVTIAQGGVLPNIQAVLLPKKTEKPAKSK

>BPTI12

MFLSLESVLLAAALACVWAQDLRDDRRAKSRTKTMAVNIYDGQAIIDEDCSLELAFLLDSSESAKDNHEQEKQFTMNLVDRLKGLKLATGRSLSLRVALLQYSSTVITEQRFNDWRGTENFKNRITPIVYIGHGTYTTYAITNMTKIYQEESSPGSIKVAVLLTDGSSHPRNPDIFSAVAEVKNQGVKFFTLGITRTANEPSNVAQLRLLASSPTSDFLHNLQDKDIVEKMATKITDLADEGCPLAQNCACEKGERGLSGPAGKKGRPGEDGAPGLKGQKGERGECGTPGIKGDRGPEGLIGGRGLRGLQGLPGPHGDIGPEGPQGKQALVHTVHQDLKGHKVFKGKRDQPVRVFLAQRGIEGSRDRGDQEDNRGELGPPGPPGPTGLTGVGIQGEKGVEGPRGPPGVRGIPGEGLPGPKGDQGLPGEQGSPGERGIGEAGPKGEPGSAGLGGLPGLPGEDGAPGQKGEPGLPGSRGSEGLPGIGTQGEKGDQGLRGIRGLHGPPGISGPSGPKGDLGPTGPPGPIGETGLGLPGPKGDIGFRGLPGLPGPPGEGIQGPPGNVGRAGPPGPNGPPGEGIQGPKGDPGSQGMTGPRGPRGDGFPGAKGDRGSQGERGMKGAKGDLGDPGLPGEAGKTGTKGDAGLTVSSFASVSDCSDKLYRFGSNTVGFVFQREDIIKLIKEICGCGVKCKERPMELVFVIDSSESVGPDNFEIIKDFVTRLVDRTTVGRNATRIGLVLYSLDVHLEFNLVRYINKQDVKQAIRKMPYMGEGTYTGTAIRKATQEAFYSARPGVRKVAIVITDGQADKREPVKLDIAVREAHAANIEMYALGIVNSSDPTQAEFLRELNLIASDPDSEHMYLIDDFNTLPALESKLISQFCEDENGAVIYNRIANGHWNGNNGVGIHGNNGYGQNSNGFNDGYGTTKNEVNGNGYQEEVTNVRRANSHGRGDTFTLPISADPLPIQVEEDEDGEDLDLRAHVRASGSVVVVNKTLSLPRAKESTVSKEAVLSSKSSSSSSSSSSTSTAGSSSSNSNQLQPVVSPVLPEEVALRDPRCNLGLDQGTCRAYNIRWYYDKQANSCAQFWYGGCAGNDNRFETEDECRKTCVILKTASNHSCFIIDDKNKTKGSSDHISNMASVLDALWEDRDVRFDITAQQMKTRPGEVLIDCLDSIEDTKGNNGDRAVGYNSIINITTRTANSKLRGQTEALYILTKSNNTRFEFIFTNLVPGSPRLFTSVIAVHRAYETSKMYRDLKLRAALIQNKNLRLLPREQVYDKINGVWNLSSDQGNLGTFFVTNVRIVWHANMNESFNVSIPYLQIWSIRIRDSKFGLALVIESSRQSGGYVLGFKIDPVDKLQDALKEINSLHKVYSANPIFGVDYEMEEKPLPLEEVTQEQPPDDVEIEPDDQTDAFTAYFADCNKDDAEGEEAKLPGYLNDTRRYGMFAKDLVLMINDTAAVAYDGQENECFLAAMTEQIPRNHVSLVSKKNNLYVLGGLFVDEDNKDNPLQCYFYQMDSLGGGWIALPPMPSPRCLFAMGETDNLIFAVAGKDLESNESHDTVMCFDTEKMKWSETKKLPLRIHGHSVVSENGLVYCIGGKTDDNKPTDKMFAYNHKRSEWKDVASMKTPRSMFGAVIHKGRIIVAGGVNEEGLTAACEAYDFSTNKYEDDKKEWTGMIREMRYAAGASCASMRLNAARMAKL

>PLA2-3

MNTLNALLLLALSLSVAQSLDYRALNQFRKMILCVMPDSWPVLDYADYGCFCGKGGSGTPVDELDRCCAVHDQCYSDAMQHPNCWPIFDNPYTELYHYSCDKEQKKVTCGSKNDKCEMFICECDRKAAECFARSPWNPEHEHLPSHMCE

>PLA2-4

MNTLNALVLLALSLSVAQSLDYRALNQFRNMILCVMPDSWPVFDYVDYGCYCGKGGSGAPVDELDRCCEVHDKCYDNAMQHPNCWPIFDNPYMEFYDYSCDKEQKKVTCGSDNNECEMFICECDRKAAECFARSPWNPEHEHLPSHMCCEVHDKCYDNAMQHPNCWPIFDNPYMEFYDYSCDKEQKKVTCGSDNNECEMFICECDRKAAECFARSPWNPEHEHLPSHMCE

>CDK-like15

MNASVDLSRRNPQEDFELIQRIGSGTYGDVYKARNVNTAELAAIKVIKLEPGEDFAVVQQEILMMKDCKHSNIVAYFGSYLRMEYCGGGSLQDIYHGDTHILIVYVYVMSSLLLSDLSLSSLYFFIPPPPVVTGPLLESQIAYMSRETLQGLYYLHNKGKMHRDIKGANILLTDNGYVKLADFGVSAQITATLAKRKSFIGTPYWMAPEVAAVERKGGYNQLCDIWAVGITAIELAELQPPMFELHPMRALFLMTKSNFQPPKLKDKVKWTDNFHHFVKVALTKNPKKRPTAEKLLQHPFVSQPLSRTLAKELLDRAKNPDHNNYNDFDDDDPEPESPVSVPHRIRSTSRSTREGKTLSEINFGQVKFDPPLRKETEPHHEPDLQLEYGHESPCLLGGNKGHVAHLGDDEDEEDDGADDDETHTQSLLKSVEEELQQRGHVAHLGDDEDEEDDGADDDETHTHKMNTILRPKVPPPLPPKPKSISSPQPKQDDSQSHSEDDGGGGGTIKRCPAPQTASPAKPASNVPPRPPPPRLPPHRRSSLGNETASERLDADTCAPEDDGSFRHFWEWLHTPHTEEELEEAWEVLKEVKEEQEKENEDERNGLSASHNGERNSPADRQQSTMPPSVPIRKDKKDVPMPSSNGLPPTPKVHMGACFSKVFNGCPLKIHCATSWINPDTRDQYLIFGAEEGIYTLNLNELHETTMEQLFPRRCTWLYVMNNNLLSVSGKASQLYSHGMPGLFDQARQLQKLPVAIPTHKLPDKMIPRKFAVSTKIPDTKGCQKCCVVRNPYTGHKYLCGAFQSHVMLLEWVESMQKFMLIKTIDFPLPCPLEVFEMLVVPEQTYPLICVAVSKGSELNQVVRFGTVNPNPNATSSWFTETDTPQTCVIHVTQLERDTILVCLDRCIKIVNLQGRLKSSRKLSAELTFNFQIESTVCLQDSVLAFWRHGMQGRSFKTNEMTTEIPTTEASLWQGIPDSLLGLLRPADIEHFGLLERKVVVLESRPTDNPTAHSNLYILAGHENSY

>Neuropep3

MSQRSGPHLLLLFALFSVLYSRTLSLPYASMRPTRHADGLFTSGYSKLLGQLSARRYLESLIGKRVNDELMDEPVKRHSDAIFTDNYSRFRKQMAVKKYLNSVLTGKRSLENAVTSDQEESRDDPDTFHESYDDINVDHLLNNLQLVCDTRRVKRVPLLQVNCLNCRFC

>Throm12

MHQATCKDGVSSYTCECRNGFIGVHCEIDLLKRCDLNNGDCGHFCEPMGIIGGKCYCAEGYKLMRDGMSCEPEAEFPCGRTALMRTARSPTLSVNPDELYPLHRIVGGASVIPAEIPWQAALVDIKEKEVFCGGSVLSAWWVITAAHCLAEAPGAFVVRVGEYNIKRKDNTEQDLQVSQQHMHPLYNPRVNPYDHDIALLHLQRPINFSIDVRPICIGPKAFTEALVKRASPATVSGWGRTRYLGLPSNTLNKVEVPYTSRAECKRTSSVSITPAMFCAGYYNEAKDACQGDSGGPHANKLNNTWFLTGIVSWGEECAKYGKYGVYTRVSLYVNWIHNMTHGLDQHHEEMSDFEDSIEWLKKQQ

>CDK-like6

MDGLSRWMRRCCCGEVEEDEGGKDPWKGVEEEEEQVEVFKMEERTVKKSNSSSSLPVSELNPDSGPQPHWFHTLQMRRLRVQRDRSNSDPWSQTNCQNPFFWKPGLQFGTANSYVSLEKLGEGSYASVYKGISRINGQLVALKVIQTKTEEGMPFTAIREASLLKGLKHANIVLLHDIVHARDSLTFVFEYVQTDLAQYMNQHPGGLHSHNVRIFMFQLLRGLSFIHQRRILHRDLKPQNLLISYLGELKLADFGLARSKSVPCQSFSSDVVTLWYRPPDVLLGSTDYSTALDIWGAGCIFVEMLQGAPAFPGVTDVFDQLQQIFMVLGIPSEDTWPGVNRLPNYHSDWALPSKLKPIRSVWKRLSRLPCKTEDLVQRMLTLAPADRIPAPDALLHSYFNTLPPLVMHLRDTVSIFKVPGVHLETEFRDTFAPGGRVRPSLLPVAKFW

>Throm1

MVGSAAVLLLFLVQGCLAEHVFLDSQQASEVLIRMRRANQMFEEVKPGNLERECYEEICDHEEAREVFEQPDKTDLFWQKYQAHKLSSDCKGTTLERTQSNIDAVKNCITGQCISSKGLNYNGDIHITKSGRLCQRWTSGFPHPIIREFNASEPDSILKENYCRNPDGQDEGPWCFTQDPMVPKETCRVPIWETFVPTTVAPAPKSAGDCLLENGVDYTGDLAVSLGGHACLQWSLPEVKKLSQGKDFLPDVSLVDNKCRNPDNDPEGPWCYVDVSGNVTVDYCNLQLCEEELIDYDSPPESEGRERSVHAPARKAFFNPRTFGQGETTCGMRPLFEKKNIEDANEKELVESFQQQRIVGGRDAQVSSAPWQVMLYKRSPQELLCGASLISDQWILTAAHCILYPPWNKNFTTKDILVRLGKHNRAKFEWNIERIVAIDKIIVHPKYDWRVNLNRDIALLHMRRPIAFTDQIHPICLPNKKVAKMLMNAGFKGRVTGWGNMKEAWSPSERALPTVLQQIHLPIADQSTCKRSTSVRITDNMFCAGYKPEDSERGDACEGDSGGPFVMKHPAENRWYQIGIVSWGEGCDRDGKYGFFTHVFRMSRWMRKVIENSDDDE

>Throm10

MDPLETGQKFSPRQDLDLDPSLQFLPAPDAKKLEKRAVPHRKLWVAAGLVSAAAALSLLTGMLVWRFHLHRDGAQKRIYVGSMSIGNQRFLPAYEDPGSEPFVKMATLVSQQLKVIYDTKAPLGKYFQRSSVEAFSDGGGDSVTAYYRSEFSVPAPQQASADMAVQSLQTAAGSPQSRQGRVLLKPQRWLNVDAVVTQVKKSFDVHVREGGVIRSPGFPDSSYPSNIFLQWRLRADAAHRVRLDFQKMILEDDCQHDFVRIYDSLAPIEENTIAEHCGLPRRSLSFVSSGNVMLLTLVTNEEKNFPGFVANFSQVSLAPPDCGGTLSGDVGVFSSPYFPSSYPPQTSCMWNIQASTCYSPLQLGDQSFQVYFDGGGDSVTAYYRSEFSVPAPQQASADMAVQSLQTAAGSPQSRQGRVLLKPQRWLNVDAVVTQAIDPRMTSPSLLVKKSFDVHVREGGVIRSPGFPDSSYPSNIFLQWRLRADAAHRVRLDFQKMILEDDCQHDFVRIYDSLAPIEENTIAEHCGLPRRSLSFVSSGNVMLLTLVTNEEKNFPGFVANFSQVSLAPPDCGGTLSGDVGVFSSPYFPSSYPPQTSCMWNIQASTCYSPLQLGDQSFQVYLLCGAKDGRTVVASRSNRMTVKFNSDWSYVHQGFSAAFEAFIPTCPGKFRCTNGLCADSRLRCDGVDDCGDASDEEDCGACSPNAFHCSNGHCVSRATVCDGRDDCTDGSDEFKCEKSVAMTTCPDSSFRCRNGRCLDKVNPECDGQEDCEDASDEEACQCGLRPYRSSRIVGGEASQEGEWPWQVSLQLVGQGHACGASVLSSRWLLTAAHCVQDKGSVRHSRADQWEALLGLRVLSRANEWTVRRKVMRIVAHRDFDRLTFDNDVALMELDSDVPLNQYIWPICLPSPAHRFPAGQEAWITGWGAGREGGVAGSILQKARVRIIDSSPCNRVMNDEVTDGMMCAGVLKGGVDACQGDSGGPLSVVNPSGRVFLAGVVSWGDGCARRNKPGIYTRVTRYRGWIREHSGL

>CDK-like21

MERMRKIKRQLSLTLGRGGGGGGGERTLSDHQDVTSHSDSETGTIRPTSAGVKVRGSSSSVQSLFQSYSRRPRGLGRSLSSYLNHSSRLEIVHEDVKMSSDGENEPTSSSDDVQSPVRVRLRTKKISTEDINKRLSLPADIRLPDDYLEKCNVIGPALFEQPISRRLRRVSLSEIGFGKLETYIKLDKLGEGTYATVYKGRSKLTDNLVALKEIRLEHEEGAPCTAIREVSLLKDLKHANIVTLHDIIHTQKSLTLVFEYLDKDLKQYLDDCGNVIHMHNVQLFLFQLLRGLAYCHRRKVLHRDLKPQNLLINERGELKLADFGLARAKSIPTKTYSNEVVTLWYRPPDILLGSTDYSTHIDMWGVGCILYEMTTGRPLFPGSTVEEELHFIFKLLGTPTEESWPGIGSNEEFVAANYPQYPPENLGDHTPRLGSNGVQLLSKFLQVSLTRAEPPQWGSRDVSPQQPPPTSPERRHSLRSTQRQDEEKSSAARRIRMTSSVVLLLLDLSVRAWASHQHFHPHSIKIPGDITLGGLFPIHARGPHGLPCGELKKEKGIHRMEAMLYALDRINGDPALLPNVTLGARILDTCSRDTYALEQSLTFVQAIIQKDTSDIRCSDGRPPLIRKPERVVAVIGASASSVSIMVANILRLFEIPQVSYASTAPELSDNNRYDFFSRVVPPDSYQAQAMLDIVKALGWNYVSTLASEGNYGESGVDAFVQISREAGGVCIAQSVKIPREPTDGEFDKIVNRLMETSNARGIIIFANEDDIKQVLEAAKRANMTGHFLFVGSDSWGAKSSPVMELEDVAEGAVTILPKRASIHRFDQYFMSRSLENNRRNIWFNEFWEDDFRCKLTRPGVEVEPDKKKCTGQEKIGHDSPYEQEGKVQFVIDAVYAVAYALHSMQQRLCGNQSDGGVCGAMDPVEGRSLLGYIRDVNFNGSAGTSVLFNENGDAPGRYDIFQFQMTNRSQPGYHVIGQWTNNLRLNVEEMQWSGGDKSLPDSICSLPCKPGERKKMVKGVPCCWHCELCDGYQYQVDQFTCEMCPADMRPVPNRTACRPTPIIKLEWKSPWALVPGSLSMLGILATCTVVITFVRFNDTPIVRASGRELSYVLLTGIFLIYLITFLMIAEPGVVVCAFRRLLLGLGMAITYSAMLTKTNRIYRIFEQGKRSVTPPKFISPTSQLLITFVLVSVQVLGVFVWFAVVPPHTIVDYEELRPPNPDLARGILKCDMSDLSIICCLGYSIVLMVTCTVYAVKSRGVPETFNEAKPIGFTMYTTCIVWLAFVPIFFGTAQSTEKMFIQTATLTVSMTLSASVSLGMLYIPKVYVILFQPELNIQKRKRSFKGVVQAAQLSHKSIKLSQNGKSKFEPDRTQ

>Throm37

MLTSVATTISLLLLSAALVESRDEARSATASRPPIPTPIPPPIPPPTSAAAVFLQRNSAEQILRSTAAARRRRANAMLLEEMFPGDLERECYEERCSQEEAAEIFQSREKTLEFWFRYTNLSPCASNPCVNGGKCMLERGDFLCLCPPRYHGSTCHLEVSSCVYRNGGCLQYCTDLPGGTAVQCGCADGFKLESDGRSCSKTVAFPCGIQQAAALERGRALWNLSDDANFTAETDFLLDDTNFTEEMDAILQNANVTGNTEDILDGGRGIRFNYSQHTGESLIPRIVGGGLERLGGSPWQVLIRRADGYGFCGGTLVSDRWVISAAHCFQQTAHHVTIGDYDKQRRDPGEQNITVERVLPHPHYHDFTMDSDVALVYLARPVVRGPTAIPACLPDPHLSKYLLKEDNRGLVTGWGATHYLRRSSRFLRKVALPVVSQRACAGSTEQVITDNMFCAGYVDAPMDACSGDSGGPFVVNYRGTWFLTGVVSWGERCAAKGKYGVFTRLGNFLSWIRTTMEKVDRNATLS

>CDK-like10

MEQYSILGRIGEGAHGIVFKAKHVETGETVALKKVALRRLEDAIPNQALREIKALQEIEDNQHVVRLKDVFPHGTGFVLVFDFMLSDLSEVIRNARRPLTPAQVKGYMLMLLKGVAFLHNNNIMHRDLKPANLLISSSGHLKIADFGLARLLSQQEDRLYSHQVATRWYRAPELLYGARKYDEGVDLWAVGCIFGELLNSSPLFPGENDIEQLCCVLRVLGTPTRDTWPEMAQLPDYNKITFKENPAVPLEEIVPDSSPHAVSLLRGFLVYPSERRCPAQQALLHPYFFRSPLPAHHSELPIPQRGGGGGHHLHPRLQAAPADFSVDLPLKSSLVRPAQLRGHASCL

>Throm39

MVILHPNFQSQSDWDNDLALIQLKHPVVMGSKVIPIPLPESNHAITGASGVITGWGYGIHLTFAESLKHLVLPFVPRADCKAEYENSQYMPTVDENMLCTGPSKYGENVCMGDAGGALAVKDPQSGDVVAVGILSYDKSCGQRKYGVYMELSKFMPWINSVIRGDKQESSALRKITMAKMYSWQS

>Histone15

MSGRGKGGKGLGKGGAKRHRKVLRDNIQGITKPAIRRLARRGGVKRISGLIYEETRGVLKVFLENVIRDAVTYTEHAKRKTVTAMDVVYALKRQGRTLYGFGG

>Histone9

MARTKQTARKSTGGKAPRKQLATKAARKSAPATGGVKKPHRYRPGTVALREIRRYQKSTELLIRKLPFQRLVREIAQDFKTDLRFQSSAVMALQESSEAYLVGLFEDTNLCAIHAKRVTIMPKDIQLARRIRVIDMSGRGKTGGKARAKAKTRSSRAGLQFPVGRVHRLLRRGNYAQRVGAGAPVYLAAVLEYLTAEILELAGNAARDNKKTRIIPRHLQLAVRNDEELNKLLGGVTIAQGGVLPNIQAVLLPKKTEKAGKAK

>Histone39

MPEPAKSAPKKGSKKAVTKVPGKPGRRRKKGRKESYAIYVYKVLKQVHPDTGISSKAMSIMNSFVNDIFERIASEASRLAHYNKRSTITSREIQTAVRLLLPGEGTKAVTKYTSSK

>Histone16

MPEPAKSAPKKGSKKAVTKVPGKPGRRRKKGRKESYAIYVYKYKMSGRGKGGKGLGKGGAKRHRKVLRDNIQGITKPAIRRLARRGGVKRISGLIYEETRGVLKVFLENVIRDAVTYTEHAKRKTVTAMDVVYALKRQGRTLYGFGG

>CDK-like11

MEQYSILGRIGEGAHGIVFKAKHVETGETVALKKVALRRLEDAIPNQALREIKALQEIEDNQHVKIDSSFGVRSRIYEIVPQRIQQWQQSPCVAEEMGRRQLERIRKEQQAARLRLTLMEKRFHELEGIIANSKLNQVQHDEEVRKTVPANEGDGDDTDLQIFCVSCSHPINPKVALRHMERCYAKYESQTSFGSMYPTRIEGATRLFCDVYNPQSKTYCKRLQVLCPEHSRDPKVAADEVCGCPLVKDVFEPTGEFCRVSKRKCNKHYCWEKLRRAEVDLERVRVWYKLDELFEQERNLRAAMTNRAGLLALMLHQTIQHDPVTTDLRSAKDR

>Histone22

MSGRGKGGKGLGKGGAKRHRKVLRDNIQGITKPAIRRLARRGGVKRISGLIYEETRGVLKVFLENVIRDAVTYTEHAKRKTVTAMDVVYALKRQGRTLYGFGG

>Histone24

MSGRGKGGKGLGKGGAKRHRKVLRDNIQGITKPAIRRLARRGGVKRISGLIYEETRGVLKVFLENVIRDAVTYTEHAKRKTVTAMDVVYALKRQGRTLYGFGG

>Lectin4

MWPLLAAPLLCLLSAVAATVDSGSFLIFNQDHNKCVKVLHASSVTLSGCEPHAKDQQFRWASADRLLSLSLNLCLGAVELKDWVKVLLFECDEASELQHWQCKNETLFGLRDRDLHFNWGNRNEKNVMIYKGSGLWSRWKIYGTHLDLCAKGYQEIFTIGGNSFGAPCLFPFKFGEQWYSECTKAGRADGQLWCATKANYNSEKKWGFCPTRTSSGWDTDPVTGVQYQRNAQAVLNWHQARKSCQQQGADLLSIVELHEQSYISGLTSTLGTALWIGLNSLDFEGGWQWSNGNPFRYLNWAPGHPSSDPGMNCATLNAGKASKWETSTCTKKLGYICRKGNSTSLPPPPIKQPSFCPNHWVAYGGNCYFIERSKKMWKDALTACRRDDADLASIHNIEEQSFIIAQSGYLATDVLWIGLNDQRTQMLFEWSDRSQVTFTQWMMDEPTHAGNLKEDCVLIQGEGGKWADKMCEKPYGYICKKKASSRAAEGTHLEGNPGCKLGSLRYGSYCYTIGAEKKTFDEAKQACSQDGGHLLDVTNRYENAFLTSLVGLRPEKYFWMGLFKKEDTHTFIWTSQRKVSFTHFNVGMPDRQVGCVALATGTSAGLWDVISCGSKEKYICKKTAMGVQATTVPPTTPTLSCASGWTPVADRNVCYKMYIKTKDLKKTWHEAKKFCMHIGGDLMSIHGAQDLNNAQFHWSDAAWIGLSLLGPNEGFVWSDGSPVGVTPKPEPVISMPTFNTTEDGWLLYNDSHYFINMDEQPMEAARAFCKKNFADLAVITGESERMFLWKQIMKGPHDQYYIGLTVNLDQSFSWLDETPVTYTAWEQNEPNFANNDENCVTIYKNMGFWNDINCGMELPSICKRSSVFVNATVMPTAEPKGGCAPEWLAFQGKCFKFVGADKKTWLNARDYCKNQGGNLVSIVSEQEQAFLTTQMLTYKEDLWLGMNDINWEMTFVWTDGRGVSYTNWAKGHPTTTPEGRYFSLNEDFDCVMMVGSVPKKMGLWKVDDCGEKRGFICKRNLDTQIEPAATTVSSKAFYKLGNDSYKLLTQKMRWDEARRQCQADDSELASILDPVAQAYVVLQISKFKEPVWIGLNSNVTGGRYKWVDKWLLSYTKWGKDEPKSNYGCVYMDVDKTWKTAACNNTYYSLCKSSPDSAPTEPPQLAGRCPETKSKANWIPFRGHCYAFLSSLVDNWAHASVECLKMGASLASIEDPQESLFIQQNLELLQDSSKSFWIGLYKAHDGRWLWIDNSAVDYTNWKAGMPGSESCVDVHSDSGKWKTTSCSRYHSYICKTNKAHIMEEVSNGSAGISVAVVLVVLALAGLVAFLLFRKKMPVPAPSGESTFINKLYFNNPIRTLVDTKSLVGNIEQNEQA

>Histone23

MSGRGKGGKGLGKGGAKRHRKVLRDNIQGITKPAIRRLARRGGVKRISGLIYEETRGVLKVFLENVIRDAVTYTEHAKRKTVTAMDVVYALKRQGRTLYGFGG

>Ubiq15

MQITLKTLQQQTIQIEIDPEQTVKALKEKIEAERGKDNFPVSGQKLIYAGKILQDDTQIKDYKIDEKNFVVVMVSKVSSFNIRLRQHLLPPHLLLPKHLKLLPRIVVPHQQAPLQPQPRPQPQHPPLQLSSPPRRRLRKSQVLQQQKPLNQPVTGAEYEAMLKEIMSMGYERERVVAALRASFNNPHRAVEYLLTGLPSSPIQDTNPPAHTPASGPAEAPSLAEGENPLAFLRTQPQFLHMRQAIQQNPAMLPALLQQLGRENPQLLQQISQHQELFIQMLNEPVGEGGEAPEVGEIGAAGEEGAPVNYIQVTPQEKEAIERLKALGFPEALVIQAYFACEKNENLAANFLLNQGLEDD

>β2m4

MHVDSVCVFNSREPNDIEYLYIYNYNKLKLLSFSSTLDRFVGYTEFGVKQATYFNNDKDIIADARTRKERTCLHNIPIDYESALTKSVKPYVRLHSESPPEGSHSAMLVCIVYDFYPKQIRVTWLRDGQEATGEVTSTDEMPDGDWYYQVHSHLVYTPKSGEKITCVVEHASLEKPLHVDWGNARLAQPPRSHLFPINPGVSVERNPSLPKSEKNKLAIGGAGLTLGVVLSLGGFLYYRKQCRGRILVPTD

>β2m3

MLAAPQLQARRAVPVHPPSLRCIAYFGFLLTRLVCISELLSHQRLACVSNLSWSTSEERASAEEAAALLPGQRTLPRAHLLLMGRLTHGRDGEWTLTDGSGSIRCEVLSPSPLWMGRLVFLPSWHYIPHDAPGQAQPEAPGYVEVIGSPVLLCPGPAQKLSSSPVAIGEAVALIHDRVPGLRLSIFGQVTSVCPLLVISGTSFFFLTLSDEMHTLPVLVKKNGKLWWAQCVSLGESVCVTALRVCVLRPWGEKNILCATDHSEIHKTHDRRRPPDEDTPPDPPPLPPTSAPRRCEGEPDGKESQVISYQGTVTEVVSAGAGLYVMDRKLGLCLAYQPELKRKLRVGDALALHHVHFLYQPCPDFPPSMLCTCLRSTVTVTKFSAVLASDSDRNCPDDGVLQRLLVERNPDVSQYLWTCHLCSQLRHSLLPSTSPQCTCLLSWKLMEVAFGQGQRRRRDIYAEMLDKPHTCPLTQYDGDSVQLQYVSLSELSDSLQKDCWASLCLSSLLPPDGGGLTRAQMNAALAWSSRTLRSDPKHKLQPGETLSIYLQFSQESIHILSPSVAMEMHLRDKGEESGEEEEEKETVARKRRRTAEDPEPSRPLRLSPVTATTCVSVVLRVEQKTGVMWREDAEASFSVKATVIGPVKAWGRDPKNRPMKDFETGNEKKVTLLCSGASARWFPLLQPGRVYRLVASGTHDVGVLAADAGPRWSQDVPLPVQPDWKFHTLTQLLAHACHCSACPTLSTVSQVLDGSADVVSFQGFVSDRINLNDSTRDAGHTNIGVRLTVCDQSGRSLHVFLNFNRNQYPPGLLPGNTLLMSAFRRRVSRSGRVYCSNIAVSWVAVLRVRDERLEDRPPAPIMHLALWTQRSRTVGRVKGHVECDDRLLDFLLLCAQGVNSLFLTCNIDNQAKEAILGIVIGSKSTSALELSVKSNRVRQKLQVNKMKTLLVFLVLLLRCGDAAADVIHTDMLMVGCSETEGAVYFSLDDEDIWVVNFTANRAVDTQPSFVDHVDFGKDMFQWAMQNLIICRINLNITQKALKDQPLESGNVALGEKNSLVCHVSGFFPAPVKVHWTKNGERVAGAVSRATSAFPDKDGSFHQTFQLDFTPAQDDIYSCVVEHPALERPRNSFWTVKVNQPGIGATVFCALGLTVGLVGVATGTFFLIKGNKCN

>Throm52

MERHTNCSSSSDEEDDDDDDDSKEHKDARVLQSTFITYASHTGGDITGLQRTGQYVSELFQSGALTCLICIASVKRTQPVWSCSGCFSLFHLPCIQKWARDSVFLLCSATDEDFGRKQHPWPCPKCRTEYSHAATPTRYVCYCGKLEDPPADPWLAPHSCGGVCHKELKPSCGHACLLLCHPGPCPPCPKMVSVSCLCGKATPLPRRCSNKAWTCQQPCGKLLPCRQHTCSQPCHSECSPCPKVSVQKCMCGRQQSERPCAGPKWKCDRTCGATLSCGNHTCELVCHDGRCPPCPRSLSRSCPCGKAKSSLPCTEEVSLCGDTCERPLSCGKHTCSMRCHRGNCDTCRQEVEKECRCGKYRKLMACHKEYLCESKCSKSRSCQKHPCRRKCCPGNCPPCDQICARTLGCRNHKCPSVCHRGSCYPCPETVDVRCSCGAASLTVPCGRERSTKPPRCKELCTAPSSCHHAARERHRCHTGPCPPCAQPCVLPLAGCPHTCPQPCHDHVLLKSQQVQLSGPWEQPSEPAFVTKALPCPPCQVPIPVSCFGEHEVSEVPCSRQGRFSCKRPCGRPLRCGNHTCGRECHLVDNSNKCDDCEEGCSKPRPLLCPHACPLPCHSGDCPPCHQMIRQRCHCKMSAIYVDCMKMTMADEETKIALVSCTNQCPKELSCGHRCKQVCHPGACALECHQKVKLRCPCRRIKKELACALSSSCVVECDHNCIEQQRKVSELKEAEQKAAEEEEQRRQQEELEAFTKRRGGRRAKKRGRRDEDDEEDGGWIGRWQRRGHVARGGGGGGGGVGGPSSCWSGGSRADFSWRVGCCEEAEEGHVLVAASHLQRGNGYGMIGGHVSHSSPLPSPELIADREHGYCGNRTIPSLPVDASQAPPTPPITTATEFLTSVPSLSSTWLTGATSASDTGGCQPLVEPQCHMLPYNQTWLSSAAAVVKSSEVDMLLRFFSYLSRLSCYKHIMLFGCSLALPQCFHNDATQHRWVVLPCASFCEAAREGCEPVLQMFNASWPDFLRCSQFSQATSSSGVPPACYTPRQAVGKPSACGGKENFLCATGICVPQKLVCNGQNDCDDWSDEAHCVCSDAQFRCQTGRCLPPLLLCDGYDDCGDLSDELNCVCDATREHRCGDGRCVSRDWLCDGDHDCVDKSDELNCSCRSQGLWECRNGQCIPPAFRCDGEDDCKDGSDEELCMREQSGCEPISLELCMNLPYNHTRFPNFLGHVSQRESSVSWESSLFPALVQTGCYQYLMFYACTLLVPKCDPLSLSTVPPCRSLCRTAKEKCESVLGIVGLQWPDDSDCAQFPEEGGNLTCLLPEAGVDECSPSHFKCRSGRCVLASKRCDGLADCDDRSDEDHCGCTERALWECPGSGVCIKTSMICDGFPDCPLLADEVNCLSLSQSSGSILTVLRTADHYHVCADEWNHQLSKLTCDQLGLGVPASVSMVPEQGSPPGRRRWLHVHPEWEERKGLALQARLEKRSHSCHSGKRVAFDCVRDECGLRPAVGAHLSRRKRILGGRVARQGAWPWQCSLQSGQSGHVCGCVLIGTKWALTVAHCFEGRDKAHLWNVLLGLNNLDHPGVHSQRRAVRAITVHPRYNRAVVDYDISVVELDSEVEETEWVRPVCLPGPNQIPTPDSYCYITGWGHMGNRMPFKLQEGQVRIISMSQCQSYFDMKTITARMLCAGYEAGTVDSCMGDSGGPLVCEELSGGRWTLFGLTSWGSVCFSKVLGPGVYSNVSHFTPWIRQQIFMRTYLPD

>LEAP2-1

MQAEGKVPLRRHSLYLLMLLLLLLLSQQVCSGPLASPVQSPYSAHKRVARMSPLWRIMNSKPFGAFCQNNYECSTGLCRVGHCSTHQRPPSEPLND

>Chem10

MKPQVVALLLLALVCLQPAAGEKLISCCLDTSNKCFPRQLVVSYYHQEAGKGCQHSATVFMSKAGKRLCAPAPHKSKCVQELVAFLDKRSKSKKQ

>Ubiq18

MLCVIFQVTEDEKVSTVKELVSERLNIPADQQRLLYKGKALADEHKLSDYSIGPEAKLNLVIRPAGERTGASGTTAMCSSNGSITQAGVWQSVSTILAKHFSPADAAKVHEQLIKDYERSLRQLSLDDIERLASRLLHPEVDDMDTTSYMD

>Throm49

MIPIVLASVLIAAALGCGTPPIEPQTSRVVNGEDAKPHSWPWQISLQYERDGEWRHTCGGSLIADNWVMTAAHCINKKLSYRVFVGKHNLVEEEVGSKAILPEKIIVHEKWNPIFVAFGNDIAMIKLSEPVTLSDQVQLACIPAADTLLTNLYPCFITGWGRLYTGGPIADKLQQALMPVADHPTCSQPDWWGIAVRTTMVCAGGDGIVAGCNGDSGGPLNCKNMEGTWEVHGIASFVSGLGCNYEKKPTVFTRVSAFNDWIDQHEVKMGLSVWAIAAIVCNSAVGVLILILFVILYKACQVPSKPTPVFTSEPAKTQPKPEHKYLLAA

>Histone30

MPEPAKTAPKKGSKKAVTKSASKPGKKRKRTRKESYAIYVYKVLKQVHPDTGISSKAMSIMNSFVNDIFERIASEASRLAHYNKRSTITSREIQTAVRLLLPGELAKHAVSEGTKAVTKYTSSK

>Histone8

MSGRGKTGGKARAKAKTRSSRAGLQFPVGRVHRLLRKGNYGERIGAGAPVYLAAVLEYLTAEILELAGNAARDNKKTRIIPRHLQLAVRNDEELNKLLGGVTIAQGGVLPNIQAVLLPKKTEKAAKSK

>Histone27

MSGRGKGGKGLGKGGAKRHRKVLRDNIQGITKPAIRRLARRGGVKRISGLIYEETRGVLKVFLENVIRDAVTYTEHAKRKTVTAMDVVYALKRQGRTLYGFGG

>Histone28

MSGRGKGGKGLGKGGAKRHRKVLRDNIQGITKPAIRRLARRGGVKRISGLIYEETRGVLKVFLENVIRDAVTYTEHAKRKTVTAMDVVYALKRQGRTLYGFGG

>Histone37

MPEPAKSAPKKGSKKAVTKSTGKPNKRRKKGRKESYAIYVYKVLKQVHPDTGISSKAMSIMNSFVNDIFERIASEASRLAHYNKRSTISSREIQTAVRLLLPGELAKHAVSEGTKAVTKWGARPPEAEPKKPRHRALQQTLHGQTEERQHGPQQHGPDTGPADPHHWAQRGGEDTGPRPTAEQAGRRESATENNRATTDGPKPQ

>Histone19

RGKGGKGLGKGGAKRHRKVLRDNIQGITKPAIRRLARRGGVKRISGLIYEETRGVLKVFLENVIRDAVTYTEHAKRKTVTAMDVVYALKRQGRTLYGFGGUTLKPSIGDPTQRPFUGPPN

>Histone29

MSGRGKGGKGLGKGGAKRHRKVLRDNIQGITKPAIRRLARRGGVKRISGLIYEETRGVLKVFLENVIRDAVTYTEHAKRKTVTAMDVVYALKRQGRTLYGFGG

>CcAMP1

MRCGVVRLQSSSATAAEAKISGEKTAAATGFSFEFTDQQREFQQLARKFAREEIVPQAAEYDRTGEYPLPIIKKAWELGLMNGHIGEEYGGMGLSIFDNCLITEELAYGCTGVQTAIEANSLGQMPVIIAGNETQKKKYLGRMTEEPLMCAYCVTEPGAGSDVAGIKTRAVKTGDEYVVNGQKMWITNGGKANWYFLLARTHPDPKCPTNKAFTGFIVDADTPGIQIGRKERNMGQRCSDTRGITFEEVRIPKENVLLGEGAGFKIAMGAFDKTRPPVAAGATGLAQRALDEATAYALERKTFGKVIAEHQAVSFLLAEMVMKVELARMAYQRAAWEVDRGRRNTYYASIAKAFAGDIANQVASDAVQVFGGNGFNSEYPVEKLMRDAKIYQIYEGTAQIQRLIVAREHLAKAKK

>Throm8

MLVLVSLLLTGLGCLLAAPTGREVYRMPQSALKALSDRGTVVLEAAMTSALSALDRASSERSRVEAGCRGCAPCLFQDCGQPSGSCSSPANALTEPSCDVISKAQKLQDEGERSWALSQACAFYQHRCQAGNSDKESCIRIMGESCSARVLQCSLLNTMQNLEPATQTHVQAVCGQRSSSARNVTQPRSRIVGGSPAPPGSWPWLVNLQLDGGLMCGGVLVESSWVVTAAHCFAGSRSESYWTAVVGEFDITKTDPDEQVLKVNRIVPHPKFNPKTFNNDIALVELTSPVVLSDRVTPVCLPSGMEPPTGSPCLVAGWGSLYEDGPSADTVMEAKVPLLPQNTCKNALGKELVTNTMLCAGYLSGGIDSCQGDSGGPLIYQDRISGRFQLHGITSWGDGCGEKGKPGVYTRVSAFSDWIQVEIQKSFGSREPTCPELLKTTEMTEEEQRSEFITLCRFYTLSCPTGQSGGSCSRMAEDKCLTRFKKCQLRSFLQTLLDLLQSAEDYIRDKVDLAFFTQTLPQLVEHIYSTAFPHSRDKRDVVPIEEQQGRPTEQGSPSVPPALFKALGPSVEDWENYLVTIAEDMEARPNDTSAENSFESSRREEKRFLQAGDDSVLRLQKEYQNMISALRSKLDSKAAPPSLRMDPSDPHQRSSDPLFLPTSSPRLQIDPGPSSSPKSQKPWSLLSALMSEIEKLKTASETLTSDESQNEAFVTSTEGTWDKAVDLGELAALVSTAVLPIHMAKTTSQPHTLHASVPFSLRRRQRSILRKRQTPGPGGKVCPGVRETSQQVSQVRASYHWVLNIPNKNLHMSFQEVLVDLSSKNDRGLYQARVRAVVGGRPLTFYSLVGLENESFYRSVPRIISLALEALKT

>Lectin8

MALPRCAAGRHVFLTLLLLHWFGFGASSSMVNMRKVTHPTVRQPLAGEAALPCVFTLPNGSSSQSVRLIWTLVRLPAGGQDSPLEHVVLSAQGDVIKVNEAFSGRVRLPGYAADPLNATMEISSLRTNDSGTYHCQVVMGNKYERDAVLLVVYGVVFHYQAPSTRYALSFADARRACQENSAQMATSGQMWAAYYDGFAGCAAGWLDDQTVRLSLSSASDRCVSLGGQLATVGQLYLAWRAGLHSCAPGWLADGSVHYPVSLPRPDCGGNEPGVHTVTLNITSDNTTALYDAYCYRGKVENTDTISQIYTSLWKPWSYLTDSSDESGNSPSNWTGLVDLEEEEESLLSVNASSDPWSSESYVSLQISPGQSIDWGEPFEPDSPEFLRPPVVPTPAEKKVISGIIESFWKPWNYLVGTGGGSEETTGTPKGTAGEEDVKKKKKEEEDEEEDRGNMTSTASPALFSWGSFLLSGTPKDDTTPANEELSAHVTPSSTASNNSLTTRTTISEEPPQTRPSPTSTPVPETTSSRGETWPRVDTETTTQSSDKREVTVMPRAGSRGGRGRGKKNRGEDRSRGEDKGRGDDEGSGEITAAEAKGEIQVSRRPVGTSKPRERSRSRERGHRKGQTTTTTTTSPTTTTEPSVMSTSQIVDLSASKLLFTVSTETQSSSIQADLDPISSTSSSSSSSSAPLPSSSPSASSSPSASSSSSSSLSSSASTSSSFWTSTSTPSPSPSPAHTAQPITTLSPSATSSPPLTLESGGPPAFPPADNQNSSANLEALLDWVPVESVAVLNISLDPPPEEEEEPAWSQTVGSGSPLPGNMEEEGSRFNVSNITLVPADVDDCQSEPCENGGTCIDKIDSFLCLCLPSYGGDTCEKDIEGCEHGWRKFHGHCYRYFTHRHTWEDAEKDCREHSAHLSSVATANEQEFINGLGHDNAWVGLNDRTVEDDFQWTDGNELVYENWRESQPDNFFAGGEDCVVTIAHEDGKWNDVPCNYNLPYICKKGTVLCGTPPAVENAHLVGRRRSHYDIHAVVRYQCSEGFYQRHIPTARCRADGSWERPRIICAKSRRSHRYRRHHRRERRERRGHRRHGEGHKARENARSYD

>Chem11

MKLNLILLCLSLWMTSVLTTHGPVSDCCPGLSSTTVSLNRIKSYVIQSVGACSFKAVVFYTHGRKRICADPNSCWTQRAMFKVDGGALRRKEESNDKSTGASGATVPCTPSRNSQGKKRRRGKLLRMKKGKTRV

>Chem13

MRFDTPLILLVLSCLSLAMAQVSYDDCCLTYVKELGKRTQARAVKYRIQETDGGCNIRAVILTMWKGRVYCCDPQETWVQELMANIDKKAVKHVKKTAPKRQKSRLIS

>Lectin9

MRWPAVLLCRLDSPFTLYNKALSSCLLKKSSRCQEVRWTSGNRIFVTSTKKCLGSQGKSVGSEVNAYDCDERSDLQKWECVNGTLLALKDQKPALYIELKSDGGIALSRTTGPGNSFTITGTANGACSRTHRVLYSIDGNAYGKPCMFPFLYKDRWFGDCTPYDSSIKRPWCAIGTKYEHEQWGYCPTTSTEYWKKNHITGAYYQLNAKAVLSWAQADESCKQQGATLVSITDTNEQAFISALLGNVKNKLWIGLVLNPDHGWQWTDYKPMRFLRWSGGFPLPNPGHNCALLDSAGQHRWESFPCSKKIGYICYKPGAPPLPPQAEQGFCSSPWIPYNGHCFHLVRTPKTWSNAQKECRKEEGELVSMRNLEDHSFVISQLGYVSTDELWIGLNDRRTEGLFEWSDAADVYFTSWEYGEPTLMTDDEDCVLIRGENGNWADRSCDEKHGFVCMKQSSTHHQPDEVHIDIGCKAGWKRHGSYCYFVGAETKTFDEAKSDCKATNSYLADVSNGVDNAFLISLVGARPEKYFWLGLSNQKNKDEFVWTNDNAVSFTHWNNEMPGSGQGCVAMTTGIFAGLWDLLPCDNKEKYICKHLAEGAVLTPPPPTLSPPQCAEGWIRVGTRNVCSKFFTGPRSFEKTWFEARDFCLAIGGDLLSIHSASELFVARHGKAWIGLHIADPSTGYVWSDGSPVNFLHWQEGEPNNFQNSESCAEFRMYRADDTGSWNDVHCESYNDWLCEIRAGVTPRPPPNDTAAAEFNTTSDGWLEWRGSQYHIHRVSMAMEDARHYCKQHHGDLVTINSEAENIFLWKQISRTYGTYYIGLSVDLDGSFWWMDGTSVGFQRWDENQPNQNSYDENCIAMSYFMGLWRGANCGEEHQFICKRGTTAPVNTTAAPTVAPKGGCPLKWSKFDSRCYSINSNRKTTWEEARKQCYSMGGNLASIPTRRVQAYLVTKMAEVAADLWIGLNSLKQDAFFWSDGKPRRYTNWGYSESCVIMTTNPTNGIGKWLIKSCNNTNGFICTRSLDPGLPSQSEPAVPNIFVSLGNDSILAVPFNMTWDGAKRHCEGNKATLASLRNEWTLAYVELLALNLNSPLWIGLNKEKTGGYFRYIDGWHMNLVNWAEGEPSQDRPCVYLDVDGKFKTATCNRTAYTVCMQSTDVPPTQSANYPGVCPDDTMADYSQSFTWLPYKSHCYMFVTDEIEWPDAATSCARHGGYLASIEDPEEQAFIQSNVEVFQDSHSSFWIGLYKTHKGTWLWLDKTVLDYTNWGADAAERDYGEMRTSDGEWSSGLKWNDRAYICETPKVMPRDKDPESQAHGGGDAGSRGHTSLIVVLIITLTSALAVVAFFLYKKSPRSLPTFDNPLYFGRERTQPDVVDTNKLMEKAEEEIPEPIIAL

>Neuropep4

MVVGEEVKRALYGVGQMREAMWRNELKHEHLMNSLHHSGEKKKGAAQLAQDVTEKLREAEEQCGASLQSQWDECRPCLEDACKTFFTNTCRRGFATFQTKVENFFRRVSRRFSFSEAAVEADDILVNQDPDRPDAEAVRIQDAFARLSRKVGLLVNSSAALASRMGDGLDRAVRRALLSAPSATVAPDDPARDSGFLQGVGLEEVLDSFFDFGRSVVEEFGAVSPPRNSKLCRDLRKQSSECWRLQNQCEACQGPLRTECPGVRELHVELERVSRLLGVSREQYDEVLSIVRLHAYETLGWLSNMAAELGWVARADTQNIFRITRVVPKSQDKNISAGETQVEGNVLNSPSLVFSVPGELNVHDPTFIRYVAQEALERYKEMISMASSSKATLILLIYGIFMHDSVYCTPLGLSYPKIRLDNDAFDEDGNSLGELGFDGDHLALRSAPSLNDDGGGGGYTLYYPTDKRAERHAEEELDRALREILGQLTTRHYLHSLMTMRAGSEDNSMEDESEPLSKRHSDGIFTDSYSRYRKQMAVQKYLAAVLGRSPARSVVAVRCEQQKAPALKEPLEEVDPQEQKDSFLHHPTVPGVALLPKREPAGDFLNPQHQPNNNLMTWTNRRRGVLA

>Throm13

LAGVRSFAAHQKAATRIIGGQEAWAHSWPWQVSLRFATMPACGGAVLAPLWVVSAAHCFKRYNKASFWTVLAGKHDLDDPDEEGQQVADVSAIVSHHRYRSGSKEFDVALLRLERALAFDRFVRPIRVWMAPLPTREKCTVTGWGATRENGPRATRLQEVNVTVMTLDLCQSYYKSRIRTRMFCAGQEAGGVDACQGDSGGPLSCYDGGRYRLAGVVSWGVGCGRARKPGVYTKLQEHAGWIADVIGKNLAGGSVVGPDTQRRCGWRREAVCRNLPGPAGLRVPRAGPARAENLTEACPGAWPWQVSLQANGVHYCSAVLIRRRWVLAARHCAVSAGDDVAVLGGHDLGLSLSQTVPVDRVFDPPQEDGFPPKNDLSLLRLAVPARLGAGVTPLCVAEEDEELDDGWRCISAGWGATSATAGLNAERLHHAGVPLVERAKCRAEWG

>β2m1

LESPMVQVYTHQPGKLGTPNTLICHVSQFHPPDITIQLFKNGKEMFDVVESDLSFGKNWHFHLTKYASFTPTREDKYMCRVTHLSDPAKF

>Throm59

GDGDDDDDDEEEKEEERKVAKKGALRPQSVSSPLERDRGKRGAFTEPSRAUDAPALRVAVTAPGRPFGDGRMTSQRPPPIUVGSARRRRSAGLRUVAAPHRSARLSFTMPPPPPPLLALLLLAGLRVVCADAGNVTRGARRGQRHSGNFAGRYELAGVRSFAAHQKAATRIIGGQEAWAHSWPWQVSLRFATMPACGGAVLAPLWVVSAAHCFKRYNKASFWTVLAGKHDLDDPDEEGQQVADVSAIVSHHRYRSGSKEFDVALLRLERALAFDRFVRPIRVWMAPLPTREKCTVTGWGATRENGPRATRLQEVNVTVMTLDLCQSYYKSRIRTRMFCAGQEAGGVDACQGDSGGPLSCYDGGRYRLAGVVSWGVGCGRARKPGVYTKLQEHAGWIADVIENQDVAYDLKPSEERRCGWRREAVCRNLPGPAGLRVPRAGPARAENLTEACPGAWPWQVSLQANGVHYCSAVLIRRRWVLAARHCAVSAGDDVAVLGGHDLGLSLSQTVPVDRVFDPPQEDGFPPKNDLSLLRLAVPARLGAGVTPLCVAEEDEELDDGWRCISAGWGATSATAGLNAERLHHAGVPLVERAKCRAEWGR

>Neuropep12

LKDKNPRRSGDTKKFRGHTERNCCRLFFLFFLVGWVETTFLRSRTLHCAFLLFLLLLILLSGGRUKCMRGGGDVKRSRSQVHRTRQAAPGSHPSSSGSGGSSGSGLRFPAWSMASSSKATLILLIYGIFMHDSVYCTPLGLSYPKIRLDNDAFDEDGNSLGELGFDGDHLALRSAPSLNDDGGGGGYTLYYPTDKRAERHAEEELDRALREILGQLTTRHYLHSLMTMRAGEDNSMEDESEPLSKRHSDGIFTDSYSRYRKQMAVQKYLAAVLGRRYRQRVRTKGRRLAYLUCSPPTHTLCACIPYKCMTPPPSRLSSPPNPHTRTRSQCHPPIIHMIUQSSGLRLCVLLRHDVIMYDVKTLKUIFYUUYRFPHPHFFFLLREKKGTULTLLLCCCQIDEHLKCKGVGUIMRMLWGLFLSFSDSSUULHIDALGQNDPFUSKKKNQPPRPQTNTUMDSIRNKSQUKYHFRTVUHKUTRPGSKUPIUKUKNCULLRUSUHFKECFFHTPPPSPFWTPVAQUTUPGSNUPIUKUMNCTSSYHIFVMKLLSVVLTMTYKHSVUPPPPHILNNLGRLNTVWVKLTQQNDRUSUQEGUTEQSMDEYLFIFMFQDVUKKDHNCAPSKLCAUEEKKKSSIHIYIHIKIYIYAEKUIDKKRQSKYUMLYLFLUAIFLSVVCFLDKNRPKEVVSMLHTRVUVCVRVCVKHSIEKLULLLFAFLFLQGCLNDAHTCTCVENMCACVCVKSALGKKECKICIYFVVILWNICMLLYCWRKVCVVCTURGLTNCSAAGCLGVRKIKNILPVYIYDVCPLPPPGDICSQSPKGUISVIIFHLIIRSVLMQGVGWCANYVRFVNQSGNFVWEFGCRPADKEDMGNQKLLNKREEGGHISKCKTPPKKVKUDFGKKYIPQNAVQULLHLLLHNLVMLFAHRWLNSKVGAHQICWDAEGGAUKLLVALVKIWVQFFFWPPITGDAKAKKKTKKKKNRYTFKUCIFFNHVUUNYKKGEGCASYVDLUGLSFVISDFVSGIQAKRRHKTETITEKEKVGTLPRIFSSAFWSKSIGTLDIAHLPVKATIUWCVPNIFIFGLVFSRSNRGKGVASTVAMRKEVRGLRSWWRSFKSTFFKTALHRCNDFGWKCPAQFLKPCLHDSTKQSGVEPVSAGDNLRLLYNIEGWRTSHLGKIGLPTMHLINCPFSVIUNQQCTWMHTUPFFRFGQMLIVFAQEHFGEGVHRLFLKGKFFQDCQSSSGFCVVURSFUDVLLNNDQHFLVNNFUERFYYVVTCYKNVGLVKUGRGGAPTLAEQURSUKQNCIDDTMMRLRQLLFHUKKKKKKIPKTKTEHHYTPUGAUHYFLEPLCLELRKRRLHSKVVTUQEKMVLHHFSKINHEHPEMTFAHPQRGISDNLGIPLSGDVKRAPPFVERAPSEGIFPMLRSFKTGRKRARERPSPQNSALPDSLDMAUVFLSAWSAFIGRSWRQLLAFGGSLRCEKKKKNQUNQLKKUIRFKRKUMKKKIAASDRWQFQWPYNAAKSMIESFPVLITWSWPKRRRISFFLFVKFFFFFHAUPMIHUVQIYINIYEKKNUMYMSKEQEMCVCACVCACAUFRGVSVSVGVIQNDQCLLLLMIRWCYULIUFWLDIKSHKKKKXXXXXXTPPPTPPNTPPHPPHQTPPPPPSPPPHTKQGVVMVVGUQMPVVIFLKAPNALLQCUIUPULGKQCVCKVNPSRTUATTHRSPPLPELWEEGGGGGXGGGGGGVGLCSLSIKVLYDLYEUIFLUNNISSSTSNLUTANHASGEALHSHARALLKENQRTKQKVELTVSLMKSETPNASKQPGTQTRRTWNGTPKSUNWGGKKRHKVYFVTLSIQTDTSRIFAVQHSQIQLFVDFLVVFYSPILDUVDFFSHGSPGLFTLHLVSSAFTAGRGTWTMGILCKLDHTFGSVGSAVEQKIARMLPPLLSRUHSEITPPNPFLFNELPDVTTPPLRSGTUVLLSAVUKGSKKEDQTLAVPARADETKPQTHRTKPQEPKWHANKGLTLAPHLSKTKPQIQTKETPKVPNGTSNAPHASQTNFESQPGGCAAFNLRRRRTRHGGKGTWKDAREDKQTKGGLRRLDRSKQTLKCTKUNPGTRLQCTRASDTKTSAFLLPLLSSASTVRPSPAGDEKGCGDAVFAIUSVRVCLCSRAQLTQSETCCVATPRTKGTRRCVRKCRHVSTHQEVGGACRLLECYLUPP

>Neuropep13

ADEEEGAKGGGEGGILKDKNPRRSGDTKKFRGHTERNCCRLFFLFFLVGWVETTFLRSRTLHCAFLLFLLLLILLSGGRUKCMRGGGDVKRSRSQVHRTRQAAPGSHPSSSGSGGSSGSGLRFPAWSMASSSKATLILLIYGIFMHDSVYCTPLGLSYPKIRLDNDAFDEDGNSLGELGFDGDHLALRSAPSLNDDGGGGGYTLYYPTDKRAERHAEEELDRALREILGQLTTRHYLHSLMTMRAGSEDNSMEDESEPLSKRHSDGIFTDSYSRYRKQMAVQKYLAAVLGRRYRQRVRTKGRRLAYLUCSPPTHTLCACIPYKCMTPPPSRLSSPPNPHTRTRSQCHPPIIHMIUQSSGLRLCVLLRHDVIMYDVKTLKUIFYUUYRFPHPHFFFLLREKKGTULTLLLCCCQIDEHLKCKGVGUIMRMLWGLFLSFSDSSUULHIDALGQNDPFUSKKKNQPPRPQTNTUMDSIRNKSQUKYHFRTVUHKUTRPGSKUPIUKUKNCULLRUSUHFKECFFHTPPPSPFWTPVAQUTUPGSNUPIUKUMNCTSSYHIFVMKLLSVVLTMTYKHSVUPPPPHILNNLGRLNTVWVKLTQQNDRUSUQEGUTEQSMDEYLFIFMFQDVUKKDHNCAPSKLCAUEEKKKSSIHIYIHIKIYIYAEKUIDKKRQSKYUMLYLFLUAIFLSVVCFLDKNRPKEVVSMLHTRVUVCVRVCVKHSIEKLULLLFAFLFLQGCLNDAHTCTCVENMCA

CVCVKSALGKKECKICIYFVVILWNICMLLYCWRKVCVVCTURGLTNCSAAGCLGVRKIKNILPVYIYDVCPLPPPGDICSQSPKGUISVIIFHLIIRSVLMQGVGWCANYVRFVNQSGNFVWEFGCRPADKEDMGNQKLLNKREEGGHISKCKTPPKKVKUDFGKKYIPQNAVQULLHLLLHNLVMLFAHRWLNSKVGAHQICWDAEGGAUKLLVALVKIWVQFFFWPPITGDAKAKKKTKKKKNRYTFKUCIFFNHVUUNYKKGEGCASYVDLUGLSFVISDFVSGIQAKRRHKTETITEKEKVGTLPRIFSSAFWSKSIGTLDIAHLPVKATIUWCVPNIFIFGLVFSRSNRGKGVASTVAMRKEVRGLRSWWRSFKSTFFKTALHRCNDFGWKCPAQFLKPCLHDSTKQSGVEPVSAGDNLRLLYNIEGWRTSHLGKIGLPTMHLINCPFSVIUNQQCTWMHTUPFFRFGQMLIVFAQEHFGEGVHRLFLKGKFFQDCQSSSGFCVVURSFUDVLLNNDQHFLVNNFUERFYYVVTCYKNVGLVKUGRGGAPTLAEQURSUKQNCIDDTMMRLRQLLFHUKKKKKKIPKTKTEHHYTPUGAUHYFLEPLCLELRKRRLHSKVVTUQEKMVLHHFSKINHEHPEMTFAHPQRGISDNLGIPLSGDVKRAPPFVERAPSEGIFPMLRSFKTGRKRARERPSPQNSALPDSLDMAUVFLSAWSAFIGRSWRQLLAFGGSLRCEKKKKNQUNQLKKUIRFKRKUMKKKIAASDRWQFQWPYNAAKSMIESFPVLITWSWPKRRRISFFLFVKFFFFFHAUPMIHUVQIYINIYEKKNUMYMSKEQEMCVCACVCACAUFRGVSVSVGVIQNDQCLLLLMIRWCYULIUFWLDIKSHKKKKXXXXXXTPPPTPPNTPPHPPHQTPPPPPSPPPHTKQGVVMVVGUQMPVVIFLKAPNALLQCUIUPULGKQCVCKVNPSRTUATTHRSPPLPELWEEGGGGGXGGGGGGVGLCSLSIKVLYDLYEUIFLUNNISSSTSNLUTANHASGEALHSHARALLKENQRTKQKVELTVSLMKSETPNASKQPGTQTRRTWNGTPKSUNWGGKKRHKVYFVTLSIQTDTSRIFAVQHSQIQLFVDFLVVFYSPILDU

VDFFSHGSPGLFTLHLVSSAFTAGRGTWTMGILCKLDHTFGSVGSAVEQKIARMLPPLLSRUHSEITPPNPFLFNELPDVTTPPLRSGTUVLLSAVUKGSKKEDQTLAVPARADETKPQTHRTKPQEPKWHANKGLTLAPHLSKTKPQIQTKETPKVPNGTSNAPHASQTNFESQPGGCAAFNLRRRRTRHGGKGTWKDA

REDKQTKGGLRRLDRSKQTLKCTKUNPGTRLQCTRASDTKTSAFLLPLLSSASTVRPSPAGDEKGCGDAVFAIUSVRVCLCSRAQLTQSETCCVATPRTKGTRRCVRKCRHVSTHQEVGGACRLLECYLUPP

>Neuropep11

ADEEEGAKGGGEGGILKDKNPRRSGDTKKFRGHTERNCCRLFFLFFLVGWVETTFLRSRTLHCAFLLFLLLLILLSGGRUKCMRGGGDVKRSRSQVHRTRQAAPGSHPSSSGSGGSSGSGLRFPAWSMASSSKATLILLIYGIFMHDSVYCTPLGLSYPKIRLDNDAFDEDGNSLGELGFDGDHLALRSAPSLNDDGGGGGYTLYYPTDKSSEDNSMEDESEPLSKRHSDGIFTDSYSRYRKQMAVQKYLAAVLGRRYRQRVRTKGRRLAYLUCSPPTHTLCACIPYKCMTPPPSRLSSPPNPHTRTRSQCHPPIIHMIUQSSGLRLCVLLRHDVIMYDVKTLKUIFYUUYRFPHPHFFFLLREKKGTULTLLLCCCQIDEHLKCKGVGUIMRMLWGLFLSFSDSSUULHIDALGQNDPFUSKKKNQPPRPQTNTUMDSIRNKSQUKYHFRTVUHKUTRPGSKUPIUKUKNCULLRUSUHFKECFFHTPPPSPFWTPVAQUTUPGSNUPIUKUMNCTSSYHIFVMKLLSVVLTMTYKHSVUPPPPHILNNLGRLNTVWVKLTQQNDRUSUQEGUTEQSMDEYLFIFMFQDVUKKDHNCAPSKLCAUEEKKKSSIHIYIHIKIYIYAEKUIDKKRQSKYUMLYLFLUAIFLSVVCFLDKNRPKEVVSMLHTRVUVCVRVCVKHSIEKLULLLFAFLFLQGCLNDAHTCTCVENMCACVCVKSALGKKECKICIYFVVILWNICMLLYCWRKVCVVCTURGLTNCSAAGCLGVRKIKNILPVYIYDVCPLPPPGDICSQSPKGUISVIIFHLIIRSVLMQGVGWCANYVRFVNQSGNFVWEFGCRPADKEDMGNQKLLNKREEGGHISKCKTPPKKVKUDFGKKYIPQNAVQULLHLLLHNLVMLFAHRWLNSKVGAHQICWDAEGGAUKLLVALVKIWVQFFFWPPITGDA

KAKKKTKKKKNRYTFKUCIFFNHVUUNYKKGEGCASYVDLUGLSFVISDFVSGIQAKRRHKTETITEKEKVGTLPRIFSSAFWSKSIGTLDIAHLPVKATIUWCVPNIFIFGLVFSRSNRGKGVASTVAMRKEVRGLRSWWRSFKSTFFKTALHRCNDFGWKCPAQFLKPCLHDSTKQSGVEPVSAGDNLRLLYNIEGWRTSHLGKIGLPTMHLINCPFSVIUNQQCTWMHTUPFFRFGQMLIVFAQEHFGEGVHRLFLKGKFFQDCQSSSGFCVVURSFUDVLLNNDQHFLVNNFUERFYYVVTCYKNVGLVKUGRGGAPTLAEQURSUKQNCIDDTMMRLRQLLFHUKKKKKKIPKTKTEHHYTPUGAUHYFLEPLCLELRKRRLHSKVVTUQEKMVLHHFSKINHEHPEMTFAHPQRGISDNLGIPLSGDVKRAPPFVERAPSEGIFPMLRSFKTGRKRARERPSPQNSALPDSLDMAUVFLSAWSAFIGRSWRQLLAFGGSLRCEKKKKNQUNQLKKUIRFKRKUMKKKIAASDRWQFQWPYNAAKSMIESFPVLITWSWPKRRRISFFLFVKFFFFFHAUPMIHUVQIYINIYEKKNUMYMSKEQEMCVCACVCACAUFRGVSVSVGVIQNDQCLLLLMIRWCYULIUFWLDIKSHKKKKXXXXXXTPPPTPPNTPPHPPHQTPPPPPSPPPHTKQGVVMVVGUQMPVVIFLKAPNALLQCUIUPULGKQCVCKVNPSRTUATTHRSPPLPELWEEGGGGGXGGGGGGVGLCSLSIKVLYDLYEUIFLUNNISSSTSNLUTANHASGEALHSHARALLKENQRTKQKVELTVSLMKSETPNASKQPGTQTRRTWNGTPKSUNWGGKKRHKVYFVTLSIQTDTSRIFAVQHSQIQLFVDFLVVFYSPILDUVDFFSHGSPGLFTLHLVSSAFTAGRGTWTMGILCKLDHTFGSVGSAVEQKIARMLPPLLSRUHSEITPPNPFLFNELPDVTTPPLRSGTUVLLSAVUKGSKKEDQTLAVPARADETKPQTHRTKPQEPKWHANKGLTLAPHLSKTKPQIQTKETPKVPNGTSNAPHASQTNFESQPGGCAAFNLRRRRTRHGGKGTWKDAREDKQTKGGLRRLDRSKQTLKCTKUNPGTRLQCTRASDTKTSAFLLPLLSSASTVRPSPAGDEKGCGDAVFAIUSVRVCLCSRAQLTQSETCCVATPRTKGTRRCVRKCRHVSTHQEVGGACRLLECYLUPP

>Lectin30

VFQQKSICCATRDNAHKMFFLANVRQRESPPYSLFHFEKYNRCSVAGDUIPITTNTGSAEKVMVLIVLPSLQHCHMCMSGUUCPQVAKAHTSEUATQFIPNDCTNVNVSRVDNAFLISLVGARPEKYFWLGLSNQKNKDEFVWTNDNAVSFTHWNNEMPGSGQGCVAMTTGIFAGLWDLLPCDNKEKYICKHLAEGAVLTPPPPTLSPPQCAEGWIRVGTRNVCSKFFTGPRSFEKTWFEARDFCLAIGGDLLSIHSASELFVARHGKAWIGLHIADPSTGYVWSDGSPVNFLHWQEGEPNNFQNSESCAEFRMYRADDTGSWNDVHCESYNDWLCEIRAGVTPRPPPNDTAAAEFNTTSDGWLEWRGSQYHIHRVSMAMEDARHYCKQHHGDLVTINSEAENIFLWKQISRTYGTYYIGLSVDLDGSFWWMDGTSVGFQRWDENQPNQNSYDENCIAMSYFMGLWRGANCGEEHQFICKRGTTAPVNTTAAPTVAPKGGCPLKWSKFDSRCYSINSNRKTTWEEARKQCYSMGGNLASIPTRRVQAYLVTKMAEVAADLWIGLNSLKQDAFFWSDGKPRRYTNWGYSIHRRRPGTFYQRWNEESCVIMTTNPTNGIGKWLIKSCNNTNGFICTRSLDPGLPSQSEPAVPNIFVSLGNDSILAVPFNMTWDGAKRHCEGNKATLASLRNEWTLAYVELLALNLNSPLWIGLNKEKTGGYFRYIDGWHMNLVNWAEGEPSQDRPCVYLDVDGKFKTATCNRTAYTVCMQSTDVPPTQSANYPGVCPDDTMADYSQSFTWLPYKSHCYMFVTDEIEWPDAATSCARHGGYLASIEDPEEQAFIQSNVEVFQDSHSSFWIGLYKTHKGTWLWLDKTVLDYTNWGADAAERDYGEMRTSDGEWSSGLKWNDRAYICETPKVMPRDKDPESQAHGGGDAGSRGHTSLIVVLIITLTSALAVVAFFLYKKSPRSLPTFDNPLYFGRERTQPDVVDTNKLMEKAEEEIPEPIIALUSQANVSHWDTGTVFVLYCTVCAMYSVIUUEKGUVSMTWPCLTPQSWNFUGSGTDTDLMDVSVPLHMTVUNESHAVHCSRWSRLYKWHCFQUQLKGGFKCLRVYENSRESWRAFLHNAAKUEIEFKLHLLSESESVQLDSVLKLALUAASLSLSLSGYULUWRTQFCQSTQLASWLAFSAHFDVDLCLMCSVILLFULEDG

>Lectin29

FLTULFTKADNHLLQTKUTELQSNDFAQCHKLYSIFFLNYTIKLTFRLISFESMMKGGYRSVELTLMSHVUHKRLTHHSLPPLLGPGQEVNGDTFSMKLRKKKGKKKVSSCUEVFLFMVTLISPLLATRAMFAALTGRMLKAGITLAALALLAPMSLCIQLDDSPFTLYNKALSSCLLKKSSRCQEVRWTSGNRIFVTSTKKCLGSQGKSVGSEVNAYDCDERSDLQKWECVNGTLLALKDQKPALYIELKSDGGIALSRTTGPGNSFTITGTANGACSRTHRVLYSIDGNAYGKPCMFPFLYKDRWFGDCTPYDSSIKRPWCAIGTKYEHEQWGYCPTTSTEYWKKNHITGAYYQLNAKAVLSWAQADESCKQQGATLVSITDTNEQAFISALLGNVKNKLWIGLVLNPDHGWQWTDYKPMRFLRWSGGFPLPNPGHNCALLDSAGQHRWESFPCSKKIGYICYKPGAPPLPPQAEQGFCSSPWIPYNGHCFHLVRTPKTWSNAQKECRKEEGELVSMRNLEDHSFVISQLGYVSTDELWIGLNDRRTEGLFEWSDAADVYFTSWEYGEPTLMTDDEDCVLIRGENGNWADRSCDEKHGFVCMKQSSTHHQPDEVHIDIGCKAGWKRHGSYCYFVGAETKTFDEAKSDCKATNSYLADVSNGVDNAFLISLVGARPEKYFWLGLSNQKNKDEFVWTNDNAVSFTHWNNEMPGSGQGCVAMTTGIFAGLWDLLPCDNKEKYICKHLAEGAVLTPPPPTLSPPQCAEGWIRVGTRNVCSKFFTGPRSFEKTWFEARDFCLAIGGDLLSIHSASELFVARHGKAWIGLHIADPSTGYVWSDGSPVNFLHWQEGEPNNFQNSESCAEFRMYRADDTGSWNDVHCESYNDWLCEIRAGVTPRPPPNDTAAEFNTTSDGWLEWRGSQYHIHRVSMAMEDARHYCKQHHGDLVTINSEAENIFLWKQISRTYGTYYIGLSVDLDGSFWWMDGTSVGFQRWDENQPNQNSYDENCIAMSYFMGLWRGANCGEEHQFICKRGTTAPVNTTAAPTVAPKGGCPLKWSKFDSRCYSINSNRKTTWEEARKQCYSMGGNLASIPTRRVQAYLVTKMAEVAADLWIGLNSLKQDAFFWSDGKPRRYTNWGYSIHRRRPGTFYQRWNEESCVIMTTNPTNGIGKWLIKSCNNTNGFICTRSLDPGLPSQSEPAVPNIFVSLGNDSILAVPFNMTWDGAKRHCEGNKATLASLRNEWTLAYVELLALNLNSPLWIGLNKEKTGGYFRYIDGWHMNLVNWAEGEPSQDRPCVYLDVDGKFKTATCNRTAYTVCMQSTDVPPTQSANYPGVCPDDTMADYSQSFTWLPYKSHCYMFVTDEIEWPDAATSCARHGGYLASIEDPEEQAFIQSNVEVFQDSHSSFWIGLYKTHKGTWLWLDKTVLDYTNWGADAAERDYGEMRTSDGEWSSGLKWNDRAYICETPKVMPRDKDPESQAHGGGDAGSRGHTSLIVVLIITLTSALAVVAFFLYKKSPRSLPTFDNPLYFGRERTQPDVVDTNKLMEKAEEEIPEPIIALUSQANVSHWDTGTVFVLYCTVCAMYSVIUUEKGUVSMTWPCLTPQSWNFUGSGTDTDLMDVSVPLHMTVUNESHAVHCSRWSRLYKWHCFQUQLKGGFKCLRVYENSRESWRAFLHNAAKUEIEFKLHLLSESESVQLDSVLKLALUAASLSLSLSGYULUWRTQFCQSTQLASWLAFSAHFDVDLCLMCSVILLFULEDG

>Lectin27

FLTULFTKADNHLLQTKUTELQSNDFAQCHKLYSIFFLNYTIKLTFRLISFESMMKGGYRSVELTLMSHVUHKRLTHHSLPPLLGPGQEVNGDTFSMKLRKKKGKKKVSSCUEVFLFMVTLISPLLATRAMFAALTGRMLKAGITLAALALLAPMSLCIQLDDSPFTLYNKALSSCLLKKSSRCQEVRWTSGNRIFVTSTKKCLGSQGKSVGSEVNAYDCDERSDLQKWECVNGTLLALKDQKPALYIELKSDGGIALSRTTGPGNSFTITGTANGACSRTHRVLYSIDGNAYGKPCMFPFLYKDRWFGDCTPYDSSIKRPWCAIGTKYEHEQWGYCPTTSTEYWKKNHITGAYYQLNAKAVLSWAQADESCKQQGATLVSITDTNEQAFISALLGNVKNKLWIGLVLNPDHGWQWTDYKPMRFLRWSGGFPLPNPGHNCALLDSAGQHRWESFPCSKKIGYICYKPGAPPLPPQAEQGFCSSPWIPYNGHCFHLVRTPKTWSNAQKECRKEEGELVSMRNLEDHSFVISQLGYVSTDELWIGLNDRRTEGLFEWSDAADVYFTSWEYGEPTLMTDDEDCVLIRGENGNWADRSCDEKHGFVCMKQSSTHHQPDEVHIDIGCKAGWKRHGSYCYFVGAETKTFDEAKSDCKATNSYLADVSNGVDNAFLISLVGARPEKYFWLGLSNQKNKDEFVWTNDNAVSFTHWNNEMPGSGQGCVAMTTGIFAGLWDLLPCDNKEKYICKHLAEGAVLTPPPPTLSPPQCAEGWIRVGTRNVCSKFFTGPRSFEKTWFEARDFCLAIGGDLLSIHSASELFVARHGKAWIGLHIADPSTGYVWSDGSPVNFLHWQEGEPNNFQNSESCAEFRMYRADDTGSWNDVHCESYNDWLCEIRAGVTPRPPPNDTAAAEFNTTSDGWLEWRGSQYHIHRVSMAMEDARHYCKQHHGDLVTINSEAENIFLWKQISRTYGTYYIGLSVDLDGSFWWMDGTSVGFQRWDENQPNQNSYDENCIAMSYFMGLWRGANCGEEHQFICKRGTTAPVNTTAAPTVAPKGGCPLKWSKFDSRCYSINSNRKTTWEEARKQCYSMGGNLASIPTRRVQAYLVTKMAEVAADLWIGLNSLKQDAFFWSDGKPRRYTNWGYSIHRRRPGTFYQRWNEESCVIMTTNPTNGIGKWLIKSCNNTNGFICTRSLDPGLPSQSEPAVPNIFVSLGNDSILAVPFNMTWDGAKRHCEGNKATLASLRNEWTLAYVELLALNLNSPLWIGLNKEKTGGYFRYIDGWHMNLVNWAEGEPSQDRPCVYLDVDGKFKTATCNRTAYTVCMQSTDVPPTQSANYPGVCPDDTMADYSQSFTWLPYKSHCYMFVTDEIEWPDAATSCARHGGYLASIEDPEEQAFIQSNVEVFQDSHSSFWIGLYKTHKGTWLWLDKTVLDYTNWGADAAERDYGEMRTSDGEWSSGLKWNDRAYICETPKVMPRDKDPESQAHGGGDAGSRGHTSLIVVLIITLTSALAVVAFFLYKKSPRSLPTFDNPLYFGRERTQPDVVDTNKLMEKAEEEIPEPIIALUSQANVSHWDTGTVFVLYCTVCAMYSVIUUEKGUVSMTWPCLTPQSWNFUGSGTDTDLMDVSVPLHMTVUNESHAVHCSRWSRLYKWHCFQUQLKGGFKCLRVYENSRESWRAFLHNAAKUEIEFKLHLLSESESVQLDSVLKLALUAASLSLSLSGYULUWRTQFCQSTQLASWLAFSAHFDVDLCLMCSVILLFULEDG

>Chem16

WULIMTITSHSLGYNIRIHYFMLRIUCNHRSHQKQRQSLLMLRGRKNKINPKSKINMPLKTVHNILSCQHVAHRNISLHLLISTQLFPHSLKSIMSCRQTAGHHRTDTAFILPTCLSUSGKEKKKKKERKITSSGKAKEKUIGVQRUVAFAEIRHLARRPPFAIIKRPRPRRUHTHTHHQFARRTHRIAALNRSSGSMRFDTPLILLVLSCLSLAMAQVSYDDCCLTYVKELGKRTQARAVKYRIQETDGGCNIRAVILTMWKGRVYCCDPQETWVQELMANIDKKAVKHVKKTAPKRQKSRLISUVRPHPPGEKANRRFDFHCULFILTTRDHDMTKQCLRANGNHKMUIUTVCSMSACFNIVTIKLLSHSNSVWFMFMCIDFVIRSSKDTVVLSVHMFYSTACVQKSQLSMLTVSTACUQLACQSLAFUIVGSLLLLYTTCNFLCFMLSVKGHFQVSVAVUSLFFFMNCSLFAKLPAYAKVELSSITRDGLYIKATSSUIAHKCGRGLTQMSSHPCHYVHIQIQNPYPWVEPAQDQKINSLLLFLUILCVSLFPISQALTYS

>Chem15

KKIVUKTAGDRKURRPUKDGRDNWIKVMAEYSEIATIALLLFPTCYLSEAGLFCSNGDUNKITTUTGHKTHTUGVIVSYYHEMGPSGCRKSSSGLUTVVSGLFAHQFHRRLSSNHRRTTSWKSSVVPAPKMKLNLILLCLSLWMTSVLTTHGPVSDCCPGLSSTTVSLNRIKSYVIQSVGACSFKAVVFYTHGRKRICADPNSCWTQRAMFKVDGGALRRKEESNDKSTGASGATVPCTPSRNSQGKKRRRGKLLRMKKGKTRVUIYLNECPKUMWYNNDLCAVCKSTHPSIHFQIRLSSHGSRHVLEPSPALFEKUTEYTLNCLPANRRDTHRRTTICTHNHTUGQFRVSHUTATHVFGMWEETHAGTIHLSSULAIYRACULASYLHNTGSYLHVSSLMYYVLCCIILGCTLLQCTLQARAALLHSILYNTIQUFTVLYQLCKSVECKTEKESLIPIKKKYHLNLRRQFQPTCNGSMTVDSSEFTKPPIKLFQVRUVVLKVVHFMVALNSANVGKLUVHITRKPQIUSSTUSHTLPHKCSIGWWVGGGGXXXGPIIVIDVCIKENPFTSSKLHUEAAVLFSHIVSKYVWTGSPLNLHLGSQKTSVTQLRTRTYGGCGGLCKTSEYHLSHLWSKTDRISFPPANEGNALAYLSNSSKNGRISDLTAGDSSIDFLPRNVFQKPFFIURALADIGLVPTLLKIQTKKKHFLNLHGLFLVYMERLYURGDPFFFFKKGVFUCRKYEDITTVHVKRCARFRCVPSFWAUQFFPSRYSMLVWVHDLFGSAQILLPFNVTKLRNKYKRNIRWRKVNUNLSNIIFLNLKURDIVHGVFGLLLFFTILHFHATNAPPGLAFGHICLKYFPCNFSLVIYCIIEIIYLICIQQNRCTVENISNVTYTTACKAQEGGPWMUYLVTRCRRTCVSKILRQQLGNPPEKKIWGQKKIKKRTRQINNTSSHMUANLTUHLPRAKAPGTMTATRKRPTKLRAVIAHVGRNQVUSGCLRCGLLKPANVVKYVNLCHLRVWHDAPTVAHTPGNNQTFFLPLKTGQNSPWPSFRPRARVSMAYFSIAGVSCQSTFSCNYNTPQFVNNSQYFILIFAVNPMFQLCAUPEKQIHFLALFFFVCLFVCAFUGAFSRVGALDTELDSSQSUVTNFRTWDLRPFIGFLENNKTHVLYUTTKTCHHCHGRSELPQREEMKPRRTLSDIKAKRGEYLSRKCMNAVSQYQRANIAKSLSNDPHALHYFWGVIRDFLDNHAMFLRFNKKKRIHYSTVUCLWNLKTQFRATVWHSLCWCEEPVTVFYLYLSQLVULTRPWTFRIYDSKQVERAHFLKSPSIVLRCCVAAATKQFFTYUUURMTFSTAIFQQRLFNSYIETQYLPHLKSSSSSSSYFQAIADCIYLDLLLKAGEKUTRPWVFFLREKVTLTEQLAVCKRIQCTNKVSPKTPPLFVVTUSLLKGSARCTHVVVSTTFSCMTACRIIKVHRDIFYLUCMKHEKKKSPLSSRHRLLVEDDEAKHPVRCIAVPYSLHUTIPKUSCARFYAERRGARQPKUFCLNSFFHMKSSDSYYFPGTGULUVSNFTISIISADLWNPMSHFENVSPDLLSAVVSISPSDUUSLSVCHGPEKUNESLFIHSQSSNHMIRFREVDPRFSUMLPSSLWVTLLLFTCNKFUCFIQKLQRNLGSVIFTAGTVRTHGPNFQTDDESWWR

>Lectin32

GVKRFYTLFQKGKFVPKREHHUKMDGAQPSALDGITRKSAAVWAASCILMRSPNLDSKLLQVGQLPAALPSAPSLFFFFFFFSFPLSLFXFFFFFFFFALAHLSYQWHDUDSKNLPPUHEGRCIIQKHAEHLATACRSTLGQITHFATLSFFFSPSLFLULRMNCEQULQUTRVUSVSALRMSSUYNDPNAALVKSQILPUIUGKITLTSASRLESAGIGFSIPAAFCDRQUUMREGRCAVPHSRUQQHIFIRDDLSCPUCURTFPIGTYMFCYRURHKUIFFLIVHFSNVYYIYLCLRPGNKWSTDFGLVVWNHFSVVGSLTAAHLLAMTLIIFRSSSRTRTVLDCSLAFFALLSAVHRGMSDVNISKTCMLFLFMRFVVLDYCVESVSKSEIQFDVRLILIFFFFURVSNKVNSTVUIRRIIARNCMKAUKGMURNNSFVKCKYCGDNLKVTLSVTCGRUSEKUADSHSQQPAGCDAIFVPAISMPVGDRLLLFCCYLLFQRVSHEERERASGGCDEVKLMVRLTFUFNSQTPAQPLKVYIGLWSYKDPPPTPAFFLFLFFSSPNQPPSPFHLHCHPLSLNGGAGAACLPVCLTACQPSLFQTDQKHPKVANQLAAITPPELEDEAPIISRSLKSGLPQSAAKCCFTPAPSSCTCKSCFFFPPFFFFFFFFCPPRTFHLCFVPLRSKMALPRCAAGRHVFLTLLLLHWFGFGASSSMVNMRKVTHPTVRQPLAGEAALPCVFTLPNGSSSQSVRLIWTLVRLPAGGQDSPLEHVVLSAQGDVIKVNEAFSGRVRLPGYAADPLNATMEISSLRTNDSGTYHCQVVMGNKYERDAVLLVVYGVVFHYQAPSTRYALSFADARRACQENSAQMATSGQMWAAYYDGFAGCAAGWLDDQTVRYSVQSTELDCSGHKEYSAGVRNYGKRDPKELFDVYCFAKELDGEVFHASVPGRLSLSSASDRCVSLGGQLATVGQLYLAWRAGLHSCAPGWLADGSVHYPVSLPRPDCGGNEPGVHTVTLNITSDNTTALYDAYCYRGKVENTDTISQIYTSLWKPWSYLTDSSGASSGTESPPAITTKDESGNSPSNWTGLVDLEEEEESLLSVNASSDPWSSESYVSLQISPGQSIDWGEPFEPDSPEFLRPPVVPTPAEKKVISGIIESFWKPWNYLVGTGGGSEETTGTPKGTAGEEDVKKKKKEEEDEEEDRGNMTSTASPALFSWGSFLLSGTPKDDTTPANEELSAHVTPSSTASNNSLTTRTTISEEPPQTRPSPTSTPVPETTSSRGETWPRVDTETTTQSSDKREVTVMPRAGSRGGRGRGKKNRGEDRSRGEDKGRGDDEGSGEITAAEAKGEIQVSRRPVGTSKPRERSRSRERGHRKGQTTTTTTTSPTTTTEPSVMSTSQIVDLSASKLLFTVSTETQSSSIQADLDPISSTSSSSSSSSAPLPSSSPSASSSPSASSSSSSSLSSSASTSSSFWTSTSTPSPSPSPAHTAQPITTLSPSATSSPPLTLESGGPPAFPPADNQNSSANLEALLDWVPVESVAVLNISLDPPPEEEEEPAWSQTVGSGSPLPGNMEEEGSRFNVSNITLVPAVEPCVTNPCLHGGKCLPQGTGYSCYCPQGYAGENCEIDVDDCQSEPCENGGTCIDKIDSFLCLCLPSYGGDTCEKDIEGCEHGWRKFHGHCYRYFTHRHTWEDAEKDCREHSAHLSSVATANEQEFINGLGHDNAWVGLNDRTVEDDFQWTDGNELVYENWRESQPDNFFAGGEDCVVTIAHEDGKWNDVPCNYNLPYICKKGTVLCGTPPAVENAHLVGRRRSHYDIHAVVRYQCSEGFYQRHIPTARCRADGSWERPRIICAKSRRSHRYRRHHRRERRERRGHRRHGEGHKARENARSYDUTKUHAACKAEVLLVAPVAPASKAVISSVCFULLSSSURVRKVLLLSHIEVISLTAPALAVLMFCFPPPPPTFQLTSFNUGPTRTLTVTLDLTLPNLSLSRPPUTUVGQTGGRVCVRVCACMUERNVLGLUYENDSSSILLQSTRHUGKRARUPSALSFFVRASTRIAISISSTDMLFFFUYLLLFLSRIIYYFCUUKCSSVILYSFGETCSVFSNPVLCHMVSQYUHESVDCLUSUVLYILWCLLADVUSIAGPCPIGSCASSTLCDSVSGIDNLIDYMNEIIULHSLVPKLLKTSFHHCMATFTUWLECGDYYYFIFLVFDIUIVIYLFWCSYDVLLLFHSUURGKUYSI

>Lectin26

QPLKVYIGLWSYKDPPPTPAFFLFLFFSSPNQPPSPFHLHCHPLSLNGGAGAACLPVCLTACQPSLFQTDQKHPKVANQLAAITPPELEDEAPIISRSLKSGLPQSAAKCCFTPAPSSCTCKSCFFFPPFFFFFFFFCPPRTFHLCFVPLRSKMALPRCAAGRHVFLTLLLLHWFGFGASSSMVNMRKVTHPTVRQPLAGEAALPCVFTLPNGSSSQSVRLIWTLVRLPAGGQDSPLEHVVLSAQGDVIKVNEAFSGRVRLPGYAADPLNATMEISSLRTNDSGTYHCQVVMGNKYERDAVLLVVYGVVFHYQAPSTRYALSFADARRACQENSAQMATSGQMWAAYYDGFAGCAAGWLDDQTVRYSVQSTELDCSGHKEYSAGVRNYGKRDPKELFDVYCFAKELDGEVFHASVPGRLSLSSASDRCVSLGGQLATVGQLYLAWRAGLHSCAPGWLADGSVHYPVSLPRPDCGGNEPGVHTVTLNITSDNTTALYDAYCYRGKVENTDTISQIYTSLWKPWSYLTDSSGASSGTESPPAITTKDESGNSPSNWTGLVDLEEEEESLLSVNASSDPWSSESYVSLQISPGQSIDWGEPFEPDSPEFLRPPVVPTPAEKKVISGIIESFWKPWNYLVGTGGGSEETTGTPKGTAGEEDVKKKKKEEEDEEEDRGNMTSTASPALFSWGSFLLSGTPKDDTTPANEELSAHVTPSSTASNNSLTTRTTISEEPPQTRPSPTSTPVPETTSSR

GETWPRVDTETTTQSSDKREVTVMPRAGSRGGRGRGKKNRGEDRSRGEDKGRGDDEGSGEITAAEAKGEIQVSRRPVGTSKPRERSRSRERGHRKGQTTTTTTTSPTTTTEPSVMSTSQIVDLSASKLLFTVSTETQSSSIQADLDPISSTSSSSSSSSAPLPSSSPSASSSPSASSSSSSSLSSSASTSSSFWTSTSTPSPSPSPAHTAQPITTLSPSATSSPPLTLESGGPPAFPPADNQNSSANLEALLDWVPVESVAVLNISLDPPPEEEEEPAWSQTVGSGSPLPGNMEEEGSRFNVSNITLVPAVEPCVTNPCLHGGKCLPQGTGYSCYCPQGYAGENCEIDVDDCQSEPCENGGTCIDKIDSFLCLCLPSYGGDTCEKDIEGCEHGWRKFHGHCYRYFTHRHTWEDAEKDCREHSAHLSSVATANEQEFINGLGHDNAWVGLNDRTVEDDFQWTDGNELVYENWRESQPDNFFAGGEDCVVTIAHEDGKWNDVPCNYNLPYICKKGTVLCGTPPAVENAHLVGRRRSHYDIHAVVRYQCSEGFYQRHIPTARCRADGSWERPRIICAKSRRSHRYRRHHRRERRERRGHRRHGEGHKARENARSYDUTKUHAACKAEVLLVAPVGGUICIPLQSMTLUPFLLLTDSRGLTARRSHRYRRHHRRERRERRGHRRHGEGHKARENARSYDUTKUHAACKAEVLLVAPVAPASKAVISSVCFULLSSSURVRKVLLLSHIEVISLTAPALAVLMFCFPPPPPTFQLTSFNUGPTRTLTVTLDLTLPNLSLSRPPUTUVGQTGGRVCVRVCACMUERNVLGLUYENDSSSILLQSTRHUGKRARUPSALSFFVRASTRIAISISSTDMLFFFUYLLLFLSRIIYYFCUUKCSSVILYSFGETCSVFSNPVLCHMVSQYUHESVDCLUSUVLYILWCLLADVUSIAGPCPIGSCASSTLCDSVSGIDNLIDYMNEIIULHSLVPKLLKTSFHHCMATFTUWLECGDYYYFIFLVFDIUIVIYLFWCSYDVLLLFHSUURGKUYSI

>Lectin31

RTVLDCSLAFFALLSAVHRGMSDVNISKTCMLFLFMRFVVLDYCVESVSKSEIQFDVRLILIFFFFURVSNKVNSTVUIRRIIARNCMKAUKGMURNNSFVKCKYCGDNLKVTLSVTCGRUSEKUADSHSQQPAGCDAIFVPAISMPVGDRLLLFCCYLLFQRVSHEERERASGGCDEVKLMVRLTFUFNSQTPAQPLKVYIGLWSYKDPPPTPAFFLFLFFSSPNQPPSPFHLHCHPLSLNGGAGAACLPVCLTACQPSLFQTDQKHPKVANQLAAITPPELEDEAPIISRSLKSGLPQSAAKCCFTPAPSSCTCKSCFFFPPFFFFFFFFCPPRTFHLCFVPLRSKMALPRCAAGRHVFLTLLLLHWFGFGASSSMVNMRKVTHPTVRQPLAGEAALPCVFTLPNGSSSQSVRLIWTLVRLPAGGQDSPLEHVVLSAQGDVIKVNEAFSGRVRLPGYAADPLNATMEISSLRTNDSGTYHCQVVMGNKYERDAVLLVVYGVVFHYQAPSTRYALSFADARRACQENSAQMATSGQMWAAYYDGFAGCAAGWLDDQTVRYSVQSTELDCSGHKEYSAGVRNYGKRDPKELFDVYCFAKELDGEVFHASVPGRLSLSSASDRCVSLGGQLATVGQLYLAWRAGLHSCAPGWLADGSVHYPVSLPRPDCGGNEPGVHTVTLNITSDNTTALYDAYCYRGKVENTDTISQIYTSLWKPWSYLTDSSGASSGTESPPAITTKDESGNSPSNWTGLVDLEEEEESLLSVNASSDPWSSESYVSLQISPGQSIDWGEPFEPDSPEFLRPPVVPTPAEKKVISGIIESFWKPWNYLVGTGGGSEETTGTPKGTAGEEDVKKKKKEEEDEEEDRGNMTSTASPALFSWGSFLLSGTPKDDTTPANEELSAHVTPSSTASNNSLTTRTTISEEPPQTRPSPTSTPVPETTSSRGETWP

RVDTETTTQSSDKREVTVMPRAGSRGGRGRGKKNRGEDRSRGEDKGRGDDEGSGEITAAEAKGEIQVSRRPVGTSKPRERSRSRERGHRKGQTTTTTTTSPTTTTEPSVMSTSQIVDLSASKLLFTVSTETQSSSIQADLDPISSTSSSSSSSSAPLPSSSPSASSSPSASSSSSSSLSSSASTSSSFWTSTSTPSPSPSPAHTAQPITTLSPSATSSPPLTLESGGPPAFPPADNQNSSANLEALLDWVPVESVAVLNISLDPPPEEEEEPAWSQTVGSGSPLPGNMEEEGSRFNVSNITLVPAVEPCVTNPCLHGGKCLPQGTGYSCYCPQGYAGENCEIDVDDCQSEPCENGGTCIDKIDSFLCLCLPSYGGDTCEKDIEGCEHGWRKFHGHCYRYFTHRHTWEDAEKDCREHSAHLSSVATANEQEFINGLGHDNAWVGLNDRTVEDDFQWTDGNELVYENWRESQPDNFFAGGEDCVVTIAHEDGKWNDVPCNYNLPYICKKGTVLCGTPPAVENAHLVGRRRSHYDIHAVVRYQCSEGFYQRHIPTARCRADGSWERPRIICAKSRRSHRYRRHHRRERRERRGHRRHGEGHKARENARSYDUTKUHAACKAEVLLVAPVAPASKAVISSVCFULLSSSURVRKVLLLSHIEVISLTAPALAVLMFCFPPPPPTFQLTSFNUGPTRTLTVTLDLTLPNLSLSRPPUTUVGQTGGRVCVRVCACMUERNVLGLUYENDSSSILLQSTRHUGKRARUPSALSFFVRASTRIAISISSTDMLFFFUYLLLFLSRIIYYFCUUKCSSVILYSFGETCSVFSNPVLCHMVSQYUHESVDCLUSUVLYILWCLLADVUSIAGPCPIGSCASSTLCDSVSGIDNLIDYMNEIIULHSLVPKLLKTSFHHCMATFTUWLECGDYYYFIFLVFDIUIVIYLFWCSYDVLLLFHSUURGKUYSI

>Lectin28

QPLKVYIGLWSYKDPPPTPAFFLFLFFSSPNQPPSPFHLHCHPLSLNGGAGAACLPVCLTACQPSLFQTDQKHPKVANQLAAITPPELEDEAPIISRSLKSGLPQSAAKCCFTPAPSSCTCKSCFFFPPFFFFFFFFCPPRTFHLCFVPLRSKMALPRCAAGRHVFLTLLLLHWFGFGASSSMVNMRKVTHPTVRQPLAGEAALPCVFTLPNGSSSQSVRLIWTLVRLPAGGQDSPLEHVVLSAQGDVIKVNEAFSGRVRLPGYAADPLNATMEISSLRTNDSGTYHCQVVMGNKYERDAVLLVVYGVVFHYQAPSTRYALSFADARRACQENSAQMATSGQMWAAYYDGFAGCAAGWLDDQTVRYSVQSTELDCSGHKEYSAGVRNYGKRDPKELFDVYCFAKELDGEVFHASVPGRLSLSSASDRCVSLGGQLATVGQLYLAWRAGLHSCAPGWLADGSVHYPVSLPRPDCGGNEPGVHTVTLNITSDNTTALYDAYCYRGKVENTDTISQIYTSLWKPWSYLTDSSGASSGTESPPAITTKDESGNSPSNWTGLVDLEEEEESLLSVNASSDPWSSESYVSLQISPGQSIDWGEPFEPDSPEFLRPPVVPTPAEKKVISGIIESFWKPWNYLVGTGGGSEETTGTPKGTAGEEDVKKKKKEEEDEEEDRGNMTSTASPALFSWGSFLLSGTPKDDTTPANEELSAHVTPSSTASNNSLTTRTTISEEPPQTRPSPTSTPVPETTSSRGETWPRVDTETTTQSSDKREVTVMPRAGSRGGRGRGKKNRGEDRSRGEDKGRGDDEGSGEITAAEAKGEIQVSRRPVGTSKPRERSRSRERGHRKGQTTTTTTTSPTTTTEPSVMSTSQIVDLSASKLLFTVSTETQSSSIQADLDPISSTSSSSSSSSAPLPSSSPSASSSPSASSSSSSSLSSSASTSSSFWTSTSTPSPSPSPAHTAQPITTLSPSATSSPPLTLESGGPPAFPPADNQNSSANLEALLDWVPVESVAVLNISLDPPPEEEEEPAWSQTVGSGSPLPGNMEEEGSRFNVSNITLVPAVEPCVTNPCLHGGKCLPQGTGYSCYCPQGYAGENCEIDVDDCQSEPCENGGTCIDKIDSFLCLCLPSYGGDTCEKDIEGCEHGWRKFHGHCYRYFTHRHTWEDAEKDCREHSAHLSSVATANEQEFINGLGHDNAWVGLNDRTVEDDFQWTDGNELVYENWRESQPDNFFAGGEDCVVTIAHEDGKWNDVPCNYNLPYICKKGTVLCGTPPAVENAHLVGRRRSHYDIHAVVRYQCSEGFYQRHIPTARCRADGSWERPRIICAKSRRSHRYRRHHRRERRERRGHRRHGEGHKARENARSYDUTKUHAACKAEVLLVAPVGGUICIPLQSMTLUPFLLLTDSRGLTARRSHRYRRHHRRERRERRGHRRHGEGHKARENARSYDUTKUHAACKAEVLLVAPVAPASKAVISSVCFULLSSSURVRKVLLLSHIEVISLTAPALAVLMFCFPPPPPTFQLTSFNUGPTRTLTVTLDLTLPNLSLSRPPUTUVGQTGGRVCVRVCACMUERNVLGLUYENDSSSILLQSTRHUGKRARUPSALSFFVRASTRIAISISSTDMLFFFUYLLLFLSRIIYYFCUUKCSSVILYSFGETCSVFSNPVLCHMVSQYUHESVDCLUSUVLYILWCLLADVUSIAGPCPIGSCASSTLCDSVSGIDNLIDYMNEIIULHSLVPKLLKTSFHHCMATFTUWLECGDYYYFIFLVFDIUIVIYLFWCSYDVLLLFHSUURGKUYSI

>Throm60

KTLQYLLSVWQRPAGVACKLWSRGPSQSICRDDITRSPLVHCMSKLELPQQFHSRPSAVPGQQSRQQSFSLPRFWIWTPIRRPAMAASKPTTTSFSPRGUGAPUWPRSSRLRRIUYKTTSAAADPTWSGSSAPPSSQTDATFUCHRRSEGGRKPFAETPTWTGSGLGRPASSRHSLRYRQVSPRLSSPDGACSDSMLVLV

SLLLTGLGCLLAAPTGREVYRMPQSALKALSDRGTVVLEAAMTSALSALDRASSERSRVEAGCRGCAPCLFQDCGQPSGSCSSPANALTEPSCDVISKAQKLQDEGERSWALSQACAFYQHRCQAGNSDKESCIRIMGESCSARVLQCSLLNTMQNLEPATQTHVQAVCGQRSSSARNVTQPRSRIVGGSPAPPGSWPWLVNLQLDGGLMCGGVLVESSWVVTAAHCFAGSRSESYWTAVVGEFDITKTDPDEQVLKVNRIVPHPKFNPKTFNNDIALVELTSPVVLSDRVTPVCLPSGMEPPTGSPCLVAGWGSLYEDGPSADTVMEAKVPLLPQNTCKNALGKELVTNTMLCAGYLSGGIDSCQGDSGGPLIYQDRISGRFQLHGITSWGDGCGEKGK

PGVYTRVSAFSDWIQVEIQKSFGSREPTCPELLKTTEMTEEEQRSEFITLCRFYTLSCPTGQSGGSCSRMAEDKCLTRFKKCQLRSFLQTLLDLLQSAEDYIRDKVDLAFFTQTLPQLVEHIYSTAFPHSRDKRDVVPIEEQQGRPTEQGSPSVPPALFKALGPSVEDWENYLVTIAEDMEARPNDTSAENSFESSRREEKRFLQAGDDSVLRLQKEYQNMISALRSKLDSKAAPPSLRMDPSDPHQRSSDPLFLPTSSPRLQIDPGPSSSPKSQKPWSLLSALMSEIEKLKTASETLTSDESQNEAFVTSTEGTWDKAVDLGELAALVSTAVLPIHMAKTTSQPHTLHASVPFSLRRRQRSILRKRQTPGPGGKVCPGVRETSQQVSQVRASYHWVLNIPNKNLHMSFQEVLVDLSSKNDRGLYQARVRAVVGGRPLTFYSLVGLENESFYRSVPRIISLALEALKTURRRNHRKDDHKNRSARRPRSHRRGTQTQTVASYRGAYPRSRIPUPFREPDCGSAEKLUMYPASHQLALTTSLSNLUIYPSD

KLFDSHRHKVFCKGUASCEGHGLLIMFISYLFFEKKNLLYFCNSKIYMNDVRIKLLLTPWHLGGLSFQSECLMGDIEMSCQGCCYSGSILHQRGGGLPPAREHULAGTVTGHUTQAANHDINRAHAVYIYNRLPSSYQDEESUKDAKSLSIINWQKMVYTHTHKQTNKTLPTFGKHSRLRFGDVLNKYVCDAQADSUQRF

PNVQATSRNLHSARSAFIDMCLQMSHTYHLFSQLEPFVLLFHTYYPSSSPALFFFFFVYKRLCAGLCANFVPCLFYSLKINARLANAVCVCFPAHVELUFQGCIFSC

>CcAMP2

LSESSLRPRDUTHHRDWASGQSYSRGGLIUVEVATMFLNKVLRAGMRCGVVRLQSSSATAAEAKISGEKTAAATGFSFEFTDQQREFQQLARKFAREEIVPQAAEYDRTGEYPLPIIKKAWELGLMNGHIGEEYGGMGLSIFDNCLITEELAYGCTGVQTAIEANSLGQMPVIIAGNETQKKKYLGRMTEEPLMCAYCVTEPGAGSDVAGIKTRAVKTGDEYVVNGQKMWITNGGKANWYFLLARTHPDPKCPTNKAFTGFIVDADTPGIQIGRKERNMGQRCSDTRGITFEEVRIPKENVLLGEGAGFKIAMGAFDKTRPPVAAGATGLAQRALDEATAYALERKTFGKVIAEHQAVSFLLAEMVMKVELARMAYQRAAWEVDRGRRNTYYASIAKAFAGDIANQVASDAVQVFGGNGFNSEYPVEKLMRDAKIYQIYEGTAQIQRLIVAREHLAKAKKUAVCVCVYTCVKTQFKULIFFFAFNSPLSPVALLNGVQIGGVPMULUPFKQMTHMPLECELKYESLMYKCQTEKNHVLSVLLQTLSVSVTCSHKALMEAKKLPCGVYNVILLLVNPTPDVNHUTMHFUSSTTSASLLEHNUHFNGKFUFVUIKHFWPNLCRAVAWDMRIVVGFQFLQUWLLPATSIMGVHERQLSALIMSTMRFLGCCDRPSRGUHLLNNEQQHFSPVFITSUATKDFHFIPCAFSGAUTCFAUKUMPYHKMSKQKVFLPCVFSCAFPCSLFEUMFFRKLDNURAFLQCAFUYVIPSLPFEUMFYRTLSNUSVFLAHVFSCGLUSVPFERMFFHKQSRUNVLDAFVKFSSSNLQSLRRFYKVLUSRQYLAMELRGHRVVVLLSGHHLDCEDEAVASQEKVHFHCLHPAURIESVVSCRHHLAPLPSUHGGAVDASLLPLALPLUQASAVETPPPPNWSDCVAAERGVLPDEQPAPGHLQDTSNUVWINUSUIVLVAAKPLLSFRPSVTVVDNHRRRAGPCMNMRQISAWVLKUKTSSLVLTPPTLSCWTNMQTTSLLMAPKRMTMSTRFLSTCEUAASTGVURUWTUCLSCRAUTARRLWTSLRRVSTNAAALAPALAMTHTCSTRSALCRFCACMTAWTHWTWTKLWSTSKGCSRLTDHLQEINGVKSTHDFPSAQWPPFRYWARWTPLTWTRRWSSSCPVUTSTVVSAAGPGPSLTPVRFTAARASCRURASCTSSTPTCUAGGSARDSCRQAASMGGPRSFRTCATPGGFWHLSESSARFTGSTRTSCAPSSWPAKTRRREALPTDRAIWWTRSTLCLVSPDSPCWEMRRSRQUILYCACPRTCCRSSAUSRSSSANPRMLICYTCTDTLRNCPHUTFLSTKLTIKQYUNPISHKDSSFHPFPKTANLQYVDQTVTUIMKCELSAPFYFIFCIKIISCEYEYVTYLFPSGNKCLDVLLRUCCFCSVMQRUMALLP

>Chem14

LKWHLCACVCVCVCVCVSKTGSYSFUUAVYASDKHAQFPLUUESSYDITUQLRRAPCESDGLGSPVLLRUTTQCKSSPYLPHTHSVHIPAEHIRYSGIQADGRRFLTVVSGLFAHQFHRRLSSNHRRTTSWKSSVVPAPKMKLNLILLCLSLWMTSVLTTHGPVSDCCPGLSSTTVSLNRIKSYVIQSVGACSFKAVVFYTHGRKRICADPNSCWTQRAMFKVDGGALRRKEESNDKSTGASGATVPCTPSRNSQGKKRRRGKLLRMKKGKTRVUIYLNECPKUMWYNNDLCAVCKSTHPSIHFQIRLSSHGSRHVLEPSPALFEKUTEYTLNCLPANRRDTHRRTTICTHNHTUGQFRVSHUTATHVFGMWEETHAGTIHLSSULAIYRACULASYLHNTGSYLHVSSLMYYVLCCIILGCTLLQCTLQARAALLHSILYNTIQUFTVLYQLCKSVECKTEKESLIPIKKKYHLNLRRQFQPTCNGSMTVDSSEFTKPPIKLFQVRUVVLKVVHFMVALNSANVGKLUVHITRKPQIUSSTUSHTLPHKCSIGWWVGGGGXXXGPIIVIDVCIKENPFTSSKLHUEAAVLFSHIVSKYVWTGSPLNLHLGSQKTSVTQLRTRTYGGCGGLCKTSEYHLSHLWSKTDRISFPPANEGNALAYLSNSSKNGRISDLTAGDSSIDFLPRNVFQKPFFIURALADIGLVPTLLKIQTKKKHFLNLHGLFLVYMERLYURGDPFFFFKKGVFUCRKYEDITTVHVKRCARFRCVPSFWAUQFFPSRYSMLVWVHDLFGSAQILLPFNVTKLRNKYKRNIRWRKVNUNLSNIIFLNLKURDIVHGVFGLLLFFTILHFHATNAPPGLAFGHICLKYFPCNFSLVIYCIIEIIYLICIQQNRCTVENISNVTYTTACKAQEGGPWMUYLVTRCRRTCVSKILRQQLGNPPEKKIWGQKKIKKRTRQINNTSSHMUANLTUHLPRAKAPGTMTATRKRPTKLRAVIAHVGRNQVUSGCLRCGLLKPANVVKYVNLCHLRVWHDAPTVAHTPGNNQTFFLPLKTGQNSPWPSFRPRARVSMAYFSIAGVSCQSTFSCNYNTPQFVNNSQYFILIFAVNPMFQLCAUPEKQIHFLALFFFVCLFVCAFUGAFSRVGALDTELDSSQSUVTNFRTWDLRPFIGFLENNKTHVLYUTTKTCHHCHGRSELPQREEMKPRRTLSDIKAKRGEYLSRKCMNAVSQYQRANIAKSLSNDPHALHYFWGVIRDFLDNHAMFLRFNKKKRIHYSTVUCLWNLKTQFRATVWHSLCWCEEPVTVFYLYLSQLVULTRPWTFRIYDSKQVERAHFLKSPSIVLRCCVAAATKQFFTYUUURMTFSTAIFQQRLFNSYIETQYLPHLKSSSSSSSYFQAIADCIYLDLLLKAGEKUTRPWVFFLREVTLTEQLAVCKRIQCTNKVSPKTPPLFVVTUSLLKGSARCTHVVVSTTFSCMTACRIIKVHRDIFYLUCMKHEKKKSPLSSRHRLLVEDDEAKHPVRCIAVPYSLHUTIPKUSCARFYAERRGARQPKUFCLNSFFHMKSSDSYYFPGTGULUVSNFTISIISADLWNPMSHFENVSPDLLSAVVSISPSDUUSLSVCHGPEKUNESLFIHSQSSNHMIRFREVDPRFSUMLPSSLWVTLLLFTCNKFUCFIQKLQRNLGSVIFTAGTVRTHGPNFQTDDESWWR
