# Supplementary material for: High-Throughput Identification of Putative Antimicrobial Peptides from Multi-Omics Data of the Lined Seahorse (Hippocampus erectus)
Source: Mar Drugs. 2019 Dec 29;18(1):30. doi: 10.3390/md18010030 (PMC7024384; doi:10.3390/md18010030)
Supplement: Supplementary file 1 [file marinedrugs-18-00030-s001.zip › Table S1.docx]

Table S1. Statistics of spectra, peptides and proteins in each proteome dataset.

| **Sample** | **Total spectra** | **Identified spectra** | **Identified peptides** | **Identified proteins** |
| --- | --- | --- | --- | --- |
| MS.F.20^1^_1 | 363,995 | 94,615 | 21,737 | 4,235 |
| MS.F.20_2 | 373,857 | 109,204 | 28,067 | 5,247 |
| MS.F.20_3 | 366,213 | 81,879 | 21,776 | 4,337 |
| MS.F.1^2^_1 | 34,050 | 16,769 | 6,061 | 934 |
| MS.F.1_2 | 34,092 | 17,325 | 6,039 | 882 |
| MS.M.20^3^_1 | 331,294 | 66,976 | 16,916 | 3,530 |
| MS.M.20_2 | 316,052 | 6,1061 | 16,008 | 3,641 |
| MS.M.20_3 | 314,000 | 68,620 | 16,363 | 3,695 |
| MS.M.1^4^_1 | 33,301 | 16,067 | 5,339 | 766 |
| MS.M.1_2 | 33,449 | 17,141 | 5,589 | 820 |
| DIA_F^5^ | / | / | 10,072 | 1,864 |
| DIA_M^6^ | / | / | 10,747 | 1,864 |

^1-6^See more details in Section 2.4.
